# Stabilizing sub-2 nm δ-Bi_2_O_3_ via strong lanthanide-oxide-support interaction for durable CO_2_ electroreduction to formate

**DOI:** 10.1038/s41467-026-71855-5

**Published:** 2026-04-24

**Authors:** Qianmin Wu, Cui Li, Yuxuan Wu, Qing Liang, Xuyu Lv, Yanhong Li, Chang Wang, Mengjie Wu, Lichun Kong, Ji-Qing Lu, Wei Zhang, Zhengquan Li, De-Li Chen, Jing Zhou, Fa Yang

**Affiliations:** 1 Key Laboratory of the Ministry of Education for Advanced Catalysis Materials, Zhejiang Key Laboratory of Advanced Catalysis and Adsorption Materials, College of Chemistry and Materials Science, Zhejiang Normal University, Jinhua, China; 2 Zhejiang Institute of Photoelectronics, Zhejiang Normal University, Jinhua, China; 3 Electron Microscopy Center, School of Materials Science & Engineering, Key Laboratory of Automobile Materials MOE, Jilin University, Changchun, China

**Keywords:** Electrocatalysis, Carbon capture and storage, Electrocatalysis

## Abstract

Stabilizing metal oxides is a prerequisite for elucidating their intrinsic mechanistic roles and sustaining high electrocatalytic activity. Here, we synthesize a high-temperature-phase La_2_O_3_-socketed sub-2 nm δ-Bi_2_O_3_ heterojunction (δ-Bi_2_O_3_/La_2_O_3_) that suppresses Bi^3+^ reduction to metallic Bi, achieving ≥95% formate Faradaic efficiency for ~200 hours in industrial-level electrolyzers. Electronic structure analyses reveal that strong electrostatic interactions between δ-Bi_2_O_3_ and La_2_O_3_ drive oxygen migration to the interface, contracting δ-Bi_2_O_3_ domains and enhancing La–Bi *d-p* orbital hybridization. This structural relaxation stabilizes interfacial Bi–O–La linkages and electron-deficient Bi_2_O_3+x_ species under cathodic potentials, as confirmed by in situ X-ray absorption spectroscopy. Pourbaix diagrams and in situ infrared spectroscopy demonstrate that La_2_O_3_ promotes water dissociation to form a hydroxylated δ-Bi_2_O_3_ surface under working potentials, enhancing protonation propensity. Consequently, the energy barrier for the rate-determining step (*CO_2_ → *HCOO) is lowered to +0.15 eV on δ-Bi_2_O_3_/La_2_O_3_, significantly lower than the +0.83 eV barrier on pristine δ-Bi_2_O_3_. This work establishes a sub-nanoscale oxide/oxide heterojunction strategy to stabilize high-valent metal sites, enabling sustainable electrochemical conversion.

## Abstract (web summary)

Stabilizing metal oxides is a prerequisite for efficient electrochemical CO2 reduction. Here, the authors report a La2O3-socketed δ-Bi2O3 heterojunction that impedes Bi2O3 reduction to metallic Bi, achieving high-efficiency formate production in industrial-level electrolyzers.

## Introduction

The electrocatalytic CO_2_ reduction reaction (CO_2_RR) powered by renewable electricity plays a vital role in storing intermittent renewable energy. Among the CO_2_RR products, formate (or formic acid) is one of the most economically viable, serving as a key chemical precursor in pharmaceutical and chemical industries. Formate can also function as a liquid hydrogen carrier for fuel cells due to its high energy density, storability and transportability^1–4^. Bismuth-based materials, especially bismuth oxide (Bi_2_O_3_), have drawn significant attention because the Bi−O coordination structure favors the initial electron transfer and subsequent hydrogenation steps in the selective formate production^5,6^. Bi_2_O_3_ exists in six crystalline phases: α, β, γ, ε, ω, and δ. Among them, the cubic fluorite-type δ-Bi_2_O_3_ phase, which contains approximately 25% oxygen vacancies, exhibits an optimal conductivity in theory. However, δ-Bi_2_O_3_ is a high-temperature phase that is stable only between 730 and 830 °C, and it readily transforms into the α- or β-phase upon cooling, which limits its practical use in electrochemical reactions^7,8^. Moreover, another fact that cannot be ignored is that prolonged cathodic polarization inevitably reduces Bi_2_O_3_ to metallic Bi, which not only causes severe structural degradation but also promotes the competing hydrogen evolution reaction (HER)^9,10^.

Strong metal oxide–support interaction (SMOSI) is recognized as an effective strategy to stabilize the oxidation state of supported metal species^11^. However, limited by the short-range nature (≤ 5 nm, generally), supported large nanoparticles are supposed to markedly attenuate the SMOSI effect^12^. An optimal approach to leveraging the SMOSI effect is to stabilize δ-Bi_2_O_3_ as highly dispersed sub-nanoparticles (≤ 2 nm). Unfortunately, the synthesis of sub-nanometer metal oxide particles is challenging due to their high surface energy, which shows a high propensity for agglomeration or excessive growth^13^. Reports on the synthesis of Bi_2_O_3_ sub-nanoparticles remain scarce, particularly for the high-temperature δ-Bi_2_O_3_ phase. Lanthanide metal oxides (LMOs) with high thermodynamic stability and strong oxophilicity show huge potential as supports or dopants due to their characteristic electronic structure of empty 4*f* and unfilled 5*d* orbitals^14^. Moreover, LMOs can effectively suppress the migration and coalescence of supported metal species owing to their large ionic radii and strong metal–support adhesion energy^15^, thereby enabling the stabilization of sub-nanoparticles even under high-temperature sintering conditions.

In this study, we employ lanthanum oxide (La_2_O_3_), a representative irreducible oxide, as a support to construct a heterostructure comprising sub-2 nm δ-Bi_2_O_3_ nanoparticles anchored on hollow La_2_O_3_ microspheres, which enables CO_2_ electroreduction to formate with a faradaic efficiency exceeding 95% during 200 h of continuous electrolysis in both H-type cell and industrial-level electrolyzers. Scanning transmission electron microscopy (STEM) and in situ Raman spectroscopy confirm the formation of robust Bi–La oxide heterointerfaces featuring interfacial Bi–O–La linkages, which underpin the catalyst’s enhanced stability and selectivity. Theoretical calculations combined with in situ X-ray absorption spectroscopy (XAS) further reveal that the strong interfacial charge transfer and redistribution drive the spontaneous migration and condensation of oxygen atoms from the surface to interfacial regions, thereby inducing lattice contraction in the δ-Bi_2_O_3_ domains. This structural relaxation stabilizes an oxygen-rich Bi_2_O_3+x_ moiety and enhances *d–p* orbital hybridization across the Bi–O–La interface, thereby effectively suppressing the reduction of Bi_2_O_3 + x_ to metallic Bi under CO_2_RR conditions. The Pourbaix diagram and in situ infrared spectroscopy further confirm that the hydroxylated δ-Bi_2_O_3_/La_2_O_3_ surface is thermodynamically favored under operating potentials, as the La_2_O_3_ support promotes water adsorption and dissociation, thereby facilitating surface protonation. As a result, the formation of the key *HCOO intermediate is kinetically favored on δ-Bi_2_O_3_/La_2_O_3_, with a energy barrier of only +0.15 eV, compared to +0.83 eV on pristine δ-Bi_2_O_3_. This study establishes a generalizable lanthanide oxide heterojunction platform to stabilize sub-nanometer metal sites in high oxidation states, enabling durable and scalable CO_2_ electrolysis.

## Results

**Fig. 1:**
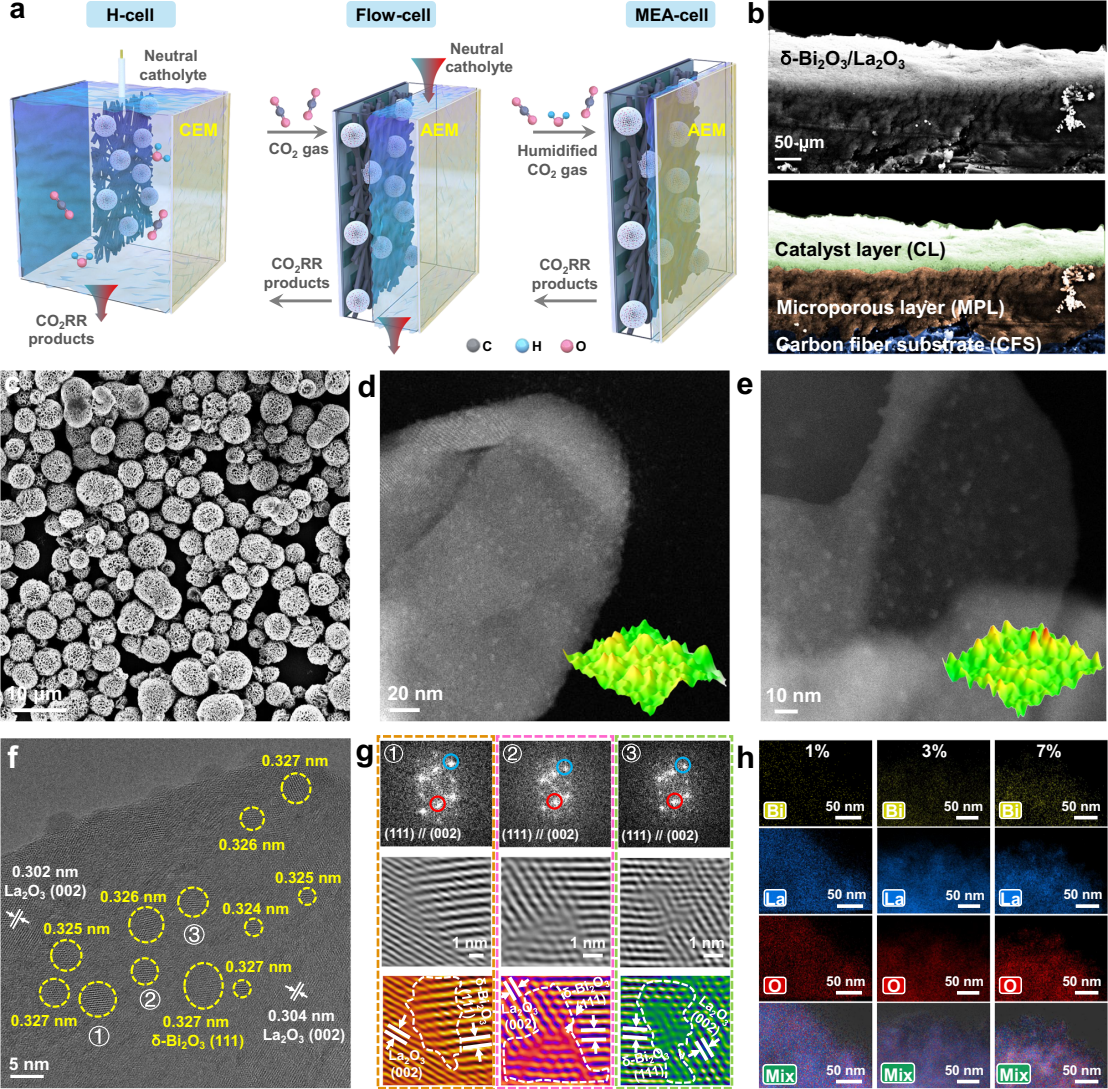
Morphology and structural characterizations of δ-Bi_2_O_3_/La_2_O_3_. **a** Schematic illustration of the electrolytic cell configurations and **b** the corresponding cross-sectional SEM images of the cathode GDE. **c** Typical top-view SEM images. **d**, **e** Aberration-corrected HAADF-STEM images, the inset is 3D atom-overlapping Gaussian function fitting mapping. **f** HRTEM image and **g** corresponding FFT analysis of the selected region. The spots highlighted by blue and red circles in (**g**) correspond to the (002) plane of La_2_O_3_ and the (111) plane of δ-Bi_2_O_3_, respectively. **h** EDS element mapping of δ-Bi_2_O_3_/La_2_O_3_ with different Bi content.

The δ-Bi_2_O_3_/La_2_O_3_ heterostructure with δ-Bi_2_O_3_ sub-nanoparticles anchoring on the La_2_O_3_ microspheres are synthesized via a two-step hydrothermal-calcination method at 700–1000 °C, as schematically illustrated in Supplementary Fig. 1. The experimental conditions for both the hydrothermal and calcination steps are optimized based on the scanning electron microscopy (SEM) images (Supplementary Fig. 2). The δ-Bi_2_O_3_/La_2_O_3_ with different Bi contents (0.8, 2.7, 7.1, 9.6, and 15.2 Bi at.% quantified by ICP-AES, denoted as 1, 3, 7, 10, and 15% Bi_2_O_3_/La_2_O_3_) are prepared for control. Additionally, pure La_2_O_3_ microspheres (Supplementary Fig. 3) and Bi_2_O_3_ powder (Supplementary Fig. 4) are also prepared using a similar synthesis procedure (see “Methods” for details). In this work, to systematically evaluate the CO_2_RR activity and stability of the δ-Bi_2_O_3_/La_2_O_3_ catalysts, electrolysis tests are conducted using three types of electrolyzers equipped with gas diffusion electrodes (GDEs) as shown in Fig. 1a. The cross-sectional SEM image in Fig. 1b shows a well-defined, densely packed catalyst layer on the carbon fiber-supported microporous layer (MPL). The detailed architecture of the gas diffusion layer (GDL) can be seen in Supplementary Fig. 5. Typical top-view SEM images (Fig. 1c and Supplementary Figs. 6, 7) show that the δ-Bi_2_O_3_/La_2_O_3_ exhibit homogeneous porous microsphere morphology, and an obvious contrast between the dark edge and the light center is observed as imaged by transmission electron microscopy (TEM) images in Supplementary Fig. 6c, f, i, showcasing a hierarchically hollow property. The N_2_ adsorption-desorption isotherms and pore size distribution (Supplementary Fig. 8a) reveal that the 7% δ-Bi_2_O_3_/La_2_O_3_ possesses abundant mesopores (~2.39 nm) and a high BET surface area of 258.3 m^2^ g^−1^. Such a porous structure is highly favorable for CO_2_ adsorption, as evidenced by the CO_2_ uptake in Supplementary Fig. 8b^16^. The aberration-corrected high-angle annular dark-field scanning transmission electron microscopy (HAADF-STEM) is further conducted to identify the dispersion state of δ-Bi_2_O_3_ in the La_2_O_3_ support. The isolated bright dots represent well-dispersed sub-2 nm Bi_2_O_3_ nanoparticles in the 7% δ-Bi_2_O_3_/La_2_O_3_, with an average particle size of ~2 nm (Fig. 1d, e, and Supplementary Fig. 9). The distribution of La_2_O_3_-socketed sub-2 nm Bi_2_O_3_ nanoparticles is further confirmed by the 3D atom-overlapping Gaussian function fitting mapping of the selected area (insets of Fig. 1d, e), where the highest peaks are assigned to Bi_2_O_3_ particles. The HAADF-STEM images of 1, 3, 10, and 15% δ-Bi_2_O_3_/La_2_O_3_ samples are also displayed in Supplementary Figs. 10–12. The areal density of sub-2 nm Bi_2_O_3_ nanoparticles in the 1 and 3% δ-Bi_2_O_3_/La_2_O_3_ samples is lower than that in the 7% δ-Bi_2_O_3_/La_2_O_3_ sample. In the 10 and 15% Bi_2_O_3_/La_2_O_3_ samples, the highly active sub-2 nm Bi_2_O_3_ particles begin to agglomerate into larger particles (~8 nm) as the Bi loading increases, resulting in a coexistence of sub-2 nm and ~8 nm particles. This agglomeration reduces the density of highly active sub-nanoparticles and may diminish the Bi_2_O_3_/La_2_O_3_ interfacial area, both of which are critical for improving catalytic activity and stability. Subsequently, the structural and chemical properties of pure Bi_2_O_3_ and the δ-Bi_2_O_3_/La_2_O_3_ samples are characterized by X-ray diffraction (XRD), Raman spectroscopy, and X-ray photoelectron spectroscopy (XPS). The XRD pattern (Supplementary Fig. 4d) reveals that the diffraction peaks of pure Bi_2_O_3_ can be indexed to a mixture of cubic δ-Bi_2_O_3_ (JCPDS: 01-071-0466) and monoclinic α-Bi_2_O_3_ (JCPDS: 01-071-0467). The Raman spectrum of pure Bi_2_O_3_ powder (Supplementary Fig. 4e) exhibits characteristic peaks at ~115, ~215, and ~316 cm^−1^, assigned to Bi–O vibrational modes of α-Bi_2_O_3_^17^, while the broad Raman band at ~620 cm^−1^ corresponds to the *T*_2g_ mode of δ-Bi_2_O_3_^18^, which arises from symmetric Bi–O stretching vibrations. The above XRD and Raman results demonstrate that pure Bi_2_O_3_ undergoes a partial phase transition from the δ-cubic to the α-monoclinic phase during cooling, consistent with previous literature reports. On the contrary, the XRD patterns (Supplementary Fig. 13) reveal that the diffraction peaks of the Bi_2_O_3_/La_2_O_3_ samples can be well indexed to cubic δ-Bi_2_O_3_ (JCPDS: 01-071-0466) and hexagonal La_2_O_3_ (JCPDS: 01-074-2430), with no α-Bi_2_O_3_ phase detected. Notably, the sub-2 nm Bi_2_O_3_ nanoparticles in the 1, 3, and 7% Bi_2_O_3_/La_2_O_3_ samples lack long-range crystalline order, and consequently, no Bragg reflections attributable to Bi_2_O_3_ are discernible in the XRD pattern. Additionally, the Raman spectra of δ-Bi_2_O_3_/La_2_O_3_ (Supplementary Fig. 14) exhibit only the δ-Bi_2_O_3_ *T*_2g_ mode at ~620 cm^−1^ and La_2_O_3_-related peaks at ~110, ~189, and ~449 cm^−1^. This suggests that the δ-Bi_2_O_3_ phase, which is typically stable only at high temperatures, is stabilized at room temperature (25 °C) through strong interfacial interaction in the constructed δ-Bi_2_O_3_/La_2_O_3_ heterostructure^19^. Therefore, Bi_2_O_3_ in Bi_2_O_3_/La_2_O_3_ is recognized as the δ-Bi_2_O_3_ phase. With increasing Bi loading from 1 to 7%, the Raman peaks shift to lower wavenumbers, likely due to the strong interaction between La_2_O_3_ and δ-Bi_2_O_3_^20^. This is further supported by a distinct Raman band at 390 cm^–1^ (Supplementary Fig. 14), which arises from interfacial Bi–O–La bonding^21,22^. We also performed XPS measurements. As shown in Supplementary Fig. 15, the peaks of La 3*d*_5/2_ 833.3/836.3 eV and La 3*d*_3/2_ 850.4/854.7 eV are attributed to La^3+^ species^23,24^, while the two characteristic peaks of 158.5 and 164.2 eV belong to Bi^3+^ species^25,26^. For δ-Bi_2_O_3_/La_2_O_3_, the La^3+^ 3*d* binding energy is shifted to lower values compared to that in pure La_2_O_3_, while the Bi^3+^ 4*f* binding energy is shifted to higher values compared to that in pure Bi_2_O_3_, suggesting an electron transfer from δ-Bi_2_O_3_ to La_2_O_3_^25,27^. Consistent with the Raman results, the increasingly negative shift in the Bi 4*f* binding energy with increasing Bi loading from 1 to 7% suggests enhanced electron transfer from La_2_O_3_ to δ-Bi_2_O_3_, indicative of stronger interfacial electronic coupling. Moreover, compared to pure Bi_2_O_3_ and La_2_O_3_, the O 1*s* XPS spectrum of the δ-Bi_2_O_3_/La_2_O_3_ (Supplementary Fig. 16) can be fitted with two peaks at 528.7 and 531.1 eV, assigned to lattice oxygen in Bi–O–La and oxygen vacancies (O_vac_), respectively^22,28^. The presence of the low-binding-energy component at 528.7 eV also supports the formation of Bi–O–La interfacial bonds.

Furthermore, the high-resolution TEM (HRTEM) images of 7% δ-Bi_2_O_3_/La_2_O_3_ (Fig. 1f and Supplementary Figs. 17a, 18a) reveal lattice fringes with spacings of ~0.326 and ~0.302 nm, assigned to the δ-Bi_2_O_3_ (111) and La_2_O_3_ (002) planes, respectively, as compared with that of pure La_2_O_3_ (~0.309 nm, Supplementary Fig. 3e) and pure Bi_2_O_3_ (~0.337 nm, Supplementary Fig. 4c). This compressive interfacial strain implies a strong interaction between the two phases^20^. Besides, as shown in Fig. 1g and Supplementary Figs. 17b, 18b, the selected area FFT analysis of the enlarged HRTEM images confirms the presence of δ-Bi_2_O_3_/La_2_O_3_ heterointerfaces. The disordered atomic arrangement at the 7% δ-Bi_2_O_3_/La_2_O_3_ interface is highlighted by white circled regions. Notably, the HRTEM images also reveal a partially distorted lattice (Supplementary Fig. 19), also supporting the formation of interfacial Bi–O–La linkages between the δ-Bi_2_O_3_ (111) and La_2_O_3_ (002) planes. This structural distortion likely arises from the lattice mismatch between the two phases and the scramble between Bi and La for oxygen^29^. To further determine whether interfacial Bi and La atoms interdiffuse, we perform X-ray absorption spectroscopy (XAS) measurements and simulations. The La L_3_-edge XANES spectra show that the La absorption edge position in the δ-Bi_2_O_3_/La_2_O_3_ is essentially identical to that of pure La_2_O_3_ (Supplementary Fig. 20), demonstrating that Bi is not significantly incorporated into the La_2_O_3_ lattice. Notably, the fitted La–O bond lengths are shorter in δ-Bi_2_O_3_/La_2_O_3_ than in pure La_2_O_3_, further suggesting a lattice contraction in the La_2_O_3_ support (Supplementary Figs. 21, 22, and Supplementary Table 1). We further carried out theoretical XAS simulations for Bi-doped La_2_O_3_ and pure α-, β-, and δ-Bi_2_O_3_ to identify the local coordination structure of Bi in δ-Bi_2_O_3_/La_2_O_3_ by comparing the simulated and experimental XANES spectra. As shown in Supplementary Fig. 23, the simulated Bi L_3_-edge XANES spectrum for Bi-doped La_2_O_3_ does not match the experimental spectrum of the δ-Bi_2_O_3_/La_2_O_3_ sample, whereas the simulated spectrum for pure δ-Bi_2_O_3_ shows good agreement, also verifying negligible incorporation of Bi into the La_2_O_3_ lattice. Besides, the simulated Bi L_3_-edge XANES spectra for α-Bi_2_O_3_ and β-Bi_2_O_3_ do not match the experimental spectrum, further distinguishing that the Bi_2_O_3_ in the composite exists as the δ-phase. Subsequently, to determine whether La is incorporated into the δ-Bi_2_O_3_ lattice, we perform La L_3_-edge XANES simulations based on these structural models—La-doped α-Bi_2_O_3_, La-doped β-Bi_2_O_3_, La-doped δ-Bi_2_O_3_, and undoped La_2_O_3_. As shown in Supplementary Fig. 24, the simulated La L_3_-edge XANES spectra for La-doped α-, β-, and δ-Bi_2_O_3_ show poor agreement with the experimental spectrum of the δ-Bi_2_O_3_/La_2_O_3_ sample, whereas the simulated spectrum for pristine La_2_O_3_ closely matches the experimental data, also indicating that La is not significantly incorporated into the δ-Bi_2_O_3_ lattice. Consequently, large-scale interdiffusion of Bi and La atoms is unlikely to occur. Finally, the EDS elemental maps shown in Fig. 1h and Supplementary Fig. 25 indicate that Bi, La, and O are evenly distributed in the δ-Bi_2_O_3_/La_2_O_3_ composites across different Bi loadings. Overall, the above characterization results demonstrate the successful construction of a La_2_O_3_-socketed sub-2 nm δ-Bi_2_O_3_ heterostructure with strong interfacial interaction.

**Fig. 2:**
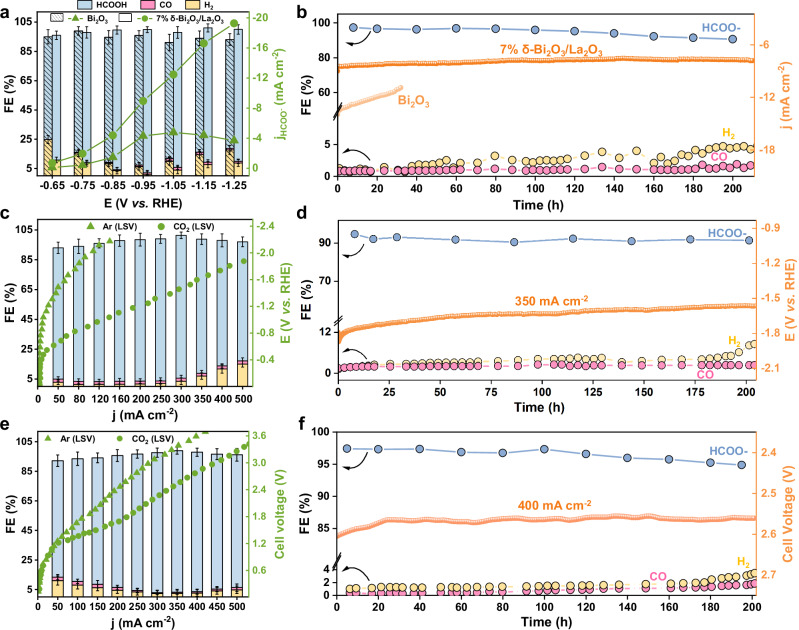
Electrocatalytic CO_2_RR performance. H-type cell: **a** FEs of formate and H_2_ and relevant *j*_formate_ on 7% δ-Bi_2_O_3_/La_2_O_3_ and pure Bi_2_O_3_ recorded under different potentials; **b** durability test of 7% δ-Bi_2_O_3_/La_2_O_3_ and pure Bi_2_O_3_ at a fixed potential of −0.95 V vs. RHE. CO_2_-saturated 0.5 M KHCO_3_ was used as the catholyte (pH = 7.22 ± 0.05). Flow-cell: **c** LSV curves of 7% δ-Bi_2_O_3_/La_2_O_3_ under CO_2_ and Ar atmospheres, and FEs of various products on 7% δ-Bi_2_O_3_/La_2_O_3_ at varying current density; **d** durability test of 7% δ-Bi_2_O_3_/La_2_O_3_ at a current density of 350 mA cm^−2^. CO_2_-saturated 0.5 M KHCO_3_ was used as the catholyte (pH = 7.22 ± 0.05). MEA-electrolyzer: **e** LSV curves of δ-Bi_2_O_3_/La_2_O_3_ under CO_2_ and Ar atmospheres, and FEs of various products on 7% δ-Bi_2_O_3_/La_2_O_3_ at different current densities; **f** durability test and corresponding product distributions of continuous 200 h electrolysis under a constant current density of 400 mA cm^−2^, with humified CO_2_ flowed through the cathode side and 1.0 M KHCO_3_ as the anolyte (pH = 8.34 ± 0.06). The error bars represent standard deviations from three independent measurements. Catalyst mass loading was fixed at 1 mg cm^−2^. Typical solution resistance in CO_2_-saturated 0.5 M KHCO_3_ was determined to be 5.3 ± 0.2 Ω for the H-type cell and 1.1 ± 0.4 Ω for the flow cell, respectively. All potentials are reported without iR compensation. Source data are provided as a Source data file.

The as-prepared 7% δ-Bi_2_O_3_/La_2_O_3_ heterogeneous catalysts are evaluated in a H-type cell using CO_2_-saturated 0.5 M KHCO_3_ aqueous electrolyte. All potentials were referenced to a reversible hydrogen electrode (RHE) without iR compensation. Linear sweep voltammetry (LSV) curves (Supplementary Fig. 26) demonstrate that the La_2_O_3_ substrate is inactive against CO_2_ reduction. With the decoration of sub-2 nm δ-Bi_2_O_3_, the current densities of δ-Bi_2_O_3_/La_2_O_3_ samples in CO_2_ are much higher than that in N_2_ atmosphere, showcasing their intrinsic activity for CO_2_ reduction. Among them, 7% δ-Bi_2_O_3_/La_2_O_3_ exhibits the largest current density and the most positive onset potential, signifying its optimal CO_2_RR activity (Supplementary Figs. 26a and 27). Subsequently, controlled-potential electrolysis is conducted over a potential interval from ‒0.65 to ‒1.25 V vs. RHE (Supplementary Fig. 26b–f). The gaseous and liquid products are quantitatively analyzed by gas chromatography (GC) and ^1^H nuclear magnetic resonance (^1^H NMR), with calibration against standard curves (Supplementary Fig. 28). As shown in Supplementary Fig. 29a, pure La_2_O_3_ mainly produces H_2_, suggesting its inert activity for CO_2_RR. For 7% δ-Bi_2_O_3_/La_2_O_3_ and pure Bi_2_O_3_, formate (HCOO^‒^) is the dominant product with minor CO and H_2_. Specifically, 7% δ-Bi_2_O_3_/La_2_O_3_ exhibits a FE_HCOO‒_ of ≥ 90% over a wide potential interval of ‒0.65 to ‒1.25 V vs. RHE with a maximum FE_HCOO‒_ of 97.8% at ‒0.95 V vs. RHE, which is higher than that of pure Bi_2_O_3_ powder with a FE_HCOO‒_ of 88.5% at ‒0.95 V vs. RHE (Fig. 2a). Noted, compared to 7% δ-Bi_2_O_3_/La_2_O_3_, both 1% and 3% δ-Bi_2_O_3_/La_2_O_3_ exhibit lower HCOO^‒^ selectivity, as the low density of δ-Bi_2_O_3_ sites is insufficient to effectively promote the reaction between *CO_2_^•−^ and *H, resulting in the enhanced competitive HER (Supplementary Fig. 29b–d). We further analyzed the formate partial current density (*j*_HCOO‒_) as shown in Fig. 2a, 7% δ-Bi_2_O_3_/La_2_O_3_ shows a steady rise in the *j*_HCOO‒_ as the potential shifts negatively, while the *j*_HCOO‒_ of pure Bi_2_O_3_ undergoes a significant decay from‒1.05 V vs. RHE. Longterm stability test of 7% δ-Bi_2_O_3_/La_2_O_3_ catalyst is further conducted at a fixed potential of‒0.95 V vs. RHE. As shown in Fig. 2b, the 7% δ-Bi_2_O_3_/La_2_O_3_ catalyst maintains a steady current density of ~7.8 mA cm^‒2^ and FE_HCOO‒_ of >90% for ~210 h, as further indicated by the steady production rate of HCOO^‒^ based on ^1^H NMR quantitative results (Supplementary Fig. 30). On the other hand, pure Bi_2_O_3_ sample shows relatively poor durability, and FEs of HCOO^‒^ distinctly drop from 88.5 to 55.6% only after electrolysis for 30 h at ‒0.95 V vs. RHE (Supplementary Fig. 31). This result indicates that the strong metal oxide–support interaction in the 7% δ-Bi_2_O_3_/La_2_O_3_ heterostructure is beneficial for enhancing electrochemical stability. Ultraviolet photoelectron spectroscopy (UPS) is further used to probe the interfacial electronic interactions by measuring the work function (WF) of the samples. As shown in Supplementary Fig. 32a, the measured WF values for La_2_O_3_, Bi_2_O_3_, and 7% δ-Bi_2_O_3_/La_2_O_3_ are 3.40, 2.60, and 1.74 eV, respectively. The reduced work function of 7% δ-Bi_2_O_3_/La_2_O_3_ facilitates electron transfer to adsorbed CO_2_, providing evidence for interfacial electronic modulation associated with the SMOSI effect^20^. The charge-transfer kinetic for HCOO^‒^ formation is also evaluated by the Tafel slope (Supplementary Fig. 32b). The 7% δ-Bi_2_O_3_/La_2_O_3_ heterostructure yields a Tafel slope of of 96.8 mV dec^−1^, much smaller than that of pure Bi_2_O_3_ (133.5 mV dec^−1^), implying faster electron transfer during the CO_2_RR^30^.

To assess the practical potential of 7% δ-Bi_2_O_3_/La_2_O_3_ catalyst at industrially relevant current densities, the electrocatalytic CO_2_RR performance is further evaluated in both the flow-cell and membrane electrode assembly (MEA) electrolyzers using KHCO_3_ as the electrolyte (Fig. 1a, b and Supplementary Figs. 33, 34)^31–33^. All potentials were referenced to a reversible hydrogen electrode (RHE) without iR compensation. Because the produced formate can diffuse from the cathode to the anode compartment through the anion exchange membrane (AEM), it was collected from both compartments. To ensure accurate quantification, the formate that migrated to the anolyte was quantified by both ^1^H NMR and high-performance liquid chromatography (HPLC, Supplementary Fig. 35). In a flow-cell, the LSV curves under a CO_2_ atmosphere (Fig. 2c) reveal that 7% δ-Bi_2_O_3_/La_2_O_3_ achieves a current density of 500 mA cm^−2^ at −1.85 V vs. RHE, higher than the 160 mA cm^−2^ achieved in an Ar atmosphere at −2.2 V vs. RHE, demonstrating its enhanced intrinsic activity and favorable kinetics for CO_2_ reduction. The faradaic efficiencies for the various CO_2_RR products in the current range of 50–500 mA cm^−2^ are shown in Fig. 2c, and the galvanostatic curves are presented in Supplementary Fig. 36. A high formate faradaic efficiency (>90%) is maintained over a wide current density range of 50–350 mA cm^−2^. Supplementary Figs. 37a and 38a show the quantitative analysis results of formate in the anolyte by ¹H NMR and HPLC, respectively. The corresponding crossover ratios (i.e., FE_anolyte_/ [FE_anolyte_ + FE_catholyte_]) as a function of current density are listed in Supplementary Table 2^34^. Subsequently, we conducted a stability test of 7% δ-Bi_2_O_3_/La_2_O_3_ in a flow-cell at 350 mA cm^−2^. The formate faradaic efficiency remains above 90% during 205 h of continuous electrolysis (Fig. 2d), with the overall CO and H_2_ faradaic efficiencies below 6%. Structural characterizations reveal that, after electrochemical stability testing, δ-Bi_2_O_3_ sub-nanoparticles remain uniformly dispersed on La_2_O_3_ porous microspheres, with well-defined heterointerfaces and no apparent structural degradation (Supplementary Figs. 39 and 40). We also evaluated the CO_2_RR performance of 1, 3, 10, and 15% δ-Bi_2_O_3_/La_2_O_3_ catalysts in a flow-cell under identical conditions. Compared to the 7% δ-Bi_2_O_3_/La_2_O_3_, the 1% and 3% δ-Bi_2_O_3_/La_2_O_3_ catalysts show inferior CO_2_RR performance due to the low density of sub-2 nm δ-Bi_2_O_3_ active sites (Supplementary Fig. 41), while the 10% and 15% δ-Bi_2_O_3_/La_2_O_3_ catalysts exhibit lower formate faradaic efficiency and poorer operational stability due to the aggregation of δ-Bi_2_O_3_ sub-nanoparticles (Supplementary Fig. 42). Specifically, the 10% δ-Bi_2_O_3_/La_2_O_3_ catalyst delivers a formate faradaic efficiency of ~91.5% and maintains stable performance for ~175 h, whereas the 15% sample exhibits a lower efficiency (~89.3%) and deactivates after ~154 h. These results suggest that a 7% Bi loading may represent an optimal balance between active site density and dispersion. To verify the distinctive advantage of sub-nanoparticles dispersion to markedly increase the SMOSI effect, we also synthesized the La_2_O_3_-socketed 20 nm δ-Bi_2_O_3_ catalyst (Supplementary Fig. 43a–c). CO_2_RR performance in the flow-cell electrolyzer show that this catalyst remains stable for only ~105 h, delivering a formate faradaic efficiency of ~84.7% at 350 mA cm^−2^ (Supplementary Fig. 43d, e). Furthermore, we employed the MEA-based system feed with humidified CO_2_ to evaluate the practical CO_2_RR performance of the 7% δ-Bi_2_O_3_/La_2_O_3_ catalyst. The galvanostatic curves in the current range of 50–500 mA cm^−2^ are shown in Supplementary Fig. 44. The analysis results of formate in the anolyte by ¹H NMR and HPLC are displayed in Supplementary Figs. 37b and 38b, respectively. The corresponding crossover ratios as a function of current density are listed in Supplementary Table 3. The MEA-based electrolyzer achieves a formate faradaic efficiency of ~93.2% and a current density of 500 mA cm^−2^ at a cell voltage of ~2.95 V, with the H_2_ faradaic efficiency remaining below 4% (Fig. 2e). Notably, to prevent severe accumulation and anodic oxidation of formate at the anode, the anolyte was circulated at a flow rate of 30 mL min^−1^ and refreshed every 45 min. Therefore, the faradaic efficiency loss due to anodic oxidation of formate is less than 5% in both flow cell and MEA electrolyzers^34^. The full-cell energy efficiency (EE) for formate production in the MEA electrolyzer reaches ~52.6% at 400 mA cm^−2^, a value comparable to most reported Bi-based CO_2_RR catalysts under similar conditions (Supplementary Fig. 45 and Supplementary Table 4). Last but not least, by maintaining an industrially relevant current density of 400 mA cm^−2^ in an MEA cell for 200 h of continuous electrolysis, the formate selectivity remains above 95%, together with a stable full cell voltage of about 2.56 V (Fig. 2f). The performance of 7% δ-Bi_2_O_3_/La_2_O_3_ for CO_2_-to-formate conversion is competitive with that of recently reported Bi_2_O_3_-based catalysts in both H-type and industrial-level electrolyzers (Supplementary Table 4), indicating its potential for practical application. Taken together, these results demonstrated that the strong SMOSI effect arising from interfacial Bi–O–La bonds in a La_2_O_3_-socketed sub-2 nm δ-Bi_2_O_3_ catalyst underpins its enhanced stability (>200 h) for CO_2_-to-formate conversion.

**Fig. 3:**
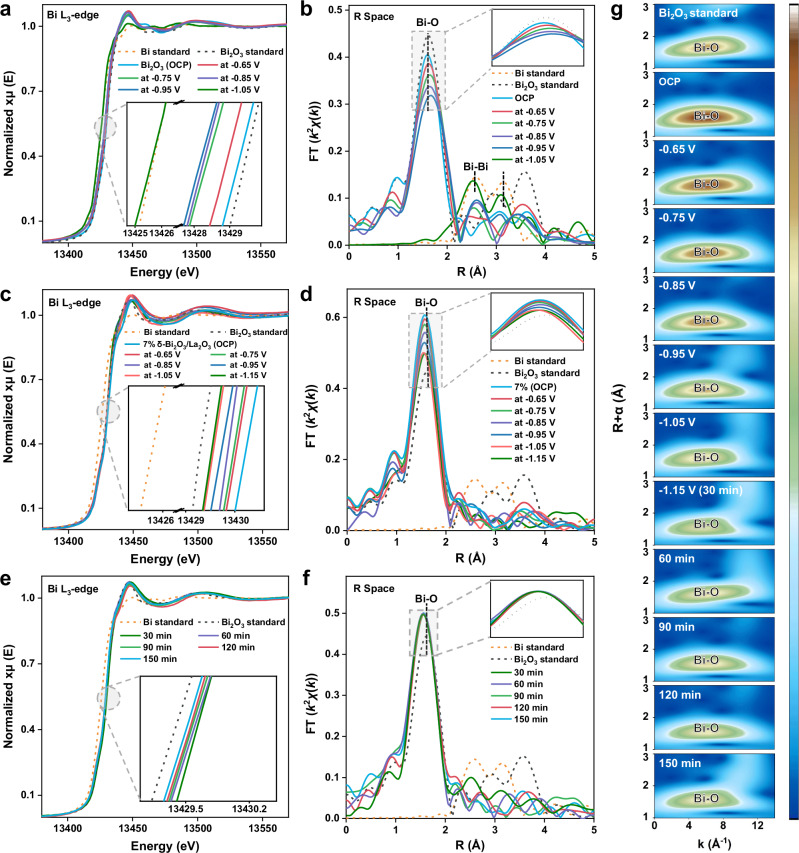
In situ XAS characterization. **a**, **c** Bi L_3_-edge XANES spectra and **b**, **d** corresponding *k*^2^-weighted FT-EXAFS spectra of pure Bi_2_O_3_ and 7% δ-Bi_2_O_3_/La_2_O_3_ under various applied potentials (vs. RHE) during CO_2_ electroreduction. **e**, **f** Time-evolved Bi L_3_-edge XANES spectra and *k*^2^-weighted FT-EXAFS spectra of 7% δ-Bi_2_O_3_/La_2_O_3_ at −1.15 V vs. RHE. The insets in (**a**–**f**) show zoomed-in views of selected spectral regions. **g** WT-EXAFS map of 7% δ-Bi_2_O_3_/La_2_O_3_ during CO_2_ electroreduction. CO_2_-saturated 0.5 M KHCO_3_ was used as the catholyte (pH = 7.22 ± 0.05). Catalyst mass loading was fixed at 1 mg cm^−2^. Typical solution resistance in CO_2_-saturated 0.5 M KHCO_3_ was determined to be 2.3 ± 0.2 Ω for the XAS electrolysis cell. All potentials are reported without iR compensation. Source data are provided as a Source data file.

Given that metal oxides inevitably undergo spontaneous reduction under realistic CO_2_RR conditions, a series of in situ and quasi*-*in situ spectroscopy characterizations were conducted. La_2_O_3_ is generally considered an irreducible oxide due to its highly negative reduction potential (La^3+^/La^0^, −2.90 V vs. RHE)^35,36^. We therefore performed in situ Bi L_3_-edge X-ray absorption spectroscopy (XAS, Supplementary Fig. 46) to monitor and compare the evolution of the active Bi–O layer in the δ-Bi_2_O_3_/La_2_O_3_ heterostructure with that in pure Bi_2_O_3_. To ensure the catalyst reaches a stable electrochemical state, the electrode potential is stepped from the open-circuit potential (OCP) to −1.15 V vs. RHE and held at this potential for at least 30 min during the measurement. For pure Bi_2_O_3_, as the potential is scanned from the open-circuit potential (OCP) to −1.05 V vs. RHE, the Bi L_3_-edge absorption edge progressively shifts to lower energy and toward the Bi foil reference (Fig. 3a), signifying a continuous decrease in the Bi oxidation state. The Fourier transform (FT) *k*^2^-weighted extended X-ray absorption fine structure (EXAFS) spectra reveal that the dominant Bi–O scattering peak in R-space shifts from ~1.60 Å at OCP to ~1.67 Å at −0.95 V vs. RHE (Fig. 3b), accompanied by a decrease in the Bi–O coordination number from ~3.2 to ~2.1 (Supplementary Table 5 and Supplementary Fig. 47). Additionally, new peaks corresponding to Bi–Bi scattering emerge at ~2.56 and ~3.11 Å in the FT-EXAFS spectrum at −1.05 V vs. RHE, suggesting the formation of metallic Bi. This is further confirmed by the appearance of characteristic Bi–Bi features in the wavelet transform (WT) spectra (Supplementary Fig. 48). In contrast, the Bi L_3_-edge absorption energy of 7% δ-Bi_2_O_3_/La_2_O_3_ decreases slightly with applied potentials (Fig. 3c), reflecting a relatively stable Bi valence state under operating conditions. Moreover, the average Bi L_3_-edge energy of 7% δ-Bi_2_O_3_/La_2_O_3_ is approximately 0.9 eV higher than that of the Bi_2_O_3_ reference (Fig. 3c), indicative of enhanced electron deficiency at Bi sites and supporting the presence of Bi_2_O_3 + x_ moiety with a highly oxidized local structure. This effect arises from the formed interfacial Bi–O–La bonding, which polarizes the Bi–O bonds and redistributes electron density. Consequently, the Bi–O coordination environment in δ-Bi_2_O_3_ is perturbed by adjacent La atoms at the heterointerface, leading to reduced electron density around Bi^37,38^. This electron-deficient state is consistent with the positive binding-energy shift observed in the Bi 4*f* XPS spectra (Supplementary Fig. 15b). These experimental observations are further rationalized by DFT calculations presented in the following section, which quantify the interfacial charge transfer and orbital hybridization. Additionally, the EXAFS fitting results (Supplementary Table 6 and Supplementary Fig. 49) reveal a shorter average Bi–O bond length in 7% δ-Bi_2_O_3_/La_2_O_3_ (~2.10 Å) compared to pure Bi_2_O_3_ (~2.15 Å), indicating that the interphase interaction between La_2_O_3_ and δ-Bi_2_O_3_ effectively contracts the Bi−O bonds. This bond contraction suggests an enhanced local structural rigidity, which contributes to improve the stability under operational CO_2_RR conditions. As shown in Fig. 3d, when the potential is swept from OCP to −1.15 V vs. RHE, the Bi–O coordination number decreases slightly from ~4.9 to ~4.3 and remains stable. Concurrently, the peak position of the first Bi–O coordination shell in R-space exhibits negligible shift, and no Bi–Bi scattering features are detected in the EXAFS spectra. Furthermore, an extended electrolysis experiment is performed at −1.15 V vs. RHE for 150 min to assess the robustness of the Bi–O bonds in the δ-Bi_2_O_3_/La_2_O_3_. Throughout this process, the Bi L_3_-edge XANES spectra exhibit only a minor energy shift (Fig. 3e), and the Bi–O coordination number undergoes a slight change from ~4.3 to ~4.1 (Fig. 3f, Supplementary Fig. 50, and Supplementary Table 7). The wavelet transform analysis of the EXAFS data (Fig. 3g) further provides a clear visualization of the coordination state of Bi in 7% δ-Bi_2_O_3_/La_2_O_3_ as a function of applied potential and electrolysis time. Finally, linear combination fitting (LCF) of the Bi L_3_-edge XANES spectra for the 7% δ-Bi_2_O_3_/La_2_O_3_ (Supplementary Fig. 51) quantifies the metallic Bi^0^ content as less than 5% (~4.7%) during the 150-min CO_2_RR at −1.15 V vs. RHE^10^, indicating that Bi remains predominantly in its oxidation state under these cathodic conditions. Overall, the enhanced interphase interaction between La_2_O_3_ and δ-Bi_2_O_3_ in the heterojunction catalyst results in the shorter Bi–O bond length, indicative of a more robust local coordination environment. This structural reinforcement helps suppress the quick reduction of the active oxidized Bi species during prolonged CO_2_RR electrolysis.

**Fig. 4:**
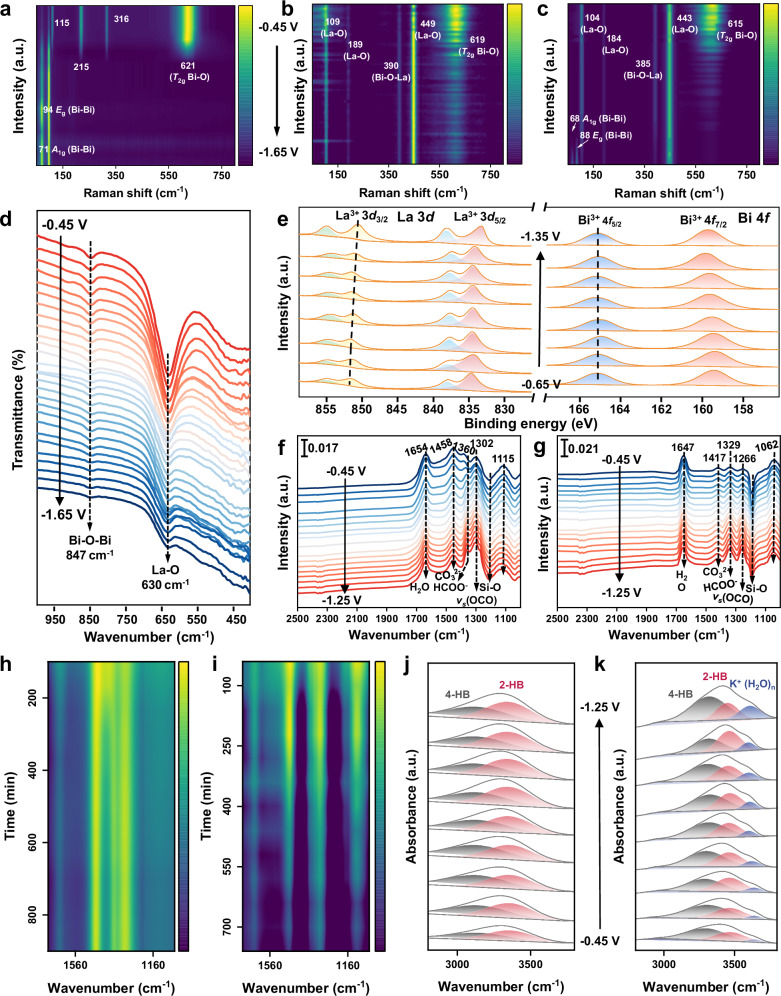
Structural evolution and intermediates detection during CO_2_ electroreduction. In situ Raman spectra of **a** pure Bi_2_O_3_, **b** 7% δ-Bi_2_O_3_/La_2_O_3_, and **c** 15% δ-Bi_2_O_3_/La_2_O_3_. **d** In situ FT-IR spectra of 7% δ-Bi_2_O_3_/La_2_O_3_. All spectra were collected at applied potentials from −0.45 to −1.65 V vs. RHE. **e** Quasi-in situ XPS spectra of 7% δ-Bi_2_O_3_/La_2_O_3_ collected after electrolysis at potentials ranging from −0.65 to −1.35 V vs. RHE. In situ ATR-SEIRA spectra of 7% δ-Bi_2_O_3_/La_2_O_3_ recorded at different applied potentials in (**f**) ^12^CO_2_-saturated and **g** isotope labeling ^13^CO_2_-saturated 0.5 M KHCO_3_ solution, using a reference spectrum at −0.25 V vs. RHE. Time-evolved ATR-SEIRAS color contour map for **h** 7% δ-Bi_2_O_3_/La_2_O_3_ and **i** pure Bi_2_O_3_ at −0.95 V vs. RHE. Gaussian fits of three O−H stretching modes for **j** pure Bi_2_O_3_ and **k** 7% δ-Bi_2_O_3_/La_2_O_3_ based on the ATR-SEIRA spectra. CO_2_-saturated 0.5 M KHCO_3_ was used as the catholyte (pH = 7.22 ± 0.05). Typical solution resistance in CO_2_-saturated 0.5 M KHCO_3_ was determined to be 4.1 ± 1.2 Ω for the ATR-SEIRA cell and 1.6 ± 0.3 Ω for the Raman cell, respectively. All potentials are reported without iR compensation. Source data are provided as a Source data file.

Subsequently, in situ Raman and FT-IR characterizations were also carried out to probe the potential-dependent structural evolution of these electrocatalysts. We performed in situ Raman measurements based on a spectro-electrochemical flow cell (Supplementary Fig. 52). As shown in Fig. 4a, the characteristic peaks of pure Bi_2_O_3_ at 115, 215, 316, and 621 cm^−1^ gradually decreased in intensity and vanished completely as the applied cathodic potential was swept from −0.45 to −1.65 V vs. RHE^17,18^. Meanwhile, new characteristic peaks at ~71 and ~94 cm^−1^, assigned to the *E*_g_ and *A*_1g_ vibrational modes of metallic Bi, were initially detected at −0.65 V vs. RHE^39^, which suggests the severe disruption of Bi−O bonds in pure Bi_2_O_3_ under reductive conditions. For the 7% δ-Bi_2_O_3_/La_2_O_3_ catalyst, as shown in Fig. 4b, the vibration peaks associated with La−O (109, 189, and 449 cm^−1^), Bi−O (619 cm^−1^) and Bi−O−La (390 cm^−1^) exhibit negligible attenuation as the cathodic potential decreases, with no Bi−Bi signals observed^21,40^. Notably, in the high-loading (10 and 15%) samples, the Bi–O and Bi–O–La signals could not be maintained over such a wide potential window. As shown in Fig. 4c and Supplementary Fig. 53, the characteristic peaks of metallic Bi−Bi at ~70 and ~95 cm⁻¹ appeared at −1.45 V vs. RHE for the 10% δ-Bi_2_O_3_/La_2_O_3_ sample and at −1.25 V vs. RHE for the 15% δ-Bi_2_O_3_/La_2_O_3_ sample, respectively. The Raman results again highlight the distinct advantage of well-dispersed sub-2 nm δ-Bi_2_O_3_ particles in the 7% δ-Bi_2_O_3_/La_2_O_3_ catalyst in enhancing the SMOSI effect. Time-dependent Raman spectra (Supplementary Fig. 54) further show that the Bi−O and Bi−O−La signals of 7% δ-Bi_2_O_3_/La_2_O_3_ remained detectable throughout a 740-min electrolysis period at −0.95 V vs. RHE. Additionally, we employed in situ FT-IR spectroscopy (Supplementary Fig. 55) on a reflecting plane of ZnSe prism to probe the structural evolution of δ-Bi_2_O_3_/La_2_O_3_ catalyst during the CO_2_RR process. As displayed in Fig. 4d, the characteristic band near 847 cm^−1^ is assigned to the Bi−O bond, and the absorption band at ~630 cm^−1^ is attributed to the stretching vibration of the La−O bond^41,42^. Potentiodynamic FT-IR spectra show that the Bi–O and La–O signals persist throughout the cathodic potential sweep from −0.45 to −1.65 V vs. RHE. Notably, a slight attenuation of the La–O vibrational signals was observed as the cathodic potential decreases. Time-dependent FT-IR analysis at a fixed potential of −0.95 V vs. RHE also shows a slight weakening trend of La–O bonds with time (Supplementary Fig. 56). This result suggests that the strong electronic interaction contributes to the transfer of excessive electrons from δ-Bi_2_O_3_ to La_2_O_3_ during long-term CO_2_RR electrolysis, thus protecting the Bi−O bonds from attack by the accumulated electrons^20^. The XPS peak shift previously observed in Supplementary Fig. 15 also confirms this interfacial electron transfer. Furthermore, the quasi-in situ XPS measurements shown in Fig. 4e and Supplementary Fig. 57a reveal that the La^3+^ 3*d* peak gradually shifts to lower binding energies as the cathodic potential decreases, indicating that La_2_O_3_ functions as an electron acceptor at the interface^43^. It is noteworthy that the lattice oxygen signals associated with the Bi−O−La bonds remain visible throughout the electrolysis process from −0.65 to −1.35 V vs. RHE (Supplementary Fig. 57b), which strongly verifies a robust electronic interaction between δ-Bi_2_O_3_ and La_2_O_3_ phase. To further confirm the strong oxide–support interaction on the δ-Bi_2_O_3_/La_2_O_3_ heterojunction, we conducted H_2_ temperature programmed reduction (H_2_-TPR) measurements on 7% δ-Bi_2_O_3_/La_2_O_3_ and a physically mixed Bi_2_O_3_-La_2_O_3_ sample. The H_2_-TPR profiles (Supplementary Fig. 58) demonstrated that the high-temperature peak at 616.5 °C is attributed to the reduction of La_2_O_3_ species^44^, while a broad medium-temperature feature (350–450°C) corresponds to the reduction of Bi_2_O_3_ species^45^. An obvious shift of the Bi_2_O_3_ reduction peak to a higher temperature region (414.5 and 454.6 °C) is observed on the 7% δ-Bi_2_O_3_/La_2_O_3_, indicating that the Bi_2_O_3_ species in this heterostructure are more difficult to be reduced.

We further conducted attenuated total reflection surface-enhanced infrared absorption spectroscopy (ATR-SEIRAS, Supplementary Fig. 55) using an H-cell under CO_2_RR conditions to identify the adsorbed intermediates and elucidate the reaction mechanism. With the spectrum at −0.25 V vs. RHE as the reference, the potential-dependent SEIRA spectra were acquired for pure Bi_2_O_3_ and δ-Bi_2_O_3_/La_2_O_3_ electrodes in CO_2_-saturated 0.5 M KHCO_3_ from −0.45 to −1.25 V vs. RHE (Fig. 4f and Supplementary Fig. 59). The characteristic peaks located at ~1302 and ~1360 cm^−1^ are attributed to the *ν*_s_(OCO) stretching vibrations of bidentate *HCOO and dissolved HCOO^−^, respectively^46^. The presence of the *HCOO intermediate indicates that CO_2_ adsorbs on the catalyst surface in a bidentate oxygen-bound configuration and undergoes carbon protonation (C–H bond formation) rather than oxygen protonation to form *COOH, since no obvious *CO signal was detected. To confirm that the observed IR peaks originate from CO_2_ reduction, we carried out control experiments using a bare Au-coated Si-based internal reflection element (IRE) (Supplementary Fig. 60a) and pure La_2_O_3_ sample on the Au-coated IRE (Supplementary Fig. 60b) under identical CO_2_RR conditions. No characteristic peaks for formate-related species at ~1310 and ~1360 cm^−1^ were observed in the ATR-SEIRA spectra. Moreover, we performed a ^13^C isotope labeling experiment (^13^CO_2_, 99.0 atom%, Sigma-Aldrich; Supplementary Fig. 61). Using a ^13^CO_2_-saturated 0.5 M KHCO_3_ electrolyte, the spectra for the 7% δ-Bi_2_O_3_/La_2_O_3_ electrode show obvious red shifts (Fig. 4g) for the IR bands at 1266 cm^−1^ (*ν*_s_(OCO)) and 1329 cm^−1^ (HCOO^−^). The red shifts in vibrational frequency align with the theoretical ^13^C/^12^C isotopic effect, further confirming the assignment of these IR bands^47,48^. We further analyzed the potential-dependent evolution of these formate-related IR bands. For the 7% δ-Bi_2_O_3_/La_2_O_3_ catalyst (Fig. 4f), an enhancement in the peak intensity at ~1302 and ~1360 cm^−1^ can be observed with decreasing cathodic potential, and eventually attains a steady state. For pure Bi_2_O_3_ (Supplementary Fig. 59), the peak intensities at ~1304 and ~1359 cm^−1^ are gradually weakening as the potential shift negatively, consistent with the reduction of Bi^3+^ to metallic Bi observed in the Raman spectra. Notably, the signal intensity of the *v*_s_(OCO) band on the 10% and 15% δ-Bi_2_O_3_/La_2_O_3_ samples shows a pronounced decreasing trend at more negative potentials (Supplementary Figs. 62 and 63), implying that the agglomeration of sub-nanoparticles induced by high Bi loading reduces the catalytic stability. Given the key role of *HCOO intermediate toward CO_2_-to-formate conversion, the time-evolved ATR-SEIRAS were also conducted to obtain more details during the long-term electrolysis process. At a fixed potential of −0.95 V vs. RHE, the ATR-SEIRAS contour map shows that the *HCOO intermediates can be stably retained on 7% δ-Bi_2_O_3_/La_2_O_3_ electrode throughout the 900-minute CO_2_RR process (Fig. 4g and Supplementary Fig. 64a). In contrast, the *HCOO intermediates formed on pure Bi_2_O_3_ electrode undergo a rapid decay during the reduction process of 720 min (Fig. 4h and Supplementary Fig. 64b) due to the reduction of Bi^3+^ sites. Taken together, these results clearly demonstrate that the Bi sites in 7% δ-Bi_2_O_3_/La_2_O_3_ remain in an oxidized state even under very negative potentials, which sustains the adsorption of the *HCOO intermediate and enables highly efficient and stable CO_2_ reduction to formate.

Besides, the characteristic peaks centered at ~1115 and ~1458 cm^−1^ (Fig. 4f and Supplementary Fig. 59 are also observed, which are attributed to the vibrational bands of adsorbed HCO_3_^−^ and CO_3_^2−^, respectively^49,50^. As the cathodic potential shifts negatively, the intensity of adsorbed HCO_3_^−^ in pure Bi_2_O_3_ gradually decreases, accompanied by the increased CO_3_^2−^ signal. However, the CO_3_^2−^ signal in 7% δ-Bi_2_O_3_/La_2_O_3_ is very weak, and the peak intensity of adsorbed HCO_3_^−^ remains almost unchanged. A more pronounced differences in the variation of these peak signals can be seen in the enlarged spectra of Supplementary Fig. 65. To elucidate the role of HCO_3_^−^ in the CO_2_-to-formate conversion pathway, the effect of HCO_3_^−^ concentration (ranging from 0.1 to 0.5 mol/L) on the CO_2_RR performance was systematically investigated^51^. As shown in Supplementary Fig. 66, the *j*_formate_ (log(*j*_formate_)) plotted against the HCO_3_^−^ concentration (log([HCO_3_^−^])) was calculated at a fixed potential of −0.95 V vs. RHE. For pure Bi_2_O_3_, the plot (slope = 0.87) shows a quasi-one-order dependence of HCO_3_^−^ concentration, indicating that protons (H^+^) are sourced from HCO_3_^−^ rather than from H_2_O in the formation of *HCOO intermediates^39^. In contrast, the reaction order of 7% δ-Bi_2_O_3_/La_2_O_3_ displays a slope of 0.14, an approximate zero-order dependence of HCO_3_^−^ concentration, which suggests that H_2_O rather than HCO_3_^−^ may be directly involved in the formate pathway^52^. We further studied the differences in H_2_O adsorption and activation over pure Bi_2_O_3_ and 7% δ-Bi_2_O_3_/La_2_O_3_ electrodes based on the SEIRA spectra. As shown in Fig. 4i, j, and Supplementary Fig. 67, the *ν*(O−H) stretching band of H_2_O in the range of 3000 to 3800 cm^−1^ can be deconvoluted into three gaussian peaks, corresponding to: (i) 4-coordinated hydrogen-bonded water (4-HB·H_2_O, low wavenumber component of ~3250 cm^−1^, gray); (ii) 2-coordinated hydrogen-bonded water (2-HB·H_2_O, primary component of ~3400 cm^−1^, red); and (iii) K^+^-hydrated water with weak hydrogen-bond interaction (K^+^(H_2_O)_n_, high wavenumber component of ~3580 cm^−1^, blue), respectively^53–55^. The K^+^(H_2_O)_n_ signal is primarily present on the δ-Bi_2_O_3_/La_2_O_3_ electrodes, and the K^+^(H_2_O)_n_ population increases as the potential shifts negatively, which indicates that the introduction of La_2_O_3_ support as well as the induced SMOSI effect promotes interfacial water enrichment and facilitates H_2_O adsorption and activation at the heterojunction catalyst.

**Fig. 5:**
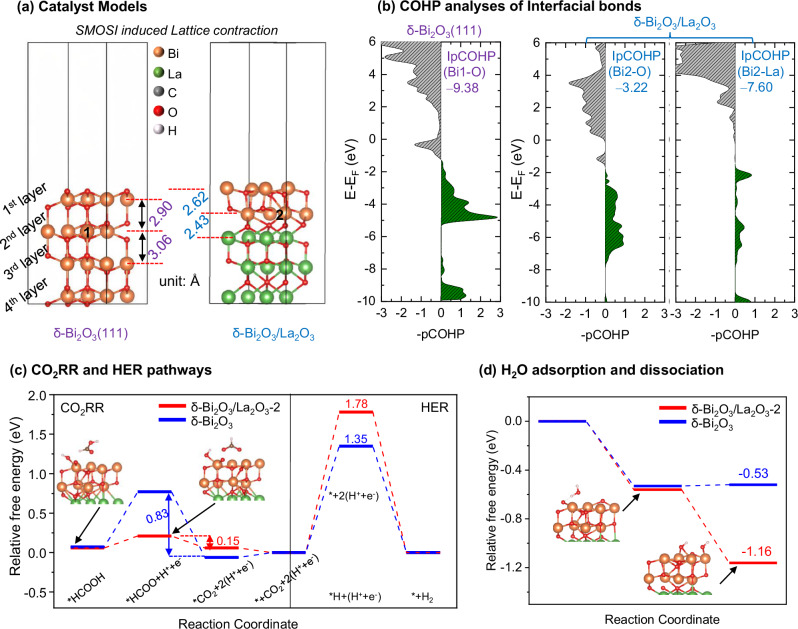
Theoretical calculations. **a** DFT-optimized structural models for the δ-Bi_2_O_3_(111) surface and the δ-Bi_2_O_3_/La_2_O_3_ heterojunction. **b** pCOHPs for Bi_1_-O in δ-Bi_2_O_3_, Bi_2_-O and Bi_2_-La in δ-Bi_2_O_3_/La_2_O_3_. **c** Comparison of the CO_2_RR and HER reaction pathways on the pristine δ-Bi_2_O_3_ and δ-Bi_2_O_3_/La_2_O_3_-2 models. The insets show the optimized structures of the *HCOO and *HCOOH intermediates on the δ-Bi_2_O_3_/La_2_O_3_-2 model. **d** Comparison of H_2_O dissociation pathways on the pristine δ-Bi_2_O_3_ and δ-Bi_2_O_3_/La_2_O_3_-2 models. The insets show the optimized structures of *H_2_O and *OH + *H intermediates on the δ-Bi_2_O_3_/La_2_O_3_. La: green; Bi: yellow; C: brown; O: red; H: pink. Source data are provided as a Source data file.

We further conducted the density functional theory (DFT) calculations to understand the stabilizing effect of La_2_O_3_ support on δ-Bi_2_O_3_ by constructing pristine δ-Bi_2_O_3_(111) and δ-Bi_2_O_3_(111)/La_2_O_3_(001) heterojunction (theoretical details are given in “Methods”). As shown in Fig. 5a, the δ-Bi_2_O_3_(111) model consists of four δ-Bi_2_O_3_ layers, each featuring a 3O-4Bi-3O moiety. The δ-Bi_2_O_3_/La_2_O_3_ heterojunction comprises two δ-Bi_2_O_3_ layers built upon three La_2_O_3_ layers, of which the two counterparts are cut from the pristine δ-Bi_2_O_3_(111) and La_2_O_3_(001), respectively. As shown in Supplementary Fig. 68a, there are three O layers (each with 3 oxygen atoms) in the δ-Bi_2_O_3_ phase, with Bi atoms at the bottom and O atoms at the top. For the La_2_O_3_ support, there are four O atoms at the surface. The initial structure was then optimized using the DFT method, and the final heterojunction structure was shown in Fig. 5a and Supplementary Fig. 68b. Compared to the initial structure, the structural relaxation due to the strong interfacial interactions leads to significant atomic rearrangements, particularly among the oxygen atoms in the top two Bi_2_O_3_ layers. Specifically, one oxygen atom in the second O layer migrates into the third O layer, and simultaneously, three oxygen atoms in the first layer migrate into the second layer. The resulting top layer features a 4Bi-5O moiety, while the second layer possesses a 5O-4Bi-4O structure. This migration of oxygen atoms from the surface to the subsurface induces a notable lattice contraction in the Bi_2_O_3_ layers. Figure 5a shows that the interlayer distances of *d*_12_ (between the 1st and 2nd Bi layers) and *d*_23_ (between the 2nd Bi layer and 3rd La layers) in the δ-Bi_2_O_3_/La_2_O_3_ are 2.62 and 2.43 Å, respectively, which are significantly smaller than the corresponding distances of *d*_12_ = 2.90 Å (between the 1st and 2nd Bi layers) and *d*_23_ = 3.06 Å (between the 2nd and 3rd Bi layers) in pristine δ-Bi_2_O_3_(111). This further supports an enhanced interfacial compressive stress between δ-Bi_2_O_3_ and La_2_O_3_ phases, ascribing to the SMOSI effect. Notably, our previous XAS measurements and simulations in Supplementary Figs. 20–24 have demonstrated that the large-scale interdiffusion of Bi and La atoms is unlikely to occur spontaneously. However, it should be noted that the realistic heterojunction structure is much more complicated, and the penetration of Bi and La atoms at the interface may be possible. We thus constructed a δ-Bi_2_O_3_/La_2_O_3_ heterostructure with an intermixed interface, in which one Bi atom substitutes into the La_2_O_3_ lattice and one La atom incorporates into the δ-Bi_2_O_3_ lattice (Supplementary Fig. 69). By comparing the system energies of different interface configurations, we find that the model after penetration is 0.25 eV higher in energy than the non-intermixed δ-Bi_2_O_3_/La_2_O_3_ interface, implying that the latter is thermodynamically more stable. Consequently, the δ-Bi_2_O_3_/La_2_O_3_ model in Fig. 5a reasonably represents the experimental heterojunction catalyst. The interlayer contraction of the Bi_2_O_3_ layer in the δ-Bi_2_O_3_/La_2_O_3_ heterojunction predicted by DFT is consistent with the reduced bond length (*R* value) in the Bi–O coordination shell from EXAFS fitting (Supplementary Tables 5 and 6). The formation of 5O-4Bi-4O layer near the interface shows an increased O:Bi ratio in the δ-Bi_2_O_3_/La_2_O_3_ (i.e., the existence of Bi_2_O_3 + x_ species), which is in agree with the higher absorption edge energy in the XANES spectra relative to that of reference Bi_2_O_3_ (Fig. 3c).

The driving force for oxygen migration and the resulting interlayer contraction in the δ-Bi_2_O_3_/La_2_O_3_ heterojunction can be elucidated by analyzing the electronic structure. Strong interfacial charge transfer was identified between the two phases. The charge density difference plot shows electron accumulation in the La_2_O_3_ phase and electron depletion in the δ-Bi_2_O_3_ phase (Supplementary Fig. 70), indicating interfacial charge transfer and redistribution between the two phases. In the δ-Bi_2_O_3_ region of the δ-Bi_2_O_3_/La_2_O_3_ heterostructure, the average Bader charge on Bi atoms increases from +1.66 |e| in the topmost (surface) layer to +2.03 |e| in the subsurface (interface) layer, indicating more electrons transfer to neighboring O and La atoms. This electrostatic gradient facilitates the migration of surface oxygen atoms toward the more positively charged Bi sites in the subsurface (interface) layer. Moreover, *p*–*d* hybridization between Bi and La atoms at the interface is evidenced by the projected density of state (PDOS). The analysis shows that the Bi 6*p* states shift downward in δ-Bi_2_O_3_/La_2_O_3_ compared to pristine δ-Bi_2_O_3_. The contraction of δ-Bi_2_O_3_ layers in the δ-Bi_2_O_3_/La_2_O_3_ heterostructure induces hybridization between the Bi 6*p* and La 5*d* orbitals, as evidenced by features at an energy level of approximately −5.0 eV in the PDOS plot (Supplementary Fig. 71). To further evaluate the bond strengths between the Bi atom in the second layer of δ-Bi_2_O_3_/La_2_O_3_ and the O/La atoms in the third interfacial layer, we calculated the integrated projected crystal orbital Hamilton population (IpCOHP) up to the Fermi level,^56,57^ as shown in Fig. 5b. The IpCOHP value for the Bi2 atom with three La atoms in the δ-Bi_2_O_3_/La_2_O_3_ structure is −7.60, confirming the formation of Bi–La bonds at the interface. Additionally, the IpCOHP value for the three Bi_2_-O bonds is −3.22, lower than the corresponding value of −9.38 (Bi_1_-O) in the pristine δ-Bi_2_O_3_ model. Thus, the total IpCOHP value of −10.82 for δ-Bi_2_O_3_/La_2_O_3_ is larger than the value of −9.38 for δ-Bi_2_O_3_, indicating that the Bi atom interacts with both O and La atom at the interfacial site, forming Bi–O–La and Bi–La bonds and stabilizing δ-Bi_2_O_3_ sub-nanoparticle on the La_2_O_3_ support. These findings are further supported by the calculated crystal orbital bond index (COBI)^58^, which quantifies covalent bond orders. The summation of integrated COBI (ICOBI) is 1.55 in δ-Bi_2_O_3_/La_2_O_3_ (0.89 and 0.66 for Bi_2_-La and Bi_2_-O bonds, respectively), higher than the value of 1.45 for Bi_1_-O in δ-Bi_2_O_3_, as shown in Supplementary Fig. 72. Overall, the condensation of oxygen atoms at the subsurface and interfacial sites leads to a contraction of δ-Bi_2_O_3_ layers, resulting in p–d hybridization in Bi–La bonds and the formation of Bi–O–La bonds, thereby stabilizing δ-Bi_2_O_3_ sub-nanoparticle. These analyses well support the SMOSI effect in the δ-Bi_2_O_3_/La_2_O_3_ Heterojunction. It should be noted that the DFT calculations are based on idealized models and may not fully capture the dynamic complexity of the catalyst under operating conditions.

We further evaluated the adsorption of oxygen molecule on the top of δ-Bi_2_O_3_/La_2_O_3_, giving rise to δ-Bi_2_O_3_/La_2_O_3_-1 model. The process is exothermic, with an adsorption energy of −2.96 eV (Supplementary Fig. 73), indicating that it is thermodynamically favorable for the heterojunction to be covered with oxygen. Given that surface oxygen atoms can spontaneously migrate to the subsurface of the heterojunction, this may lead to the easier formation of surface oxygen vacancies (O_v_). As shown in Supplementary Fig. 74, the calculated O_v_ formation energy for δ-Bi_2_O_3_/La_2_O_3_-1 is 1.12 eV, which is lower than the formation energy of 2.19 eV for δ-Bi_2_O_3_. The abundant oxygen vacancies in δ-Bi_2_O_3_/La_2_O_3_ are also confirmed by the obvious electron-paramagnetic resonance (EPR) signals, as shown in Supplementary Fig. 75. Moreover, the EPR signal of δ-Bi_2_O_3_/La_2_O_3_ decreases once the surface is covered with oxygen. Taken together, the above results identify that the spontaneous migration of oxygen atoms from the surface to the subsurface and lattice contraction between the δ-Bi_2_O_3_ and La_2_O_3_ phases induce the strong oxide–support interaction. Before the oxygen-molecule adsorbed δ-Bi_2_O_3_/La_2_O_3_-1 model is used in CO_2_RR and HER studies, its stability under realistic operating conditions must be evaluated. To this end, surface Pourbaix diagrams with respect to electrode potential versus standard hydrogen electrode (*U*_SHE_) and pH are plotted for both the δ-Bi_2_O_3_/La_2_O_3_-1 and δ-Bi_2_O_3_ models. Considering that in an aqueous electrochemical environment, surface-adsorbed oxygen can be protonated, leading to surface hydroxylation (i.e., forming a termination state covered with hydroxyl (–OH) groups). To more accurately reflect the realistic surface structure, we also included hydroxylated surface configurations in our computational models. As shown in Supplementary Fig. 76, the monohydroxylated surface (denoted as δ-Bi_2_O_3_/La_2_O_3_-2) is thermodynamically more stable than both the oxygen-adsorbed surface (i.e., δ-Bi_2_O_3_/La_2_O_3_-1 model in the original manuscript) and dihydroxylated surfaces with two –OH groups (denoted as δ-Bi_2_O_3_/La_2_O_3_-3) under experimentally relevant operating conditions. Specifically, in CO_2_-saturated 0.5 M KHCO_3_ solution (pH ≈ 7.2), within the potential window optimal for formate production (*U*_RHE_ = − 0.75 V to −1.05 V, corresponding to *U*_SHE_ = − 1.17 V to −1.47 V), the δ-Bi_2_O_3_/La_2_O_3_-2 model exhibits the lowest free energy among all considered terminations, making it the thermodynamically preferred structure for catalytic simulations under CO_2_RR conditions. In contrast, for the δ-Bi_2_O_3_ model, the Pourbaix diagram shows that the pristine surface is more stable than the hydroxylated models in the potential window of *U*_RHE_ = − 0.75 V to −1.05 V (*U*_SHE_ = − 1.17 V to −1.47 V), and thus the pristine δ-Bi_2_O_3_ model is a reasonable model for catalytic simulations under CO_2_RR conditions. In fact, the aforementioned SEIRA spectra in Fig. 4i, j have shown that, compared to pure Bi_2_O_3_, the δ-Bi_2_O_3_/La_2_O_3_ heterojunction surface is more readily protonated, facilitating the formation of surface-bound OH* species. We further studied the projected densities of states (PDOS) of surface oxygen atoms in the δ-Bi_2_O_3_ and δ-Bi_2_O_3_/La_2_O_3_ (Supplementary Fig. 77). The δ-Bi_2_O_3_/La_2_O_3_ exhibits an obvious upshift in the O 2*p*-band centers (*E*_p_) of −2.09 eV, compared to −2.58 eV in pristine δ-Bi_2_O_3_. This shift suggests that the strong oxide–support interaction reduces the occupancy of O-centered antibonding states, thereby enhancing the surface protonation propensity. Additionally, the adsorption and activation behavior of water molecules on both δ-Bi_2_O_3_/La_2_O_3_-1 and δ-Bi_2_O_3_ surfaces are compared. Theoretical results (Fig. 5d and Supplementary Fig. 78) show that the water dissociation is more favorable on δ-Bi_2_O_3_/La_2_O_3_-1 with a free energy of −1.16 eV, compared to −0.53 eV for δ-Bi_2_O_3_. This implies that the introduction of La_2_O_3_ can efficiently increase the *H coverage, which not only facilitates the surface protonation but also enhances the hydrogenation kinetics for formate production on δ-Bi_2_O_3_ sites. Therefore, δ-Bi_2_O_3_/La_2_O_3_-2 and pristine δ-Bi_2_O_3_ models are then used to investigate their CO_2_RR and HER activities. Complete reaction pathways are calculated and illustrated in Fig. 5c and Supplementary Figs. 79, 80. For pristine δ-Bi_2_O_3_, the formation of *HCOO from *CO_2_ intermediate, with an energy barrier of 0.83 eV, is identified as the rate-determining step (RDS). For δ-Bi_2_O_3_/La_2_O_3_-2, the RDS is also the protonation of *CO_2_ to form *HCOO, but with a reduced energy barrier of 0.15 eV, suggesting favorable kinetics for formate production on the heterojunction. Additionally, the energy barriers for the competitive HER are 1.35 and 1.78 eV for δ-Bi_2_O_3_ and δ-Bi_2_O_3_/La_2_O_3_-2, respectively, both of which are higher than the energy barriers of RDS for *HCOO formation in the CO_2_RR, indicating that the HER is less favorable on both catalysts. Moreover, the higher barrier of 1.78 eV indicates that the HER is kinetically more sluggish on the δ-Bi_2_O_3_/La_2_O_3_-2 heterojunction. Lastly, a schematic overview illustrating the stabilization of sub-2 nm δ-Bi_2_O_3_ nanoparticles during CO_2_RR-to-formate conversion via strong interfacial interactions with the La_2_O_3_ support is presented in Supplementary Fig. 81.

## Discussion

In summary, this work establishes a stabilization strategy for Bi_2_O_3_-based CO_2_RR catalyst through a La_2_O_3_-supported δ-Bi_2_O_3_ heterojunction featuring sub-2 nm domains and robust Bi–O–La interfacial linkages, achieving stable formate electrosynthesis for over 200 h. In situ XAS, Raman spectroscopy, STEM, and theoretical calculations reveal the spontaneous migration and condensation of oxygen atoms from the surface to interfacial regions, leading to contraction in δ-Bi_2_O_3_ domains and enhanced *d*–*p* orbital hybridization between interfacial La and Bi atoms. This structural relaxation stabilizes interfacial Bi–O–La linkages and an electron-deficient Bi_2_O_3 + x_ moiety, thereby effectively suppressing the reduction of Bi_2_O_3 + x_ to metallic Bi under CO_2_RR conditions. The Pourbaix diagram and in situ infrared spectroscopy further demonstrate that the La_2_O_3_ support stabilizes a hydroxylated δ-Bi_2_O_3_ surface by promoting surface protonation and lowers the barriers of RDS for *HCOO formation. These atomic-scale insights into oxygen-mediated interfacial reinforcement provide mechanistic guidance for developing robust electrocatalysts in sustainable energy conversion systems.

## Methods

### Chemicals and materials

Acrylamide (C_3_H_5_NO, 99.99%) and bismuth nitrate pentahydrate (Bi(NO_3_)_3_·5H_2_O, 99.99%) were purchased from Shanghai Aladdin Biochemical Technology Co., Ltd, China. Lanthanum nitrate hexahydrate (La(NO_3_)_3_·6H_2_O, 99.99%) was purchased from Shanghai Boer Chemical Reagents Co., Ltd, China. Glucose monohydrate (C_6_H_12_O_6_·H_2_O, ≥ 99.0%), potassium bicarbonate (KHCO_3_, ≥ 99.5%) and ammonia monohydrate (NH_3_·H_2_O, 25–28%) purchased at Sinopharm Chemical Reagents Co., Ltd. Deionized water (18.2 MΩ cm^−1^) was used in all solution preparations. All reagents were analytical grade and were used without further purification. Nitrogen (N_2_, 99.999%), carbon dioxide (CO_2_, 99.999%) were purchased from Datong Co., Ltd. The electrolytes were prepared on a daily basis and stored at ambient conditions.

### Synthesis of catalysts

Synthesis of 7% δ-Bi_2_O_3_/La_2_O_3_. Based on a hydrothermal-calcination two-step method, in a typical experiment, glucose (5.0 mmol) was dissolved into deionized water (40 mL) under stirring, followed by the addition of acrylamide (7.5 mmol), Bi(NO_3_)_3_·5H_2_O (0.23 mmol), and La(NO_3_)_3_·6H_2_O (2.27 mmol) to form a transparent solution. After that, 1.6 mL of 25 wt% ammonia solution was added dropwise under stirring, and a milk-white stiff gel formed. The pH value of the resultant gelatinous mixture was about 10. The gelatinous mixture was stirred for 5 h before being transferred into a 80 mL Teflon-lined autoclave. The autoclave was then sealed and kept at 180 °C for 72 h in an electric oven. After that, the autoclave was cooled down to room temperature naturally. The orange suspension and precipitate were collected by filtration, and washed with deionized water and alcohol three times. Finally, it was dried at 80 °C for more than 10 h and calcined at 800 °C for 4 h in air at a heating rate of 2 °C min^–1^. By altering the mass ratios of added Bi(NO_3_)_3_·5H_2_O and La(NO_3_)_3_·6H_2_O, 1% δ-Bi_2_O_3_/La_2_O_3_ (0.08 mmol Bi(NO_3_)_3_·5H_2_O and 2.40 mmol La(NO_3_)_3_·6H_2_O), 3% δ-Bi_2_O_3_/La_2_O_3_ (0.12 mmol Bi(NO_3_)_3_·5H_2_O and 2.37 mmol La(NO_3_)_3_·6H_2_O), 10% δ-Bi_2_O_3_/La_2_O_3_ (0.31 mmol Bi(NO_3_)_3_·5H_2_O and 2.18 mmol La(NO_3_)_3_·6H_2_O), and 15% δ-Bi_2_O_3_/La_2_O_3_ (0.39 mmol Bi(NO_3_)_3_·5H_2_O, 2.11 mmol La(NO_3_)_3_·6H_2_O) catalysts were obtained, respectively.

Synthesis of pure La_2_O_3_. Glucose (5.0 mmol) was dissolved into deionized water (40 mL) under stirring, followed by the addition of acrylamide (7.5 mmol) and La(NO_3_)_3_·6H_2_O (2.5 mmol) to form a transparent solution. After that, 1.6 mL of 25 wt% ammonia solution was added dropwise under stirring, and a milk-white stiff gel formed. The pH value of the resultant gelatinous mixture was about 10. The gelatinous mixture was stirred for 5 h before being transferred into a 80 mL Teflon-lined autoclave. The autoclave was then sealed and kept at 180 °C for 72 h in an electric oven. After that, the autoclave was cooled down to room temperature naturally. The orange suspension and precipitate were collected by filtration, and washed with deionized water and alcohol three times. Finally, it was dried at 80 °C for more than 10 h and calcined at 800 °C for 4 h in air at a heating rate of 2 °C min^–1^.

Synthesis of pure Bi_2_O_3_. Glucose (5.0 mmol) was dissolved into deionized water (40 mL) under stirring, followed by the addition of acrylamide (7.5 mmol) and Bi(NO_3_)_3_·5H_2_O (2.5 mmol) to form a transparent solution. After that, 1.6 mL of 25 wt% ammonia solution was added dropwise under stirring, and a milk-white stiff gel formed. The pH value of the resultant gelatinous mixture was about 10. The gelatinous mixture was stirred for 5 h before being transferred into a 80 mL Teflon-lined autoclave. The autoclave was then sealed and kept at 180 °C for 72 h in an electric oven. After that, the autoclave was cooled down to room temperature naturally. The orange suspension and precipitate were collected by filtration, and washed with deionized water and alcohol three times. Finally, it was dried at 80 °C for more than 10 h and calcined at 800 °C for 4 h in air at a heating rate of 2 °C min^–1^.

Synthesis of δ-Bi_2_O_3_/La_2_O_3_ catalyst with ~20 nm δ-Bi_2_O_3_. Glucose (5.0 mmol) was dissolved into deionized water (40 mL) under stirring, followed by the addition of acrylamide (7.5 mmol), Bi(NO_3_)_3_·5H_2_O (0.23 mmol), and La(NO_3_)_3_·6H_2_O (2.27 mmol) to form a transparent solution. After that, 1.6 mL of 25 wt% ammonia solution was added dropwise under stirring, and a milk-white stiff gel formed. The pH value of the resultant gelatinous mixture was about 10. The gelatinous mixture was stirred for 5 h before being transferred into a 80 mL Teflon-lined autoclave. The autoclave was then sealed and kept at 180 °C for 72 h in an electric oven. After that, the autoclave was cooled down to room temperature naturally. The orange suspension and precipitate were collected by filtration, and washed with deionized water and alcohol three times. Finally, it was dried at 80 °C for more than 10 h and calcined at 900 °C for 4 h in air at a heating rate of 2 °C min^–1^.

### Fabrication of the working electrodes

The catalyst ink was formed by mixing 4 mg of catalyst, 1 mg of Vulcan XC-72 carbon black (Cabot Corporation), 50 μL of 5 wt% Nafion solution (Sigma‑Aldrich), and 950 μL of ethanol (99.9%) together to form a homogeneous ink by ultrasonicating for at least 60 min. This blending treatment with conductive carbon black would significantly enhance the conductivity of the catalyst layer (CL). Then, 250 μL of homogeneous ink was evenly spray-coated onto a PTFE-modified gas diffusion layer (GDL, 1 cm^2^, SGL 38BC, Germany) surface under ambient conditions. The catalyst loading was controlled to be 1 mg cm^−2^ by weighing.

### Characterization

Transmission electron microscopy (TEM) was conducted using a JEOL JEM-2100F electron microscope operated at 200 kV. Spherical aberration (Cs)-corrected HAADF-STEM images and energy-dispersive X-ray spectroscopy (EDS) mapping were carried out on the JEM ARM300F Grand ARM electron microscope. X-ray diffraction (XRD) characterization was performed on a Philips X’Pert Pro Super diffractometer (Cu Kα, *λ* = 1.5418 Å). X-ray photoelectron spectra (XPS) data were acquired with an ESCALAB 250 instrument using Al Kα radiation. Charge-induced shifts in binding energy were corrected by referencing the C1s signal to 284.8 eV during spectral analysis. EPR measurements were performed on a Bruker EPR EMXplus-9.5/12 spectrometer at a low temperature of 194 K to enhance detection sensitivity. ^1^H NMR was performed on a BRUKER ADVANCE-Ⅲ 600 HD (Switzerland). The nitrogen adsorption-desorption isotherms at 77 K were measured using a NOVA4000 Surface Area & Pore Size Analyzer (Quantachrome Instruments, Boynton Beach, FL) after the samples were vacuum-dried at 200 °C for at least 10 h. The work functions were determined using ultraviolet photoelectron spectroscopy (UPS). UPS measurements were performed on a Thermo Scientific K-Alpha^+^ photoelectron spectrometer equipped with a He I discharge lamp (photon energy = 21.2 eV) and a pass energy of 5 eV. Bi L_3_-edge X-ray absorption spectra (XAS) measurements were performed at the BL14W1 beamline of the Shanghai Synchrotron Radiation Facility (SSRF). The XAS data were processed in Athena software using standard procedures: background subtraction, edge-step normalization, and Fourier transformation. EXAFS fitting of the Fourier-transformed data was performed in Artemis based on structural models derived from reference compounds. The normalized χ(E) spectra were converted to k-space, and the *k*^2^-weighted χ(*k*) functions (*k* = 3–10.8 Å^–1^ for the Bi L_3_-edge) were Fourier-transformed into R-space to resolve contributions from different coordination shells.

### Electrocatalytic reduction of CO_2_

All electrochemical measurements were conducted at room temperature (25 °C) and ambient pressure. Conventional CO_2_RR tests were carried out in a gas-tight H-type glass electrochemical cell separated by a cation-exchange membrane (Nafion 117, DuPont, 178 μm thick). Prior to use, the membrane was pretreated by sequential boiling: first in a 5 wt% H_2_O_2_ solution at 80 °C for 1 h, then in deionized water at 80 °C for 1 h, followed by immersion in 5 wt% H_2_SO_4_ at 80 °C for 3 h, and finally rinsed in deionized water at 80 °C for 1 h. The cell was operated at 25 °C and equipped with an Ag/AgCl (saturated KCl) reference electrode and a platinum mesh counter electrode. A CHI 760E electrochemical workstation (CH Instruments, Chenhua Co., Ltd.) was used for the H-cell electrochemical measurements. Before the CO_2_RR experiments, the catholyte and anolyte compartments were purged with CO_2_ until the open-circuit voltage (OCV) stabilized. The uncompensated solution resistance (*R*_u_) was determined from electrochemical impedance spectroscopy (EIS) under OCV conditions over a frequency range of 100 kHz to 0.1 Hz. The Ag/AgCl (saturated KCl) reference electrode was calibrated in H_2_‑saturated electrolyte using a Pt working electrode against a reversible hydrogen electrode (RHE) via cyclic voltammetry at a scan rate of 20 mV s^−^^1^. The measured potential was 0.197 V vs. RHE at 25 °C, consistent with the theoretical value. All electrode potentials were converted to the RHE scale without iR compensation based on:

1 $${{{\rm{E}}}}({{{\rm{vs}}}}.{{{\rm{RHE}}}})={{{\rm{E}}}}({{{\rm{vs}}}}.{{{\rm{Ag}}}}/{{{\rm{AgCl}}}})+0.197\,{{{\rm{V}}}}+0.0591\times {{{\rm{pH}}}}$$

The pH of the solutions was measured using a Mettler-Toledo S400 benchtop pH meter. For H-cell and Flow cell measurements, the catholyte was 0.5 M KHCO_3_ aqueous solution saturated with CO_2_, with a measured pH of 7.22 ± 0.05. For MEA measurements, humified CO_2_ flowed through the cathode side, and 1.0 M KHCO_3_ was used as the anolyte (pH = 8.34 ± 0.06). Each compartment or reservoir held 65 mL of electrolyte. The electrolyte was pre-saturated with CO_2_ via continuous sparging for at least 30 min. During CO_2_ reduction, CO_2_ was maintained at a constant flow rate of 20 mL min^–1^ and directly routed into the gas sampling loop of Shimadzu GC-2014 gas chromatograph. The system featured a thermal conductivity detector (TCD) for H_2_ quantification and a flame ionization detector (FID) with a methanizer for CO analysis. Separation of gaseous components was achieved using two Porapak N 80/100 columns packed with 13X molecular sieves, with high-purity argon as the carrier gas. The GC system was calibrated using certified standard gas mixtures of known concentrations purchased from YJ Technical Company.

The faradaic efficiencies (FEs) of CO and H_2_ production were calculated as below:

2 $${{{\mathrm{FE}}}}_{{{s}}}=\frac{2F{\nu }_{{{{\rm{s}}}}}G{P}_{0}}{R{T}_{0}{i}_{{total}}}\times 100\%$$

3 $${{{\mathrm{FE}}}}_{{{s}}}=\frac{2\times 96485(\frac{{{{\rm{C}}}}}{{{\mathrm{mol}}}})\times {\nu }_{{{{\rm{s}}}}}\times G(\frac{{{\mathrm{mL}}}}{\min })\times {10}^{-6}(\frac{{{{{\rm{m}}}}}^{3}}{{{\mathrm{mL}}}})\times 1.01\times {10}^{5}(\frac{{{{\rm{N}}}}}{{{{{\rm{m}}}}}^{2}})}{8.314\left(\frac{{{\mathrm{N\; m}}}}{{{\mathrm{mol\; K}}}}\right)\times 298.15(K)\times {i}_{{total}}(\frac{{{{\rm{C}}}}}{{{{\rm{s}}}}})\times 60(\frac{{{{\rm{s}}}}}{\min })}\times 100\%$$

where *v*_s_ (vol%) denotes the volumetric concentration of species *s* (i.e., CO or H_2_) in the exhaust gas from the electrochemical cell, as determined by gas chromatography (GC), *P*_0_ = 1.013 bar and *T*_0_ = 273.15 K, *G* (mL min^–1^) is the gas flow rate measured at the cell outlet using an FL-1802 rotameter, *i*_total_ (mA) represents the steady-state cell current, *F* = 96485 C mol^−1^ (Faraday constant), *R* = 8.314 ⋅ J mol^−1^ ⋅ K^−1^.

The amount of liquid products was quantified by ^1^H NMR spectroscopy (AV 600). Briefly, 15 μL of dimethyl sulfoxide (DMSO, 99.99%, Sigma) was added as the internal standard to 50 mL of catholyte. Then, 0.5 mL of the resulting mixture was thoroughly mixed with 0.1 mL D_2_O to prepare the NMR samples. Given that formate is generated via a two-electron reduction process, the faradaic efficiency for formate was calculated as follows: FE_formate_ = 2 *F* × *n*_formate_/(*i*_total_ × t), where *F* is the Faraday constant and t is the electrolysis time. The partial current densities of formate production were calculated as below:

4 $${j}_{{formate}}={{{\mathrm{FE}}}}_{{formate}}\times {i}_{{{\mathrm{total}}}}\times {({{\mathrm{electrode\; area}}})}^{-1}$$

where electrode area is “cm^2^”. The unit of *j* in the equations is “mA/cm^2^”.

### Flow cell assembly

The flow cell setup purchased from Gaoss Union was composed of three chambers: anolyte chamber, catholyte chamber, and gas flow chamber (Supplementary Fig. 33). All potentials are referenced to a reversible hydrogen electrode (RHE) without iR compensation. Each chamber has an inlet and outlet for electrolyte or gas. The size of the electrode exposed was 0.5 cm × 2 cm. The cathode GDE of interest (1 mg cm^−2^) was clamped between the catholyte chamber and gas diffusion chamber, with the substrate side facing the gas chamber and catalyst side facing the catholyte chamber. A Pt tablets was employed in the anolyte chamber. The catholyte and anolyte chambers were separated by an anion exchange membrane (AEM, 1 cm × 3 cm, thickness 50 μm, FAA-3-PK-130). Prior to use, the AEM was pretreated by soaking in 0.5 M KOH for 1 h, followed by another 1 h soaking in fresh 0.5 M KOH, and then rinsed thoroughly with deionized water. An Ag/AgCl reference electrode (saturated KCl) was installed in the catholyte compartment. Throughout the measurements, CO_2_ gas was introduced into the gas chamber at a flow rate of 10 mL min^–1^, and the 0.5 M KHCO_3_ aqueous electrolyte was continuously circulated through the cathode and anode compartments at 30 mL min^–1^ using a peristaltic pump and a mixed-flow pump, respectively. Electrochemical tests in the flow cell were performed using a CHI 1140c electrochemical workstation (CH Instruments, Chenhua Co.).

### MEA assembly

Flow CO_2_RR durability tests at high current densities were conducted in a commercial MEA-based electrolyzer (1 cm × 1 cm, Gaoss Union) with a two-electrode system (Supplementary Fig. 34), in which IrO_2_ (0.2 mg cm^−2^) and VXC-72 Cabot black carbon foam (0.8 mg cm^−2^) were ultrasonically mixed served as the anode. The catalysts (1 mg cm^−2^) served as the cathode. The full-cell potentials in the two-electrode MEA system were shown without iR correction. An anion-exchange membrane (AEM, 1 cm × 1 cm, thickness 50 μm, FAA-3-PK-130) was located between the cathode and anode to separate the chambers. During the measurements, CO_2_ gas was continuously supplied into the cathode flow field after being humidified in 100 °C deionized water at a flow rate of 10 mL min^−1^ controlled by a mass flow controller. Anolyte (1.0 M KHCO_3_) was circulated through the anode flow field at a flow rate of 30 mL min^−1^ by a mixed-flow pump. During the electrochemical tests, the electrolyzer was heated and maintained at 70 °C. The electrochemical experiments were performed using a VersaSTAT 3F Galvanostat (Ametek Scientific Instruments, Princeton Applied Research).

### In situ Raman spectra tests

In situ confocal laser Raman characterization (Lab RAM, Horiba-JY) was conducted in a three-electrode spectro-electrochemical cell containing CO_2_-presaturated 0.5 M KHCO_3_ electrolyte. The test configuration employed a δ-Bi_2_O_3_/La_2_O_3_ loaded glassy carbon working electrode, a Pt wire counter electrode, and an Ag/AgCl (saturated KCl) reference electrode (Supplementary Fig. 52). Throughout CO_2_RR, fresh CO_2_-saturated electrolyte was delivered into the cell at 30 mL min^−1^ using a peristaltic pump, which replenished CO_2_ and eliminated H_2_ bubbles to avoid distorting Raman signals. Real-time Raman spectra (50–800 cm^−1^) were collected to monitor the dynamic reaction process, with the Raman shift calibrated to 520 cm^−1^ using a standard Si wafer.

### In situ ATR-SEIRAS measurements

The Au-film working electrode on the reflective facet of a silicon prism was fabricated following a classic two-step wet-chemical method^59^. The Au-modified prism was integrated into a spectroelectrochemical cell mounted on a SPEC-I optical platform (Shanghai Yuanfang Technology), which was installed in a Nicolet iS50 FTIR spectrometer fitted with a liquid-nitrogen-cooled MCT detector. A fixed incident angle of ~60° was adopted for all ATR-SEIRAS measurements. Spectra were displayed in absorbance units (−log(I/I_0_)), with I and I_0_ representing the sample and reference spectra. Time- and potential-dependent spectral data were acquired against the I_0_ baseline. Unless otherwise stated, real-time single-beam spectra were collected for 5 s (~22 interferograms) at a spectral resolution of 8 cm^−1^. Electrochemical tests were performed using a CHI 760E electrochemical workstation (CH Instruments, Chenhua Co.). A Pt mesh and an Ag/AgCl (saturated KCl) electrode were used as the counter and reference electrodes, respectively. The dual-compartment, three-electrode ATR-SEIRAS cell is illustrated in Supplementary Fig. 55. The 0.5 M KHCO_3_ electrolyte was prepared by dissolving high-purity KHCO_3_ in Milli-Q water (18.2 MΩ cm) and purged with high-purity N_2_ or CO_2_ prior to each electrochemical measurement.

### XANES simulations

The theoretical XANES spectra were simulated using the ab initio multiple-scattering software FEFF 9.0^60^, which uses spherical muffin-tin potentials and the extended continuum approximation to treat all XANES features in terms of the scattering of continuum photoelectrons. Hedin-Lundqvist exchange correlation potential was used, and the radiuses for full multiple scattering calculations and self-consistent calculations were set to 8.0 and 5.0 Å, respectively. The maximum value of automatic overlapping of all muffin-tins was set to 1.3 to reduce the effects of potential discontinuities. All other parameters were set to default values in FEFF 9.0. The structural models used for simulations of La_2_O_3_, alpha-Bi_2_O_3_ and beta-Bi_2_O_3_ were constructed based on the experimental data from the inorganic crystal structure database (ICSD). For delta-Bi_2_O_3_, there are different proposed models related to the arrangements of oxygen vacancies, therefore DFT optimized structures were used as input for FEFF calculations. All clusters used for calculations contains more than 120 atoms, and all possible absorption sites have been considered. Substitution doping (Bi occupying at La sites in La_2_O_3_ and La occupying at Bi sites in Bi_2_O_3_) has been simulated to determine whether the large-scale interdiffusion of Bi and La atoms occurs. The corresponding structural models have been provided in Supplementary Figs. 23 and 24.

### DFT calculations

All DFT calculations are performed using the Vienna ab initio simulation package (VASP)^61–64^ within projector-augmented wave pseudopotentials and the revised Perdew–Burke–Ernzenhof exchange correlation functional^65^. The plane-wave energy cutoff was set to be 450 eV, and the thresholds for electronic and geometric structure convergence were set to be 10^−5^ eV and 0.02 eV/Å. DFT + *U* scheme with *U* = 7.5 eV was applied to the La atom in the investigated systems^66^. The cubic bulk structure of δ-Bi_2_O_3_ was fully optimized within the primitive cell using a 9 × 9 × 9 Monkhorst-pack k-point mesh, and was then adopted to build the slab model. The optimized lattice parameters *a* = *b* = *c* = 5.62 Å agree well with the experimental values of 5.65 Å^67^. A supercell was built to represent the Bi_2_O_3_(111) surface, and the parameters are of *a* = *b* = 7.90 Å and *γ* = 120°. The lattice parameters (*a* = *b* = 3.96 Å, c = 6.24 Å, *α* = *β* = 90°, *γ* = 120°) of the optimized bulk La_2_O_3_ are also in good agreement with the experimental values (*a* = *b* = 3.94 Å, c = 6.13 Å, *α* = *β* = 90°, *γ* = 120°^68^. A supercell consisting of 2 × 2 unit cells for La_2_O_3_(001) surface was built, and the parameters of *a* = *b* = 7.92 Å and *γ* = 120° are in good agreement with the parameters of the supercell for Bi_2_O_3_(111) surface. Therefore, the heterostructure of δ-Bi_2_O_3_/La_2_O_3_ was constructed with the La_2_O_3_(001) surface as a support, of which the parameters were fixed for the heterostructure model. All slab models had a minimum of 9 atomic layers with 4–5 atomic layers at the bottom fixed, and the top La layer was allowed to relax in the heterostructure. A vacuum spacing of approximately 15 Å was set to avoid interference between adjacent slabs. In this study, 5 × 5 × 1 k-point sampling was used for all slab calculations. For all of the species adsorbed on the surfaces, many different configurations were considered as starting structures for optimizations, and the configuration with the lowest energy was considered as the optimal structure for each species.

The Gibbs free energy of CO_2_ reduction and hydrogen evolution reactions were calculated based on the computational hydrogen electrode (CHE). The van der Waals interactions were included by Grimme’s D3 method^69^. For the HER pathway, the Gibbs free energies Δ*G* were calculated by the binding energies Δ*E* with corrections for zero-point energy (Δ*E*_ZPE_) and entropy (*S*), according to

5 $$\Delta G=\Delta E+\Delta {E}_{{{\mathrm{ZPE}}}}-T\Delta S$$

Zero-point energy corrections are calculated by summing vibrational frequencies over all normal modes, and the temperature *T* was set to 298 K. The Gibbs free energy for the adsorption of *H on the surface was calculated by Δ*G*_H*_ = Δ*E*_H*_ + 0.30 eV^70^.

The surface Pourbaix diagrams were plotted under the relevant *U*_SHE_ (*U*_RHE_ = *U*_SHE_ + 0.059 × pH) and pH. The hydrogenation process of the O* species on the surfaces of catalysts were assumed to occur with the following step:

6 $${{O}} \ast+{{{H}}}^{+}+{{{e}}}^{-}\to {OH}*$$

The Gibbs free energy of eq (S2) was is obtained

7 $$\Delta G=\Delta E+\Delta {E}_{{ZPE}}-T\Delta S$$

The effects of potential *U*_SHE_ and pH were included by:

8 $$\Delta G^{\prime}=\Delta G-{k}_{b}T{ln}10\times {{\mbox{pH}}}-e{U}_{{SHE}}$$

The Bader charges^71^ are calculated to analyze the charge transfer. The values for the integrated projected crystal orbital Hamilton population (IpCOHP) up to the Fermi level were computed to evaluate the bond strengh^56,57^. In addition, the integrated crystal orbital bond index (ICOBI) was calculated to quantify covalent bonding in solid-state materials^58^. For the electronic structure calculations, 9 × 9 × 1 *k*-point mesh within the Monkhorst-Pack scheme was applied.

## Supplementary information

Supplementary Information

Description of Additional Supplementary Files

Supplementary Data 1

Transparent Peer Review File

## Source data

Source Data

## Data Availability

The data generated in this study are provided in the Supplementary Information. All figures in the manuscript are original and were created by the authors, with no copyright disputes. Figure 1a and Supplementary Figs. 1, 46 (illustration), and 55 were produced by Q.M.W., C.L., and F.Y. using Autodesk 3ds Max 2018. Source data are provided with this paper.
