# Supplementary material for: Stabilizing sub-2 nm δ-Bi2O3 via strong lanthanide-oxide-support interaction for durable CO2 electroreduction to formate
Source: Nat Commun. 2026 Apr 24;17:5685. doi: 10.1038/s41467-026-71855-5 (PMC13319208; doi:10.1038/s41467-026-71855-5)
Supplement: Supplementary file 1 — Supplementary Information [file 41467_2026_71855_MOESM1_ESM.pdf]

Supplementary Information

Stabilizing Sub-2 nm δ-Bi_2O_3 via Strong Lanthanide-Oxide-Support Interaction for Durable CO_2 Electroreduction to Formate

Qianmin Wu[1,2,†], Cui Li[1,†], Yuxuan Wu[1,†], Qing Liang[3,†], Xuyu Lv[1], Yanhong Li[1], Chang Wang[1], Mengjie Wu[1], Lichun Kong[1], Ji-Qing Lu[1], Wei Zhang[3,*], Zhengquan Li[1,2,*], De-Li Chen[1,*], Jing Zhou[2,*], Fa Yang[1,2,*]

[1]Qianmin Wu, Cui Li, Yuxuan Wu, Xuyu Lv, Yanhong Li, Chang Wang, Mengjie Wu, Lichun Kong, Ji-Qing Lu, Zhengquan Li, De-Li Chen, Fa Yang
Key Laboratory of the Ministry of Education for Advanced Catalysis Materials, Zhejiang Key Laboratory of Advanced Catalysis and Adsorption Materials, College of Chemistry and Materials Science, Zhejiang Normal University, Jinhua, Zhejiang 321004, China.
*Corresponding Author E-mail: zqli@zjnu.edu.cn; chendl@zjnu.cn; yangfa@zjnu.edu.cn
[2]Qianmin Wu, Zhengquan Li, Jing Zhou, Fa Yang
Zhejiang Institute of Photoelectronics, Zhejiang Normal University, Jinhua, Zhejiang 321004, China.
*Corresponding Author E-mail: zhoujing5457@zjnu.edu.cn
[3]Qing Liang, Wei Zhang
Key Laboratory of Automobile Materials MOE, School of Materials Science & Engineering, Electron Microscopy Center, Jilin University, Changchun 130012, China.
*Corresponding Author E-mail: weizhang@jlu.edu.cn
[†]These authors contributed equally: Qianmin Wu, Cui Li, Yuxuan Wu, Qing Liang.

Table of Contents

1. Supplementary Figures (Figure S1-S81)
2. Supplementary Tables (Table S1-S7)
3. References

Supporting Figures and Tables

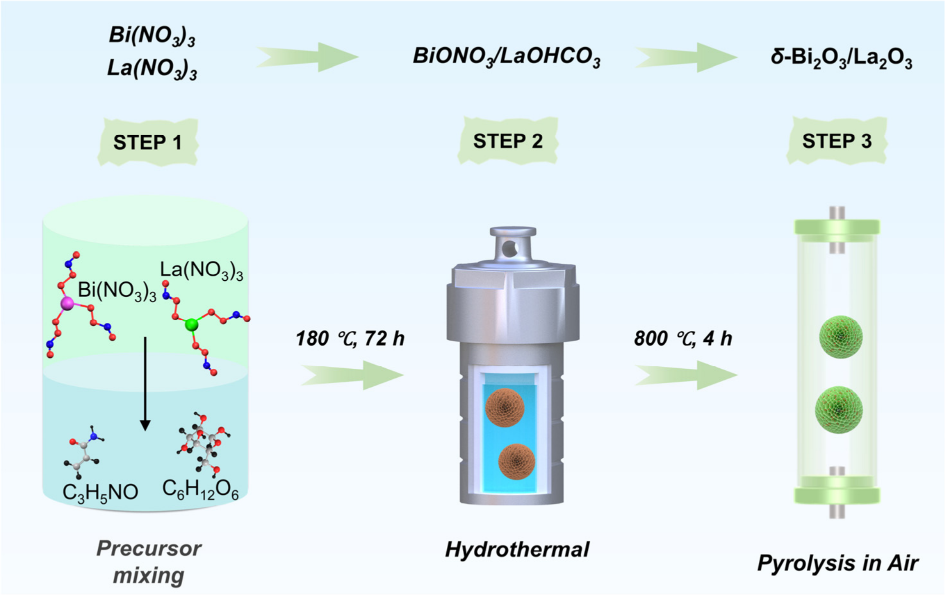

Supplementary Fig. 1 | Schematic illustration of the synthesis procedure for the δ-Bi_2O_3/La_2O_3 catalysts.

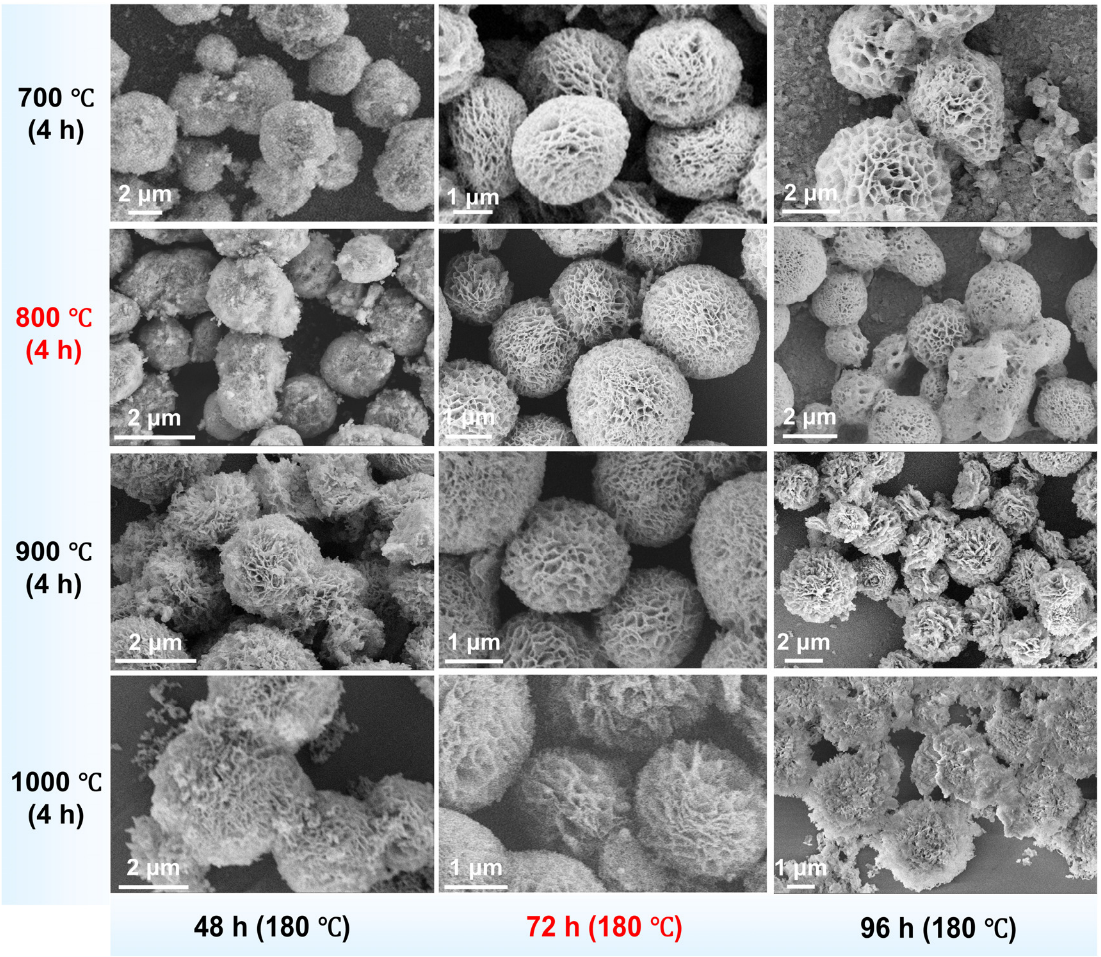

Supplementary Fig. 2 | Typical SEM images of experimental optimization for hydrothermal and calcination condition.

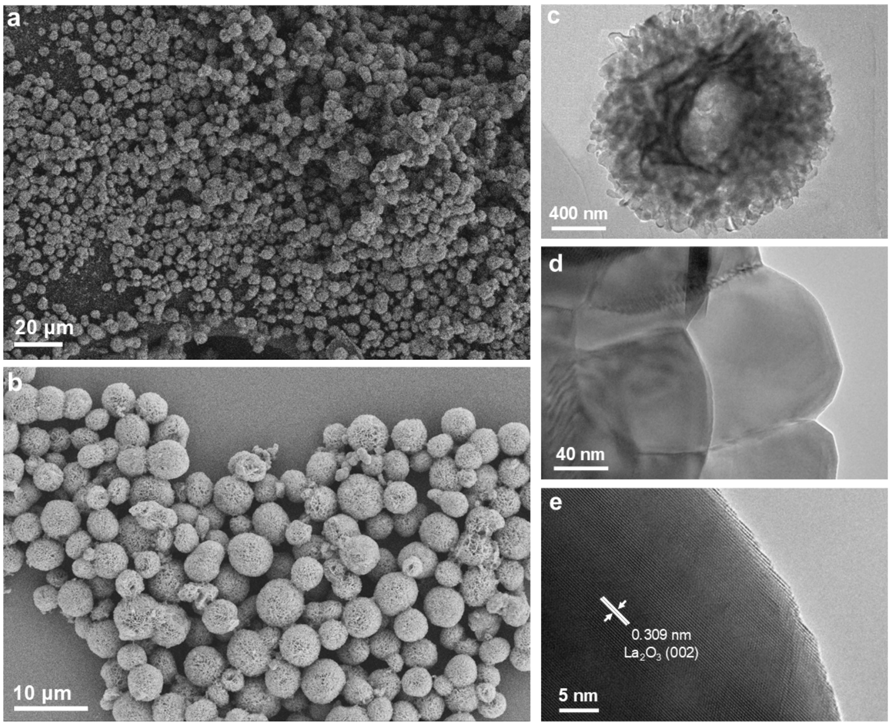

Supplementary Fig. 3 | (a, b) SEM, (c, d) TEM, and (e) HRTEM images of pure La_2O_3 microspheres.

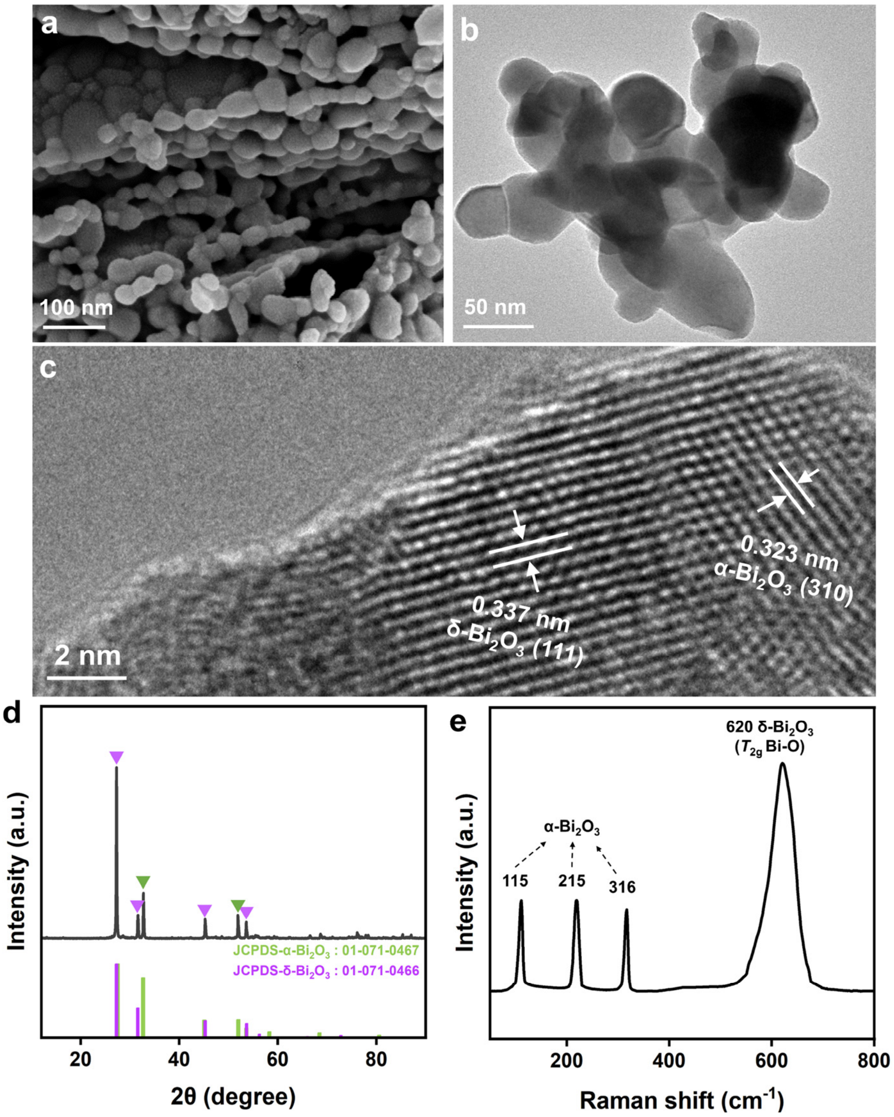

Supplementary Fig. 4 | (a) SEM, (b) TEM, (c) HRTEM, (d) XRD pattern, and (e) Raman spectrum of pure Bi_2O_3 powder. Source data are provided as a Source Data file.

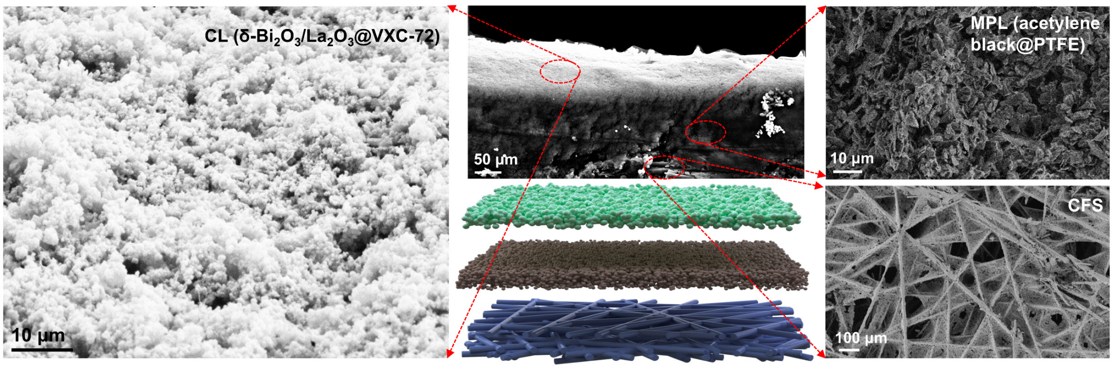

Supplementary Fig. 5 | Architecture and corresponding SEM images of a gas diffusion layer, containing the microporous layer (MPL), the carbon fiber substrate, and the catalyst layer (CL). Noted, the MPL is usually composed of hydrophobic PTFE and conductive carbon black (e.g., acetylene black), which plays a key role in striking a fine balance between the hydrophobicity and conductivity of the GDE while preventing electrolyte flooding. Regarding the use of PTFE in our method, according to the manufacturer's technical specifications, this hydrophobic GDL consists of a CFS containing 5 wt% PTFE and a MPL with 20–25 wt% PTFE.

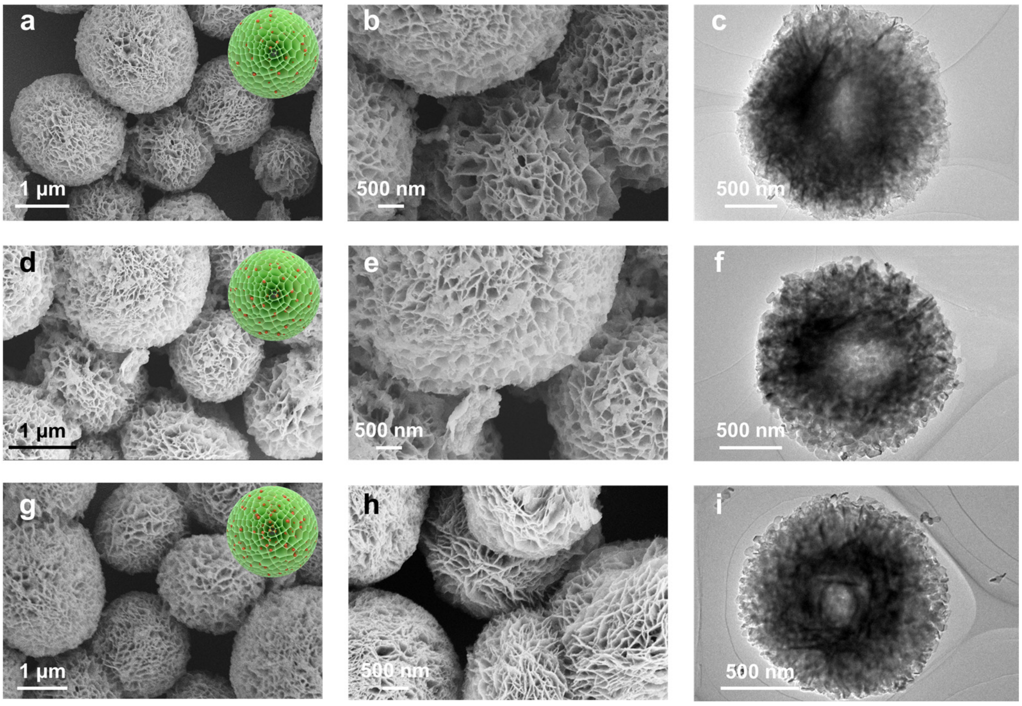

Supplementary Fig. 6 | Typical SEM and TEM images of (a-c) 1%, (d-f) 3%, and (g-i) 7% δ-Bi_2O_3/La_2O_3 sample. The insets are the corresponding 3D morphology schematics.

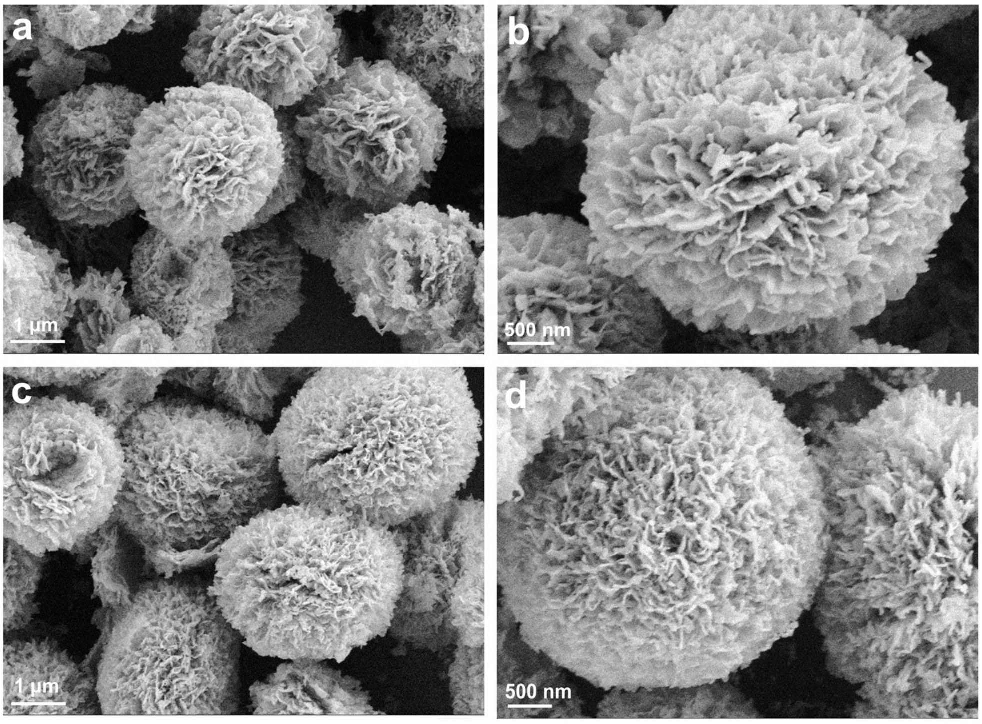

Supplementary Fig. 7 | Typical SEM images of (a-b) 10%, (c-d) 15% δ-Bi_2O_3/La_2O_3 sample.

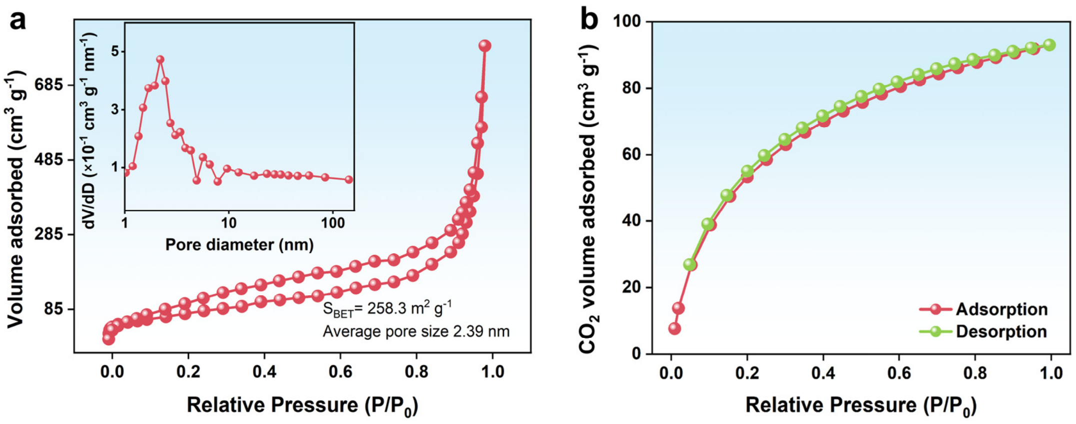

Supplementary Fig. 8 | (a) N_2 and (b) CO_2 adsorption/desorption isotherms of the 7% δ-Bi_2O_3/La_2O_3 sample. The inset in (a) is BJH pore size distribution. Source data are provided as a Source Data file.

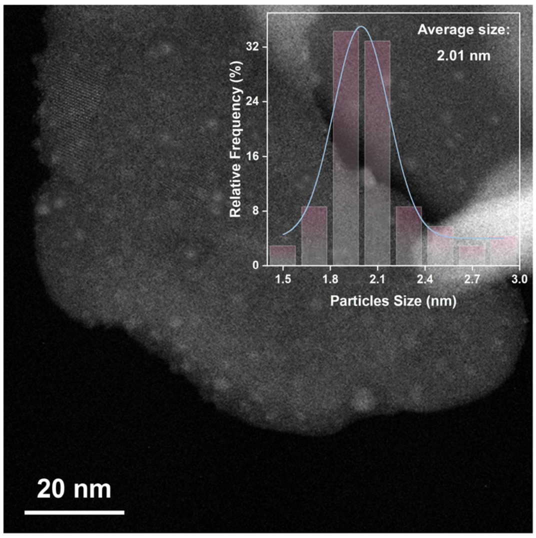

Supplementary Fig. 9 | The δ-Bi_2O_3 size distributions of 7% δ-Bi_2O_3/La_2O_3 sample. The inset is corresponding size distribution. Source data are provided as a Source Data file.

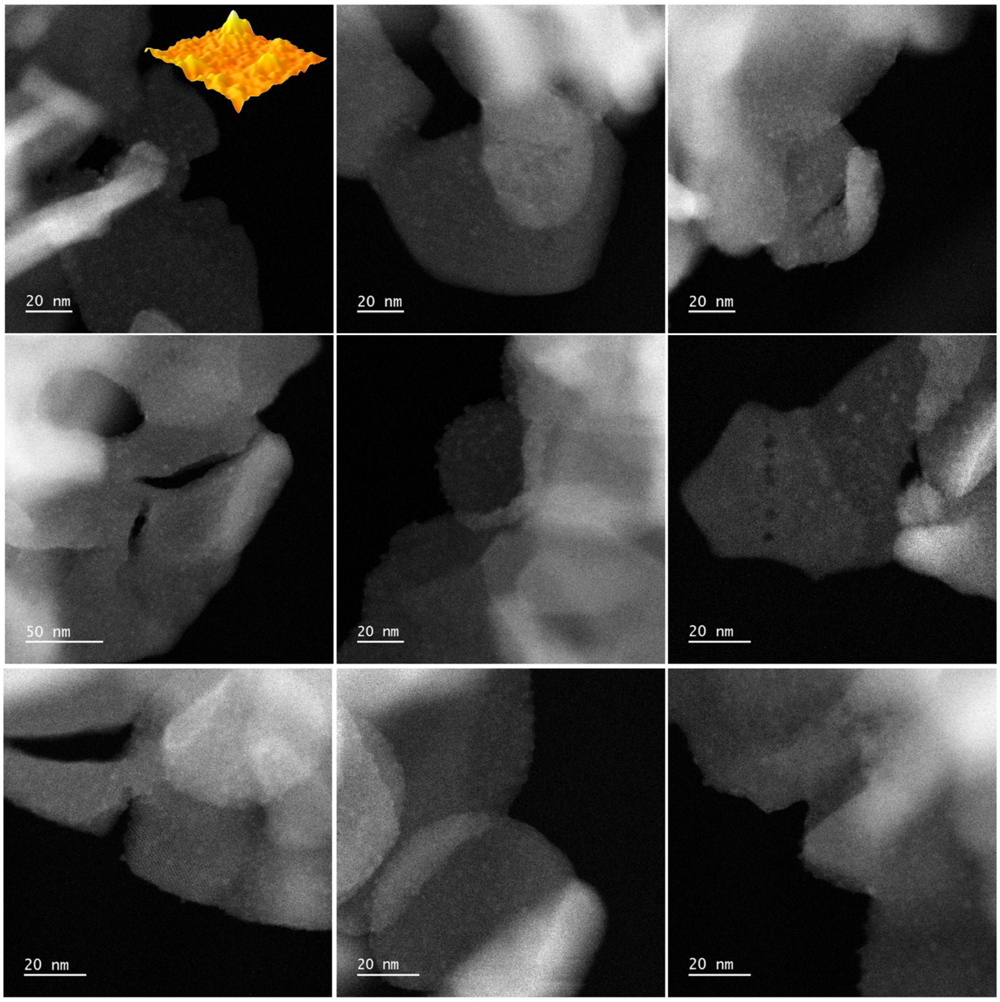

Supplementary Fig. 10 | Aberration-corrected HAADF-STEM images of 1% δ-Bi_2O_3/La_2O_3. The inset is 3D atom-overlapping Guassian function fitting mapping.

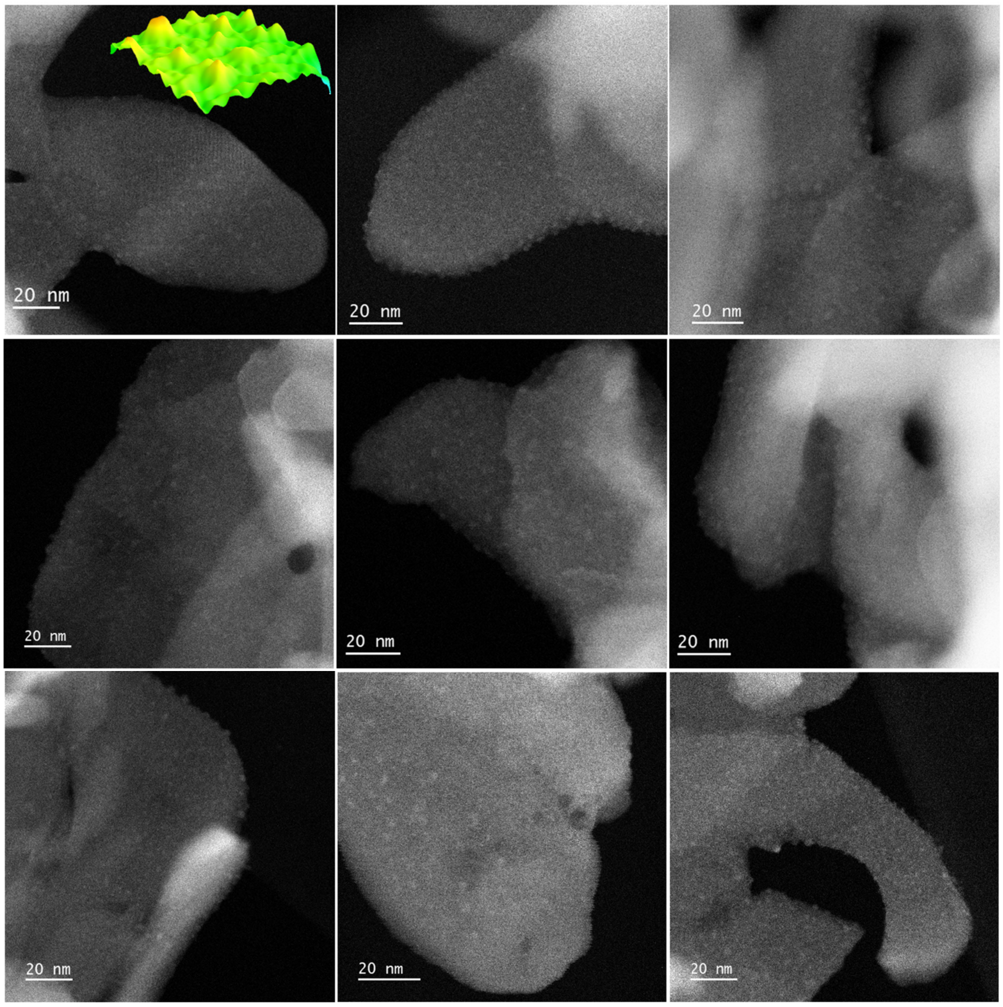

Supplementary Fig. 11 | Aberration-corrected HAADF-STEM images of 3% δ-Bi_2O_3/La_2O_3. The inset is 3D atom-overlapping Guassian function fitting mapping.

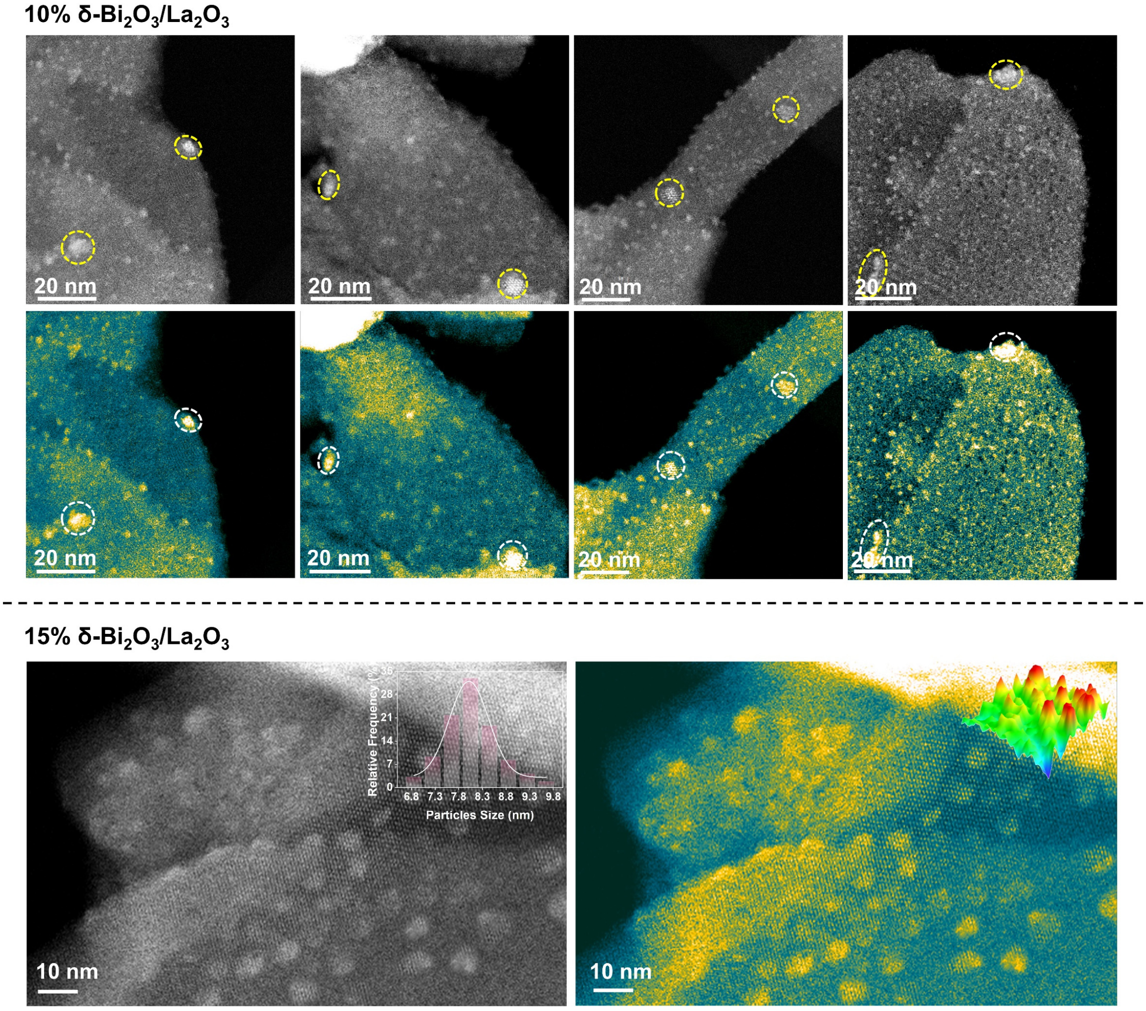

Supplementary Fig.12 | Aberration-corrected HAADF-STEM images and corresponding false-color images of 10% and 15% δ-Bi_2O_3/La_2O_3. The insets show the size distribution and the 3D atom-overlapping Gaussian function fitting mapping of 15% δ-Bi_2O_3/La_2O_3. Source data are provided as a Source Data file.

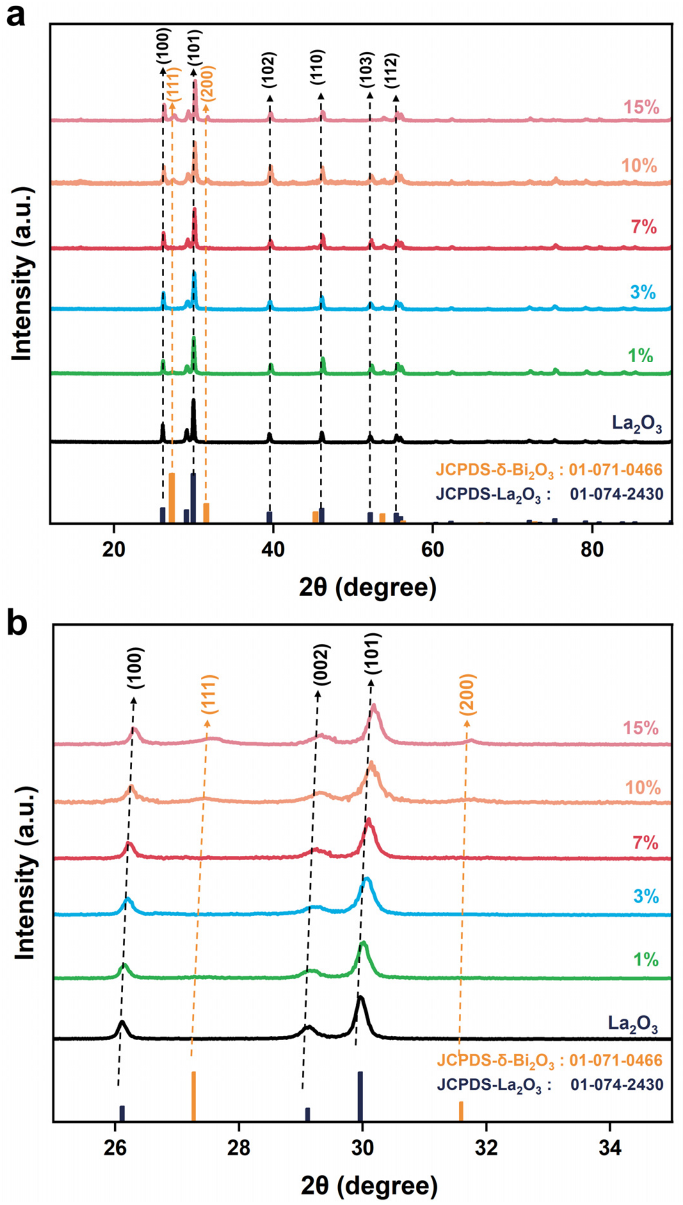

Supplementary Fig. 13 | (a) XRD patterns of the 1%, 3%, 7%, 10%, and 15% δ-Bi_2O_3/La_2O_3 samples calcined at 800 °C, with an enlarged 25–35° region shown in (b). Source data are provided as a Source Data file.

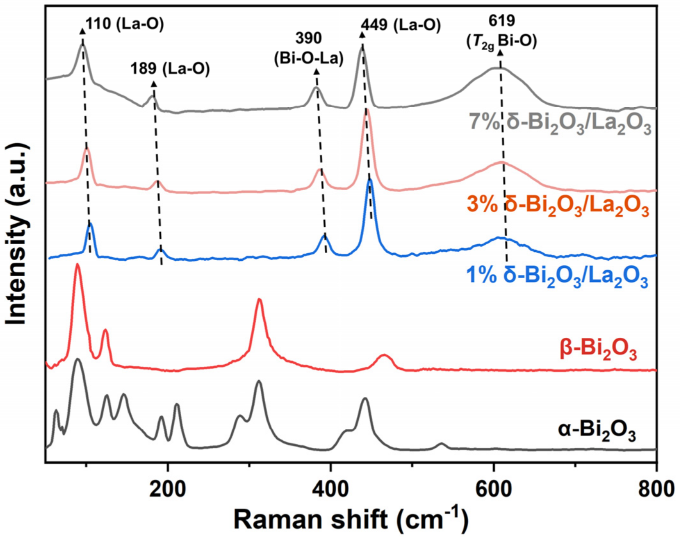

Supplementary Fig. 14 | Raman spectra of δ-Bi_2O_3/La_2O_3 with different Bi content. Source data are provided as a Source Data file.

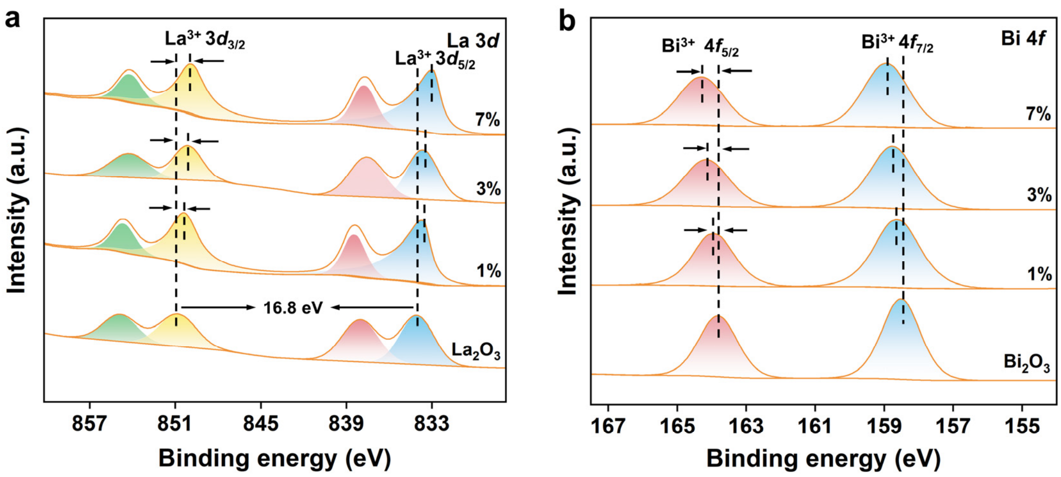

Supplementary Fig. 15 | High-resolution (a) La 3*d* and (b) Bi 4*f* XPS spectra of Bi_2O_3, La_2O_3 and δ-Bi_2O_3/La_2O_3. Source data are provided as a Source Data file.

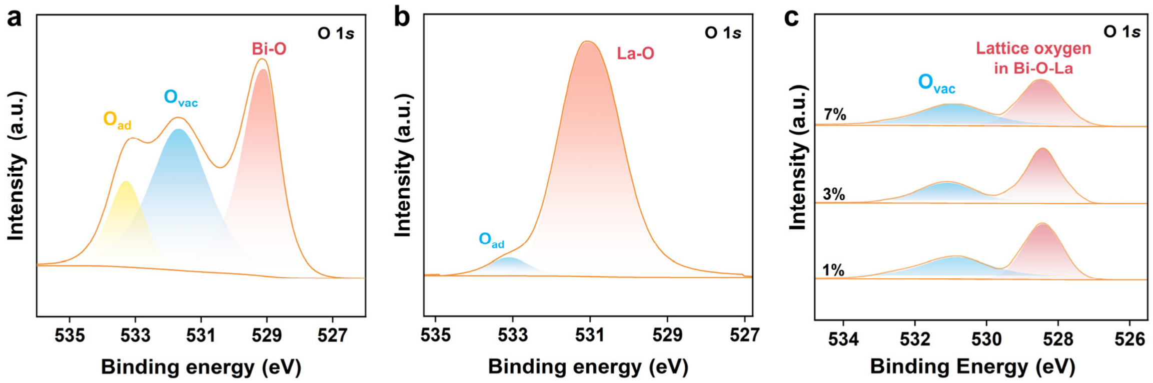

Supplementary Fig. 16 | High-resolution O 1*s* XPS spectra of (a) pure Bi_2O_3, (b) pure La_2O_3 and (c) δ-Bi_2O_3/La_2O_3 with three different Bi content. Source data are provided as a Source Data file.

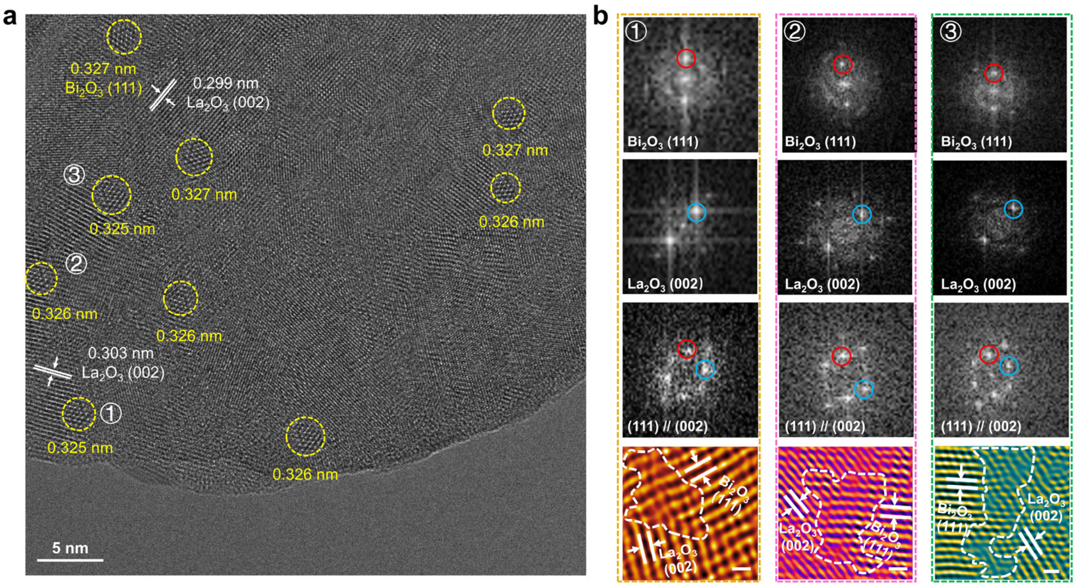

Supplementary Fig. 17 | (a) HRTEM image of 7% δ-Bi_2O_3/La_2O_3 and (b) corresponding FFT analysis of the selected region, respectively. The spots highlighted by blue and red circles correspond to the (002) plane of La_2O_3 and the (111) plane of Bi_2O_3, respectively. The scale bar in (b) is 1 nm.

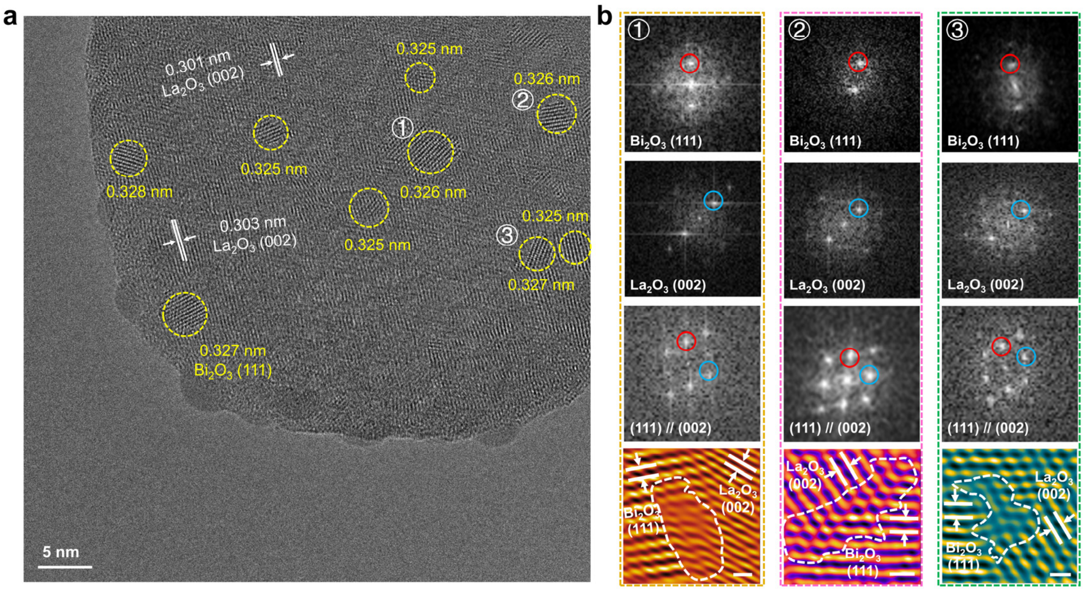

Supplementary Fig. 18 | (a) HRTEM image of 7% δ-Bi_2O_3/La_2O_3 and (b) corresponding FFT analysis of the selected region, respectively. The spots highlighted by blue and red circles correspond to the (002) plane of La_2O_3 and the (111) plane of Bi_2O_3, respectively. The scale bar in (b) is 1 nm.

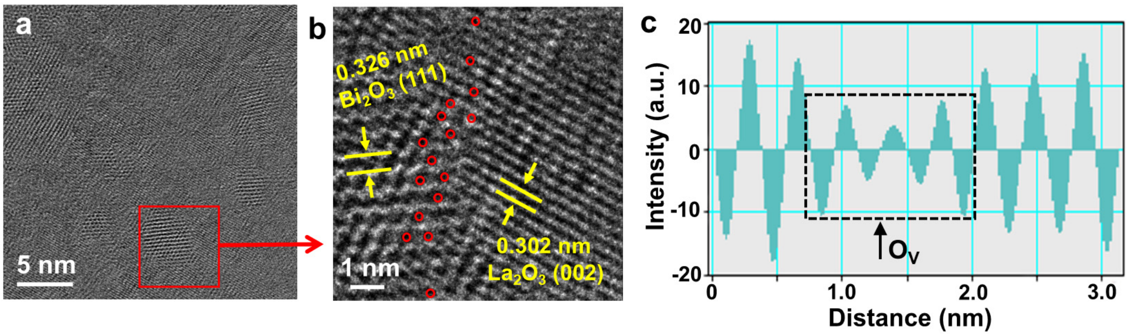

Supplementary Fig. 19 | (a) HRTEM image, (b) vacancy diagram and (c) intensity distribution map between the interface of δ-Bi_2O_3 and La_2O_3 phase.

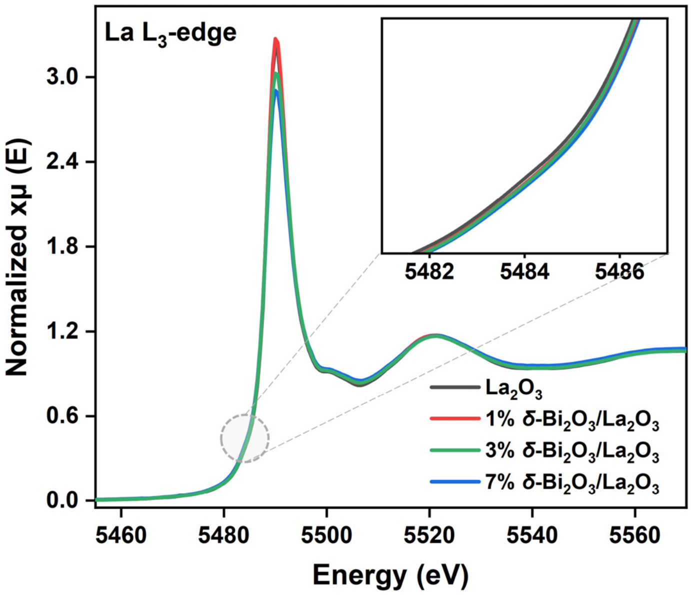

Supplementary Fig. 20 | La L_3-edge XANES spectra over pure La_2O_3 and δ-Bi_2O_3/La_2O_3. The inset show zoomed-in views of selected spectral regions. Source data are provided as a Source Data file.

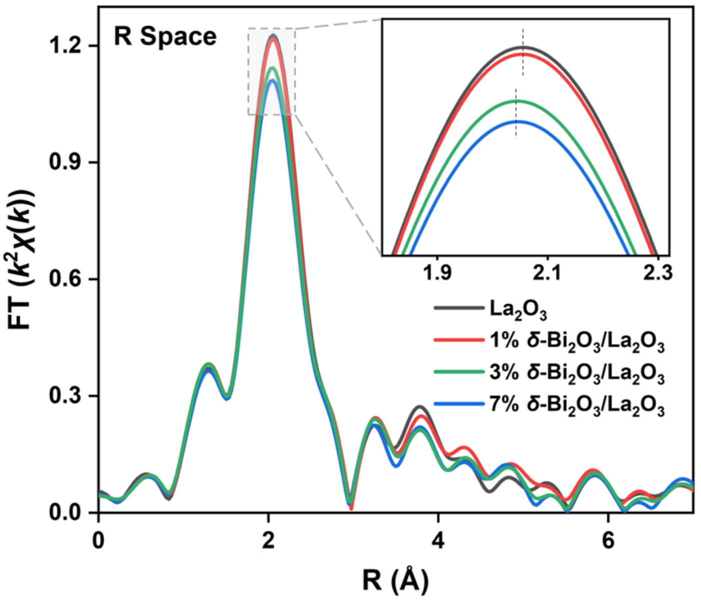

Supplementary Fig. 21 | Corresponding k^2-weighted FT-EXAFS spectra over pure La_2O_3 and δ-Bi_2O_3/La_2O_3. The inset show zoomed-in views of selected spectral regions. Source data are provided as a Source Data file.

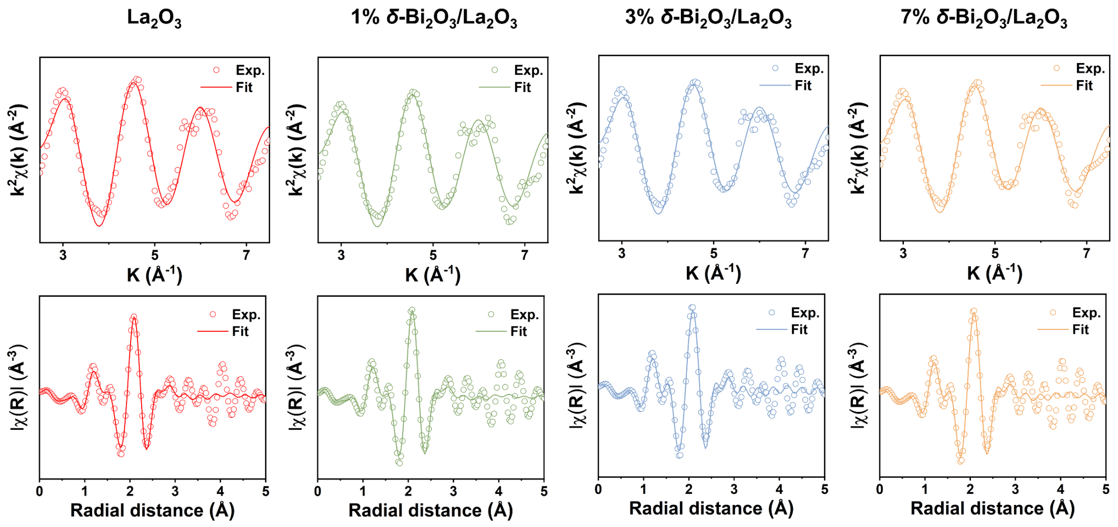

Supplementary Fig. 22 | k^2-weighted La L_3-edge EXAFS oscillations (up) and their associated Fourier transforms (down) for pure La_2O_3 and δ-Bi_2O_3/La_2O_3; Experimental data, dotted lines; Fitting curves, solid lines. Source data are provided as a Source Data file.

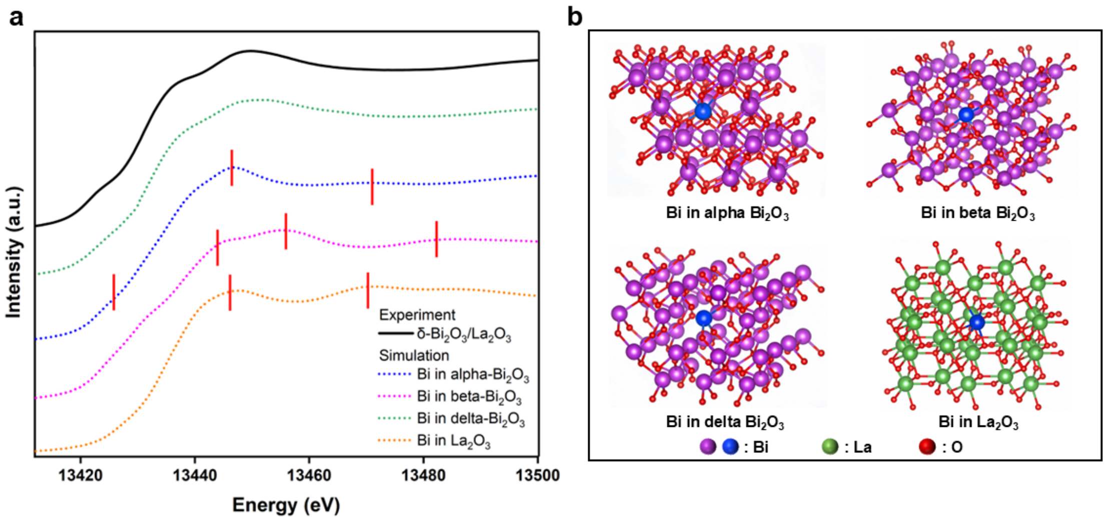

Supplementary Fig. 23 | (a) Comparison of the experimental Bi L_3-edge XANES spectrum of the δ-Bi_2O_3/La_2O_3 sample with simulated spectra for various Bi_2O_3 phases and Bi-doped La_2O_3 models. (b) The DFT-optimized structural models used for theoretical simulations. Source data are provided as a Source Data file.

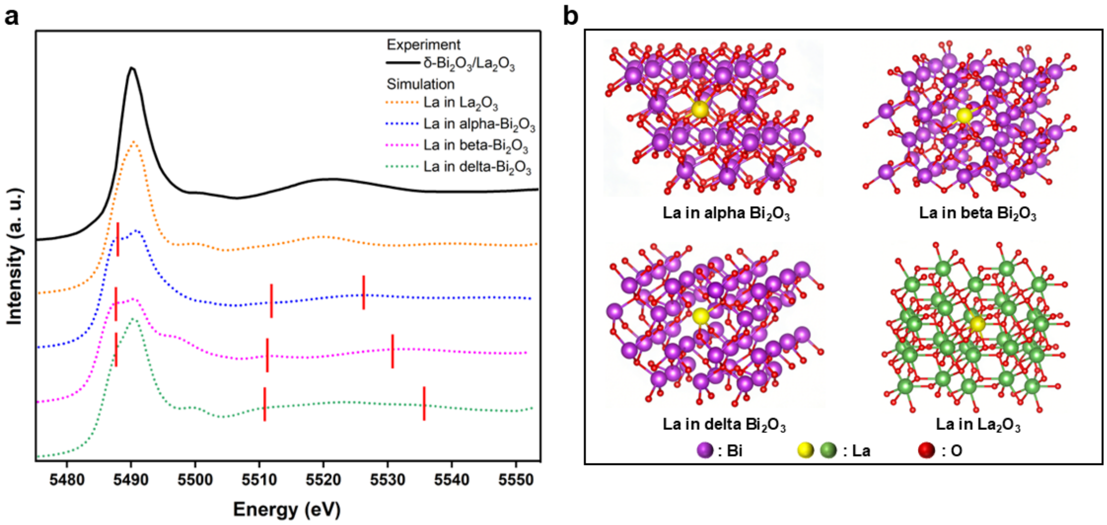

Supplementary Fig. 24 | (a) Comparison of the experimental La L_3-edge XANES spectrum of the δ-Bi_2O_3/La_2O_3 sample with simulated spectra for various Bi_2O_3 phases and La-doped La_2O_3 models. (b) The DFT-optimized structural models used for theoretical simulations. Source data are provided as a Source Data file.

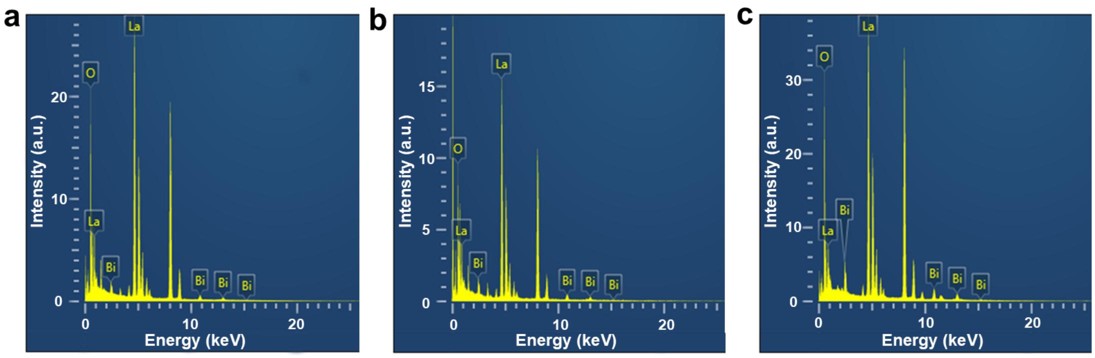

Supplementary Fig. 25 | EDS analysis of δ-Bi_2O_3/La_2O_3 with different Bi content: (a) 1% δ-Bi_2O_3/La_2O_3, (b) 3% δ-Bi_2O_3/La_2O_3, and (c) 7% δ-Bi_2O_3/La_2O_3.

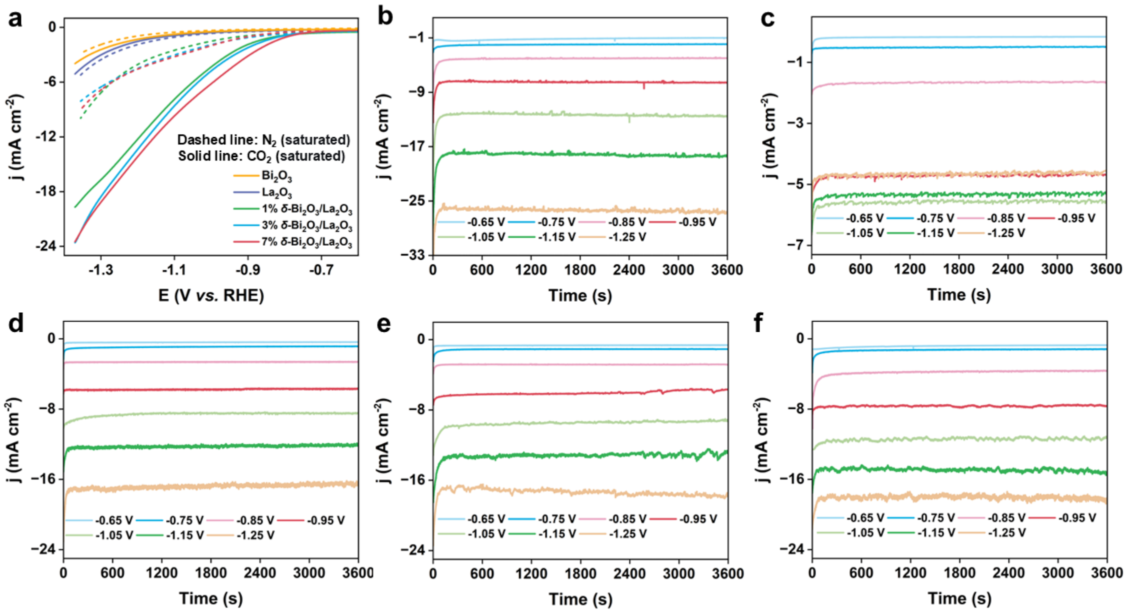

Supplementary Fig. 26 | (a) LSV curves at a scan rate of 10 mV s^{-1} in N_2-saturated 0.5 M $KHCO_3$ electrolyte (pH = 8.43 ± 0.01) and CO_2-saturated 0.5 M $KHCO_3$ electrolyte (pH = 7.22 ± 0.05). Chronoamperometry curves at various potentials over (b) pure La_2O_3, (c) pure Bi_2O_3, (d) 1% δ-Bi_2O_3/La_2O_3, (e) 3% δ-Bi_2O_3/La_2O_3, and (f) 7% δ-Bi_2O_3/La_2O_3 catalysts. Catalyst mass loading was fixed at 1 mg cm^{-2}. Typical solution resistance in CO_2-saturated 0.5 M $KHCO_3$ is determined as 5.3 ± 0.2 Ω. Source data are provided as a Source Data file.

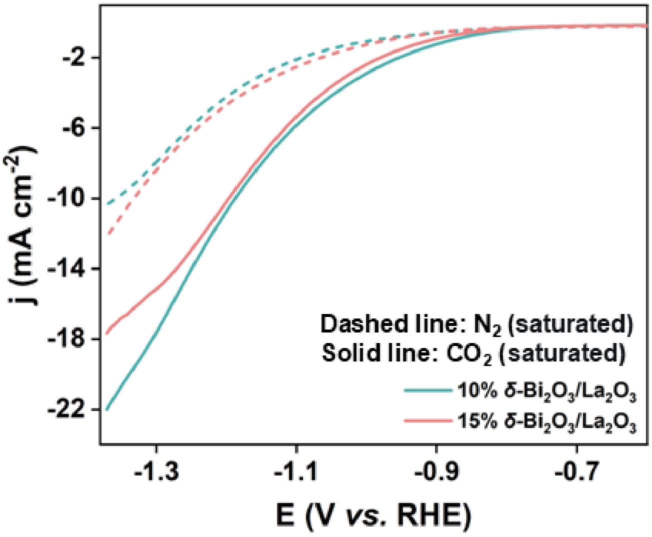

Supplementary Fig. 27 | LSV curves of 10% and 15% δ-Bi_2O_3/La_2O_3 catalysts at a scan rate of 10 mV s^{-1} in N_2-saturated 0.5 M $KHCO_3$ electrolyte (pH = 8.43 ± 0.01) and CO_2-saturated 0.5 M $KHCO_3$ electrolyte (pH = 7.22 ± 0.05). Catalyst mass loading was fixed at 1 mg cm^{-2}. Typical solution resistance in CO_2-saturated 0.5 M $KHCO_3$ is determined as 5.3 ± 0.2 Ω. Source data are provided as a Source Data file.

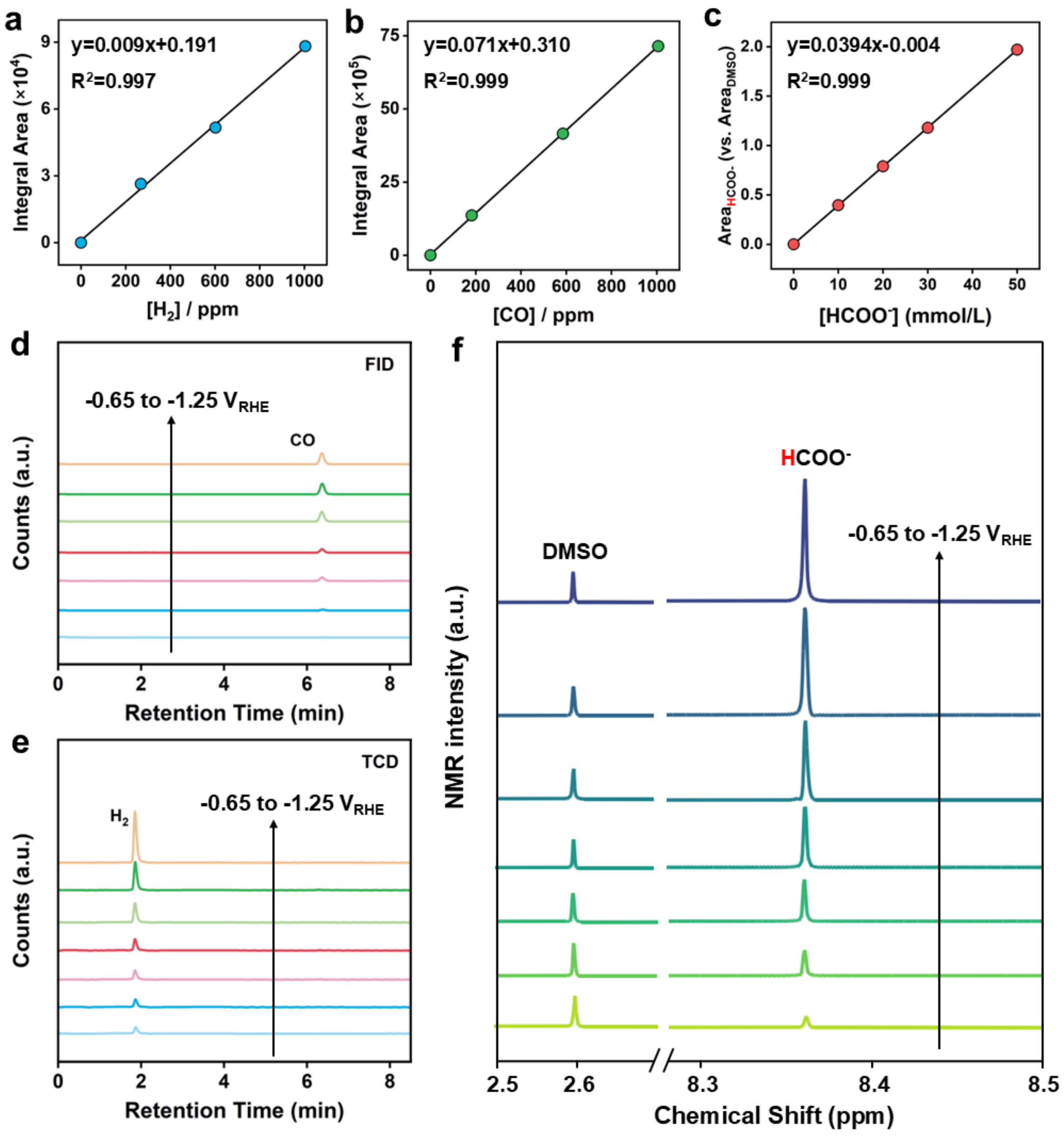

Supplementary Fig. 28 | Calibration curves for (a) CO, (b) H_2 and (c) HCOOH. Based on above calibration curves, the concentration of gas products produced was quantified accurately. Representative GC spectrum of the gas phase products (FID detector-based (d) and TCD detector-based (e)) and (f) ^{1}H NMR signals under different potentials on δ-Bi_2O_3/La_2O_3 catalyst within H-cell electrolyzer. DMSO (5 μL) is used as an internal standard for quantification of liquid products. Source data are provided as a Source Data file.

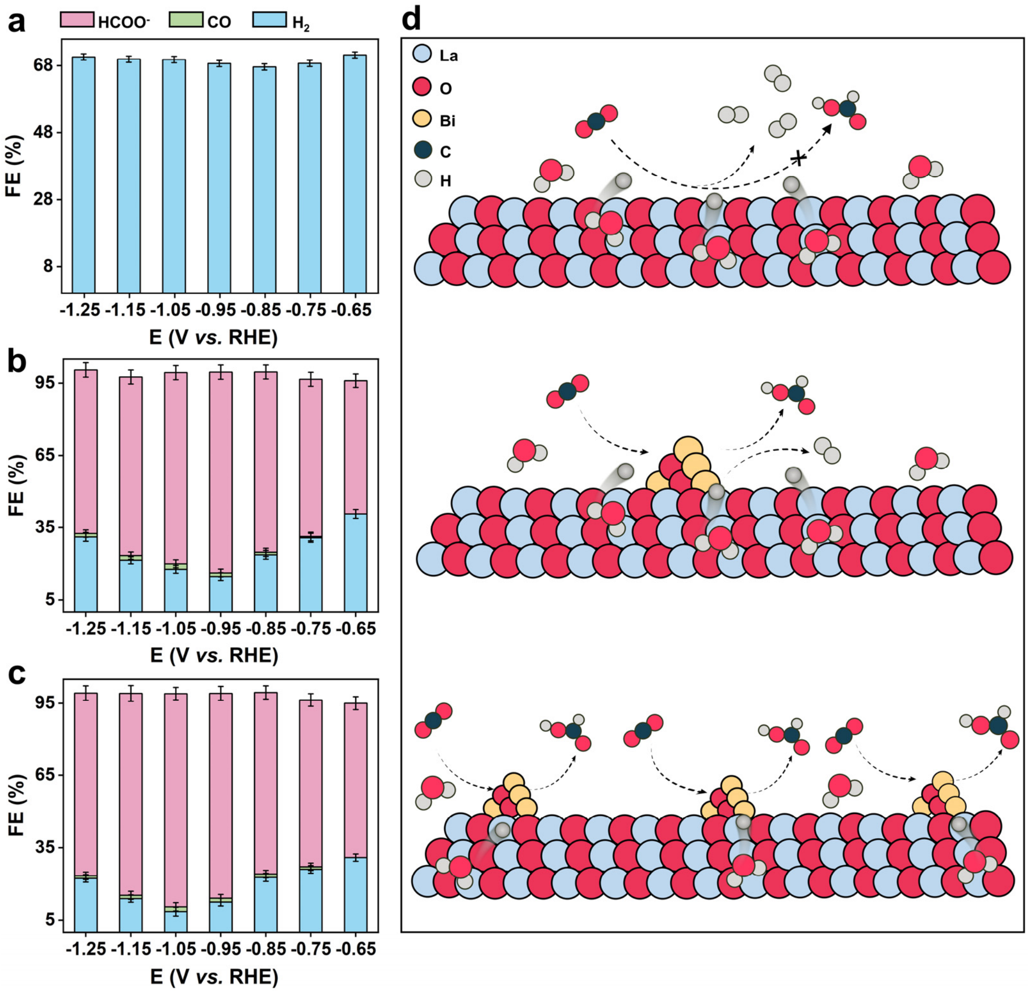

Supplementary Fig. 29 | Potential-dependent products distribution of CO_2RR over (a) pure La_2O_3, (b) 1% δ-Bi_2O_3/La_2O_3, (c) 3% δ-Bi_2O_3/La_2O_3. The error bars indicate the standard deviations in three independent measurements for each sample. (c) Schematic of the difference in CO_2RR activity with different δ-Bi_2O_3 loadings. CO_2-saturated 0.5 M $KHCO_3$ was used as the catholyte (pH = 7.22 ± 0.05) Catalyst mass loading was fixed at 1 mg cm^{-2}. Typical solution resistance in CO_2-saturated 0.5 M $KHCO_3$ is determined as 5.3 ± 0.2 Ω. Source data are provided as a Source Data file.

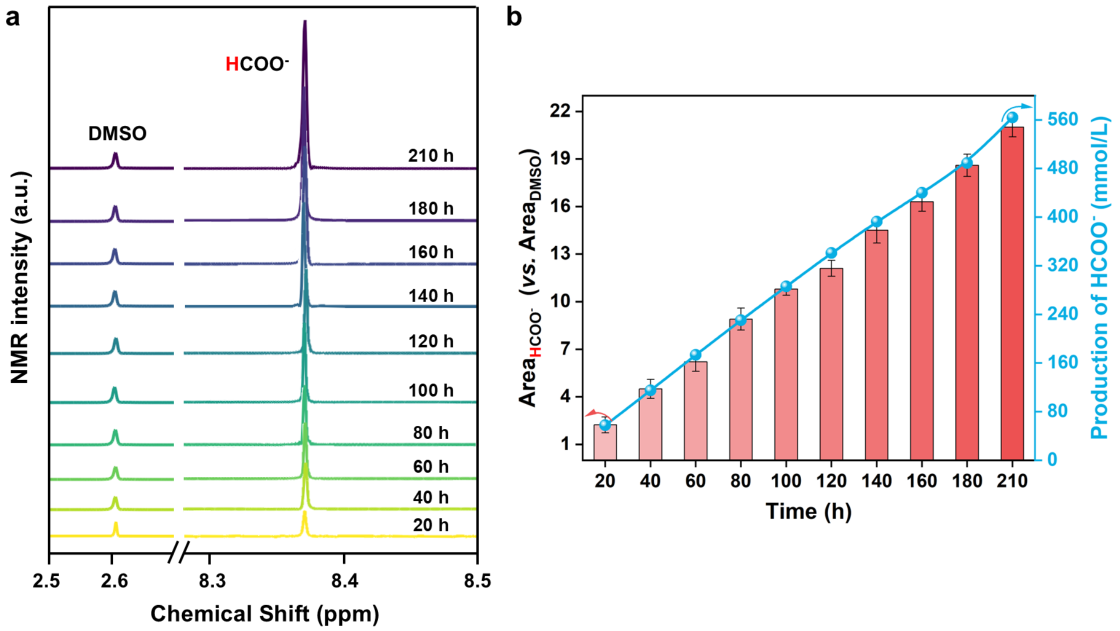

Supplementary Fig. 30 | (a) Representative ^{1}H NMR signals at different electrolysis times on δ-Bi_2O_3/La_2O_3 catalyst within H-cell electrolyzer. DMSO is used as an internal standard for the quantification of liquid products. (b) The integrated formate peak area and corresponding production based on the ^{1}H NMR data in Supplementary Fig. 30a. The error bars indicate the standard deviations in three independent measurements for each sample. Source data are provided as a Source Data file.

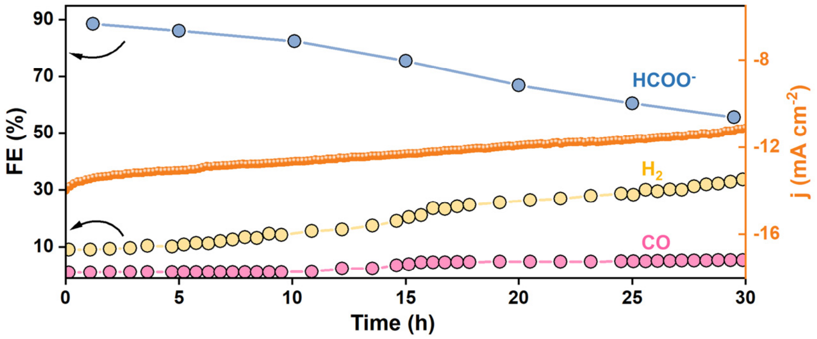

Supplementary Fig. 31 | Stability test of pure Bi_2O_3 at a fixed potential of −0.95 V vs. RHE within the H-cell. CO_2-saturated 0.5 M $KHCO_3$ was used as the catholyte (pH = 7.22 ± 0.05) Catalyst mass loading was fixed at 1 mg cm^{-2}. Typical solution resistance in CO_2-saturated 0.5 M $KHCO_3$ is determined as 5.3 ± 0.2 Ω. Source data are provided as a Source Data file.

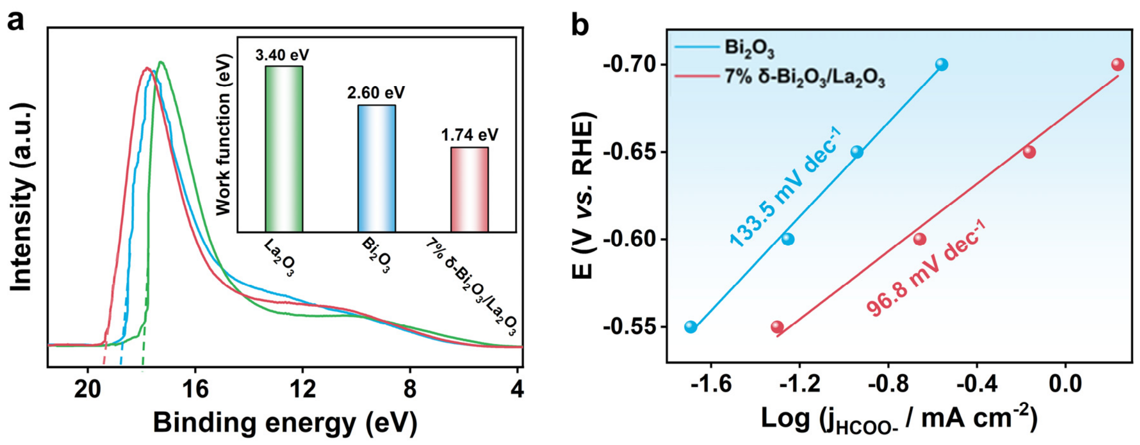

Supplementary Fig. 32 | (a) Ultraviolet photoelectron spectroscopy (UPS) measurements of the catalysts, the inset is the corresponding Work function (WF). (b) Tafel slope analyses. Source data are provided as a Source Data file.

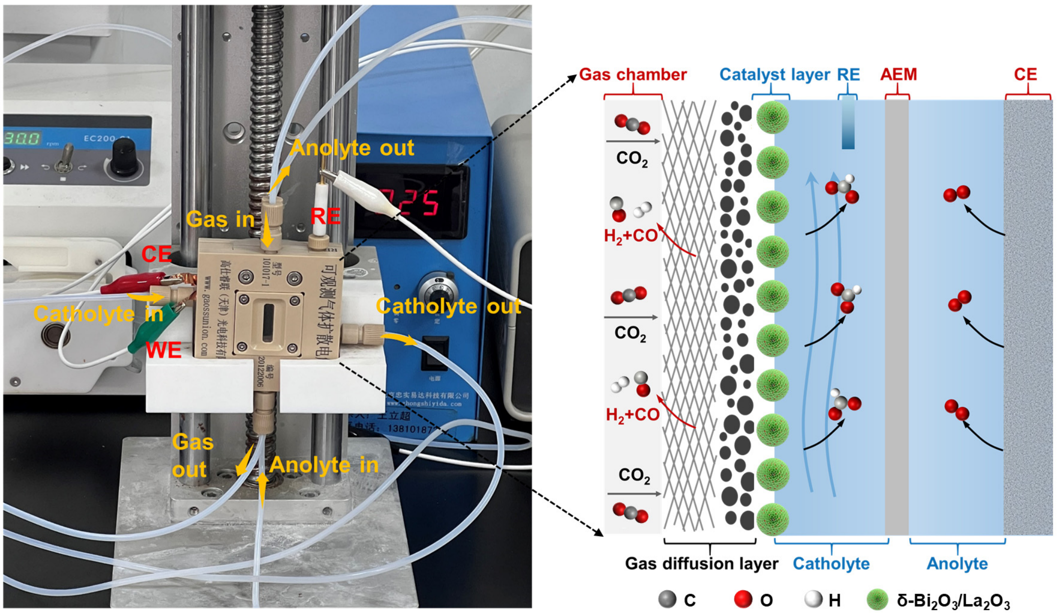

Supplementary Fig. 33 | Photograph and schematic illustration of the flow-cell configuration.

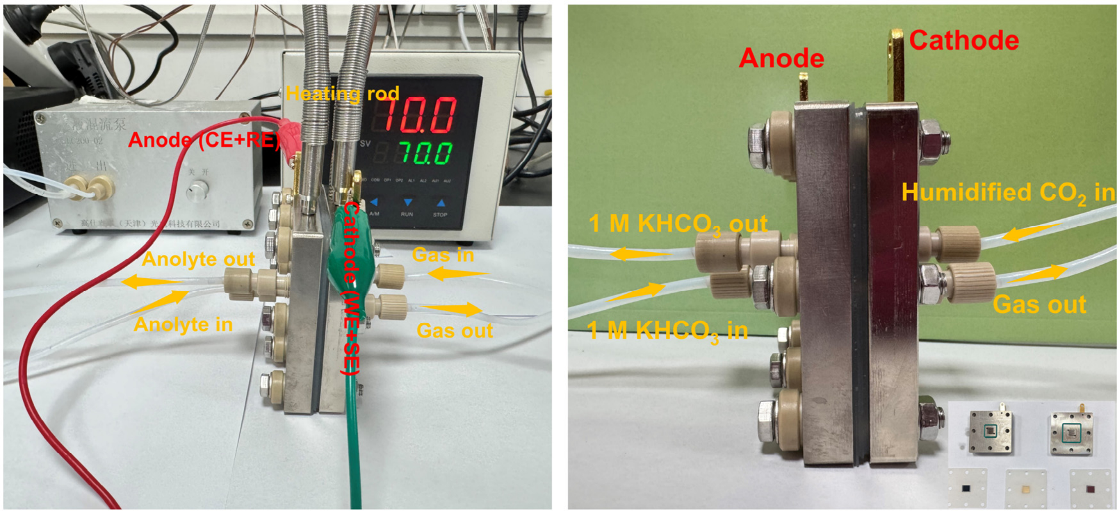

Supplementary Fig. 34 | Digital photo of the MEA electrolyzer. The inset shows an exploded-view photograph of the MEA device.

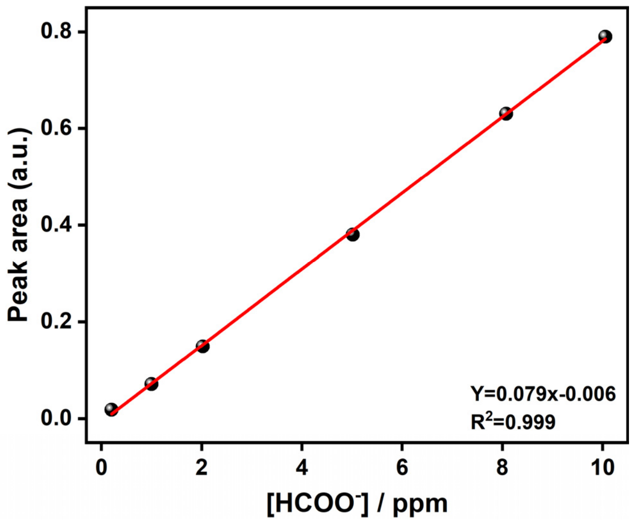

Supplementary Fig. 35 | HPLC calibration curves of the formate. Source data are provided as a Source Data file.

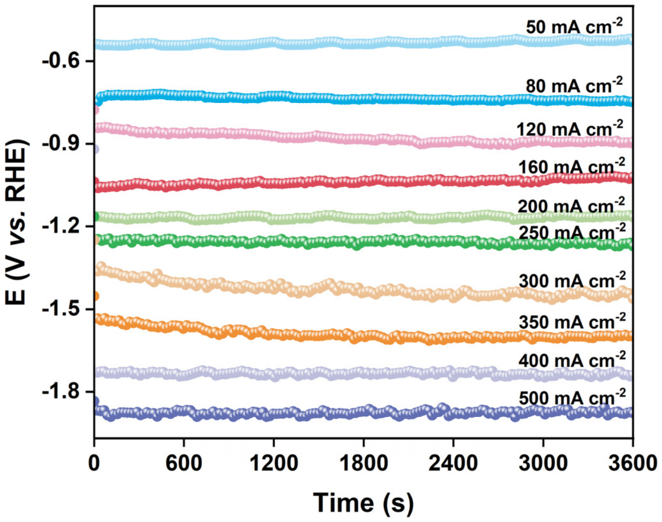

Supplementary Fig. 36 | Galvanostatic curves of 7% δ-Bi_2O_3/La_2O_3 in the flow-cell. CO_2-saturated 0.5 M $KHCO_3$ was used as the catholyte (pH = 7.22 ± 0.05) Catalyst mass loading was fixed at 1 mg cm^{-2}. Typical solution resistance in CO_2-saturated 0.5 M $KHCO_3$ is determined as 1.1 ± 0.4 Ω. Source data are provided as a Source Data file.

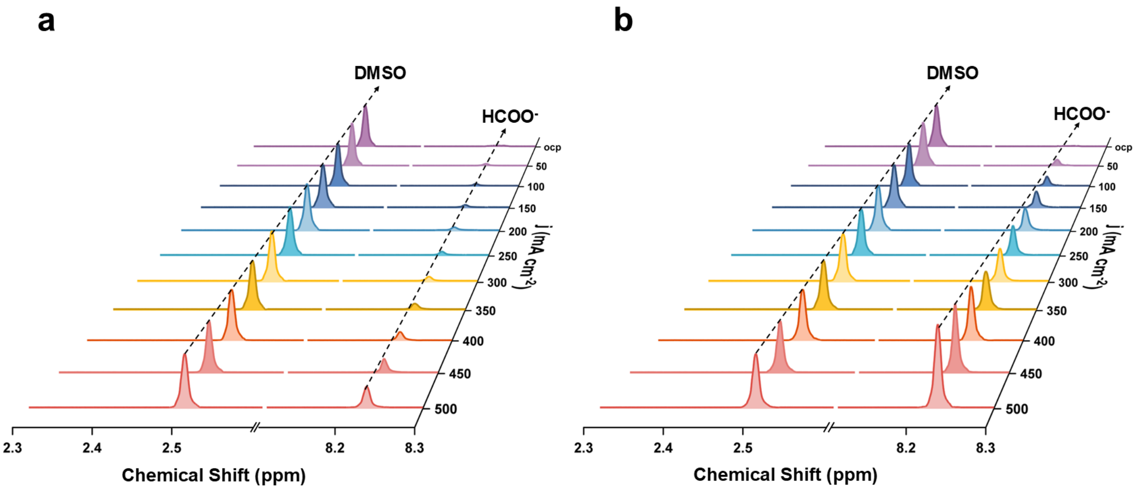

Supplementary Fig. 37 | ^{1}H NMR spectra of the anolyte for formate analysis. Representative signals at different current densities on 7% δ-Bi_2O_3/La_2O_3 within (a) Flow-cell and (b) MEA electrolyzer. Source data are provided as a Source Data file.

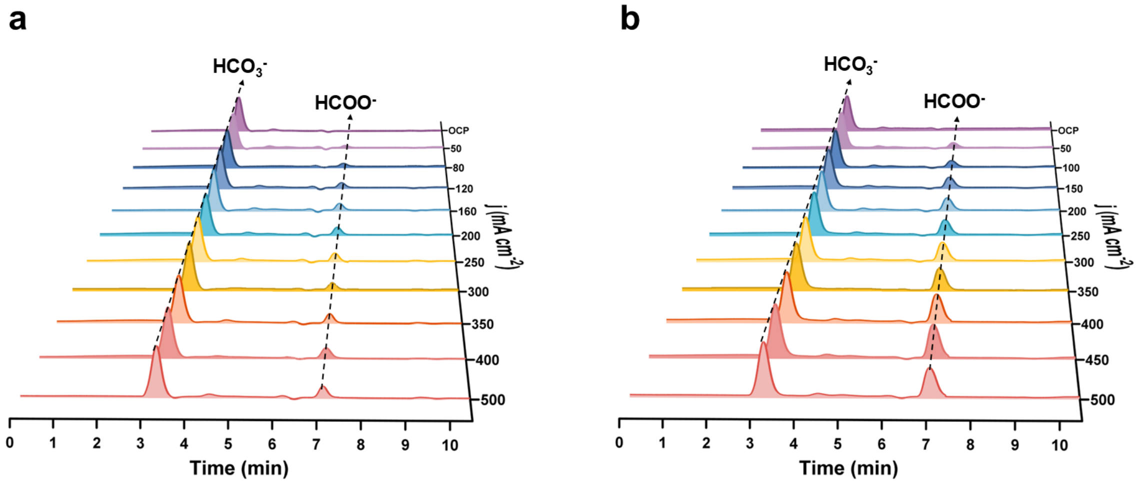

Supplementary Fig. 38 | HPLC chromatograms of the anolyte for formate analysis. Representative signals at different current densities on 7% δ-Bi_2O_3/La_2O_3 within (a) Flow-cell and (b) MEA electrolyzer. Source data are provided as a Source Data file.

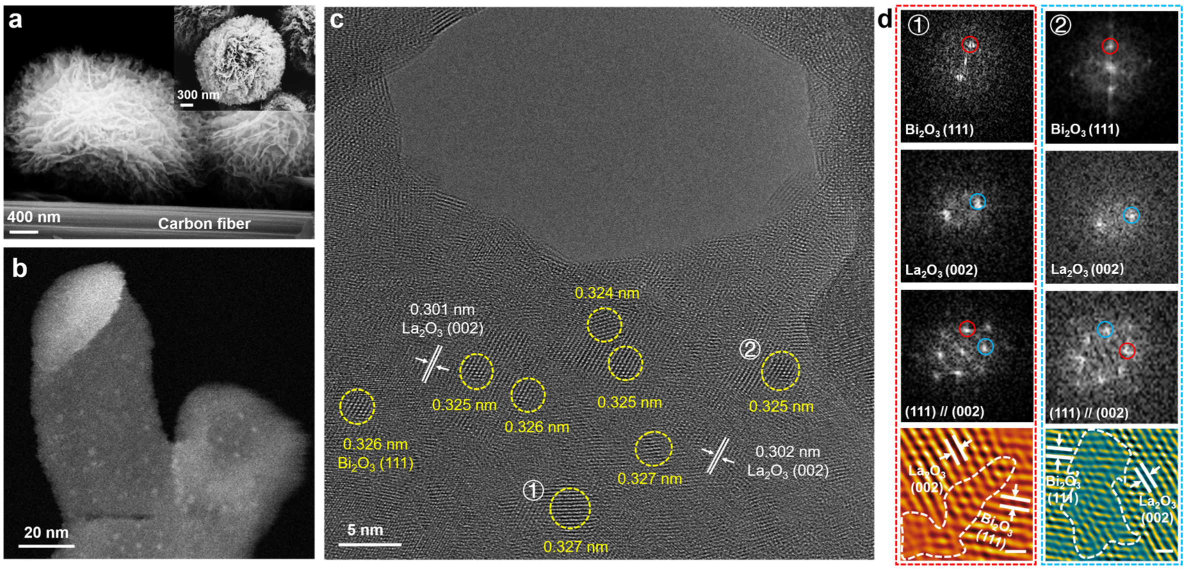

Supplementary Fig. 39 | Structural characterization for the 7% δ-Bi_2O_3/La_2O_3 after stability test. (a) The cross sectional SEM images of the cathode GDE, the inset is top-view SEM image. (b) Aberration-corrected HAADF-STEM image. (c) HRTEM image and (d) corresponding FFT analysis of the selected region. The scale bar in (d) is 1 nm.

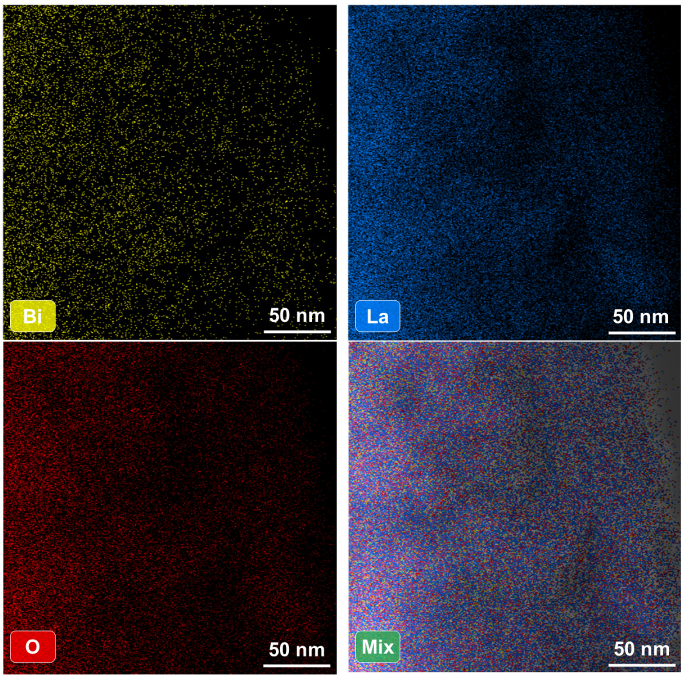

Supplementary Fig. 40 | EDS mapping of 7% δ-Bi_2O_3/La_2O_3 after stability test in the flow-cell configuration.

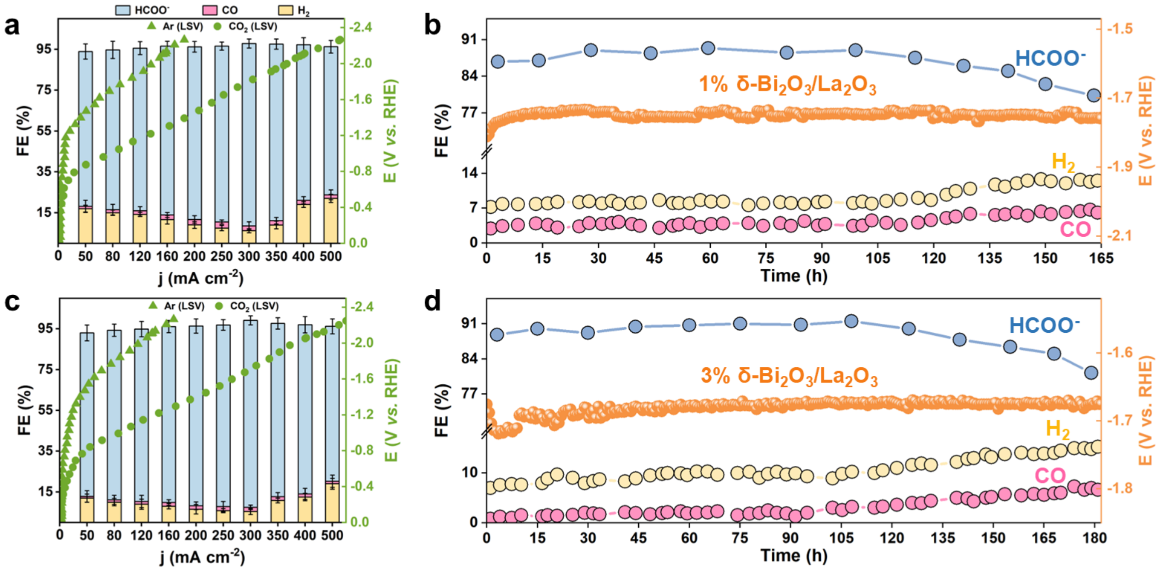

Supplementary Fig. 41 | LSV curves and corresponding FEs of various products as a function of current density for (a) 1% δ-Bi_2O_3/La_2O_3 and (c) 3% δ-Bi_2O_3/La_2O_3 catalysts. Long-term chronopotentiometric curves and corresponding FEs of (b) 1% δ-Bi_2O_3/La_2O_3 and (d) 3% δ-Bi_2O_3/La_2O_3 at 350 mA cm^{-2} in a flow cell. The error bars represent standard deviations from three independent measurements. CO_2-saturated 0.5 M $KHCO_3$ was used as the catholyte (pH = 7.22 ± 0.05). Catalyst mass loading was fixed at 1 mg cm^{-2}. Typical solution resistance in CO_2-saturated 0.5 M $KHCO_3$ is determined as 1.1 ± 0.4 Ω. Source data are provided as a Source Data file.

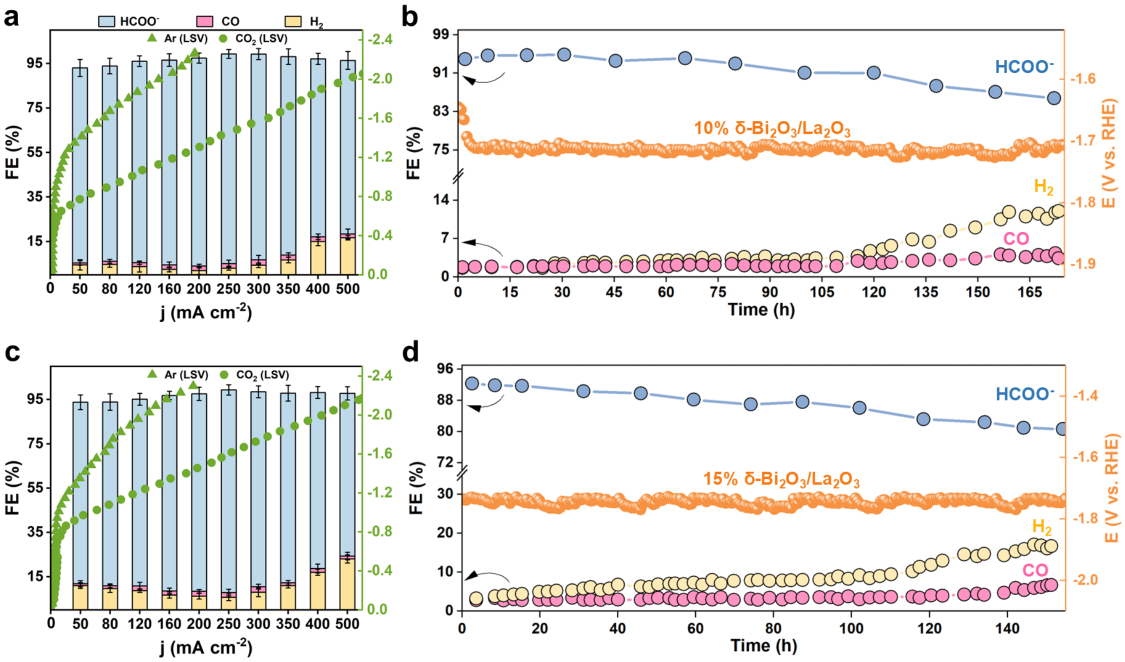

Supplementary Fig. 42 | LSV curves and corresponding FEs of various products as a function of current density for (a) 10% δ-Bi_2O_3/La_2O_3 and (c) 15% δ-Bi_2O_3/La_2O_3 catalysts. Long-term chronopotentiometric curves and corresponding FEs of (b) 10% δ-Bi_2O_3/La_2O_3 and (d) 15% δ-Bi_2O_3/La_2O_3 at 350 mA cm^{-2} in a flow cell. The error bars represent standard deviations from three independent measurements. CO_2-saturated 0.5 M $KHCO_3$ was used as the catholyte (pH = 7.22 ± 0.05) Catalyst mass loading was fixed at 1 mg cm^{-2}. Typical solution resistance in CO_2-saturated 0.5 M $KHCO_3$ is determined as 1.1 ± 0.4 Ω. Source data are provided as a Source Data file.

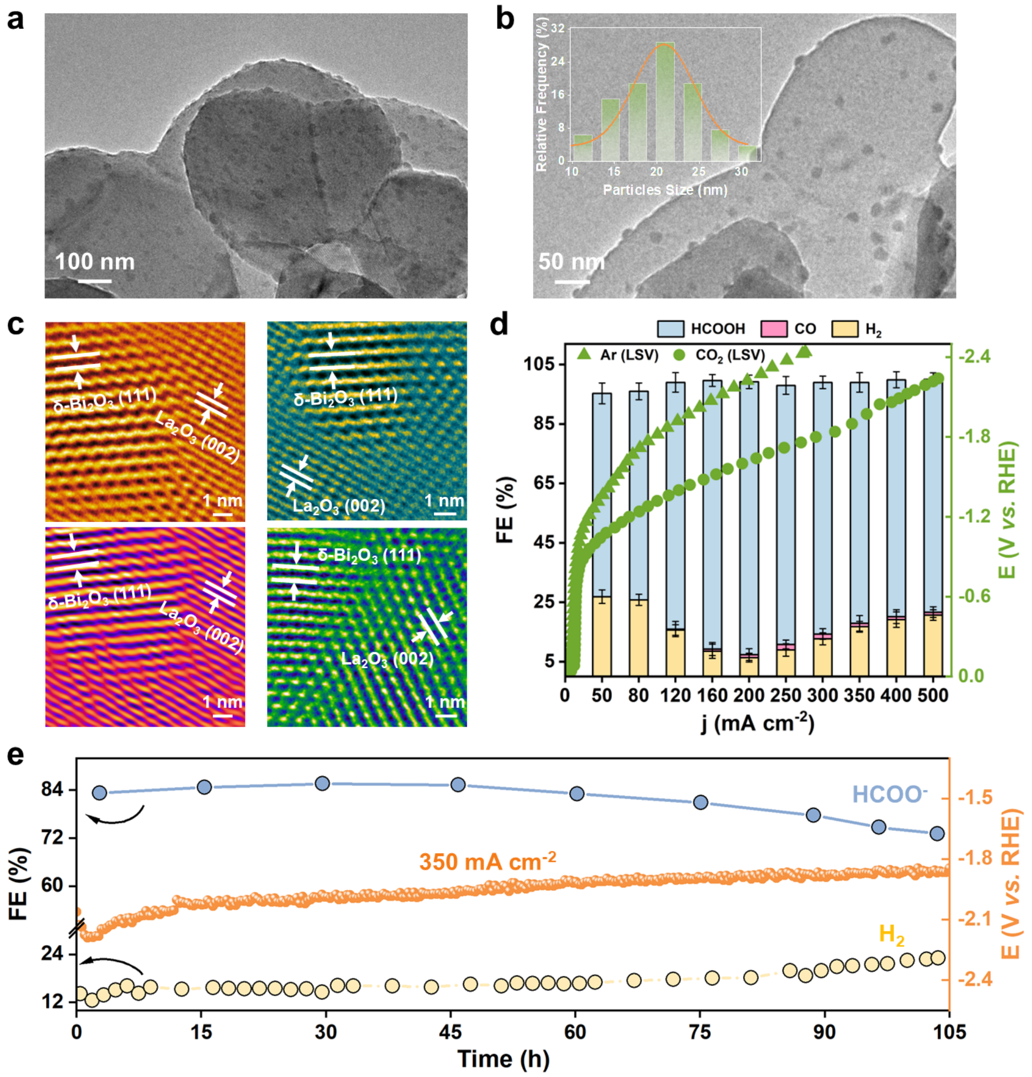

Supplementary Fig. 43 | La_2O_3-socketed 20 nm δ-Bi_2O_3. (a, b) Typical TEM images and relevant size distribution. The inset is corresponding size distribution. (c) HRTEM images in different region. (d) LSV and FEs of various products at different current densities. Data were recorded using flow cells with 0.5 M $KHCO_3$. (e) Durability test and corresponding FE_{HCOO^-} of continuous electrolysis under a constant total current density of 350 mA cm^{-2}. The error bars represent standard deviations from three independent measurements. CO_2-saturated 0.5 M $KHCO_3$ was used as the catholyte (pH = 7.22 ± 0.05) Catalyst mass loading was fixed at 1 mg cm^{-2}. Typical solution resistance in CO_2-saturated 0.5 M $KHCO_3$ is determined as 1.1 ± 0.4 Ω. Source data are provided as a Source Data file.

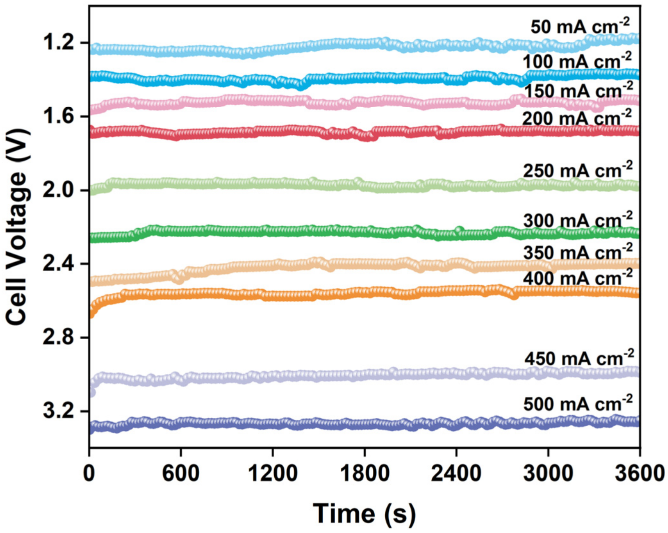

Supplementary Fig. 44 | Galvanostatic curves of 7% δ-Bi_2O_3/La_2O_3 in the MEA electrolyzer. Humified CO_2 flowed through the cathode side and 1.0 M $KHCO_3$ as the anolyte (pH=8.3 ± 0.06). Catalyst mass loading was fixed at 1 mg cm^{-2}. Source data are provided as a Source Data file.

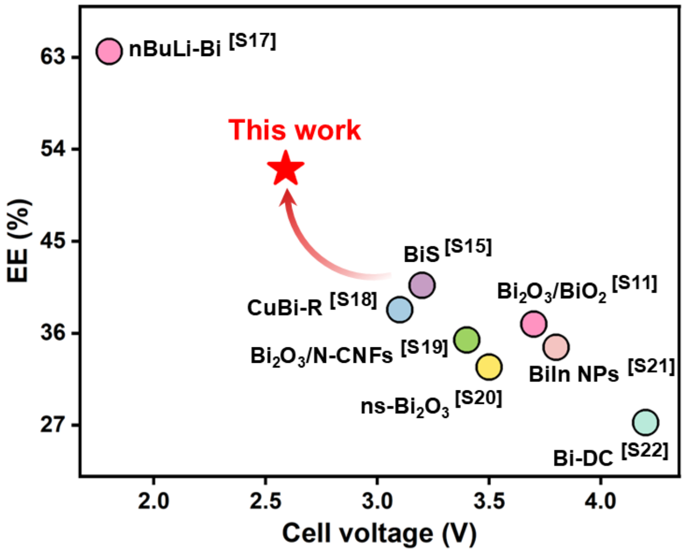

Supplementary Fig. 45 | Comparison of $EE_{formate}$ for 7% δ-Bi_2O_3/La_2O_3 and recently reported Bi-based electrocatalysts. Relevant references are listed in Table S4.

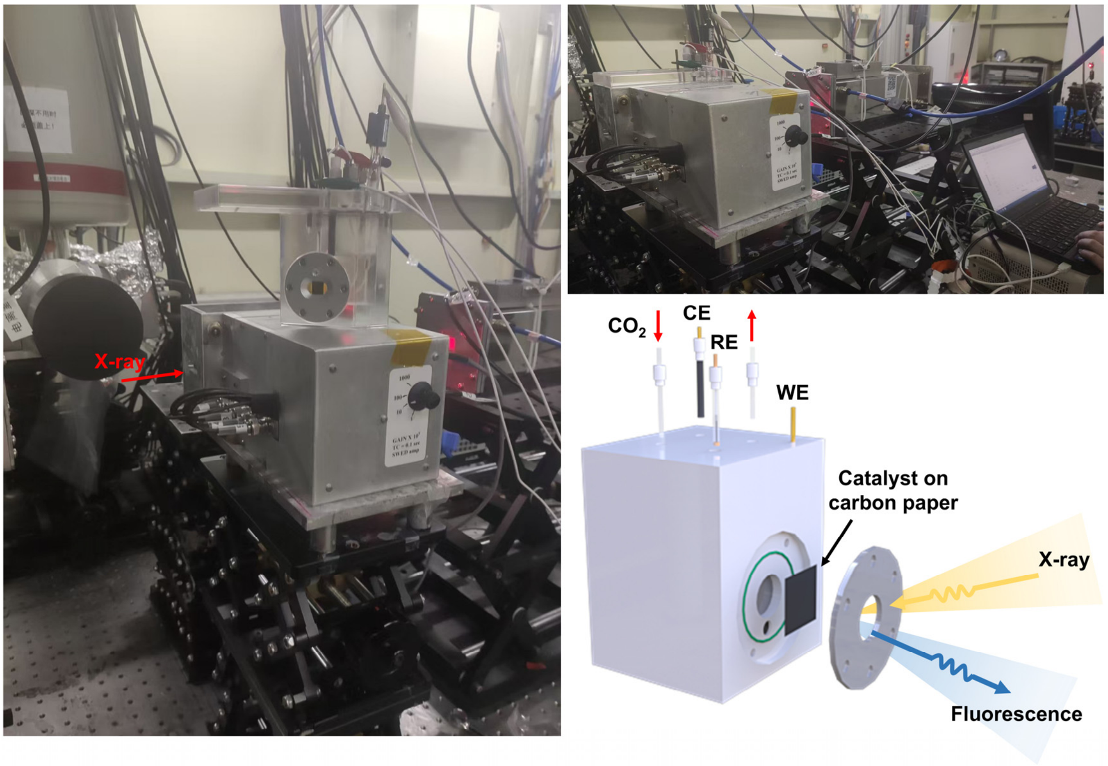

Supplementary Fig. 46 | Photograph and schematics of the electrolysis cell for in situ XAS experiments.

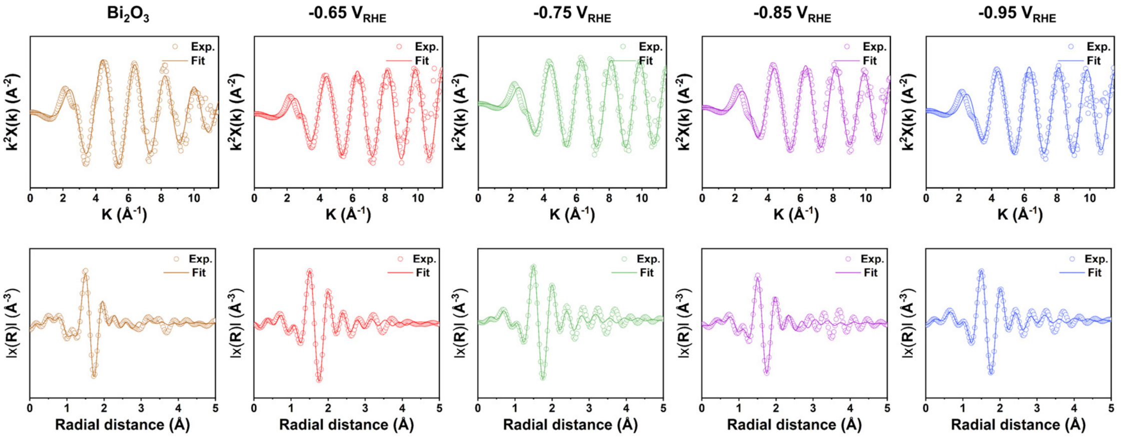

Supplementary Fig. 47 | k^2-weighted Bi L_3-edge EXAFS oscillations (up) and their associated Fourier transforms (down) for Bi_2O_3 under different applied potential; Experimental data, dotted lines; Fitting curves, solid lines. Typical solution resistance in CO_2-saturated 0.5 M $KHCO_3$ was determined to be 2.3 ± 0.2 Ω for the XAS electrolysis cell. Source data are provided as a Source Data file.

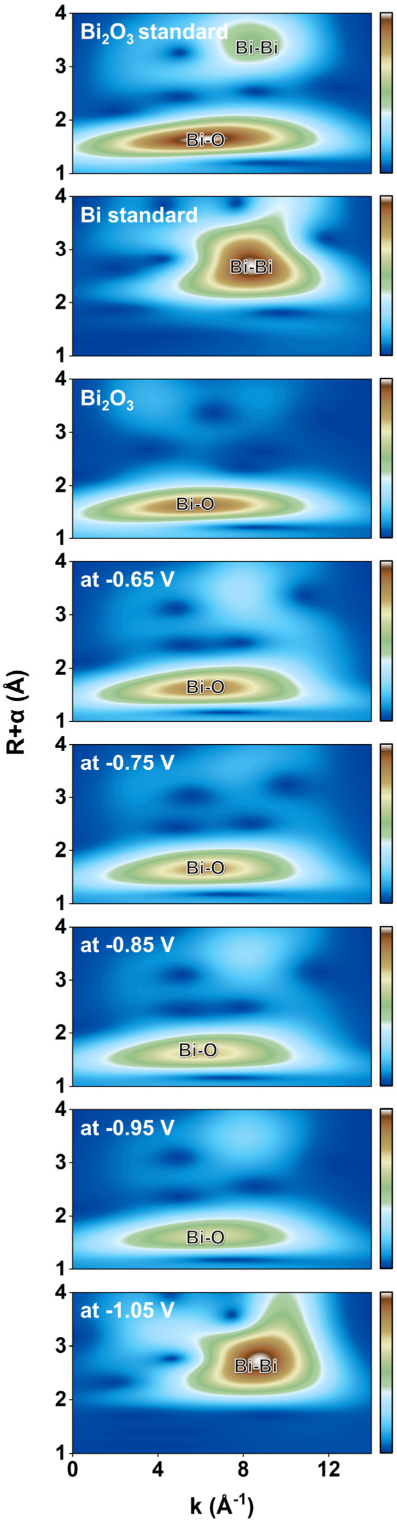

Supplementary Fig. 48 | Wavelet transform extended X-ray absorption fine structure (WT-EXAFS) map of pure Bi_2O_3 during CO_2RR process. Source data are provided as a Source Data file.

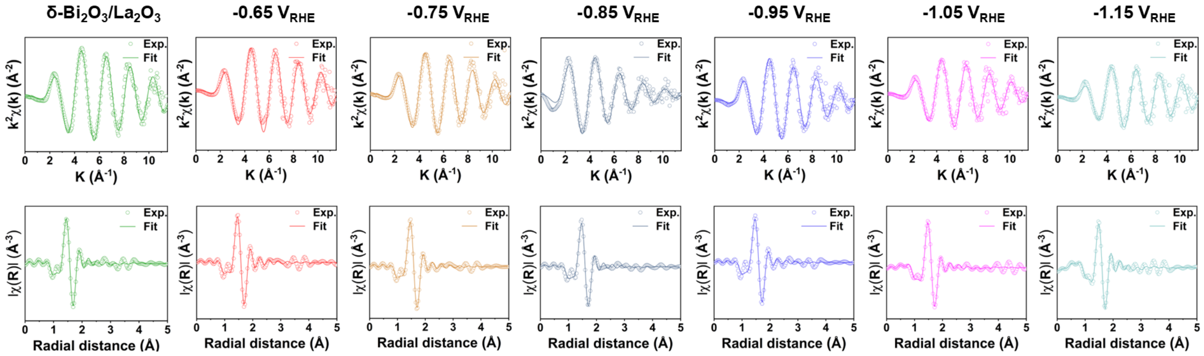

Supplementary Fig. 49 | k^2-weighted Bi L_3-edge EXAFS oscillations (up) and their associated Fourier transforms (down) for the 7% δ-Bi_2O_3/La_2O_3 catalyst under different applied potential; Experimental data, dotted lines; Fitting curves, solid lines. Typical solution resistance in CO_2-saturated 0.5 M $KHCO_3$ was determined to be 2.3 ± 0.2 Ω for the XAS electrolysis cell. Source data are provided as a Source Data file.

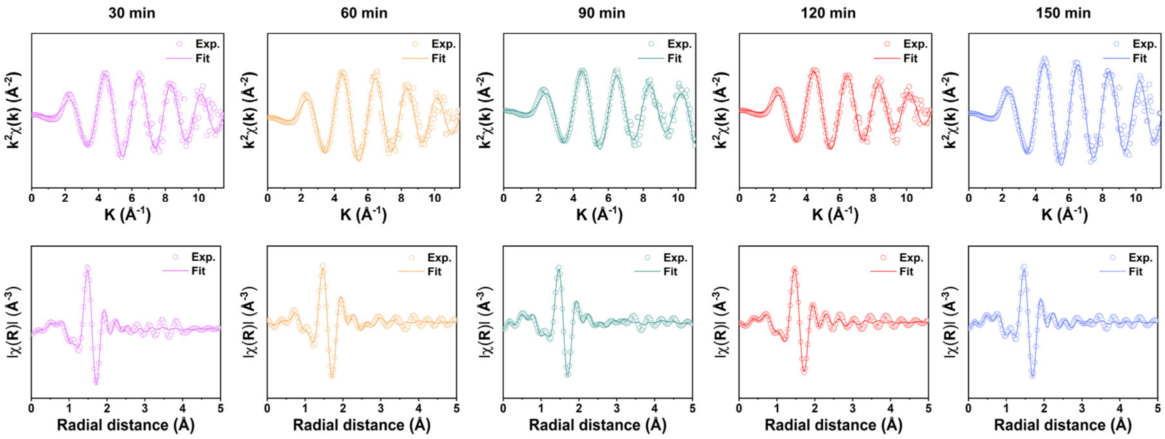

Supplementary Fig. 50 | k^2-weighted Bi L_3-edge EXAFS oscillations (up) and their associated Fourier transforms (down) for 7% δ-Bi_2O_3/La_2O_3 during 150 min of reaction at −1.15 V vs. RHE; Experimental data, dotted lines; Fitting curves, solid lines. Typical solution resistance in CO_2-saturated 0.5 M $KHCO_3$ was determined to be 2.3 ± 0.2 Ω for the XAS electrolysis cell. Source data are provided as a Source Data file.

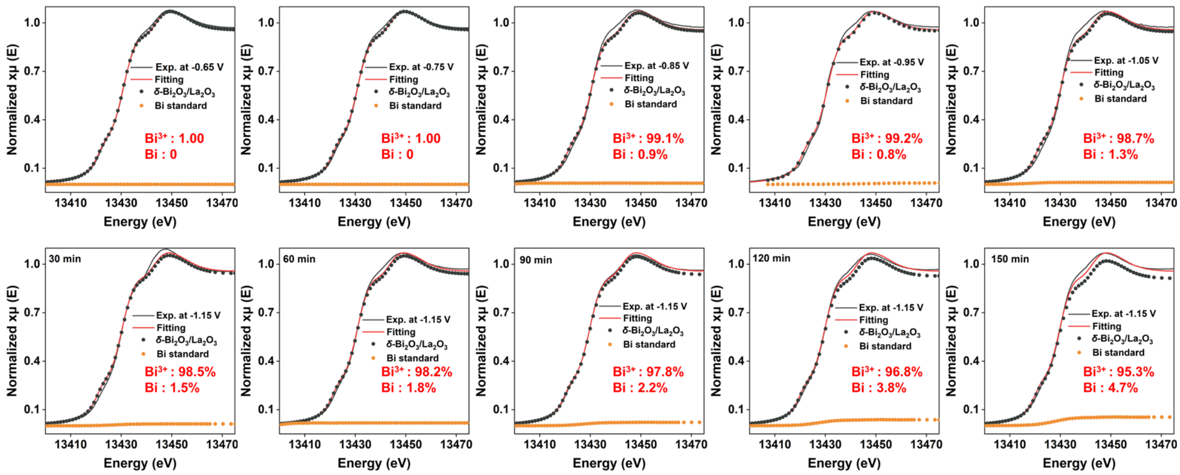

Supplementary Fig. 51 | XANES spectrum and linear combination fitting results of 7% δ-Bi_2O_3/La_2O_3. Source data are provided as a Source Data file.

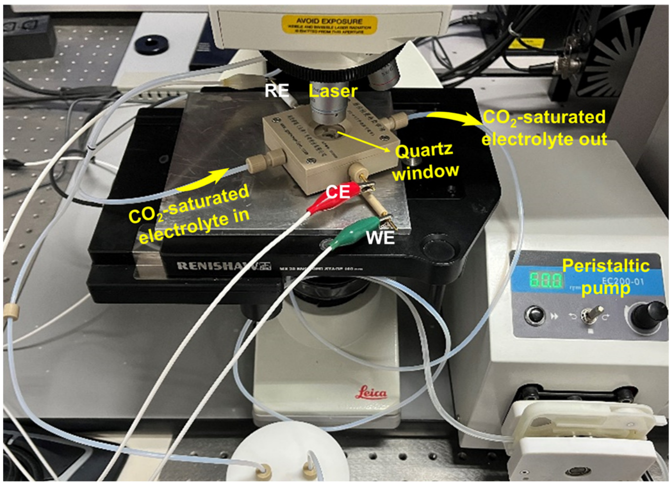

Supplementary Fig. 52 | Digital photo of the electrochemical in situ Raman measurement.

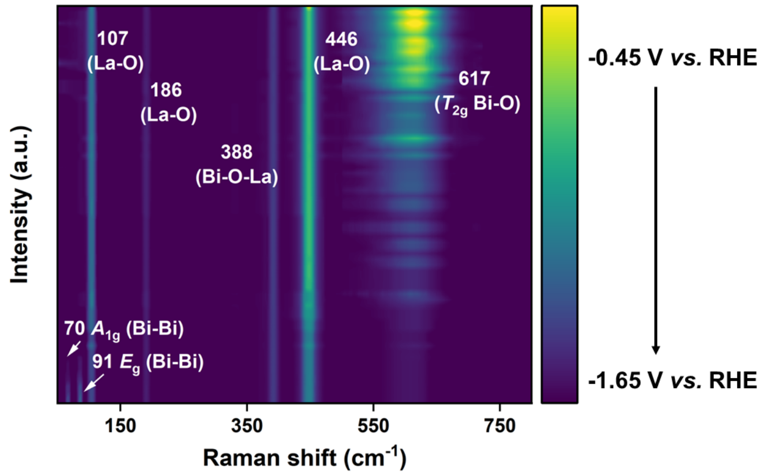

Supplementary Fig. 53 | In situ Raman spectra monitoring the surface variation of 10% δ-Bi_2O_3/La_2O_3 under different applied potentials. CO_2-saturated 0.5 M $KHCO_3$ was used as the electrolyte (pH = 7.22 ± 0.05). Typical solution resistance in CO_2-saturated 0.5 M $KHCO_3$ was determined to be 1.6±0.3 Ω for the Raman cell. Source data are provided as a Source Data file.

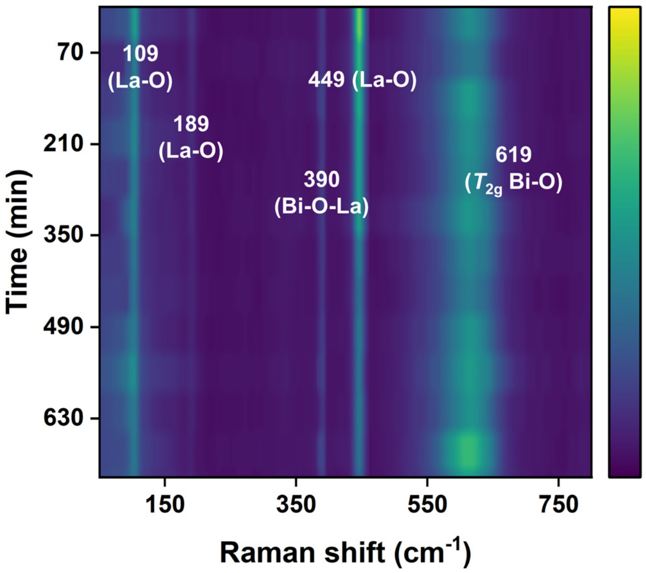

Supplementary Fig. 54 | Time-dependent counter map of in situ Raman spectra monitoring the structural variation for 7% δ-Bi_2O_3/La_2O_3 electrode at −0.95 V vs. RHE. CO_2-saturated 0.5 M $KHCO_3$ was used as the electrolyte (pH = 7.22 ± 0.05). Typical solution resistance in CO_2-saturated 0.5 M $KHCO_3$ was determined to be 1.6±0.3 Ω for the Raman cell. Source data are provided as a Source Data file.

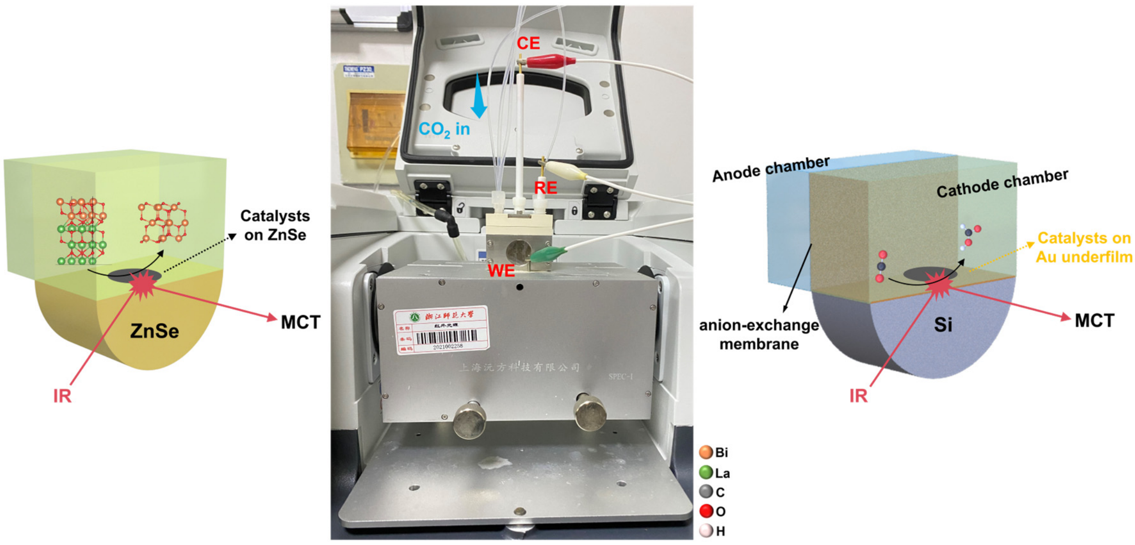

Supplementary Fig. 55 | Photograph and schematic diagram of the in situ electrochemical FT-IR and ATR-SEIRAS measurements.

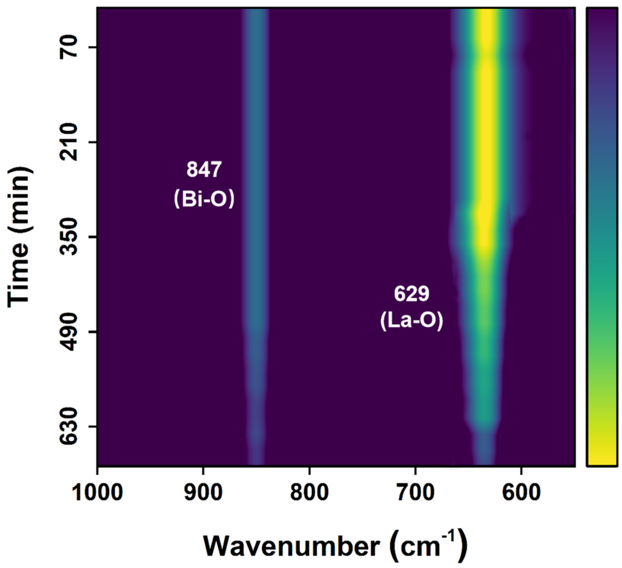

Supplementary Fig. 56 | Time dependent counter map of in situ FT-IR spectra monitoring structural variation for 7% δ-Bi_2O_3/La_2O_3 electrode. CO_2-saturated 0.5 M $KHCO_3$ was used as the electrolyte (pH = 7.22 ± 0.05). Source data are provided as a Source Data file.

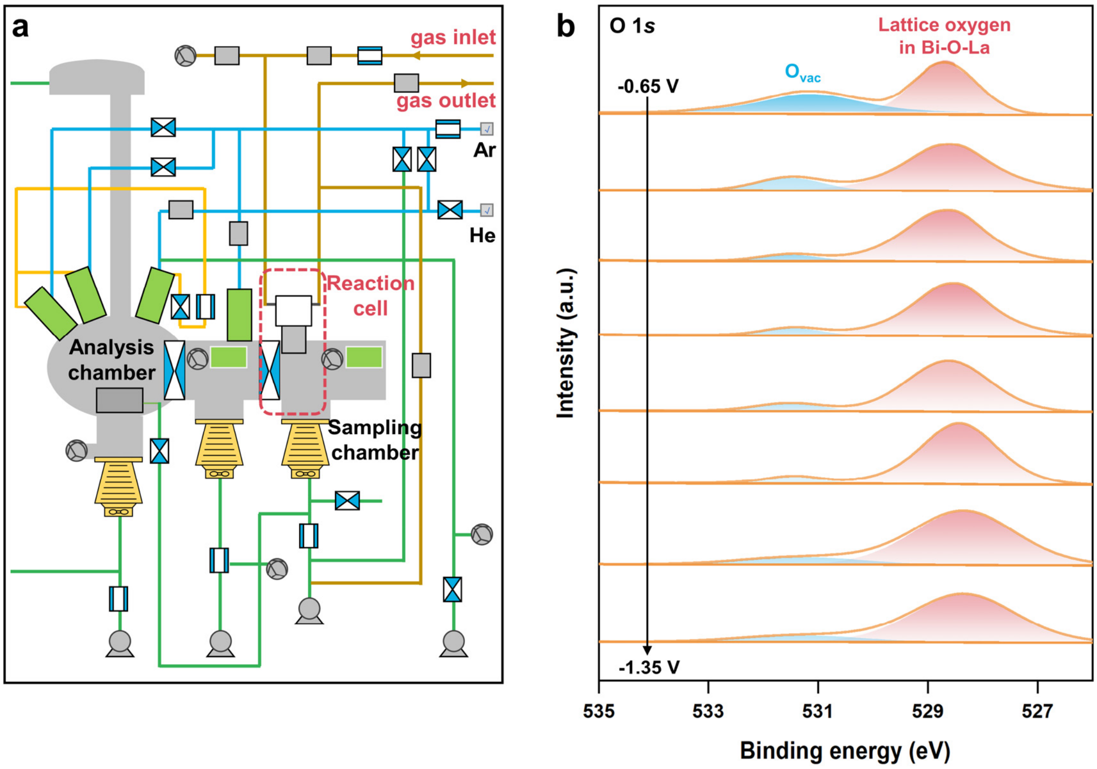

Supplementary Fig. 57 | (a) Structural schematic of quasi-in situ XPS system. (b) Potential-dependent quasi-in situ XPS tests for δ-Bi_2O_3/La_2O_3 electrode under electrolysis from −0.65 to −1.35 V vs. RHE. Source data are provided as a Source Data file.

394

395 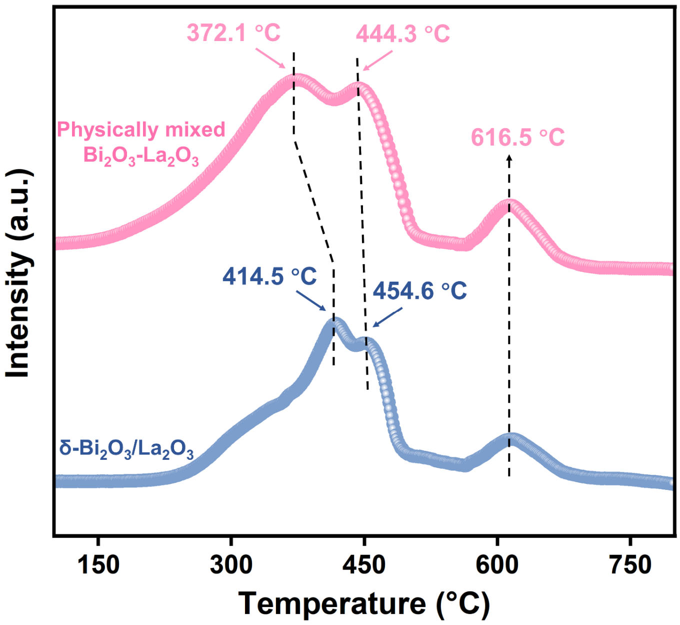

396 **Supplementary Fig. 58 |** H_2-TPR profiles of the 7% δ-Bi_2O_3/La_2O_3 and a physically mixed

397 Bi_2O_3-La_2O_3 sample. Source data are provided as a Source Data file.

398

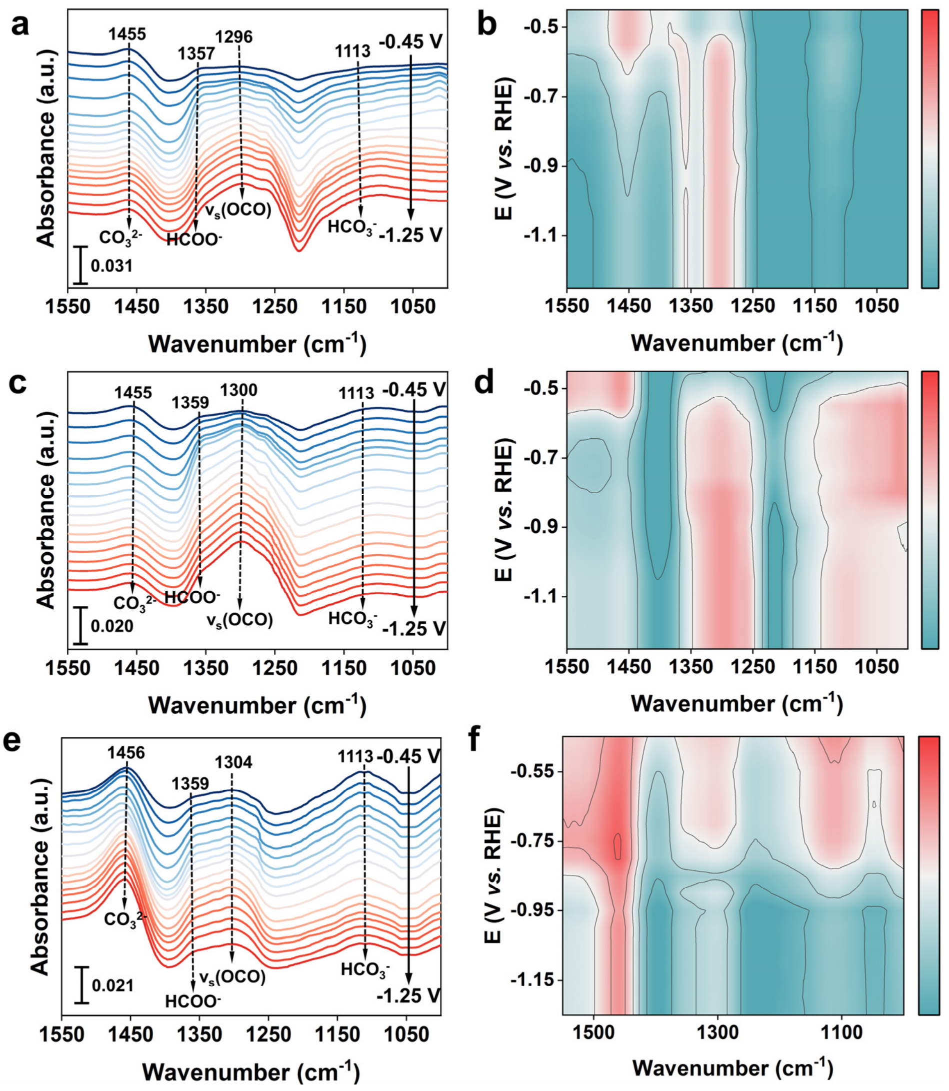

Supplementary Fig. 59 | Potentiodynamic ATR-SEIRAS and corresponding color contour map of (a, b) 1% δ-Bi_2O_3/La_2O_3, (c, d) 3% δ-Bi_2O_3/La_2O_3, (e, f) pure Bi_2O_3 electrode in CO_2-saturated 0.5 M $KHCO_3$ solution (pH = 7.22 ± 0.05). Typical solution resistance in CO_2-saturated 0.5 M $KHCO_3$ is determined as 4.1 ± 1.2 Ω for ATR-SEIRA cell. Source data are provided as a Source Data file.

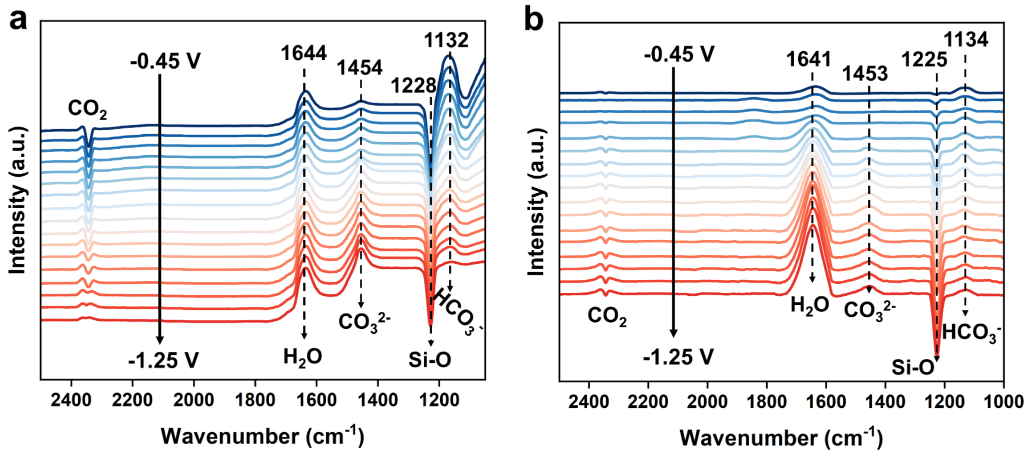

Supplementary Fig. 60 | In situ ATR-SEIRAS of control samples. (a) Bare Au-coated IRE and (b) pure La_2O_3 on Au underfilm. Spectra were collected at different applied potentials in CO_2-saturated 0.5 M $KHCO_3$ solution (pH = 7.22 ± 0.05), using a reference spectrum at −0.25 V vs. RHE. Typical solution resistance in CO_2-saturated 0.5 M $KHCO_3$ is determined as 4.1 ± 1.2 Ω for ATR-SEIRA cell. Source data are provided as a Source Data file.

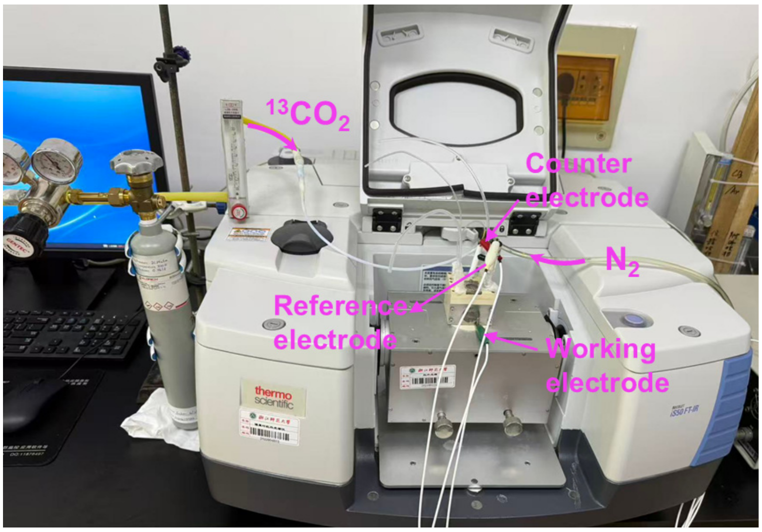

Supplementary Fig. 61 | Photograph of the electrochemical ATR-SEIRAS cell configured for $^{13}CO_2$ isotope labeling.

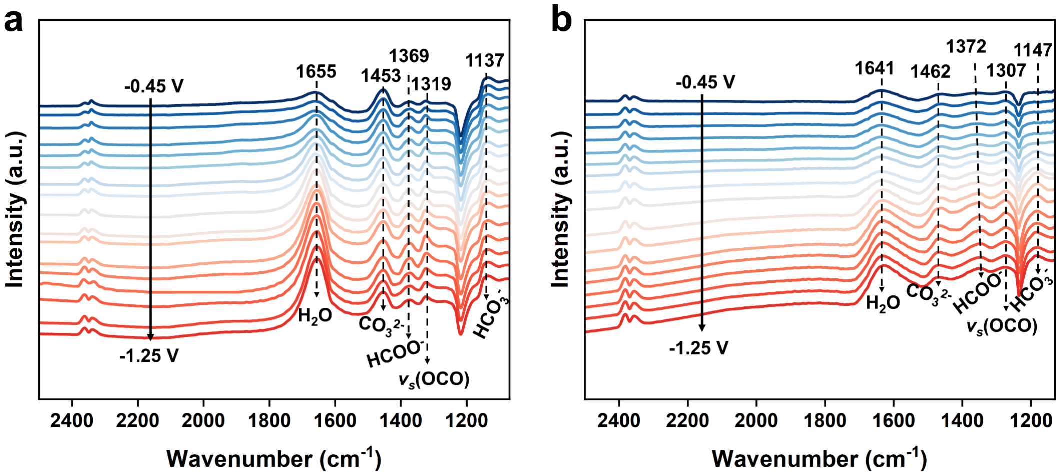

Supplementary Fig. 62 | In situ ATR-SEIRA spectra of (a) 10% and (b) 15% δ-Bi_2O_3/La_2O_3 recorded at different applied potentials in CO_2-saturated 0.5 M $KHCO_3$ solution (pH = 7.22 ± 0.05), with the spectrum at −0.25 V vs. RHE as the reference. Typical solution resistance in CO_2-saturated 0.5 M $KHCO_3$ is determined as 4.1 ± 1.2 Ω for ATR-SEIRA cell. Source data are provided as a Source Data file.

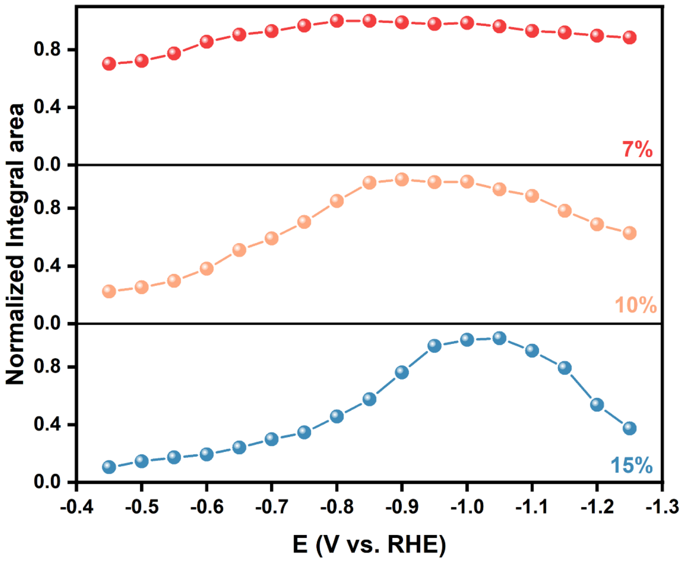

Supplementary Fig. 63 | Potential-dependent integrated intensities of the ν_s(OCO) band for the 7%, 10%, and 15% δ-Bi_2O_3/La_2O_3 samples. Source data are provided as a Source Data file.

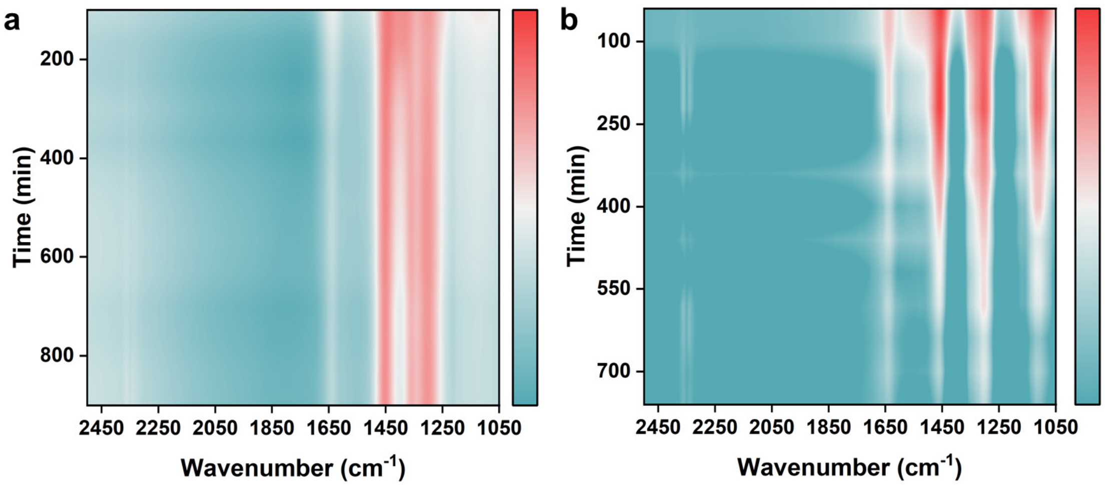

Supplementary Fig. 64 | Time-evolved ATR-SEIRAS color contour map for (a) 7% δ-Bi_2O_3/La_2O_3 and (b) pure Bi_2O_3 samples. CO_2-saturated 0.5 M $KHCO_3$ was used as the electrolyte (pH = 7.22 ± 0.05). Typical solution resistance in CO_2-saturated 0.5 M $KHCO_3$ is determined as 4.1 ± 1.2 Ω for ATR-SEIRA cell. Source data are provided as a Source Data file.

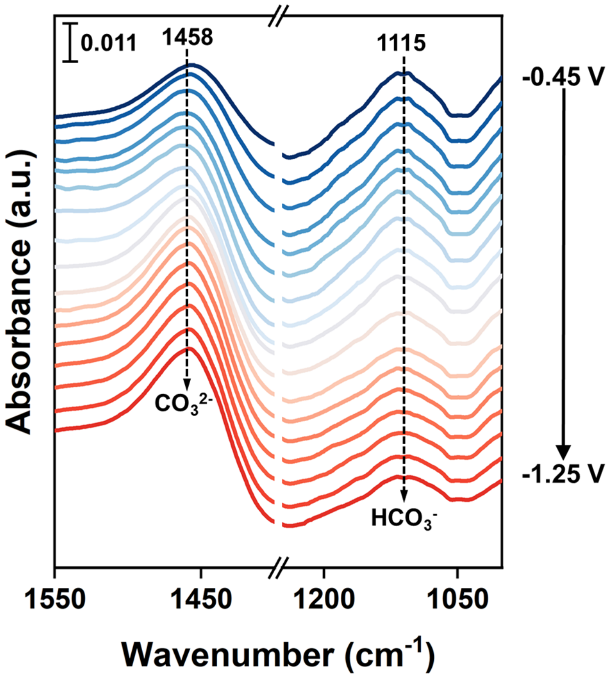

Supplementary Fig. 65 | Potentiodynamic ATR-SEIRA spectra enlarged in the HCO_3^- and CO_3^{2-} region of pure Bi_2O_3. CO_2-saturated 0.5 M $KHCO_3$ was used as the electrolyte (pH = 7.22 ± 0.05). Typical solution resistance in CO_2-saturated 0.5 M $KHCO_3$ is determined as 4.1 ± 1.2 Ω for ATR-SEIRA cell. Source data are provided as a Source Data file.

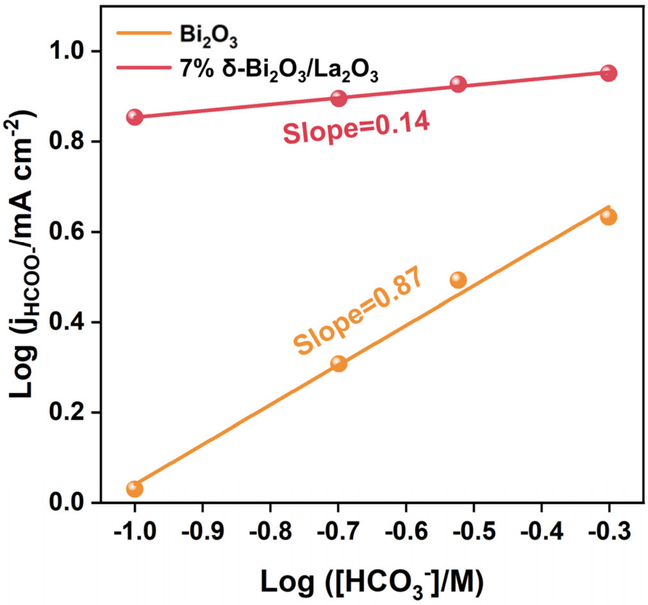

Supplementary Fig. 66 | Logarithm of j_{HCOO^-} *versus* logarithm of [HCO_3^-] for 7% δ-Bi_2O_3/La_2O_3 and pure Bi_2O_3 samples at −0.95 V vs. RHE. Source data are provided as a Source Data file.

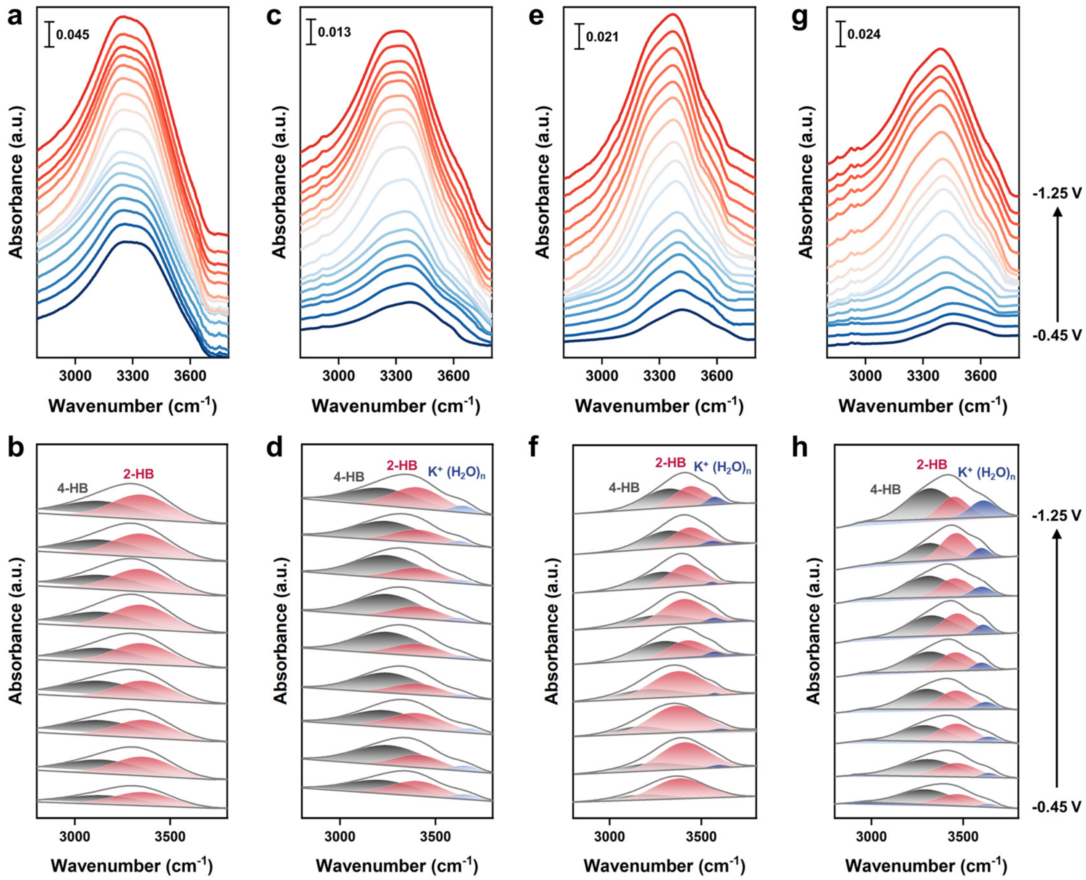

Supplementary Fig. 67 | The water stretching peak in the ATR-SEIRAS and corresponding deconvolution of the O−H stretching vibration band over (a, b) Bi_2O_3, 1% δ-Bi_2O_3/La_2O_3, 3% δ-Bi_2O_3/La_2O_3 and 7% δ-Bi_2O_3/La_2O_3 catalysts in 0.5 M $KHCO_3$ solution (pH = 7.22 ± 0.05) saturated with CO_2 gas, using a single beam spectrum at −0.25 V vs. RHE as a reference Typical solution resistance in CO_2-saturated 0.5 M $KHCO_3$ is determined as 4.1 ± 1.2 Ω for ATR-SEIRA cell. Source data are provided as a Source Data file.

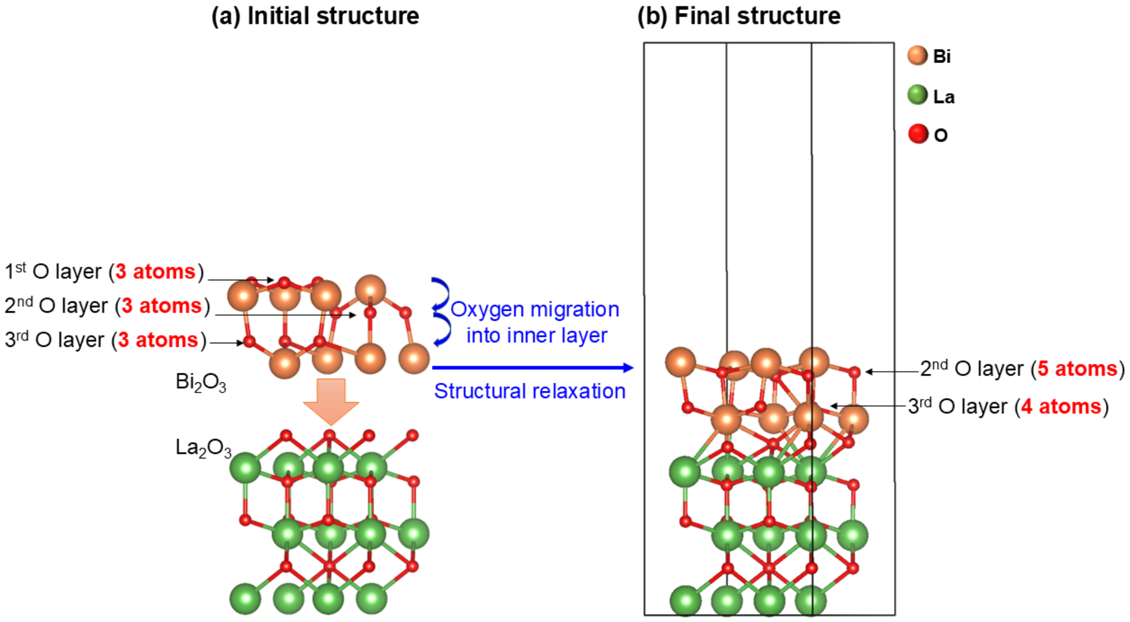

Supplementary Fig. 68 | Scheme of the formation of δ-Bi_2O_3/La_2O_3 model from (a) initial structure to (b) final structure. Source data are provided as a Source Data file.

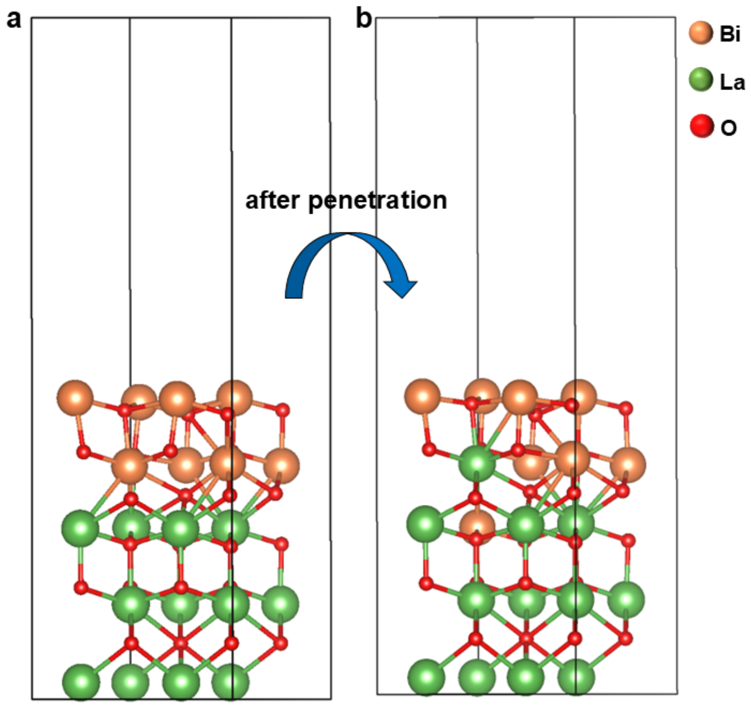

Supplementary Fig. 69 | The optimized structure of the heterojunction obtained by DFT calculation before (a) and after (b) the penetration (one Bi atom and one La atom) process at the interface. The system energy of these two structures is -350.89 and -350.64 eV, respectively.

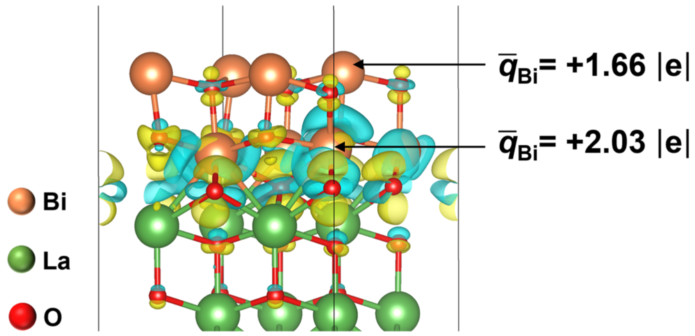

Supplementary Fig. 70 | The charge density difference plot ($\rho_{CDD} = \rho_{heterojunction} - \rho_{Bi2O3} - \rho_{La2O3}$) is also given for the obtained heterojunction model, with an isosurface value of 0.005 e/Å^3. Yellow and blue region represents an electron-accumulated area and a depleted area, respectively.

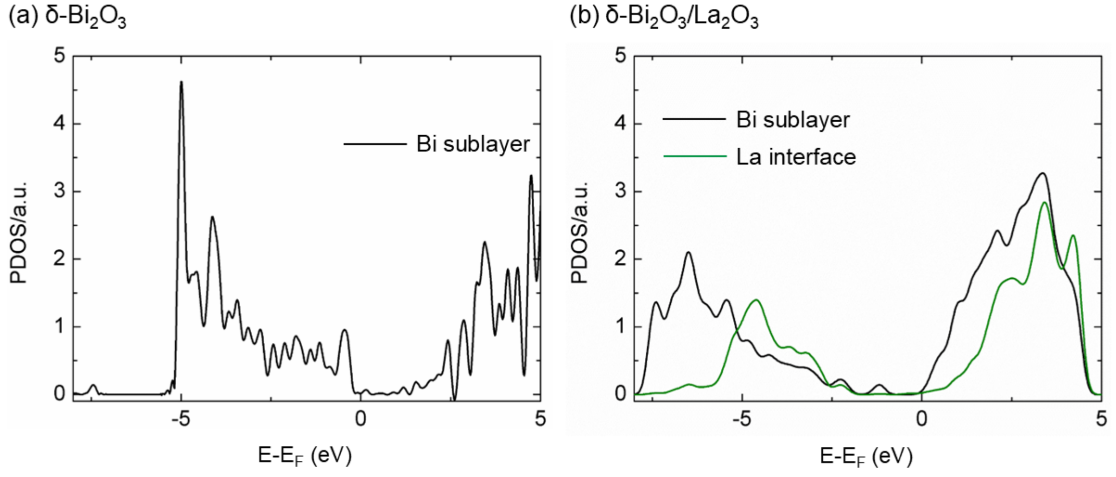

Supplementary Fig. 71 | Comparison of the projected density of states for (a) Bi atoms at sublayer of δ-Bi_2O_3 model, and (b) sublayer Bi atoms, La atoms at the interface of δ-Bi_2O_3/La_2O_3. Source data are provided as a Source Data file.

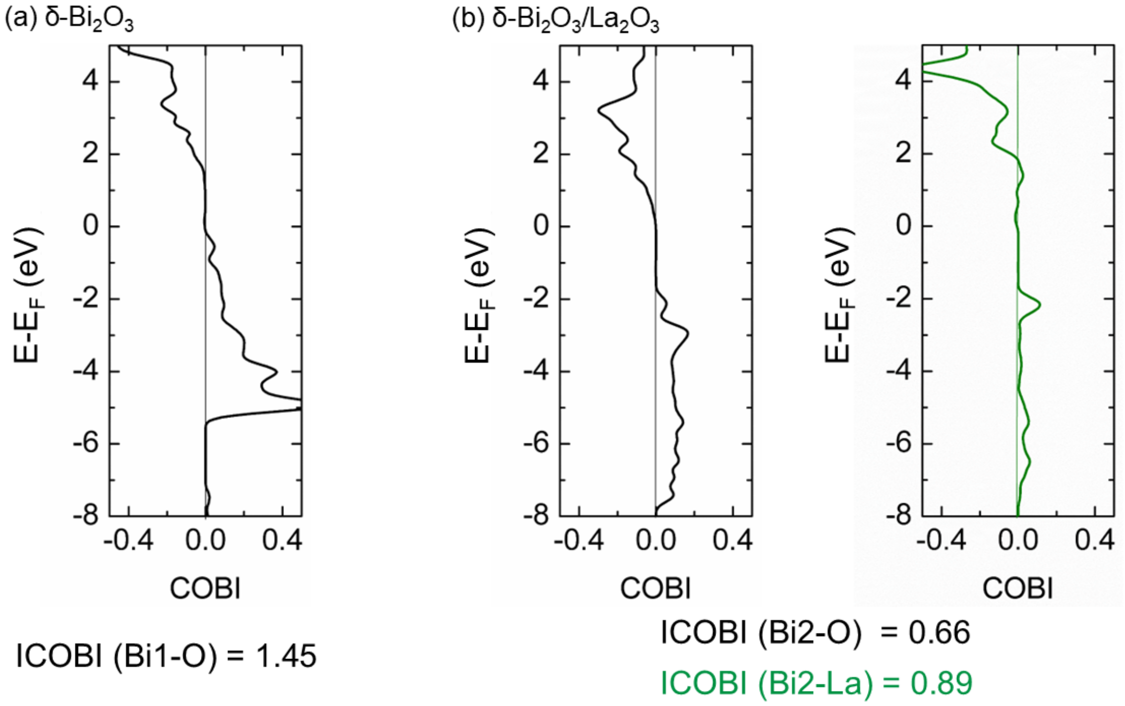

Supplementary Fig. 72 | Comparison of the crystal orbital bond index (COBI) for (a) Bi atoms at sublayer of δ-Bi_2O_3 model, and (b) sublayer Bi atoms, La atoms at the interface of δ-Bi_2O_3/La_2O_3. Source data are provided as a Source Data file.

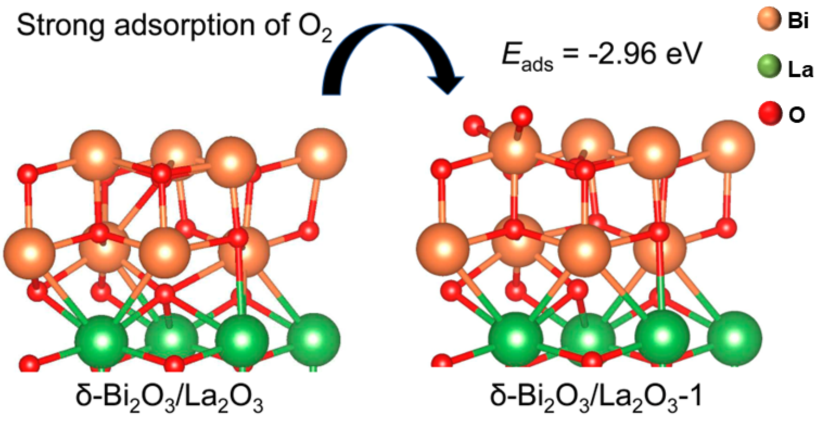

Supplementary Fig. 73 | Adsorption of O_2 molecule on δ-Bi_2O_3/La_2O_3 heterojunction. Source data are provided as a Source Data file.

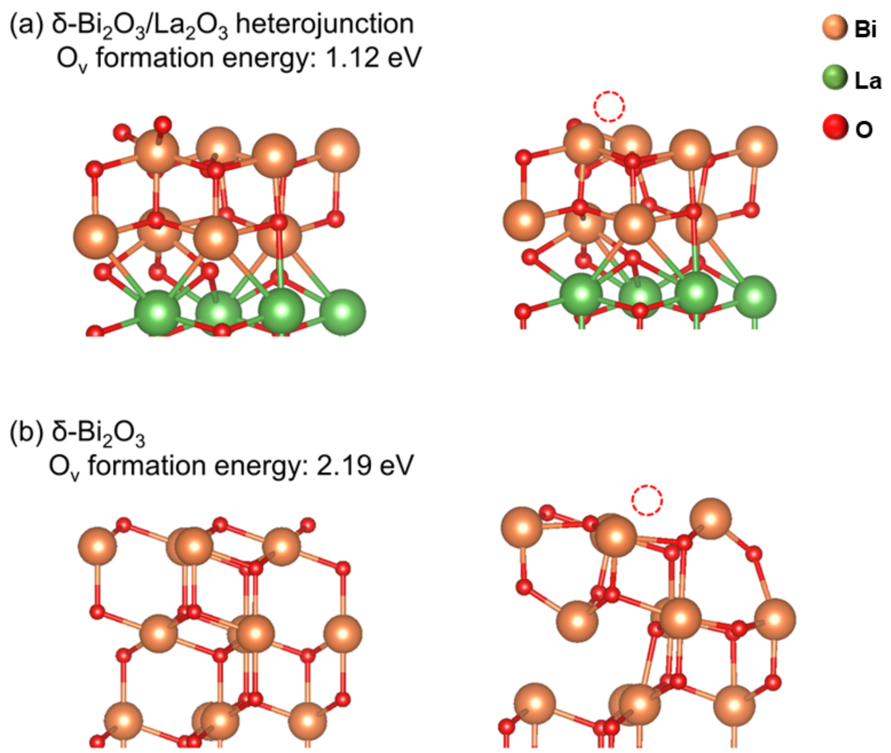

Supplementary Fig. 74 | Comparison of oxygen vacancy (O_v) formation energy for (a) δ-Bi_2O_3/La_2O_3 and (b) δ-Bi_2O_3 models. Source data are provided as a Source Data file.

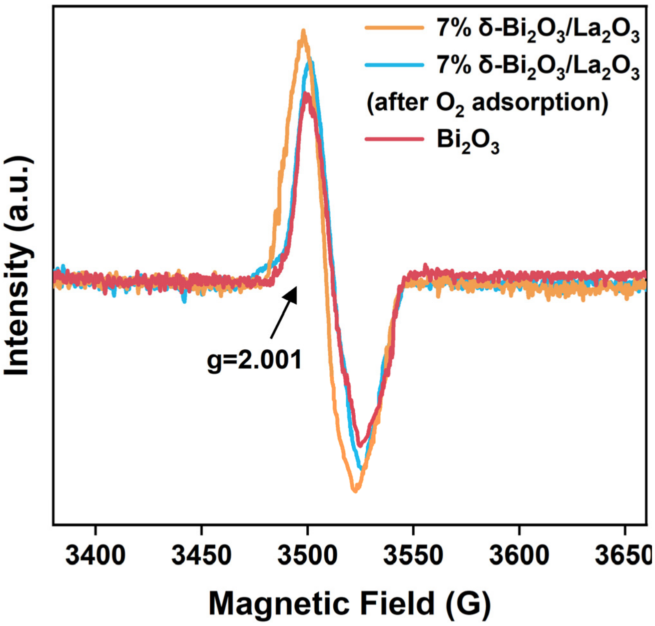

Supplementary Fig. 75 | The EPR spectra before and after adsorption of O_2 molecule on 7% δ-Bi_2O_3/La_2O_3, as well as the EPR spectrum of pure Bi_2O_3 sample. Source data are provided as a Source Data file.

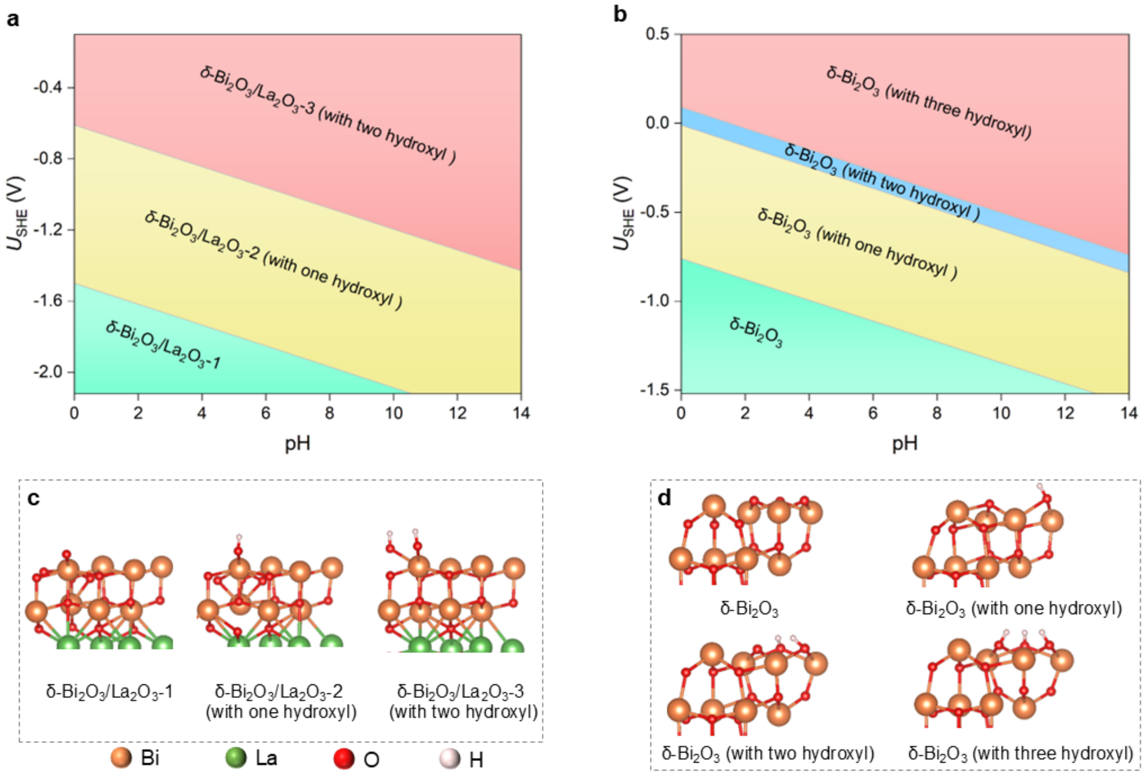

Supplementary Fig. 76 | Pourbaix diagrams of (a) the δ-Bi_2O_3/La_2O_3 heterojunction and (b) pristine δ-Bi_2O_3. (c) DFT-optimized structural models of the δ-Bi_2O_3/La_2O_3 heterojunction and its hydroxylated surface termination. (d) DFT-optimized structural models of pristine δ-Bi_2O_3 and its hydroxylated surface terminations. Source data are provided as a Source Data file.

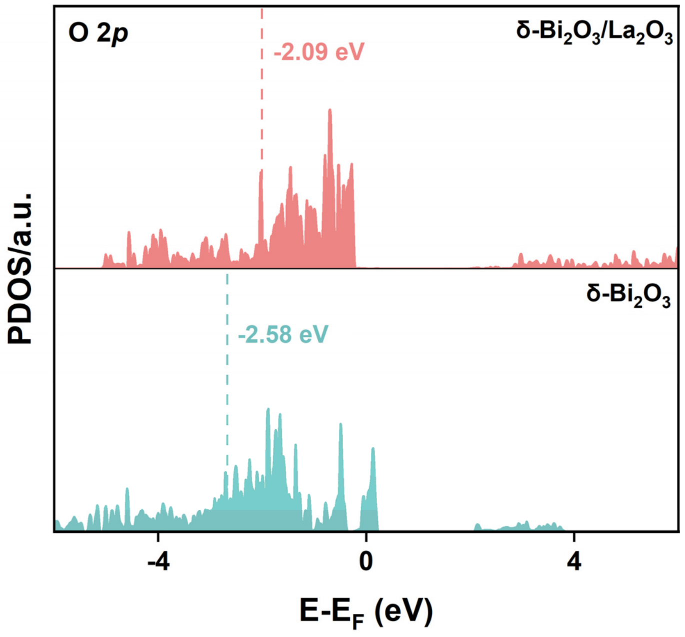

Supplementary Fig. 77 | Projected density of states (PDOS) of surface oxygen atoms in δ-Bi_2O_3 and δ-Bi_2O_3/La_2O_3. Source data are provided as a Source Data file.

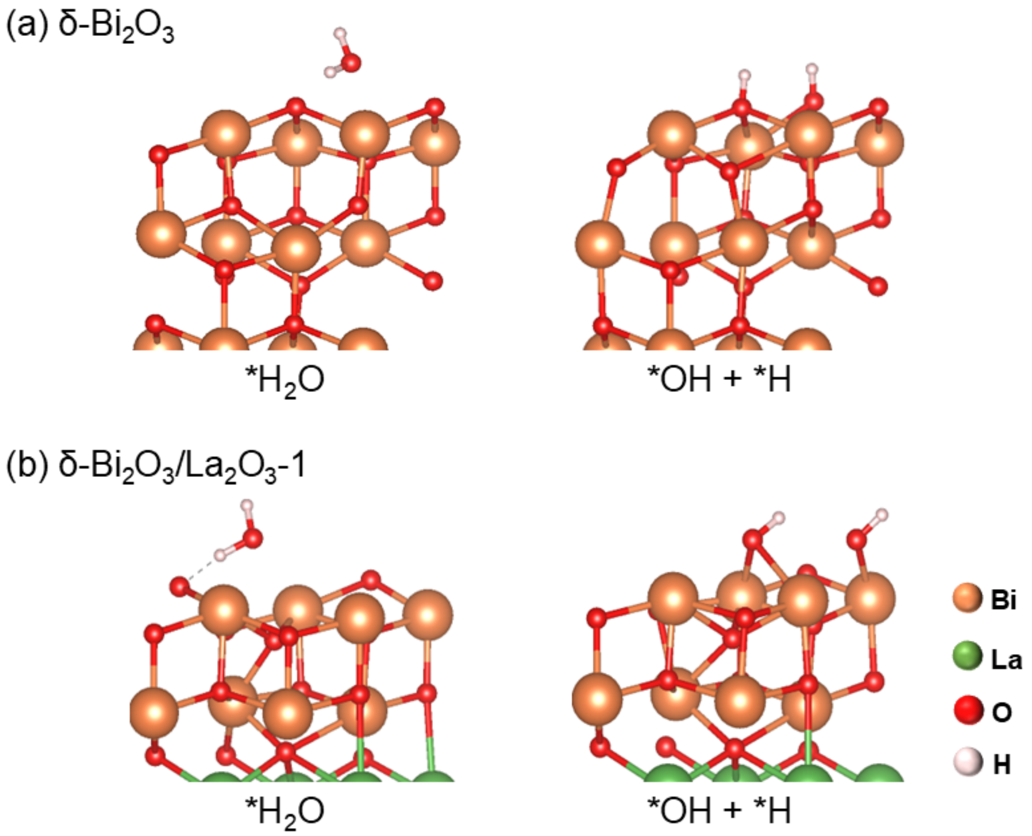

Supplementary Fig. 78 | DFT-optimized structures for *H_2O adsorption and its dissociated state (*OH+*H) on the surfaces of (a) δ-Bi_2O_3 and (b) δ-Bi_2O_3/La_2O_3-1.

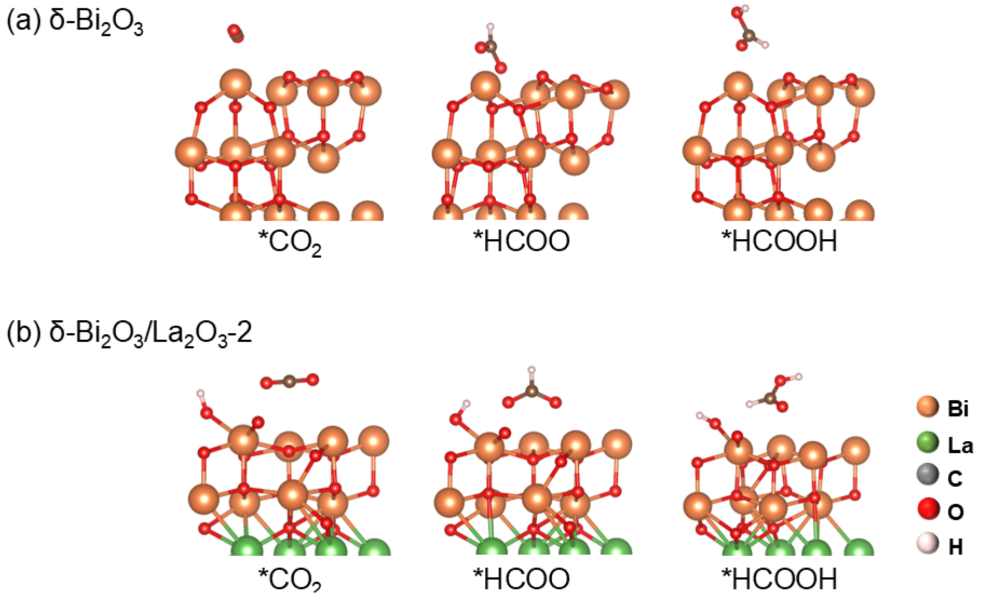

Supplementary Fig. 79 | DFT-optimized adsorption structures of $^*CO_2$, *HCOO, and *HCOOH intermediates on the surfaces of (a) δ-Bi_2O_3 and (b) δ-Bi_2O_3/La_2O_3-2.

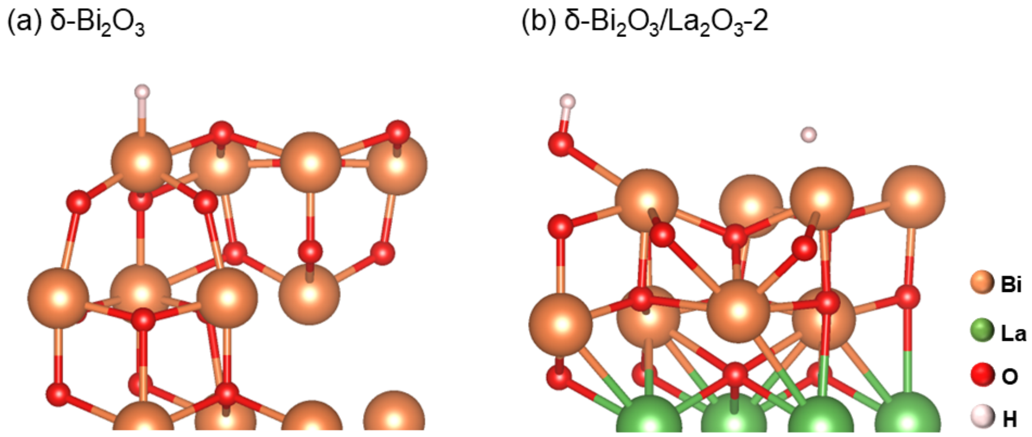

Supplementary Fig. 80 | DFT-optimized structures of *H adsorption on the surfaces of (a) δ-Bi_2O_3 and (b) δ-Bi_2O_3/La_2O_3-2.

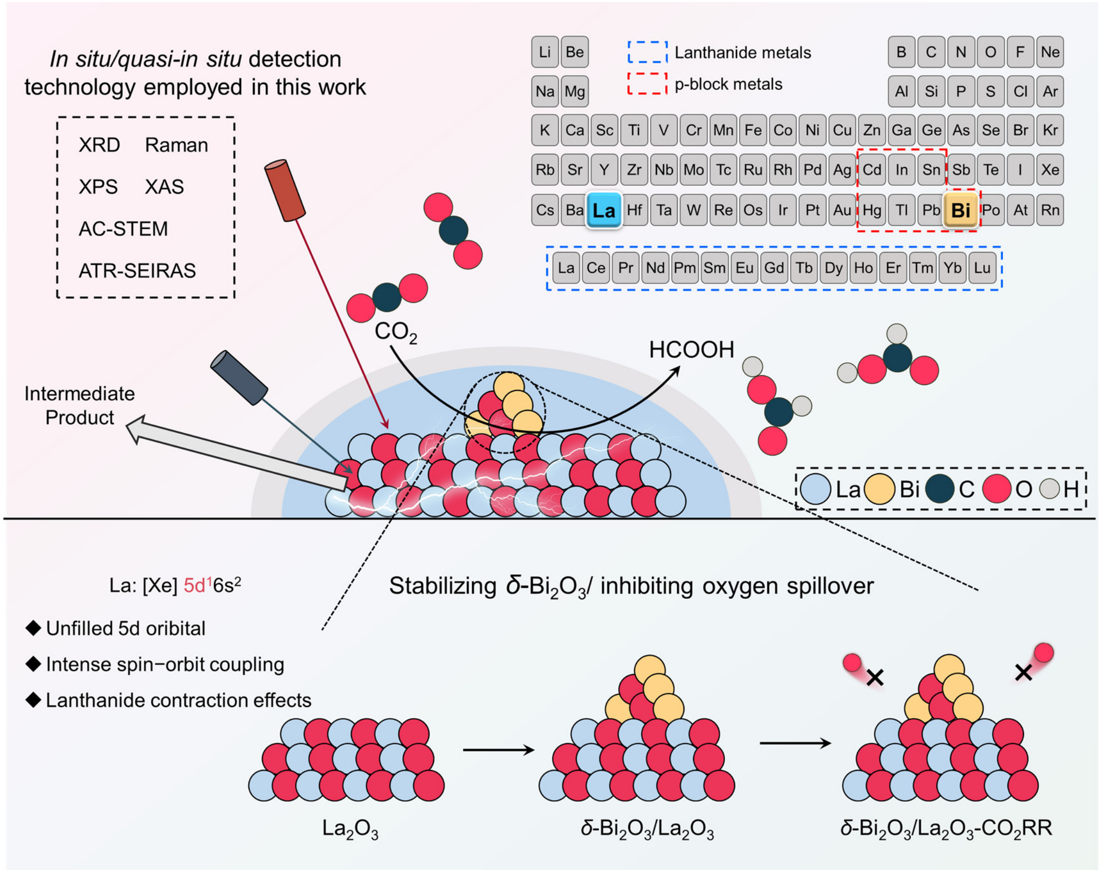

Supplementary Fig. 81 | Schematic overview of the topics discussed in this work.

Supplementary Table 1 | Structural parameters extracted from the La_2O_3 L_3-edge EXAFS fitting.

Sample	CN	S_0^2	R (Å)	ΔE_0 (eV)	σ^2 * 10^{-3} ($Å^2$)	R factor
La_2O_3	7	0.76	2.56±0.01	7.28±1.3	7.31±1.4	0.009
1%	6.5±1.9	0.76	2.55±0.02	5.83±2.0	5.52±2.1	0.015
3%	6.8±1.7	0.76	2.53±0.01	7.66±0.8	8.12±0.3	0.006
7%	6.8±1.7	0.76	2.51±0.01	4.59±2.3	9.33±2.7	0.012

CN is the coordination number.

S_0^2 is the amplitude reduction factor.

R is interatomic distance (the bond length between central atoms and surrounding coordination atoms).

ΔE_0 is edge-energy shift (the difference between the zero kinetic energy value of the sample and that of the theoretical model).

σ^2 is Debye-Waller factor (a measure of thermal and static disorder in absorber-scatterer distances).

R factor is used to value the goodness of the fitting.

Supplementary Table 2 | Faradaic Efficiencies of formate on 7% δ-Bi_2O_3/La_2O_3 in Flow-cell electrolyzer.

**Current density (mA cm^{-2})**	**$FE_{formate}$ (%) in catholyte**	**$FE_{formate}$ (%) in anolyte**	**Crossover ratio (%)**
50	85.8	2.2	2.5
80	87.8	2.7	3.0
120	88.5	3.2	3.5
160	88.7	3.5	3.8
200	89.3	3.9	4.2
250	89.7	4.1	4.4
300	89.5	4.6	4.9
350	84.1	5.8	6.4
400	78.2	6.0	7.1
500	74.3	5.5	6.9

Supplementary Table 3 | Faradaic Efficiencies of formate on 7% δ-Bi_2O_3/La_2O_3 in MEA electrolyzer.

**Current density ($mA\ cm^{-2}$)**	**$FE_{formate}$ (%) in catholyte**	**$FE_{formate}$ (%) in anolyte**	**Crossover ratio (%)**
50	72.6	6.1	7.8
100	74.3	8.8	10.5
150	73.9	11.6	13.6
200	72.7	16.6	18.6
250	70.9	21.4	23.2
300	68.0	26.7	28.2
350	66.2	29.5	30.9
400	63.6	30.7	32.5
450	58.7	32.6	35.7
500	60.6	29.3	32.6

Supplementary Table 4 | CO_2RR performance comparison of Bi-based electrocatalysts for $HCOO^-$ production.

Catalyst	Cell Type	Electrolyte	Cathodic potential or Cell Voltage	$FE_{formate}$ (%)	Stability	Ref
7% δ-Bi_2O_3/La_2O_3	H-cell	0.5 M $KHCO_3$	−0.95 V vs. RHE	97.8	210 h	This Work
	Flow cell	0.5 M $KHCO_3$	−1.58 V vs. RHE	95.7	205 h	
	MEA	1 M $KHCO_3$	2.56 V	97.3	202 h	
Bi_2O_3@C	H-cell	0.5 M $KHCO_3$	−1.40 V vs. RHE	96.7	12 h	[1]
Sn-Bi_2O_3	H-cell	0.5 M $KHCO_3$	−1.27 V vs. RHE	82.2	8 h	[2]
Bi_2O_3/p-rGO	H-cell	0.1 M $KHCO_3$	−1.09 V vs. RHE	94.3	32 h	[3]
f-Bi_2O_3	H-cell	0.1 M $KHCO_3$	−1.0 V vs. RHE	97.0	16 h	[4]
Bi_2O_3-F	H-cell	0.5 M $KHCO_3$	−0.97 V vs. RHE	97.0	100 h	[5]
Bi/Bi_2O_3	H-cell	0.5 M $NaHCO_3$	−0.86 V vs. RHE	100	24 h	[6]
In_2O_3/Bi_2O_3	H-cell	0.5 M $KHCO_3$	−0.70 V vs. RHE	99.2	30 h	[7]
Bi/Bi_2O_3	H-cell	0.5 M $KHCO_3$	−0.75 V vs. RHE	90.0	100 h	[8]
Bi-MOF	Flow cell	1 M KOH	−3.20 V vs. RHE	85.0	60 h	[9]
In-Bi_2O_3	Flow cell	1 M KOH	−1.20 V vs. RHE	91.1	25 h	[10]
Bi_2O_3/BiO_2	Flow cell	0.5 M $KHCO_3$	−0.80 V vs. RHE	90.8	30 h	[11]
	MEA	1 M KOH	3.7 V	95.4	30 h	
f-Bi_2O_3/CF	Flow cell	0.5 M $KHCO_3$	−1.10 V vs. RHE	85.0	20 h	[12]
RC-Bi_2O_3	Flow cell	1 M KOH	−0.80 V vs. RHE	96.0	40 h	[13]
Bi_2O_3-CeO_x	Flow cell	1 M KOH	−0.70 V vs. RHE	95.0	108 h	[14]
BiS	MEA	0.5 M KOH	3.86 V	90.0	150 h	[15]
$Bi(OH)_3$	MEA	0.5 M $KHCO_3$	3.0 V	85.0	200 h	[16]
nBuLi-Bi	MEA	0.1 M $KHCO_3$	1.8 V	80.0	100 h	[17]
CuBi-R	MEA	Deionized water	3.1 V	83.0	200 h	[18]
Bi_2O_3/N-CNFs	MEA	1 M KOH	3.4 V	84.0	100 h	[19]
ns-Bi_2O_3	MEA	Deionized water	3.5 V	80.0	40 h	[20]
BiIn NPs	MEA	0.1 M $KHCO_3$	3.8 V	92.0	50 h	[21]
Bi-DC	MEA	0.1 M $KHCO_3$	4.2 V	80.0	120 h	[22]

Supplementary Table 5 | Structural parameters of pure Bi_2O_3 under CO_2RR conditions extracted from the Bi L_3-edge EXAFS fitting. (S_0^2 = 0.70).

**Sample**	**CN**	**S_0^2**	**R (Å)**	**ΔE_0 (eV)**	**σ^2 * 10^{-3} ($Å^2$)**	**R factor**
OCP	3.2±0.4	0.70	2.15±0.01	−3.77±1.82	5.50±1.32	0.005
−0.65	2.8±0.4	0.70	2.18±0.02	−3.48±1.21	0.59±2.38	0.014
−0.75	2.3±0.5	0.70	2.17±0.01	−0.64±2.51	0.75±2.46	0.012
−0.85	2.2±0.4	0.70	2.17±0.02	−1.92±1.89	2.56±2.32	0.006
−0.95	2.1±0.3	0.70	2.18±0.01	−0.57±1.33	0.58±2.59	0.008
Bi_2O_3-standard	3*	0.70	2.16±0.01	−2.58±1.30	4.14±1.14	0.004

* This value was fixed during EXAFS fitting, based on the known structure of Bi_2O_3.

Supplementary Table 6 | Structural parameters of the 7% δ-Bi_2O_3/La_2O_3 under CO_2RR conditions extracted from the Bi L_3-edge EXAFS fitting. (S_0^2 = 0.70).

Sample	CN	S_0^2	R (Å)	ΔE_0 (eV)	$\sigma^2 * 10^{-3}$ (Å^2)	R factor
OCP	4.9±0.7	0.70	2.09±0.02	−6.56±1.28	6.11±2.40	0.016
−0.65	4.8±0.6	0.70	2.10±0.01	−5.94±1.30	6.13±3.00	0.017
−0.75	4.8±0.4	0.70	2.11±0.02	−5.39±1.27	7.29±3.10	0.015
−0.85	4.7±0.5	0.70	2.11±0.02	−6.05±1.02	7.84±2.10	0.019
−0.95	4.6±0.4	0.70	2.12±0.01	−5.21±1.60	8.76±3.02	0.013
−1.05	4.5±0.6	0.70	2.12±0.01	−5.22±1.32	8.69±3.56	0.010
−1.15	4.3±0.6	0.70	2.12±0.01	−7.57±0.92	7.27±1.58	0.009

Supplementary Table 7 | Structural parameters of the 7% δ-Bi_2O_3/La_2O_3 under CO_2RR conditions at -1.15V vs. RHE, extracted from the Bi L_3-edge EXAFS fitting. (S_0^2 = 0.70).

**Sample**	**CN**	**S_0^2**	**R (Å)**	**ΔE_0 (eV)**	**$\sigma^2 * 10^{-3}$ (Å^2)**	**R factor**
30 min	4.3±0.6	0.70	2.12±0.01	−7.57±0.92	7.27±1.58	0.009
60 min	4.3±0.5	0.70	2.11±0.02	−5.35±1.48	5.90±1.64	0.005
90 min	4.2±0.3	0.70	2.11±0.01	−4.99±1.21	6.30±1.20	0.006
120 min	4.1±0.4	0.70	2.11±0.01	−6.63±1.11	6.09±1.80	0.010
150 min	4.1±0.3	0.70	2.10±0.02	−6.27±1.35	5.83±1.45	0.007

References

1. Liu S-Q, *et al.* Bi_2O_3 Nanosheets Grown on Carbon Nanofiber with Inherent Hydrophobicity for High-Performance CO_2 Electroreduction in a Wide Potential Window. *ACS Nano* **15**, 17757-17768 (2021).
2. Li X, *et al.* Sn-Doped Bi_2O_3 nanosheets for highly efficient electrochemical CO_2 reduction toward formate production. *Nanoscale* **13**, 19610-19616 (2021).
3. Kou X, Zhang Y, Niu D, Han X, Ma L, Xu J. Polyethylene oxide-engineered graphene with rich mesopores anchoring Bi_2O_3 nanoparticles for boosting CO_2 electroreduction to formate. *Electrochim. Acta* **433**, 141256 (2022).
4. Tran-Phu T, Daiyan R, Fusco Z, Ma Z, Amal R, Tricoli A. Nanostructured β-Bi_2O_3 Fractals on Carbon Fibers for Highly Selective CO_2 Electroreduction to Formate. *Adv. Funct. Mater.* **30**, 1906478 (2020).
5. Liu C, *et al.* Operando formation of highly efficient electrocatalysts induced by heteroatom leaching. *Nat. Commun.* **15**, 242 (2024).
6. Li L, Ma D-K, Qi F, Chen W, Huang S. Bi nanoparticles/Bi_2O_3 nanosheets with abundant grain boundaries for efficient electrocatalytic CO_2 reduction. *Electrochim. Acta* **298**, 580-586 (2019).
7. Yang Z, *et al.* Bimetallic In_2O_3/Bi_2O_3 Catalysts Enable Highly Selective CO_2 Electroreduction to Formate within Ultra-Broad Potential Windows. *Energy Environ. Mater.* **7**, e12508 (2024).
8. Ren J, *et al.* Defect Rich Hetero-structured Bi-based Catalysts for Efficient CO_2RR to Formate in Wide Operable Windows. *Energy Technol.* **10**, (2022).
9. Chen X, *et al.* Activating inert non-defect sites in Bi catalysts using tensile strain engineering for highly active CO_2 electroreduction. *Nat. Commun.* **16**, 1927 (2025).
10. Wang J, *et al.* Stabilizing Lattice Oxygen of Bi_2O_3 by Interstitial Insertion of Indium for Efficient Formic Acid Electrosynthesis. *Angew. Chem. Int. Ed.* **64**, e202423658 (2025).
11. Feng X, *et al.* Bi_2O_3/BiO_2 Nanoheterojunction for Highly Efficient Electrocatalytic CO_2 Reduction to Formate. *Nano Letts.* **22**, 1656-1664 (2022).
12. Wang M, *et al.* In situ self-assembled bismuth oxide fractals enabling highly selective electrosynthesis of formate in flow cells at high current densities. *Nano Energy* **126**, 109659 (2024).
13. Ren X, *et al.* Reconstructed Bismuth Oxide through in situ Carbonation by Carbonate-containing Electrolyte for Highly Active Electrocatalytic CO_2 Reduction to Formate. *Angew. Chem. Int. Ed.* **63**, e202316640 (2024).
14. Yu R, *et al.* CeOx Promoted Electrocatalytic CO_2 Reduction to Formate by Assisting in the Critical Hydrogenation Step. *ACS Mater. Lett.* **4**, 1749-1755 (2022).
15. Jiang Z, *et al.* pH-Universal Electrocatalytic CO_2 Reduction with Ampere-Level Current Density on Doping-Engineered Bismuth Sulfide. *Angew. Chem. Int. Ed.* **63**, e202408412 (2024).
16. Huang Q, *et al.* In Situ Reconstructed Hydroxyl-Rich Atomic-Thin $Bi_2O_2CO_3$ Enables Ampere-Scale Synthesis of Formate from CO_2 with Activated Water Dissociation. *Adv. Mater.* **37**, 2415639 (2025).
17. Fan L, Xia C, Zhu P, Lu Y, Wang H. Electrochemical CO_2 reduction to high-concentration pure formic acid solutions in an all-solid-state reactor. *Nat. Commun.* **11**, 3633 (2020).
18. Zhang G, *et al.* Electrifying HCOOH synthesis from CO_2 building blocks over Cu-Bi nanorod arrays. *PNAS.* **121**, e2400898121 (2024).
19. Chen Z, *et al.* Nitrogen-doped carbon nanofibers confined bismuth oxide nanocrystals boost high single-pass CO_2-to-formate conversion in large area membrane electrode assembly electrolyzers. *Appl. Surf. Sci.*

620, 156867 (2023).

20. Tan Z*, et al.* Continuous Production of Formic Acid Solution from Electrocatalytic CO_2 Reduction Using Mesoporous Bi_2O_3 Nanosheets as Catalyst. *CCS Chem.* **6**, 100-109 (2024).

21. Yao K*, et al.* Metal-organic framework derived dual-metal sites for electroreduction of carbon dioxide to HCOOH. *Appl. Catal. B-Environ.* **311**, 121377 (2022).

22. Li W*, et al.* Beyond Leverage in Activity and Stability toward CO_2 Electroreduction to Formate over a Bismuth Catalyst. *ACS Catal.* **14**, 8050-8061 (2024).
