# Supplementary material for: Stabilizing sub-2 nm δ-Bi2O3 via strong lanthanide-oxide-support interaction for durable CO2 electroreduction to formate
Source: Nat Commun. 2026 Apr 24;17:5685. doi: 10.1038/s41467-026-71855-5 (PMC13319208; doi:10.1038/s41467-026-71855-5)
Supplement: Supplementary file 2 — Description of Additional Supplementary Files [file 41467_2026_71855_MOESM2_ESM.pdf]

Description of Additional Supplementary Files

File Name: Supplementary Data 1

Description: DFT-optimized structures and adsorption configurafions for CO_2RR intermediates on δ-Bi_2O_3 and δ-Bi_2O_3/La_2O_3 in Figures 5, S69, S73, S74, and the hydroxylated surface terminations in Figure S76.
