# Supplementary material for: Stabilizing sub-2 nm δ-Bi2O3 via strong lanthanide-oxide-support interaction for durable CO2 electroreduction to formate
Source: Nat Commun. 2026 Apr 24;17:5685. doi: 10.1038/s41467-026-71855-5 (PMC13319208; doi:10.1038/s41467-026-71855-5)
Supplement: Supplementary file 4 — Transparent Peer Review File [file 41467_2026_71855_MOESM4_ESM.pdf]

nature portfolio

Peer Review File

Stabilizing Sub-2 nm δ-Bi_2O_3 via Strong Lanthanide-Oxide-Support Interaction for Durable CO2 Electroreduction to Formate

Corresponding Author: Dr Fa Yang

Parts of this Peer Review File have been redacted as indicated to remove third-party material.

This file contains all reviewer reports in order by version, followed by all author rebuttals in order by version.

Version 0:

Reviewer comments:

Reviewer #1

(Remarks to the Author)
The work by Fa Yang and coworkers reported a La2O3-socketed δ-Bi2O3 heterojunction catalyst (δ-Bi2O3/La2O3) for CO2 reduction over 200 h. The strong metal oxide–support interaction (SMOSI) between La2O3 and δ-Bi2O3 enhanced the Biδ+ oxidation state stability as well as the catalysis stability. The catalyst characterizations were systematic and the performance was great. However, there are still some significant issues including structure characterizations and analysis that need to be improved.

(1) The XRD and HRTEM results claim an obvious contraction in the lattice for both δ-Bi2O3 and La2O3. The authors said, “Bi3+ (0.096 nm) entering into the La2O3 lattice and partially replacing La3+(0.106 nm).” The Bi3+ doping may happen, especially in the bottom-up synthetic process. However, the ionic radii of Bi3+ is similar to that of La3+. The content of Bi determined by ICP-AES is lower than 7% and most Bi exists in the Bi2O3 moiety, why the lattice is obviously contracted? Apart from these, whether the La3+ doped in the Bi2O3 or not? The doped La3+ is a bit larger than Bi3+, whether the lattice is expanded or not, while the fitting results of the EXAFS (Table S2, S3) exhibit a contracted lattice of Bi2O3.
(2) There are error bars in Figure S20 while no error bars in Figure 2. The authors said, “7% δ-Bi2O3/La2O3 exhibits a FEHCOO– of ≥ 90% over a wide potential, which is superior to that of pure δ-Bi2O3 powder with a FEHCOO– of 79.3% at –1.05 VRHE.” However, the FEHCOO– over pure δ-Bi2O3 can also reach around ~90% at –0.95 VRHE (Figure 2a). The comparison is unreasonable.
(3) The authors synthesized δ-Bi2O3/La2O3 catalysts with different Bi content from 0.8, 2.7 to 7.1 and considered low-density δ-Bi2O3 sites cannot effectively promote the reaction. So this work focuses on a 7% δ-Bi2O3/La2O3 sample. In fact, we don’t know if the catalysts with more content of Bi can show superior performance than the 7.1% content one. Maybe a higher Bi content catalyst is needed to study the relatively appropriate δ-Bi2O3/La2O3 ratio. The work shows a current density of 500 mA cm–2 at “only” –1.85 VRHE. However, there are some formate production works (The Innovation, 2025, 6: 100844; Angew. Chem. Int. Ed. 2025, 137, e202423370; Angew. Chem. Int. Ed. 2025, 64, e202507422.) that exhibit much lower potential to reach 500 mA cm–2. This work shows a good performance of more than 200 h at 350 mA cm-2 in the flow cell which is different from the results in Figure 2h.
(4) The authors said, “The characteristic peak for adsorbed intermediate *CO2·– at ~1262 cm–1 (Figure 4f).” However, there are interference peaks at 1100~1300 cm-1 owing to the Si-O signals (Anal. Chem. 2024, 96, 10111; Phys. B: 2000, 292, 286.) and the labeled peaks (Si-O-Si) by the authors at ~1200 cm-1 in the absorbance spectra are not clear (Figure 4f). Actually, we don’t consider that there are absorbance peaks at ~1200 cm-1. In addition, there are no reference experiments, such as carbon and Au film ATR-SEIRA spectra. Hence, we can’t determine which peak is corresponding to HCOO–.
(5) The authors set up Bi2O3(111)/La2O3(001) heterojunction as the models for DFT calculations. However, the authors also consider that Bi3+ enters into the La2O3 lattice and partially replaces La3+ to form Bi–O–La bonds. It is the main reason that leads to the average coordination (Bi-O) of Bi in the heterojunctions exceeding 6 (Table S3). We also consider that La enters Bi2O3 structures, too. Hence, the real models for DFT calculations should be composed by La-doped Bi2O3(111) and Bi-doped La2O3(001).
(6) The reasonable range of EXAFS fitting parameters, including R factor < 0.02. However, there are some results in Tables S2 and S3 that don’t comply with this rule. The R factor for pure Bi2O3 at -0.65 V even reaches 0.024. Apart from these, all

the fitting results using S02 of 0.31, which is much more unreasonable. The rational range of the S02 is 0.600 < S02 < 1.000. (7) There are many experiment details missing, such as the electrode preparation methods. The authors use PTFE in the MEA electrolyzer while the work doesn't show the method to overcome the conductivity issues. The information of the GDL in the flow cell is also missing. Apart from these, La2O3 or the heterojunctions show a relatively large morphology of more than 1 μm with average diameters of the pores of about 2.39 nm. La2O3 has poor conductivity and the method to improve the catalyst conductivity is also missing. Then, the potentials in the H cell were referenced to a reversible hydrogen electrode (RHE) without iR compensation while the potentials in the flow cell and the voltages in the MEA weren't clarified. Finally, the produced formate can move across the AEM to the cathode and encounter its reoxidation, especially in the MEA system. However, the authors don't explain it.

Reviewer #2

(Remarks to the Author)
The manuscript titled "Stabilizing Sub-2 nm δ-Bi_2O_3 via Strong Lanthanide-Oxide-Support Interaction for Durable CO2 Electroreduction to Formate Beyond 200 Hours" by Qianmin Wu et al discusses the new Bi-La catalyst for CO2RR, which shows good performance for formate production. Authors demonstrate the stability of the catalyst, and, in particular, the stabilization of Bi cationic nature by La oxide support. The presented results are interesting, however, there are some issues regarding the proposed structure model of the catalyst which need to be explained or corrected.

Authors continuously refer to their catalyst as "delta-Bi2O3". However, I do not see any conclusive evidence that the formed oxide particles indeed assume delta phase and not some other of the numerous possible crystallographic phases for bismuth oxides. In particular, authors show ex-situ and in-situ XRD data for their catalysts. However, these have little resemblance (if any) to the XRD of delta Bi2O3. Not only all peak positions are different, but also relative Bragg peak intensities do not match those in the pure Bi2O3. Perhaps even more critically, I do not understand why there should be any peaks of bismuth oxide at all in the XRD data, if authors model featuring 2 nm particles is correct. Such small particles would be X-ray amorphous, and would not produce any strong Bragg reflections at all. I would rather consider that Bi is incorporated into lanthanum oxide support, forming a new oxide phase that has nothing to do with the delta Bi2O3. Can authors exclude incorporation of significant amounts of Bi into the support?

Comparison of FE between different catalysts is confusing. Authors state that "7% δ-Bi2O3/La2O3 exhibits a FEHCOO– of ≥ 90% over a wide potential interval of –0.65 to –1.25 VRHE with a maximum FEHCOO– of 97.8% at –0.95 VRHE, which is superior to that of pure δ-Bi2O3 powder with a FEHCOO– of 79.3% at –1.05 VRHE." However, a few lines below authors say that for pure δ-Bi2O3 "FEs of HCOO– distinctly drop from 88.5% to 45.6% only after electrolysis for 30 h at –0.95 VRHE." From this I conclude that that for pure Bi2O3 FE to formate is higher and -0.95V (88.5%) than at -1.05 V (79.3%). Why then the FE for La2O3-supported catalyst at -0.95V is compared with that of pure Bi2O3 at -0.95V?

Authors also compare performance of catalysts with different amounts of bismuth oxide (1%, 3% and 7%). The best performance was observed for the catalyst with the highest bismuth loading. What will happen, if one would further increase the bismuth loading? Would this catalyst show even better catalytic properties?

When discussing XAS data, authors state that "The X-ray absorption near edge structure (XANES) spectra of the Bi L3-edge (Fig. S32a) reveal that the oxidation state of Bi in the initial δ-Bi2O3 is below +3 as its absorption edge shifts to lower energy than that of the Bi2O3 standard." However, this is likely not true. The position of absorption edge depends not only on the chemical state of Bi, but also on its local environment, in particular, on interatomic distances. In particular, the shift of the absorption edge to higher energies is likely a result of shortened Bi-O bonds, observed also in XAS and XRD, but is not related changes in oxidation state.

To conclude, the major revision of the manuscript is necessary.

Reviewer #3

(Remarks to the Author)
The manuscript titled "Stabilizing Sub-2 nm δ-Bi2O3 via Strong Lanthanide-Oxide-Support Interaction for Durable CO2 Electroreduction to Formate Beyond 200 Hours" presents intriguing experimental results and a potentially impactful heterojunction catalyst for CO2RR. However, the reviewer finds that the theoretical (DFT) section suffers from significant clarity, consistency, and interpretability issues, which undermine the mechanistic claims. Thus, major revision is required before further consideration. Specific concerns are detailed below:

(1) The description of oxygen atom migration is contradictory. The main text states: "as several oxygen atoms transfer from the first δ-Bi2O3 layer to the second" and simultaneously, "Specifically, one oxygen atom moves from the first layer to the second." This inconsistency must be resolved: How many oxygen atoms migrate in the model used?

(2) The text compares Bi–La and Bi–Bi distances in δ-Bi2O3 and the heterojunction, suggesting "Bi–La distances are smaller than in the pure δ-Bi2O3(111) surface." However, pure δ-Bi2O3 does not contain La, and thus Bi–La bonds cannot exist in that structure. This statement must be revised to reflect meaningful comparisons.

(3) The manuscript states: "The theoretical results are in excellent agreement with experimental observations..." But it remains vague: Which specific experimental observations do the computational results agree with? Furthermore, the

mechanistic origin of oxygen migration and layer contraction is not convincingly explained.

(4) The Bader results show a charge increase from 0.65 |e| to 2.53 |e| in δ-Bi_2O_3/La_2O_3. The meaning of “total charge” per structure is unclear. δ-Bi_2O_3 as a neutral solid should not carry net positive charge.

(5) The following sentence is internally contradictory: "The process is endothermic, with an adsorption energy of −2.96 eV..." A negative adsorption energy indicates an exothermic process, not endothermic. This appears to be a typographical or conceptual error, and must be corrected.

(6) The structure used for HER and CO_2RR studies (oxygen-molecule adsorbed δ-Bi_2O_3/La_2O_3-1 model) lacks sufficient justification: Is this structure relevant and stable under operating potentials? Surface Pourbaix diagram could be calculated to help understand this. Could surface oxygen atoms, rather than the interface, dominate the activity difference between δ-Bi_2O_3 and δ-Bi_2O_3/La_2O_3-1 model observed?

Minor Comments
“Bader charge analyses reveals” → should be “analysis reveals”
“is the thermodynamically favorable” → should be “thermodynamically favorable”

Version 1:

Reviewer comments:

Reviewer #1

(Remarks to the Author)
The authors have addressed all the proposed questions. I have no more concerns. The manuscript is ready for publication.

Reviewer #2

(Remarks to the Author)
During the revision, authors have addressed my previous comments, and paper can be published now, if the following is addressed: Authors have now included Figures S23 and S24, showing XANES simulation results. Please provide the details of these simulations. Which code was used? What were the values of the most important simulation parameters? How the structures used for simulations were constructed?

Reviewer #3

(Remarks to the Author)
The authors have addressed the reviewer’s previous concerns regarding the DFT calculations. The inconsistencies in the description of oxygen migration have been clarified, and the structural comparisons have been revised to avoid misleading statements. The connection between theoretical results and experimental observations is now more clearly articulated, and the stability and relevance of the computational model under operating conditions are better justified. Overall, the revisions have significantly improved the clarity and rigor of the theoretical part, and the reviewer believes the manuscript is now suitable for publication.

Version 2:

Reviewer comments:

Reviewer #2

(Remarks to the Author)
During the revision authors have addressed my previous comments. Manuscript can be published now.

Open Access This Peer Review File is licensed under a Creative Commons Attribution 4.0 International License, which permits use, sharing, adaptation, distribution and reproduction in any medium or format, as long as you give appropriate credit to the original author(s) and the source, provide a link to the Creative Commons license, and indicate if changes were made.
In cases where reviewers are anonymous, credit should be given to 'Anonymous Referee' and the source.
The images or other third party material in this Peer Review File are included in the article's Creative Commons license, unless indicated otherwise in a credit line to the material. If material is not included in the article's Creative Commons license and your intended use is not permitted by statutory regulation or exceeds the permitted use, you will need to obtain permission directly from the copyright holder.
To view a copy of this license, visit https://creativecommons.org/licenses/by/4.0/

Point-by-point Responses to the Reviewers' Comments

We thank the editors and referees for their time and comments on our manuscript. These comments are all valuable and very helpful for revising and improving our paper, as well as the important guiding significance to our researches. In the following, by supplementing new data and more discussions, we provide a point-by-point response to their comments. The author's responses are marked in red, and all changes have been highlighted in yellow in the revised main text and the revised Supporting Information.

Reviewer: 1

The work by Fa Yang and coworkers reported a La_2O_3-socketed δ-Bi_2O_3 heterojunction catalyst (δ-Bi_2O_3/La_2O_3) for CO_2 reduction over 200 h. The strong metal oxide–support interaction (SMOSI) between La_2O_3 and δ-Bi_2O_3 enhanced the $Bi^{\delta+}$ oxidation state stability as well as the catalysis stability. The catalyst characterizations were systematic and the performance was great. However, there are still some significant issues including structure characterizations and analysis that need to be improved.

[Reply]: We greatly appreciate the reviewer's positive comments on our study, and thank for the insightful suggestions that have inspired us to supplement additional experimental data and computational results along with more clarified discussions, which we believe have further improved the quality of this paper.

(1) The XRD and HRTEM results claim an obvious contraction in the lattice for both δ-Bi_2O_3 and La_2O_3. The authors said, "Bi^{3+} (0.096 nm) entering into the La_2O_3 lattice and partially replacing La^{3+}(0.106 nm)." The Bi^{3+} doping may happen, especially in the bottom-up synthetic process. However, the ionic radii of Bi^{3+} is similar to that of La^{3+}. The content of Bi determined by ICP-AES is lower than 7% and most Bi exists in the Bi_2O_3 moiety, why the lattice is obviously contracted? Apart from these, whether the La^{3+} doped in the Bi_2O_3 or not? The doped La^{3+} is a bit larger than Bi^{3+}, whether the lattice is expanded or not, while the fitting results of the EXAFS (Table S2, S3) exhibit a contracted lattice of Bi_2O_3.

[Reply]: Many thanks for your insightful comments regarding the lattice contractions over δ-Bi_2O_3/La_2O_3. First, we agree with the reviewer's judgment that the obvious lattice contraction in La_2O_3 is not primarily caused by Bi doping, but rather reflects interfacial lattice strain in the La_2O_3 phase induced by strong coupling with sub-2 nm δ-Bi_2O_3 particles. Structural analysis of the aberration-corrected HAADF-STEM and HRTEM images (Fig. 1g and Supplementary Figs. 17–19) reveals a significant lattice mismatch at the interface between the (111) plane of δ-Bi_2O_3 and the (002) plane of La_2O_3. Such interfacial lattice mismatch leads to structural distortion of both the anchored δ-Bi_2O_3 nanoparticles and the La_2O_3 support, reflecting the

strong metal oxide–support interaction (SMOSI), and thereby induces compressive strain in the La_2O_3 support. To further determine whether Bi is incorporated into the La_2O_3 lattice, we performed additional X-ray absorption spectroscopy (XAS) measurements at the La L_3-edge. As shown in **Figs. R1, R2** and **Table R1**, the fitted La–O bond lengths are shorter in δ-Bi_2O_3/La_2O_3 than in pure La_2O_3, supporting lattice contraction in the La_2O_3 support. Notably, the La absorption edge position in δ-Bi_2O_3/La_2O_3 is essentially identical to that of pure La_2O_3 (Fig. R1a), demonstrating that Bi is not significantly incorporated into the La_2O_3 lattice. We further carry out theoretical XAS simulations for Bi-doped La_2O_3 and pure α-, β-, and δ-Bi_2O_3 to identify the local coordination structure of Bi in δ-Bi_2O_3/La_2O_3 by comparing the simulated and experimental XANES spectra. As shown in **Fig. R3**, the simulated Bi L_3-edge XANES spectrum for Bi-doped La_2O_3 does not match the experimental spectrum of the δ-Bi_2O_3/La_2O_3 sample, whereas the simulated spectrum for pure δ-Bi_2O_3 shows excellent agreement, indicating that Bi is not significantly incorporated into the La_2O_3 lattice. Moreover, the simulated Bi L_3-edge XANES spectra for α-Bi_2O_3 and β-Bi_2O_3 do not match the experimental spectrum, also distinguishing that the Bi_2O_3 in the composite exists as the δ-phase. Given that the ionic radius of La^{3+} (0.106 nm) is larger than that of Bi^{3+} (0.096 nm), the incorporation of La into the δ-Bi_2O_3 lattice would be expected to induce lattice expansion. However, a lattice contraction is observed in the δ-Bi_2O_3 layer of δ-Bi_2O_3/La_2O_3 heterostructure, suggesting that La doping into δ-Bi_2O_3 is unlikely. To this end, we further performed La L_3-edge XANES simulations based on these structural models—La-doped α-Bi_2O_3, La-doped β-Bi_2O_3, La-doped δ-Bi_2O_3, and undoped La_2O_3—to identify the local coordination environment of La in the δ-Bi_2O_3/La_2O_3 catalyst. As shown in **Fig. R4**, the simulated La L_3-edge XANES spectra for La-doped α-, β-, and δ-Bi_2O_3 show poor agreement with the experimental spectrum of the δ-Bi_2O_3/La_2O_3 sample, whereas the simulated spectrum for pristine La_2O_3 closely matches the experimental data, indicating negligible incorporation of La into the δ-Bi_2O_3 lattice. It should be noted that the realistic heterojunction structure is much more complicated, and the penetration of Bi and La at the interface may occur. We thus constructed a δ-Bi_2O_3/La_2O_3 heterostructure with an intermixed interface, in which one Bi atom substitutes into the La_2O_3 lattice and one La atom incorporates into the δ-Bi_2O_3 lattice (**Fig. R5**). By comparing the system energies of different interface configurations, we find that the model after penetration is 0.25 eV higher in energy than the non-intermixed δ-Bi_2O_3/La_2O_3 interface, indicating that the latter is thermodynamically more stable. Consequently, large-scale interdiffusion of Bi and La atoms is unlikely to occur spontaneously.

Figs R1a, R1b, R2, R3, R4, R5 have been added as Supplementary Figs. 20, 21, 22, 23, 24, 69 in the revised Supporting Information, respectively. Table R1 has been added as Supplementary

Table 1 in the revised Supporting Information.

Revised parts in the manuscript:

Page 5, lines 179–201

To further determine whether interfacial Bi and La atoms interdiffuse, we perform X-ray absorption spectroscopy (XAS) measurements and simulations. First, the La L_3-edge XANES spectra show that the La absorption edge position in the δ-Bi_2O_3/La_2O_3 is essentially identical to that of pure La_2O_3 (Supplementary Fig. 20), demonstrating that Bi is not significantly incorporated into the La_2O_3 lattice. Notably, the fitted La–O bond lengths are shorter in δ-Bi_2O_3/La_2O_3 than in pure La_2O_3, further suggesting a lattice contraction in the La_2O_3 support (Supplementary Fig. 21, 22 and Supplementary Table 1). We further carry out theoretical XAS simulations for Bi-doped La_2O_3 and pure α-, β-, and δ-Bi_2O_3 to identify the local coordination structure of Bi in δ-Bi_2O_3/La_2O_3 by comparing the simulated and experimental XANES spectra. As shown in Supplementary Fig.23, the simulated Bi L_3-edge XANES spectrum for Bi-doped La_2O_3 does not match the experimental spectrum of the δ-Bi_2O_3/La_2O_3 sample, whereas the simulated spectrum for pure δ-Bi_2O_3 shows excellent agreement, also verifying negligible incorporation of Bi into the La_2O_3 lattice. Besides, the simulated Bi L_3-edge XANES spectra for α-Bi_2O_3 and β-Bi_2O_3 do not match the experimental spectrum, further distinguishing that the Bi_2O_3 in the composite exists as the δ-phase. Subsequently, to determine whether La is incorporated into the δ-Bi_2O_3 lattice, we perform La L_3-edge XANES simulations based on these structural models—La-doped α-Bi_2O_3, La-doped β-Bi_2O_3, La-doped δ-Bi_2O_3, and undoped La_2O_3. As shown in Supplementary Fig. 24, the simulated La L_3-edge XANES spectra for La-doped α-, β-, and δ-Bi_2O_3 show poor agreement with the experimental spectrum of the δ-Bi_2O_3/La_2O_3 sample, whereas the simulated spectrum for pristine La_2O_3 closely matches the experimental data, also indicating that La is not significantly incorporated into the δ-Bi_2O_3 lattice. Consequently, large-scale interdiffusion of Bi and La atoms is unlikely to occur.

Page 16, lines 537–547

Notably, our previous XAS measurements and simulations in Supplementary Figs. 20-24 have demonstrated that the large-scale interdiffusion of Bi and La atoms is unlikely to occur spontaneously. However, it should be noted that the realistic heterojunction structure is much more complicated, and the penetration of Bi and La atoms at the interface may be possible. We thus constructed a δ-Bi_2O_3/La_2O_3 heterostructure with an intermixed interface, in which one Bi atom substitutes into the La_2O_3 lattice and one La atom incorporates into the δ-Bi_2O_3 lattice (Supplementary Fig. 69). By comparing the system energies of different interface configurations, we find that the model after penetration is 0.25 eV higher in energy than the

non-intermixed δ-Bi_2O_3/La_2O_3 interface, implying that the latter is thermodynamically more stable. Consequently, the δ-Bi_2O_3/La_2O_3 model in Fig. 5a reasonably represents the experimental heterojunction catalyst.

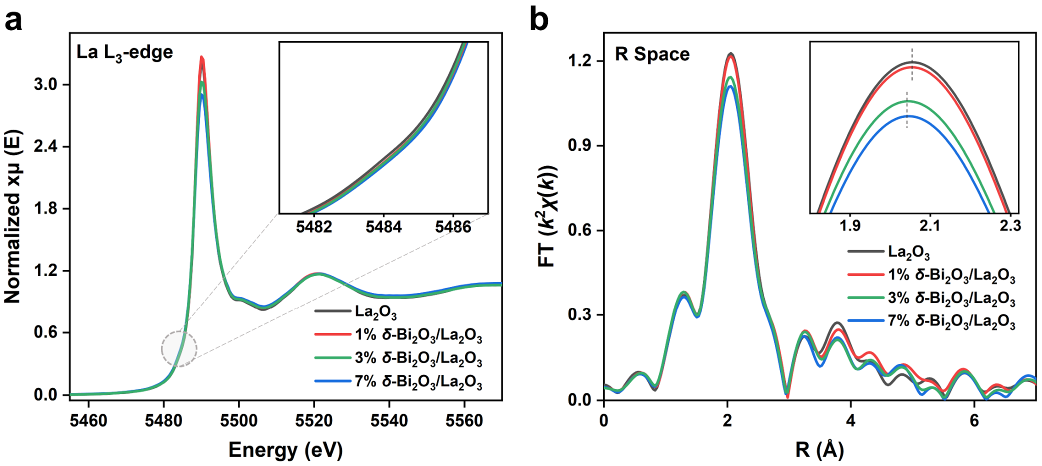

Fig. R1 | (a) La L_3-edge XANES spectra and (b) corresponding k^2-weighted FT-EXAFS spectra over pure La_2O_3 and δ-Bi_2O_3/La_2O_3.

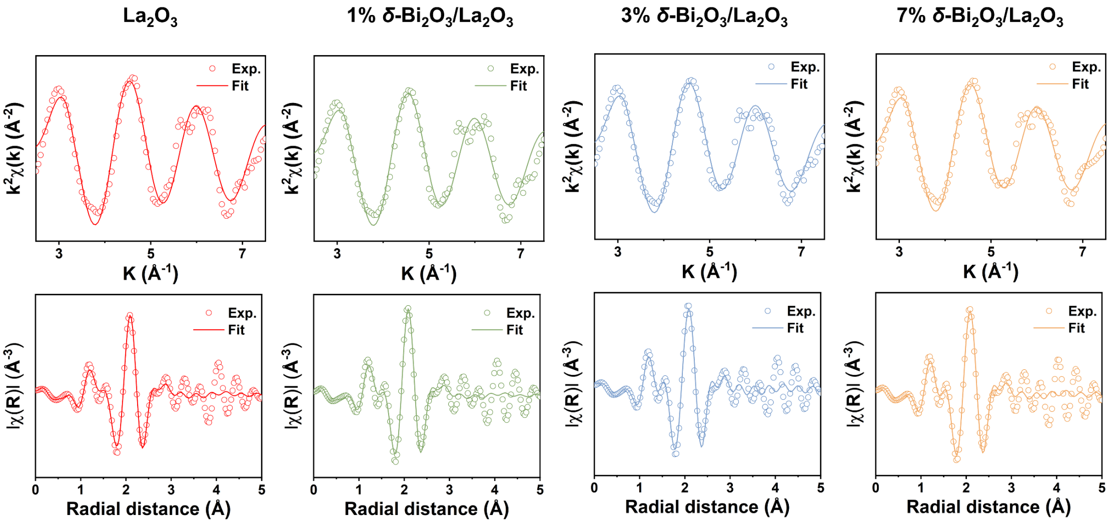

Fig. R2 | k^2-weighted La L_3-edge EXAFS oscillations (up) and their associated Fourier transforms (down) for La_2O_3 and δ-Bi_2O_3/La_2O_3; Experimental data, dotted lines; Fitting curves, solid lines.

Table R1 | Structural parameters extracted from the L_3-edge EXAFS fitting.

Sample	CN	S_0^2	R (Å)	ΔE_0 (eV)	$\sigma^2 * 10^{-3}$ (Å^2)	R factor
La_2O_3	7	0.76	2.56±0.01	7.28±1.3	7.31±1.4	0.009
1%	6.5±1.9	0.76	2.55±0.02	5.83±2.0	5.52±2.1	0.015
3%	6.8±1.7	0.76	2.53±0.01	7.66±0.8	8.12±0.3	0.006
7%	6.8±1.7	0.76	2.51±0.01	4.59±2.3	9.33±2.7	0.012

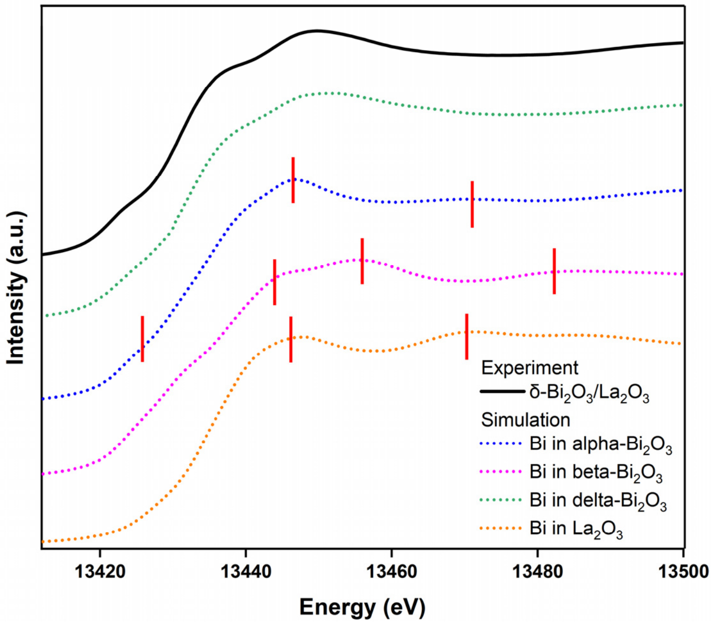

Fig. R3 | Comparison of the experimental Bi L_3-edge XANES spectrum of the δ-Bi_2O_3/La_2O_3 sample with simulated spectra for various Bi_2O_3 phases and Bi-doped La_2O_3 models.

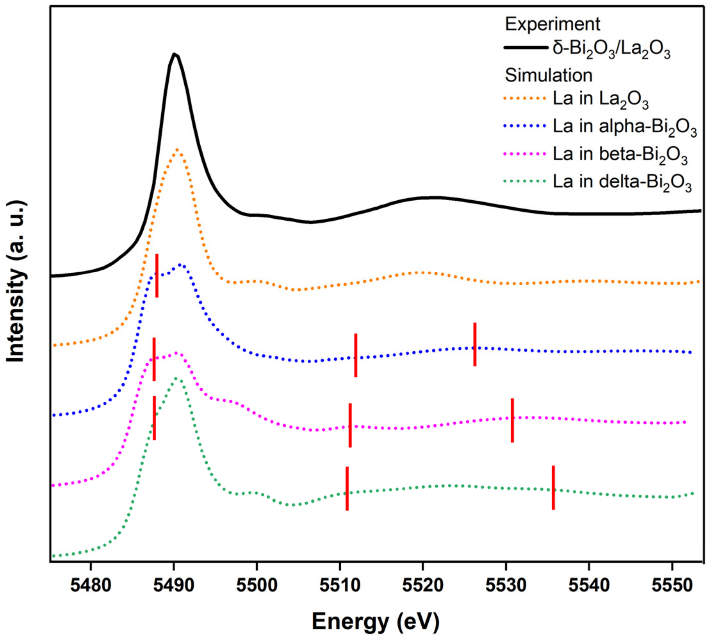

Fig. R4 | Comparison of the experimental La L_3-edge XANES spectrum of the δ-Bi_2O_3/La_2O_3 sample with simulated spectra for various Bi_2O_3 phases and La-doped La_2O_3 models.

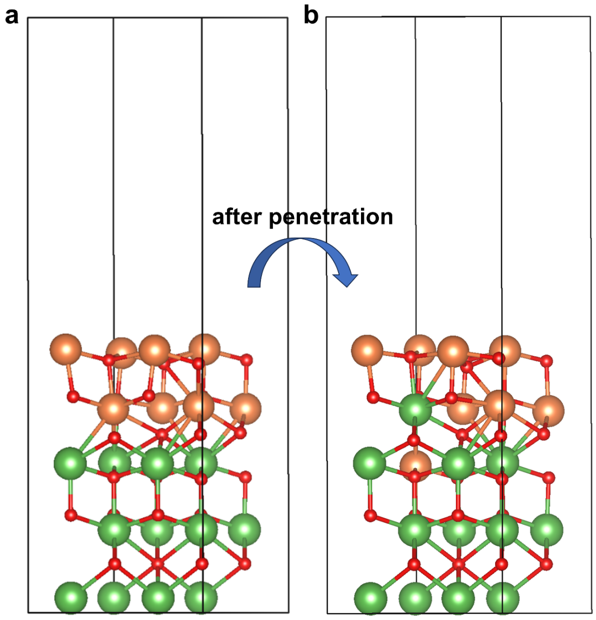

Fig. R5 | The optimized structure of the heterojunction obtained by DFT calculation before (a) and after (b) the penetration (one Bi atom and one La atom) process at the interface. The system energy of these two structures is -350.89 and -350.64 eV, respectively.

Second, in our original submission, we demonstrated that the lattice contraction in the δ-Bi_2O_3 layer results from the spontaneous migration of oxygen atoms from surface to subsurface sites (see Fig. 5a), rather than from La doping. As shown in **Fig. R6a**, there are three O layers (each with 3 oxygen atoms) in the δ-Bi_2O_3 phase, with Bi atoms at the bottom and O atoms at the top. For the La_2O_3 support, there are four O atoms at the surface. The initial structure was then optimized using DFT method and the final structure was shown in **Fig. R6b**. Compared to the initial structure, structural relaxation of the heterojunction leads to pronounced atomic rearrangements, particularly among the oxygen atoms in the δ-Bi_2O_3 phase. Specifically, one oxygen atom in the 2nd O layer migrates into the 3rd O layer, and simultaneously, three oxygen atoms in the 1st layer migrate into the 2nd layer. This rearrangement results in the contraction of the supported δ-Bi_2O_3 phase. Moreover, the structural relaxation leads to the formation of Bi−O−La and Bi−La bonds at the interface, reflecting strong interfacial coupling between the two phases. The *p-d* hybridization between Bi and La atoms (Supplementary Fig. 71) and the contribution of Bi–La bonds revealed by the crystal orbital bond index (COBI) analysis (Supplementary Fig. 72) further support a strong interfacial interaction. Additionally, the newly supplemented charge density difference plot (**Fig. R7**) show a charge accumulation in the La_2O_3 phase and a charge depletion in the δ-Bi_2O_3 phase, suggesting an obvious charge transfer and redistribution across the δ-Bi_2O_3/La_2O_3 interfaces. In the δ-Bi_2O_3 region of the δ-Bi_2O_3/La_2O_3 heterostructure, the average Bader charge on Bi atoms increases from +1.66 |e| in the topmost

(surface) layer to +2.03 |e| in the subsurface (interface) layer, indicative of interfacial charge redistribution associated with a strong oxide–support interaction. This electrostatic gradient drives the spontaneous migration of surface oxygen atoms toward the more positively charged Bi sites in the subsurface layer. Overall, these results support the strong metal oxide–support interaction (SMOSI) induced by the lattice-mismatched interface between the anchored δ-Bi_2O_3 particles and the La_2O_3 support, as reasonably reflected by the DFT-optimized heterojunction model.

Figs R6 and R7 have been added as Supplementary Figs. 68 and 70 in the revised Supporting Information, respectively.

Revised parts in the manuscript:

Page 16, lines 521–531

As shown in Supplementary Fig. 68a, there are three O layers (each with 3 oxygen atoms) in the δ-Bi_2O_3 phase, with Bi atoms at the bottom and O atoms at the top. For the La_2O_3 support, there are four O atoms at the surface. The initial structure was then optimized using DFT method and the final heterojunction structure was shown in Fig. 5a and Supplementary Fig. 68b. Compared to the initial structure, the structural relaxation due to the strong interfacial interactions leads to significant atomic rearrangements, particularly among the oxygen atoms in the top two Bi_2O_3 layer. Specifically, one oxygen atom in the second O layer migrates into the third O layer, and simultaneously, three oxygen atoms in the first layer migrate into the second layer. The resulting top layer features a 4Bi-5O moiety, while the second layer possesses a 5O-4Bi-4O structure. This migration of oxygen atoms from the surface to the subsurface induces a notable lattice contraction in the Bi_2O_3 layers.

Pages 16–17, lines 556–563

The charge density difference plot shows electron accumulation in the La_2O_3 phase and electron depletion in the δ-Bi_2O_3 phase (Supplementary Fig. 70), demonstrating an obvious interfacial charge transfer and redistribution between the two phases. In δ-Bi_2O_3 region of the δ-Bi_2O_3/La_2O_3 heterostructure, the average Bader charge on Bi atoms increases from +1.66 |e| in the topmost (surface) layer to +2.03 |e| in the subsurface (interface) layer, indicating more electrons transfer to neighboring O and La atoms. This electrostatic gradient facilitates the migration of surface oxygen atoms toward the more positively charged Bi sites in the subsurface (interface) layer.

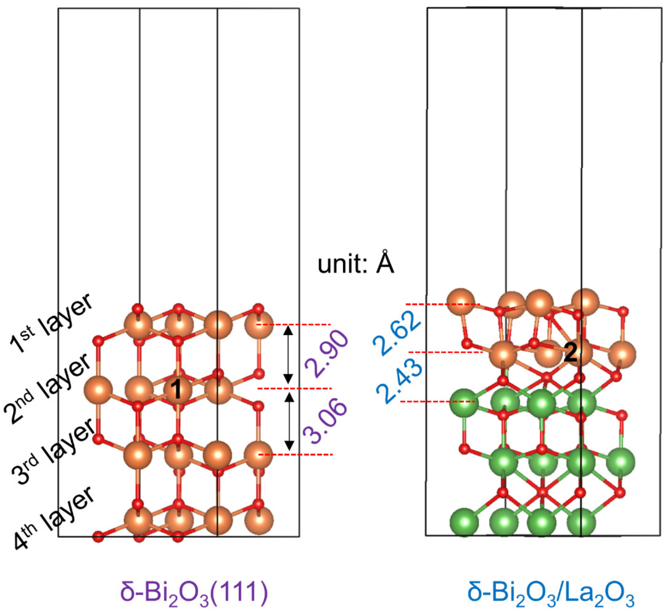

Fig. 5a | Optimized catalyst models for δ-Bi_2O_3(111) and δ-Bi_2O_3/La_2O_3 heterojunction.

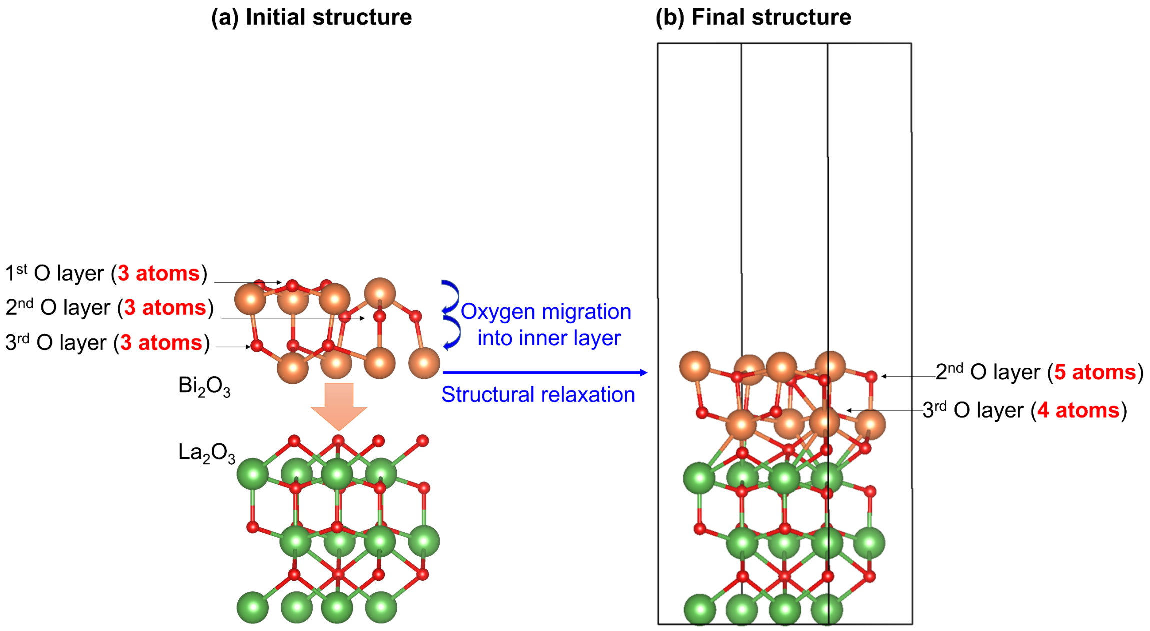

Fig. R6 | Scheme of the formation of δ-Bi_2O_3/La_2O_3 model from (a) initial structure to (b) final structure.

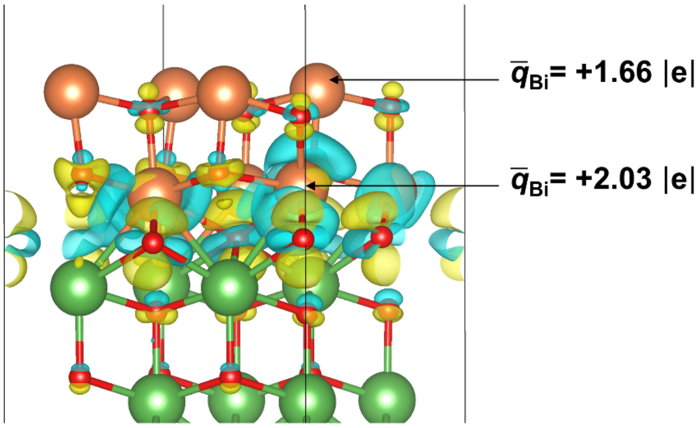

Fig. R7 | The charge density difference plot ($\rho_{CDD} = \rho_{heterojunction} - \rho_{Bi2O3} - \rho_{La2O3}$) is also given for the obtained heterojunction model, with an isosurface value of 0.005 e/Å^3.

(2) There are error bars in Figure S20 while no error bars in Figure 2. The authors said, "7% δ-Bi_2O_3/La_2O_3 exhibits a FE_{HCOO-} of ≥ 90% over a wide potential, which is superior to that of pure δ-Bi_2O_3 powder with a FE_{HCOO-} of 79.3% at –1.05 V_{RHE}." However, the FE_{HCOO-} over pure δ-Bi_2O_3 can also reach around ~90% at –0.95 V_{RHE} (Figure 2a). The comparison is unreasonable.

[Reply]: We thank the reviewer for this valuable comment, which helps clarify the performance metrics and avoids potential confusion. To enable a fairer comparison, the original statement on page 13, "...which is superior to that of pure δ-Bi_2O_3 powder with a FE_{HCOO-} of 79.3% at –1.05 V_{RHE}..." has been revised to "...which is superior to that of pure Bi_2O_3 powder with a FE_{HCOO-} of 88.5% at –0.95 V vs. RHE..." in the revised manuscript. Additionally, we have added the error bars in the revised Figs. 2a, c, e, which represent the standard deviations derived from three independent measurements. We are sorry for such negligence and checked these issues in the revised manuscript and Supporting Information. Fig. 2 has been updated in the revised main text.

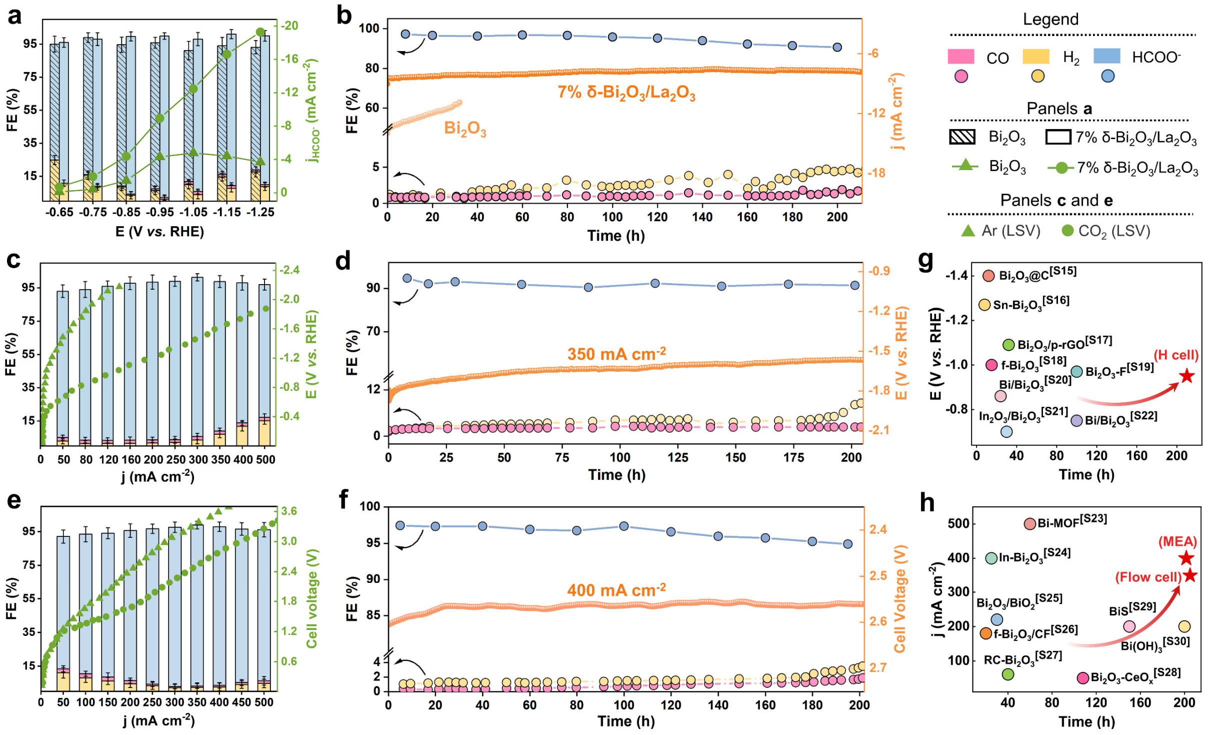

Fig. 2 | Electrocatalytic CO_2RR Performances. H-type cell: (a) FEs of formate and H_2 and relevant $j_{formate}$ on 7% δ-Bi_2O_3/La_2O_3 and pure Bi_2O_3 recorded under different potentials; (b) Durability test of 7% δ-Bi_2O_3/La_2O_3 and pure Bi_2O_3 at the applied potential of −0.95 V vs. RHE. Flow-cell: (c) LSV curves of 7% δ-Bi_2O_3/La_2O_3 under CO_2 and Ar atmospheres, and FEs of various products on 7% δ-Bi_2O_3/La_2O_3 at varying current density; (d) Durability test of 7% δ-Bi_2O_3/La_2O_3 at the current density of 350 mA cm^{-2}. MEA-electrolyzer: (e) LSV curves of 7% δ-Bi_2O_3/La_2O_3 under CO_2 and Ar atmospheres, and FEs of various products on 7% δ-Bi_2O_3/La_2O_3 at different current densities; (f) Durability test and corresponding product distributions of continuous 200 h electrolysis under constant total current density of 400 mA cm^{-2}. (g, h) Comparison of the CO_2RR to formate performance in aqueous system. Detailed data are provided in Supplementary Table 4. The error bars represent standard deviations from three independent measurements.

(3) The authors synthesized δ-Bi_2O_3/La_2O_3 catalysts with different Bi content from 0.8, 2.7 to 7.1 and considered low-density δ-Bi_2O_3 sites cannot effectively promote the reaction. So this work focuses on a 7% δ-Bi_2O_3/La_2O_3 sample. In fact, we don't know if the catalysts with more content of Bi can show superior performance than the 7.1% content one. Maybe a higher Bi content catalyst is needed to study the relatively appropriate δ-Bi_2O_3/La_2O_3 ratio. The work shows a current density of 500 mA cm^{-2} at "only" −1.85 V_{RHE}. However, there are some formate production works (The Innovation, 2025, 6: 100844; Angew. Chem. Int. Ed. 2025, 137, e202423370; Angew. Chem. Int. Ed. 2025, 64, e202507422.) that exhibit much lower potential to reach 500 mA cm^{-2}. This work shows a good performance of more than 200 h at 350 mA cm^{-2} in the flow cell which is different from the results in Figure 2 h.

[Reply]: We sincerely appreciate these insightful comments. As suggested, we have prepared two additional δ-Bi_2O_3/La_2O_3 catalysts with higher Bi loadings (9.6 and 15.2 at.%, determined

by ICP-AES), denoted as 10% and 15% δ-Bi_2O_3/La_2O_3, respectively, and evaluated their CO_2 electroreduction performance in a flow-cell electrolyzer. As displayed in the supplementary aberration-corrected HAADF-STEM images (**Fig. R8**), the sub-2 nm, highly active δ-Bi_2O_3 particles begin to agglomerate into larger particles (~8 nm) with increasing Bi loading. This agglomeration reduces the density of highly active sites and diminishes the metal–support interface, both of which are crucial for catalytic activity and stability. As shown in **Fig. R9**, the 10% and 15% δ-Bi_2O_3/La_2O_3 catalysts exhibit lower overall formate Faradaic efficiency (FE_{HCOO^-}) and poorer operational stability than the 7% δ-Bi_2O_3/La_2O_3, suggesting that a 7% Bi loading is near the optimal. Specifically, the 10% δ-Bi_2O_3/La_2O_3 catalyst delivers a formate Faradaic efficiency of ~91.5% and maintains stable performance for ~175 h, whereas the 15% sample exhibits a lower efficiency (~89.3%) and deactivates after ~154 h. Furthermore, we conducted *in-situ* Raman and FT-IR spectroscopy on the 10% and 15% δ-Bi_2O_3/La_2O_3 samples to explore the origin of the performance degradation associated with increased Bi loading. *In-situ* Raman spectroscopy reveal that the Bi−O (619 cm^{-1}) and Bi−O−La (390 cm^{-1}) signals in the high-loading (10% and 15%) samples exhibits poorer stability compared to that in the 7% sample, as evidenced by a pronounced positive shift in the onset potential for its reduction to metallic Bi. As shown in **Fig. R10**, two new characteristic peaks at ~70 and ~90 cm^{-1}, assigned to the E_g and A_{1g} vibrational modes of metallic Bi^0, appear at approximately −1.45 V vs. RHE for 10% δ-Bi_2O_3/La_2O_3 and at −1.25 vs. RHE for 15% δ-Bi_2O_3/La_2O_3, respectively. Accordingly, the potentiodynamic ATR-SEIRA spectra reveal that the signal intensity of the ν_s(OCO) band of bidentate *HCOO intermediate at ~1310 cm^{-1} on the 10% and 15% δ-Bi_2O_3/La_2O_3 samples shows a pronounced decreasing trend at more negative potentials (**Figs. R11, R12**). In contrast, the ν_s(OCO) signal on the 7% δ-Bi_2O_3/La_2O_3 sample remains largely constant over the potential range of −0.85 to −1.15 V vs. RHE (Supplementary Fig. 63). Therefore, 7% represents an optimal balance between active site density and dispersion. We further acquired time-resolved ATR-SEIRA spectra to gain deeper insight into the long-term CO_2RR electrolysis. At a fixed potential of −0.95 vs. RHE, the contour plots show that the ν_s(OCO) bands of the adsorbed bidentate *HCOO intermediate on the 10% and 15% δ-Bi_2O_3/La_2O_3 samples gradually decrease in intensity over the 900-min CO_2RR (**Fig. R13**). The above results underscore the unique advantage of the highly dispersed sub-2 nm δ-Bi_2O_3 in the optimal 7% δ-Bi_2O_3/La_2O_3, which enables stable electrochemical CO_2 reduction to formate.

Additionally, we appreciate the reviewer's meticulous observations about the discrepancy between the CO_2RR performance of 7% δ-Bi_2O_3/La_2O_3 in Figs. 2d, 2h. In fact, the stability data summarized in the original Fig. 2h corresponds to the 3% δ-Bi_2O_3/La_2O_3 catalyst, as shown in Supplementary Fig. 41. We apologize for the misunderstanding caused by our negligence, and

have provided the corrected stability data for the 7% δ-Bi_2O_3/La_2O_3 in the revised Fig. 2h. With your reminder, we rechecked such details and issues in the revised manuscript and SI. Finally, thank you for sharing the recent literature on CO_2RR to formate. We have removed the words 'up to' and 'only' from the description: "*δ-Bi_2O_3/La_2O_3 achieves a current density of 500 mA cm^{-2} at −1.85V vs. RHE.*" The three recent studies (*The Innovation*, 2025, 6, 100844; *Angew. Chem. Int. Ed.* 2025, 137, e202423370; *Angew. Chem. Int. Ed.* 2025, 64, e202507422.) have been cited as references [31, 32, 33] in the revised manuscript.

Figs R8, R9, R10a, R11, R12 have been added as Supplementary Figs. 12, 42, 53, 62, 63 in the revised Supporting Information, respectively.

Revised parts in the manuscript:

Pages 3–4, lines 125–130

In the 10% and 15% Bi_2O_3/La_2O_3 samples, the highly active sub-2 nm Bi_2O_3 particles begin to agglomerate into larger particles (~8 nm) as the Bi loading increases, resulting in a coexistence of sub-2 nm and ~8 nm particles. This agglomeration reduces the density of highly active sub-nanoparticles and may diminish the Bi_2O_3/La_2O_3 interfacial area, both of which are critical for improving catalytic activity and stability.

Page 8, lines 281–287

..while the 10% and 15% δ-Bi_2O_3/La_2O_3 catalysts exhibit lower formate Faradaic efficiency and poorer operational stability due to the aggregation of δ-Bi_2O_3 sub-nanoparticles (Supplementary Fig. 42). Specifically, the 10% δ-Bi_2O_3/La_2O_3 catalyst delivers a formate Faradaic efficiency of ~91.5% and maintains stable performance for ~175 h, whereas the 15% sample exhibits a lower efficiency (~89.3%) and deactivates after ~154 h. These results suggest that a 7% Bi loading may represent an optimal balance between active site density and dispersion.

Page 12, lines 401–407

Notably, in the high-loading (10% and 15%) samples, the Bi–O and Bi–O–La signals could not be maintained over such a wide potential window. As shown in Fig. 4c and Supplementary Fig. 53, the characteristic peaks of metallic Bi−Bi at ~70 and ~95 cm^{-1} appeared at −1.45 V vs. RHE for the 10% δ-Bi_2O_3/La_2O_3 sample and at −1.25 V vs. RHE for the 15% δ-Bi_2O_3/La_2O_3 sample, respectively. The Raman results again highlight the distinct advantage of well-dispersed sub-2 nm δ-Bi_2O_3 particles in the 7% δ-Bi_2O_3/La_2O_3 catalyst in enhancing the SMOSI effect.

Page 13, lines 461–464

Notably, the signal intensity of the v_s(OCO) band on the 10% and 15% δ-Bi_2O_3/La_2O_3 samples

shows a pronounced decreasing trend at more negative potentials (Supplementary Figs. 62, 63), indicating that the agglomeration of sub-nanoparticles induced by high Bi loading reduces the catalytic stability.

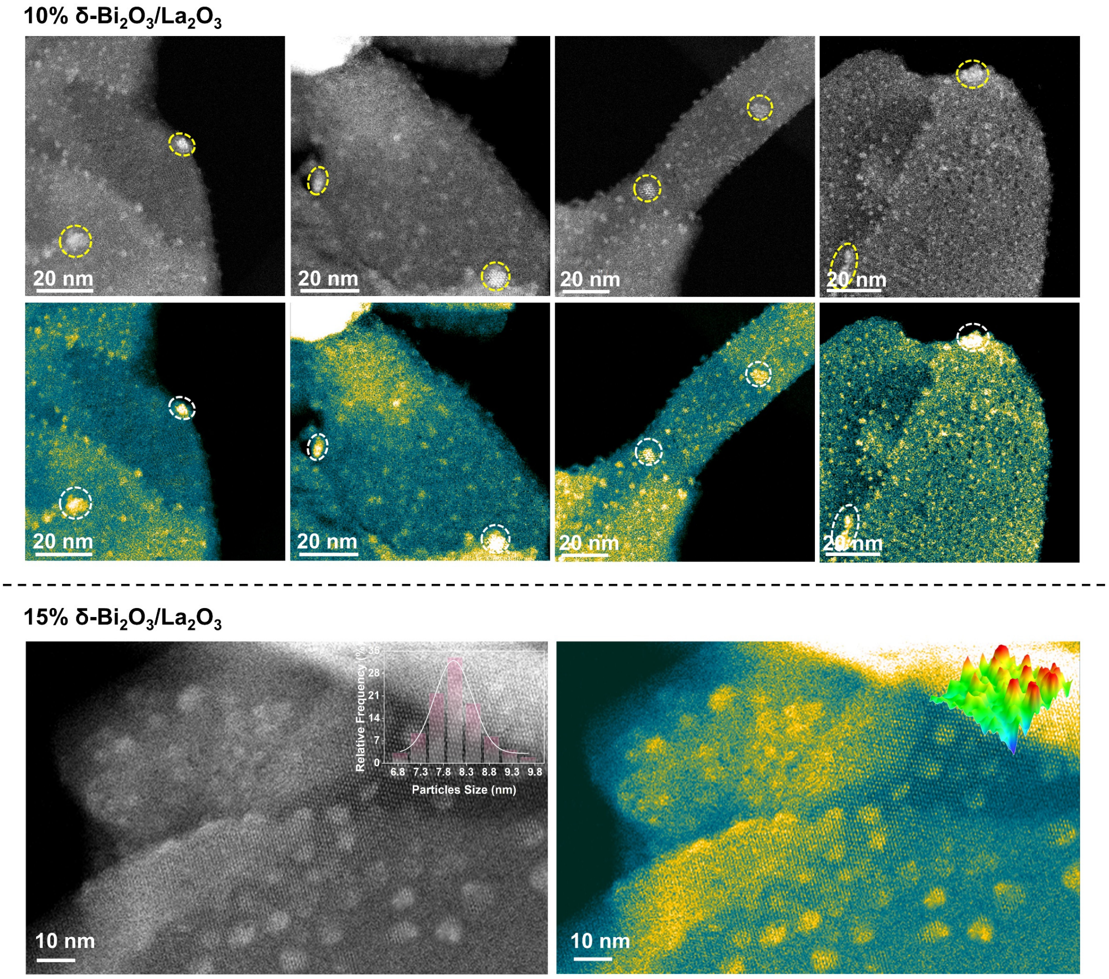

Fig. R8 | Aberration-corrected HAADF-STEM images as well as the corresponding elemental distribution intensity maps of 10% δ-Bi_2O_3/La_2O_3 and 15% δ-Bi_2O_3/La_2O_3, the inset is corresponding size distribution.

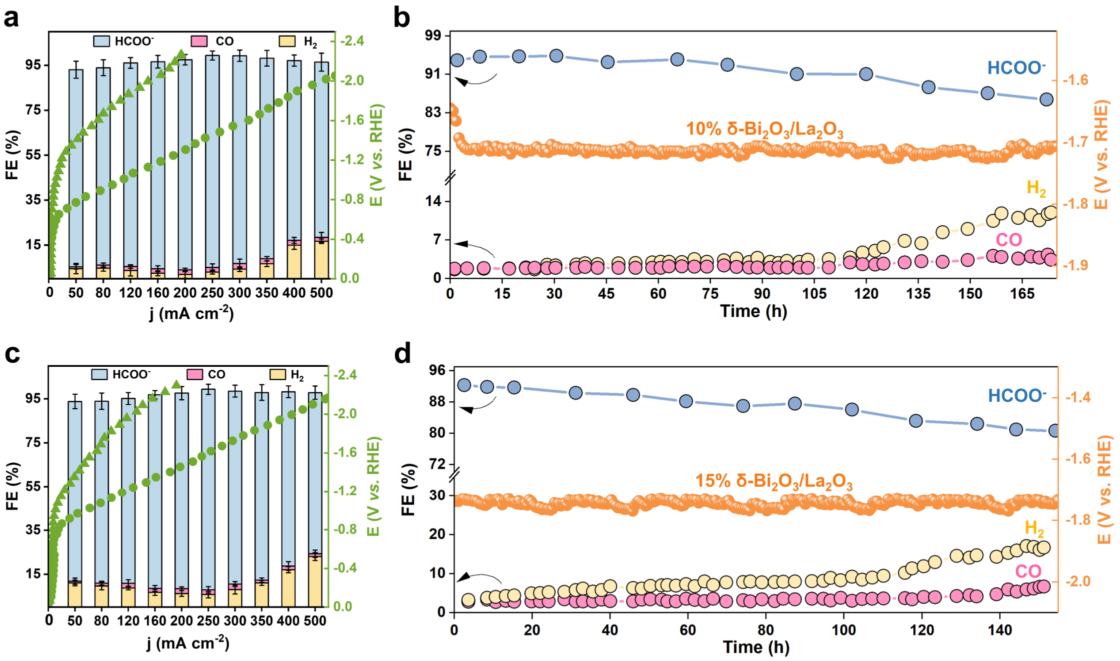

Fig. R9 | LSV curves under CO_2-saturated (circular) and Ar-saturated (triangle) 0.5 M $KHCO_3$ solution, and corresponding FEs of various products as a function of current density for (a) 10% δ-Bi_2O_3/La_2O_3 and (c) 15% δ-Bi_2O_3/La_2O_3 catalysts. Long-term chronopotentiometric curves and corresponding FEs of (b) 10% δ-Bi_2O_3/La_2O_3 and (d) 15% δ-Bi_2O_3/La_2O_3 at 350 mA cm^{-2} in a flow cell.

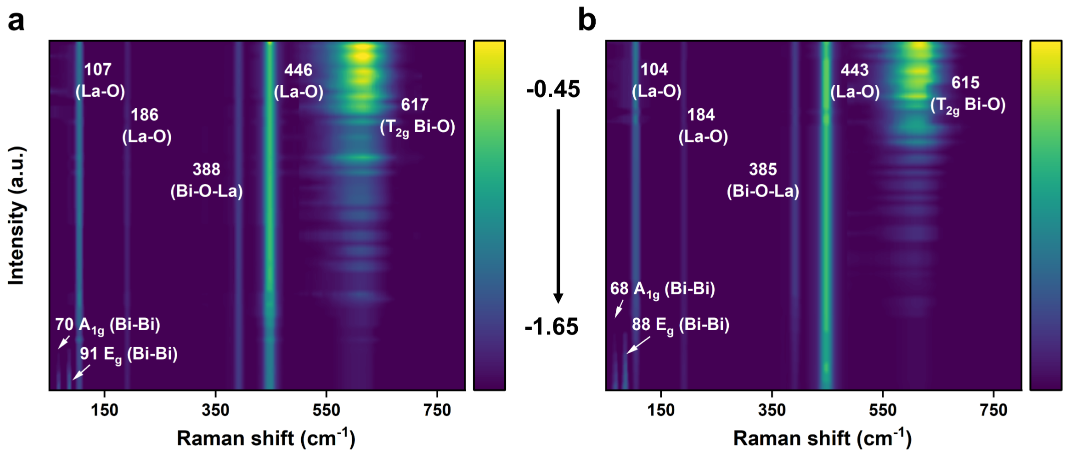

Fig. R10 | In-situ Raman spectra monitoring the surface variation of (a) 10% δ-Bi_2O_3/La_2O_3 and (b) 15% δ-Bi_2O_3/La_2O_3 under different applied potentials.

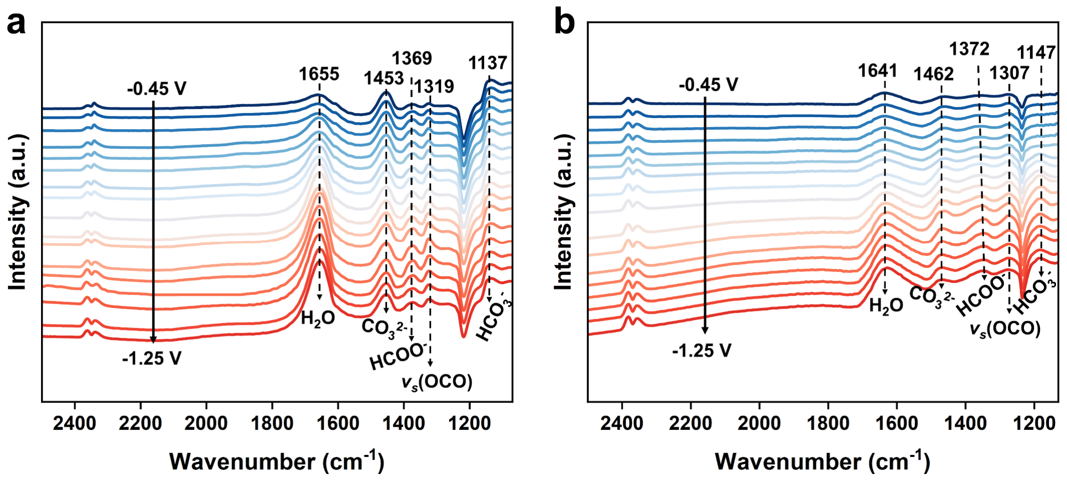

Fig. R11 | *In-situ* ATR-SEIRA spectra of (a) 10% and (b) 15% δ-Bi_2O_3/La_2O_3 recorded at different applied potentials in CO_2-saturated 0.5 M $KHCO_3$ solution, with the spectrum at −0.25 V vs. RHE as the reference.

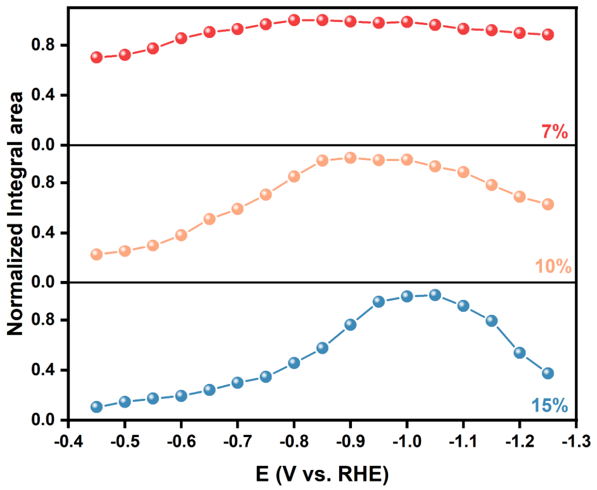

Fig. R12 | Potential-dependent integrated intensities of the ν_s(OCO) band for the 7%, 10%, and 15% δ-Bi_2O_3/La_2O_3 samples.

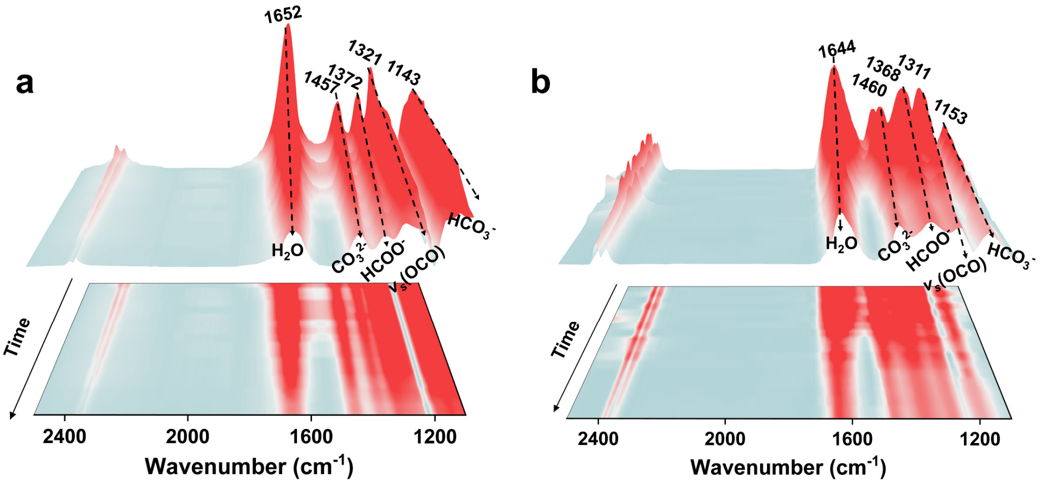

Fig. R13 | Time-evolved ATR-SEIRAS color contour map for (a) 10% and (b) 15% δ-Bi_2O_3/La_2O_3 at a fixed potential of −0.95 V vs. RHE.

(4) The authors said, "The characteristic peak for adsorbed intermediate $*CO_2^{\cdot-}$ at ~1262 cm^{-1} (Figure 4f)." However, there are interference peaks at 1100~1300 cm^{-1} owing to the Si-O signals (Anal. Chem. 2024, 96, 10111; Phys. B: 2000, 292, 286.) and the labeled peaks (Si-O-Si) by the authors at ~1200 cm^{-1} in the absorbance spectra are not clear (Figure 4f). Actually, we don't consider that there are absorbance peaks at ~1200 cm^{-1}. In addition, there are no reference experiments, such as carbon and Au film ATR-SEIRA spectra. Hence, we can't determine which peak is corresponding to $HCOO^-$.

[Reply]: We thank the reviewer for raising these important concerns regarding the assignment of vibrational bands in our ATR-SEIRA spectra. First, we fully acknowledge that the spectral region between 1100 and 1300 cm^{-1} can be overlapped by contributions from the Si-based internal reflection element (IRE), particularly Si–O stretching vibrations (~1200–1250 cm^{-1}), as pointed out by the reviewer. We observed a broad band at ~1262 cm^{-1}, consistent with the $*CO_2^{\cdot-}$ species reported in the literature (*ACS Energy Lett.* 2021, 6, 3352; *Angew. Chem. Int. Ed.* 2021, 60, 7382; *Adv. Energy Mater.* 2023, 13, 2203506; *Adv. Mater.* 2019, *31*, 1808135). However, we acknowledge that this assignment involves some uncertainty due to the very weak intensity of the band and potential spectral overlap (see Fig. 4f, and the newly added Fig. R11). Therefore, to avoid overinterpretation, we have revised the manuscript to remove the attribution of the ~1262 cm^{-1} band to $*CO_2^{\cdot-}$ intermediate. Second, the Si–O band appears broad and unresolved near 1200 cm^{-1} in Fig. 4f, primarily because the displayed wavenumber range (1000–1550 cm^{-1}) compresses the spectral features. The full spectrum (1000–2500 cm^{-1}) in the originally submitted Supplementary Fig. 45 clearly shows a sharp peak at ~1200 cm^{-1} for the 7% δ-Bi_2O_3/La_2O_3 electrode, assigned to Si–O stretching vibrations. Finally, per your suggestion, we performed control experiments using a bare Au-coated IRE (without catalyst,

Fig. R14a) and pure La_2O_3 sample on the Au-coated IRE (**Fig. R14b**) under identical CO_2RR conditions. No characteristic peaks for formate-related species were observed in the ATR-SEIRA spectra (Fig. R14), specifically neither the ν_s(OCO) stretching vibration of bidentate *HCOO at ~1310 cm^{-1} nor the band for dissolved $HCOO^-$ at ~1360 cm^{-1}. To further assign the observed IR bands to CO_2RR intermediates, we performed a ^{13}C isotope labeling experiment ($^{13}CO_2$, 99.0 atom%, Sigma-Aldrich; **Fig. R15a**). Using a $^{13}CO_2$-saturated 0.5 M $KHCO_3$ electrolyte, the spectra for the 7% δ-Bi_2O_3/La_2O_3 electrode show clear red shifts (**Fig. R15b**) for the bands at 1266 cm^{-1} (ν_s(OCO)) and 1329 cm^{-1} ($HCOO^-$). The red shifts in vibrational frequencies align with the theoretical $^{13}C/^{12}C$ isotopic effect (Equation 1), further confirming the assignment of these IR bands.

$$\frac{1}{\lambda}\sqrt{\frac{\mu^*}{\mu}} = \frac{1}{\lambda^*} \qquad \text{Equation 1}$$

where $1/\lambda$ and $1/\lambda^*$, and μ and μ^* are the wavenumbers of vibrational modes and reduced masses of the unlabeled and labeled molecules, respectively (*Nat. Catal.* 2024, 7, 807; *J. Am. Chem. Soc*. 2017, 139, 3774; *Chem. Commun*., 1987, 10, 728; *Angew. Chem. Int. Ed*. 2017, 56, 3621).

Figs R14, R15a have been added as Supplementary Figs. 60, 61 in the revised Supporting Information, Fig. R15b has been added as Fig. 4g in the revised main text, respectively.

Revised parts in the manuscript:

Page 13, lines 446–456

To confirm that the observed IR peaks originate from CO_2 reduction, we carried out control experiments using a bare Au-coated Si-based internal reflection element (IRE) (Supplementary Fig. 60a) and pure La_2O_3 sample on the Au-coated IRE (Supplementary Fig. 60b) under identical CO_2RR conditions. No characteristic peaks for formate-related species at ~1310 cm^{-1} and ~1360 cm^{-1} were observed in the ATR-SEIRA spectra. Moreover, we performed a ^{13}C isotope labeling experiment ($^{13}CO_2$, 99.0 atom%, Sigma-Aldrich; Supplementary Fig. 61). Using a $^{13}CO_2$-saturated 0.5 M $KHCO_3$ electrolyte, the spectra for the 7% δ-Bi_2O_3/La_2O_3 electrode show obvious red shifts (Fig. 4g) for the IR bands at 1266 cm^{-1} (ν_s(OCO)) and 1329 cm^{-1} ($HCOO^-$). The red shifts in vibrational frequency align with the theoretical $^{13}C/^{12}C$ isotopic effect, further confirming the assignment of these IR bands.[47, 48]

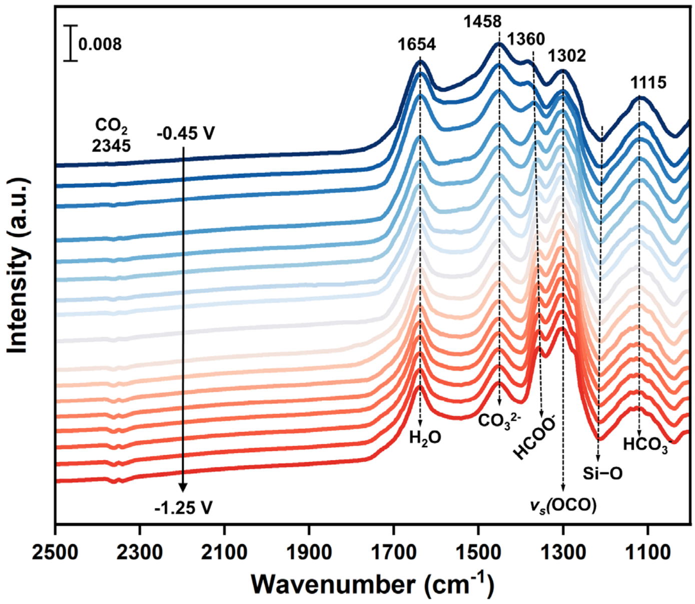

Supplementary Fig. 45 | *In-situ* ATR-SEIRA spectra of 7% δ-Bi_2O_3/La_2O_3 electrode in the range of 1000–2500 cm^{-1}.

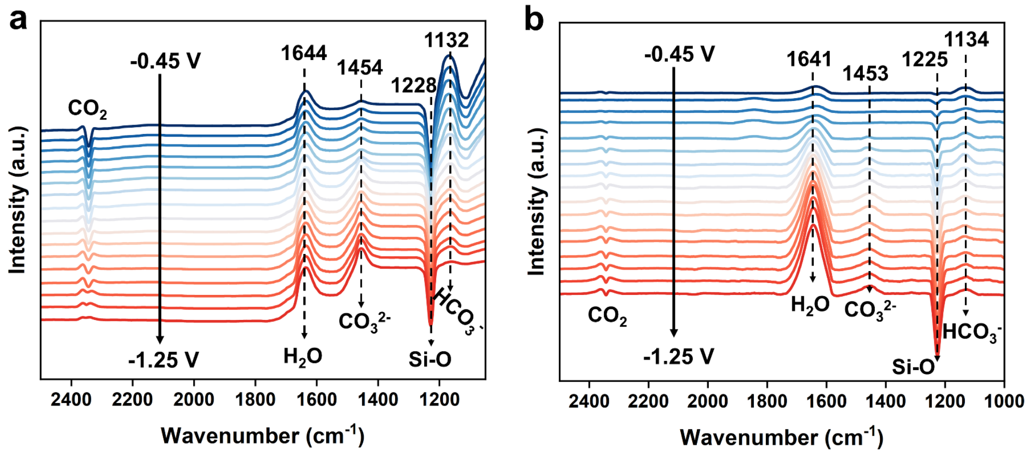

Fig. R14 | In situ ATR-SEIRAS of control samples. (a) Bare Au-coated IRE and (b) pure La_2O_3 on Au underfilm. Spectra were collected at different applied potentials in CO_2-saturated 0.5 M $KHCO_3$ solution, using a reference spectrum at −0.25 V vs. RHE.

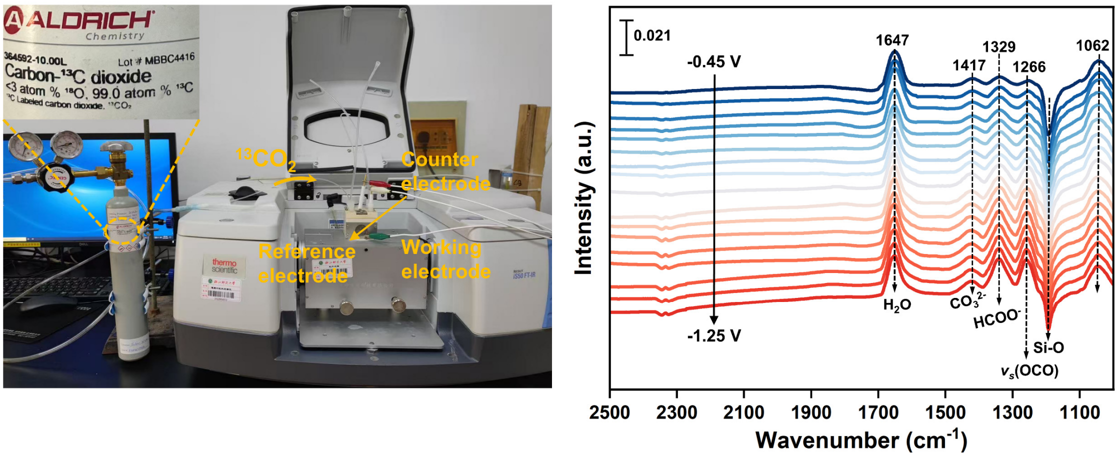

Fig. R15 | Isotope-labeling ATR-SEIRAS experiment with $^{13}CO_2$. (a) Photograph of the electrochemical ATR-SEIRAS cell configured for $^{13}CO_2$ isotope labeling. (b) In situ ATR-SEIRA spectra of the 7% δ-Bi_2O_3/La_2O_3 electrode collected at different applied potentials in $^{13}CO_2$-saturated 0.5 M $KHCO_3$ electrolyte, using a reference spectrum at −0.25 V vs. RHE.

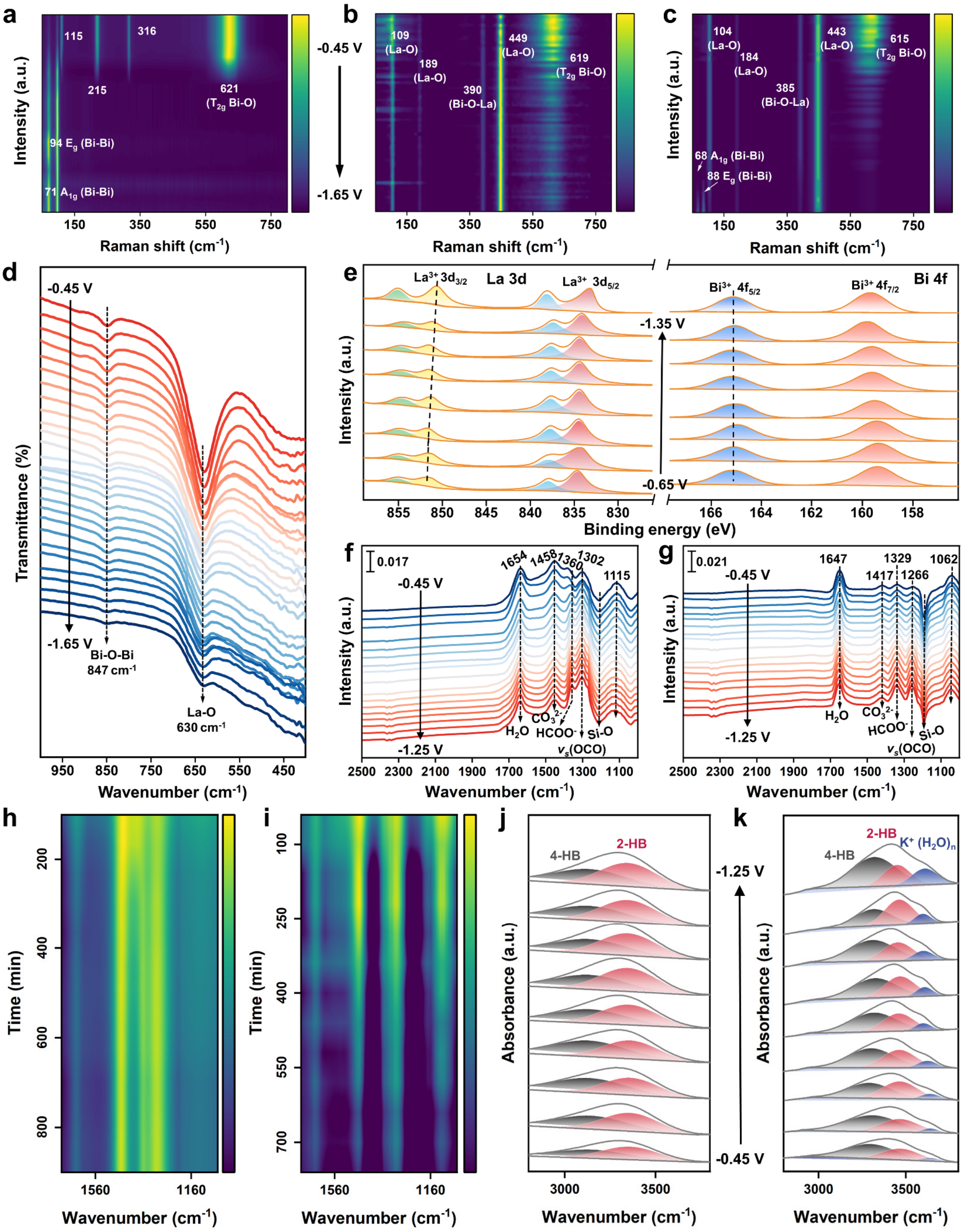

Fig. 4 | Structural evolution and intermediates detection during the CO_2RR process. *In-situ* Raman spectra monitoring the structural variation of (a) Bi_2O_3, (b) 7% δ-Bi_2O_3/La_2O_3 and (c) 15% δ-Bi_2O_3/La_2O_3 under different applied potentials. (d) Potential-dependent FT-IR spectra of 7% δ-Bi_2O_3/La_2O_3. (e) Potential-dependent quasi-in situ XPS measurement for 7% δ-Bi_2O_3/La_2O_3 electrode under electrolysis from −0.65 to −1.35 V vs. RHE. *In-situ* ATR-SEIRA spectra of 7% δ-Bi_2O_3/La_2O_3 recorded at different applied potentials in (f) CO_2-saturated and (g) isotope labeling $^{13}CO_2$-saturated 0.5 M $KHCO_3$ solution, using a reference spectrum at −0.25 V vs. RHE. Time-evolved ATR-SEIRAS color contour map for (h) 7% δ-Bi_2O_3/La_2O_3 and (i) Bi_2O_3 at −0.95 V vs. RHE. Gaussian fits of three O−H stretching modes for (j) Bi_2O_3 and (k) 7% δ-Bi_2O_3/La_2O_3 based on the ATR-SEIRA spectra.

(5) The authors set up Bi_2O_3(111)/La_2O_3(001) heterojunction as the model for DFT calculations. However, the authors also consider that Bi^{3+} enters into the La_2O_3 lattice and partially replaces La^{3+} to form Bi−O−La bonds. It is the main reason that leads to the average coordination (Bi-O) of Bi in the heterojunctions exceeding 6 (Table S3). We also consider that La enters Bi_2O_3 structures, too. Hence, the real models for DFT calculations should be composed by La-doped Bi_2O_3(111) and Bi-doped La_2O_3(001).

[Reply]: We thank the reviewer for raising these important concerns on the interdiffusion of Bi and La atoms and the DFT heterojunction model. First, to determine whether Bi is incorporated into the La_2O_3 lattice, we performed additional X-ray absorption spectroscopy measurements at the La L_3-edge. As shown in **Fig. R16**, the La absorption edge position in δ-Bi_2O_3/La_2O_3 is essentially identical to that of pure La_2O_3, indicating that Bi is not significantly incorporated into the La_2O_3 lattice. Theoretical XAS simulations are further conducted for multiple structural models—Bi-doped La_2O_3, undoped α-, β-, and δ-Bi_2O_3—to identify the local coordination environment of Bi in the δ-Bi_2O_3/La_2O_3 catalyst by comparing the simulated and experimental XANES spectra. As shown in **Fig. R17**, the simulated Bi L_3-edge XANES spectrum for Bi-doped La_2O_3 does not match the experimental spectrum of the δ-Bi_2O_3/La_2O_3 sample, whereas the simulated spectrum for undoped δ-Bi_2O_3 shows excellent agreement, indicating that Bi atom is not significantly incorporated into the La_2O_3 lattice. Moreover, the simulated Bi L_3-edge XANES spectra for both α-Bi_2O_3 and β-Bi_2O_3 do not match the experimental spectrum, also distinguishing that the Bi_2O_3 in the composite predominantly exists as the δ-phase. Given that the ionic radius of La^{3+} (0.106 nm) is larger than that of Bi^{3+} (0.096 nm), incorporation of La into the δ-Bi_2O_3 lattice would be expected to induce lattice expansion. However, a lattice contraction is observed in the δ-Bi_2O_3 layer of the δ-Bi_2O_3/La_2O_3 heterostructure, suggesting that La doping into δ-Bi_2O_3 is unlikely. To this end, we further performed La L_3-edge XANES simulations based on the structural models—La-doped α-Bi_2O_3, La-doped β-Bi_2O_3, La-doped δ-Bi_2O_3, and undoped La_2O_3—to further identify the local coordination environment of La in the δ-Bi_2O_3/La_2O_3 catalyst. As shown in **Fig. R18**, the simulated La L_3-edge XANES spectra for La-doped α-, β-, and δ-Bi_2O_3 show poor agreement with the experimental spectrum of the δ-Bi_2O_3/La_2O_3 sample, whereas the simulated spectrum for pristine La_2O_3 closely matches the experimental data, indicating negligible incorporation of La into the δ-Bi_2O_3 lattice. However, it should be noted that the realistic heterojunction structure is much more complicated, and the interdiffusion of Bi and La atoms at the interface may occur. Per your suggestions, we further constructed a δ-Bi_2O_3/La_2O_3 heterostructure with an intermixed interface, in which one Bi atom substitutes into the La_2O_3 lattice and one La atom incorporates into the δ-Bi_2O_3 lattice (**Fig. R19**). By comparing the system energies of different interface configurations, we find that the

model after penetration is 0.25 eV higher in energy than the non-intermixed δ-Bi_2O_3/La_2O_3 interface, implying that the latter is thermodynamically more stable. Based on the above results, we have removed the previous inaccurate statement: "*The rational for this finding is the similar ionic radii of La^{3+} (0.106 nm) and Bi^{3+} (0.096 nm), which allows Bi^{3+} to enter the La_2O_3 lattice and partially substitute La^{3+} to form Bi–O–La bonds*." Consequently, large-scale interdiffusion of Bi and La atoms is unlikely to occur in this work.

Additionally, we acknowledge that the construction of our DFT model was not sufficiently detailed in the original manuscript, and we have now provided a clearer description in the revised version. To build a reasonable heterojunction model, the δ-Bi_2O_3(111) layer was cut and loaded on the La_2O_3(001) surface. As shown in **Fig. R20a**, there are three O layers (each with 3 oxygen atoms) in the δ-Bi_2O_3 phase, with Bi atoms at the bottom and O atoms at the top. For the La_2O_3 support, there are four O atoms at the surface. The initial structure was then optimized using DFT method and the final structure was shown in **Fig. R20b**. Compared to the initial structure, structural relaxation of the heterojunction leads to pronounced atomic rearrangements, particularly among the oxygen atoms in the δ-Bi_2O_3 phase. Specifically, one oxygen atom in the 2nd O layer migrates into the 3rd O layer, and simultaneously, three oxygen atoms in the 1st layer migrate into the 2nd layer. This rearrangement results in the contraction of the supported δ-Bi_2O_3 phase. Moreover, the structural relaxation leads to the formation of Bi−O−La and Bi−La bonds at the interface, reflecting strong interfacial coupling between the two phases. The *p-d* hybridization between Bi and La atoms (Supplementary Fig. 70) and the contribution of Bi–La bonds revealed by the crystal orbital bond index (COBI) analysis (Supplementary Fig. 71) further support a strong interfacial interaction. Additionally, the newly supplemented charge density difference plot (**Fig. R21**) show a charge accumulation in the La_2O_3 phase and a charge depletion in the δ-Bi_2O_3 phase, suggesting an obvious charge transfer and redistribution across the δ-Bi_2O_3/La_2O_3 interfaces. In the δ-Bi_2O_3 region of the δ-Bi_2O_3/La_2O_3 heterostructure, the average Bader charge on Bi atoms increases from +1.66 |e| in the topmost (surface) layer to +2.03 |e| in the subsurface (interface) layer, indicative of interfacial charge redistribution associated with a strong oxide–support interaction. This electrostatic gradient drives the spontaneous migration of surface oxygen atoms toward the more positively charged Bi sites in the subsurface layer. Overall, the DFT-optimized heterojunction model in Fig. 5a (or Fig. R20b) reflects the strong interaction between the two phases and thus provides a plausible model for the experimentally synthesized catalyst.

Figs R16, R17, R18, R19, R20, R21 have been added as Supplementary Figs. 20, 23, 24, 69, 68, 70 in the revised Supporting Information, respectively.

Revised parts in the manuscript:

Page 5, lines 179–201

To further determine whether interfacial Bi and La atoms interdiffuse, we perform X-ray absorption spectroscopy (XAS) measurements and simulations. First, the La L_3-edge XANES spectra show that the La absorption edge position in the δ-Bi_2O_3/La_2O_3 is essentially identical to that of pure La_2O_3 (Supplementary Fig. 20), demonstrating that Bi is not significantly incorporated into the La_2O_3 lattice. Notably, the fitted La–O bond lengths are shorter in δ-Bi_2O_3/La_2O_3 than in pure La_2O_3, further suggesting a lattice contraction in the La_2O_3 support (Supplementary Fig. 21, 22 and Supplementary Table 1). We further carry out theoretical XAS simulations for Bi-doped La_2O_3 and pure α-, β-, and δ-Bi_2O_3 to identify the local coordination structure of Bi in δ-Bi_2O_3/La_2O_3 by comparing the simulated and experimental XANES spectra. As shown in Supplementary Fig.23, the simulated Bi L_3-edge XANES spectrum for Bi-doped La_2O_3 does not match the experimental spectrum of the δ-Bi_2O_3/La_2O_3 sample, whereas the simulated spectrum for pure δ-Bi_2O_3 shows excellent agreement, also verifying negligible incorporation of Bi into the La_2O_3 lattice. Besides, the simulated Bi L_3-edge XANES spectra for α-Bi_2O_3 and β-Bi_2O_3 do not match the experimental spectrum, further distinguishing that the Bi_2O_3 in the composite exists as the δ-phase. Subsequently, to determine whether La is incorporated into the δ-Bi_2O_3 lattice, we perform La L_3-edge XANES simulations based on these structural models—La-doped α-Bi_2O_3, La-doped β-Bi_2O_3, La-doped δ-Bi_2O_3, and undoped La_2O_3. As shown in Supplementary Fig. 24, the simulated La L_3-edge XANES spectra for La-doped α-, β-, and δ-Bi_2O_3 show poor agreement with the experimental spectrum of the δ-Bi_2O_3/La_2O_3 sample, whereas the simulated spectrum for pristine La_2O_3 closely matches the experimental data, also indicating that La is not significantly incorporated into the δ-Bi_2O_3 lattice. Consequently, large-scale interdiffusion of Bi and La atoms is unlikely to occur.

Page 16, lines 537–547

Notably, our previous XAS measurements and simulations in Supplementary Figs. 20-24 have demonstrated that the large-scale interdiffusion of Bi and La atoms is unlikely to occur spontaneously. However, it should be noted that the realistic heterojunction structure is much more complicated, and the penetration of Bi and La atoms at the interface may occur. We thus constructed a δ-Bi_2O_3/La_2O_3 heterostructure with an intermixed interface, in which one Bi atom substitutes into the La_2O_3 lattice and one La atom incorporates into the δ-Bi_2O_3 lattice (Supplementary Fig. 69). By comparing the system energies of different interface configurations, we find that the model after penetration is 0.25 eV higher in energy than the non-intermixed δ-Bi_2O_3/La_2O_3 interface, implying that the latter is thermodynamically more

stable. Consequently, the δ-Bi_2O_3/La_2O_3 model in Fig. 5a reasonably represents the experimental heterojunction catalyst.

Page 16, lines 521–531

As shown in Supplementary Fig. 68a, there are three O layers (each with 3 oxygen atoms) in the δ-Bi_2O_3 phase, with Bi atoms at the bottom and O atoms at the top. For the La_2O_3 support, there are four O atoms at the surface. The initial structure was then optimized using DFT method and the final heterojunction structure was shown in Fig. 5a and Supplementary Fig. 68b. Compared to the initial structure, the structural relaxation due to the strong interfacial interactions leads to significant atomic rearrangements, particularly among the oxygen atoms in the top two Bi_2O_3 layer. Specifically, one oxygen atom in the second O layer migrates into the third O layer, and simultaneously, three oxygen atoms in the first layer migrate into the second layer. The resulting top layer features a 4Bi-5O moiety, while the second layer possesses a 5O-4Bi-4O structure. This migration of oxygen atoms from the surface to the subsurface induces a notable lattice contraction in the Bi_2O_3 layers.

Pages 16–17, lines 556–563

The charge density difference plot shows electron accumulation in the La_2O_3 phase and electron depletion in the δ-Bi_2O_3 phase (Supplementary Fig. 70), demonstrating an obvious interfacial charge transfer and redistribution between the two phases. In δ-Bi_2O_3 region of the δ-Bi_2O_3/La_2O_3 heterostructure, the average Bader charge on Bi atoms increases from +1.66 |e| in the topmost (surface) layer to +2.03 |e| in the subsurface (interface) layer, indicating more electrons transfer to neighboring O and La atoms. This electrostatic gradient facilitates the migration of surface oxygen atoms toward the more positively charged Bi sites in the subsurface (interface) layer.

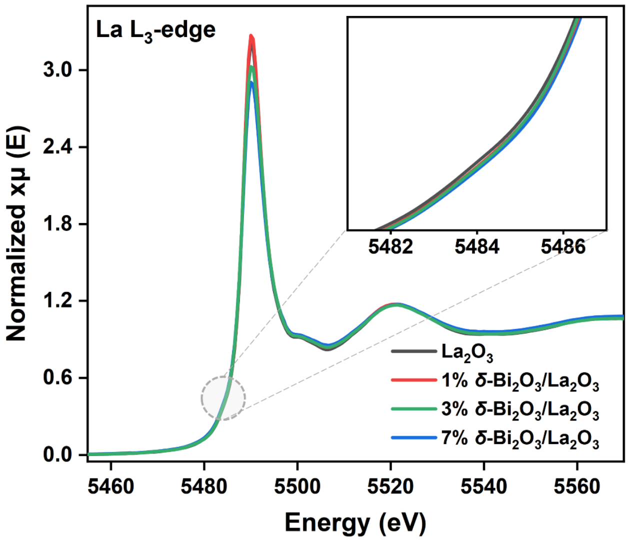

Fig. R16 | La L_3-edge XANES spectra over pure La_2O_3 and δ-Bi_2O_3/La_2O_3 samples.

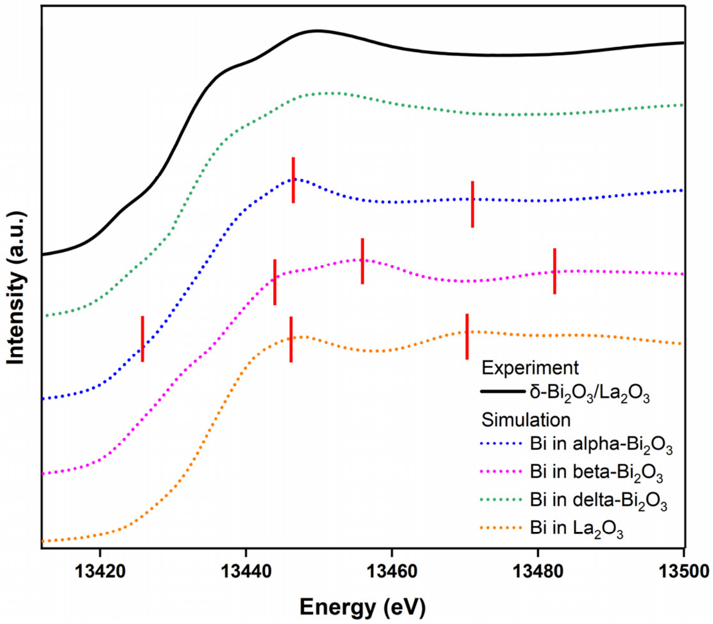

Fig. R17 | Comparison of the experimental Bi L_3-edge XANES spectrum of the Bi_2O_3/La_2O_3 sample with simulated spectra for various Bi_2O_3 phases and Bi-doped La_2O_3 models.

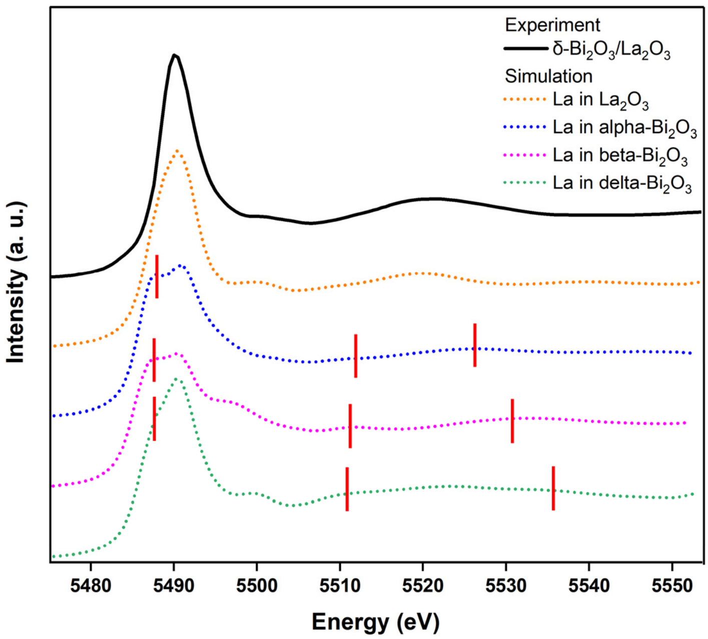

Fig. R18 | Comparison of the experimental La L_3-edge XANES spectrum of the δ-Bi_2O_3/La_2O_3 sample with simulated spectra for various La-doped Bi_2O_3 models and pristine La_2O_3.

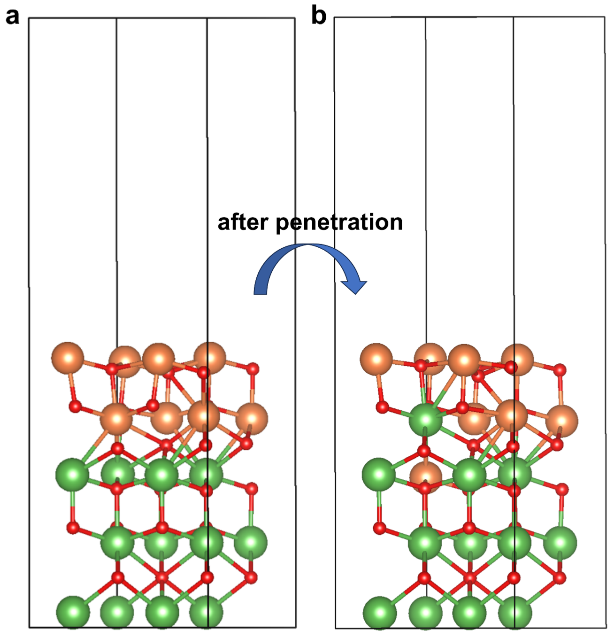

Fig. R19 | The optimized structure of the heterojunction obtained by DFT calculation before (a) and after (b) the penetration (one Bi atom and one La atom) process at the interface. The system energy of these two structures is -350.89 and -350.64 eV, respectively.

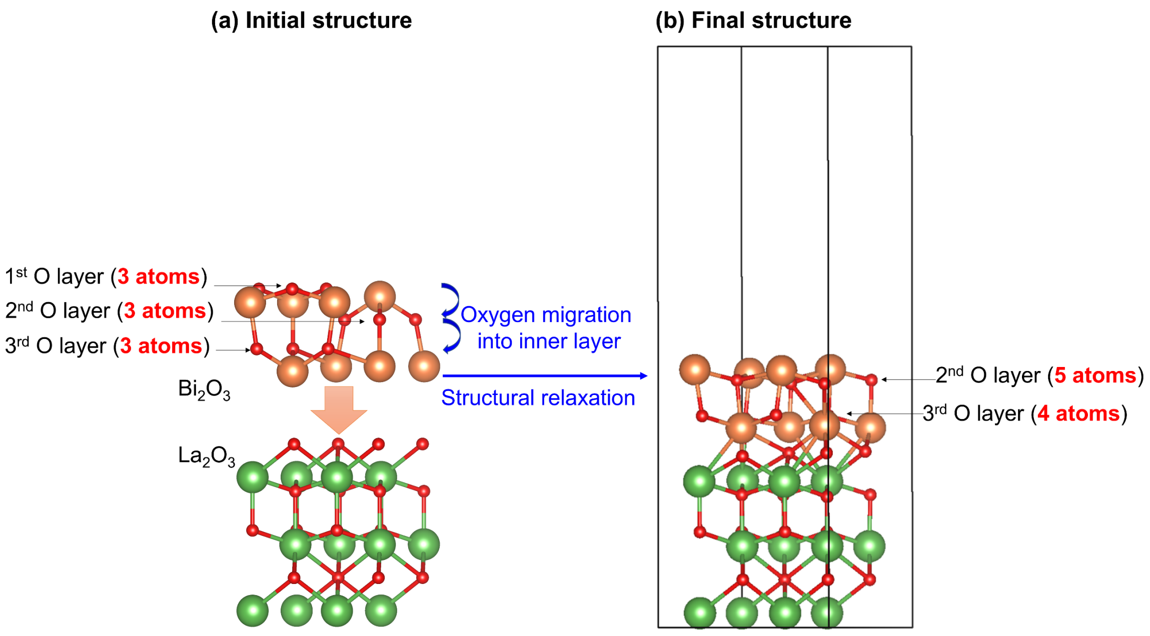

Fig. R20 | Scheme of the formation of δ-Bi_2O_3/La_2O_3 model from (a) initial structure to (b) final structure.

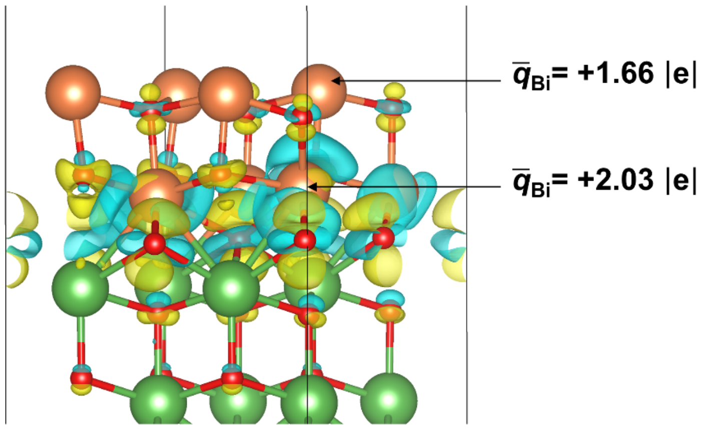

Fig. R21 | The charge density difference plot ($\rho_{CDD} = \rho_{heterojunction} - \rho_{Bi2O3} - \rho_{La2O3}$) is also given for the obtained heterojunction model, with an isosurface value of 0.005 e/Å^3.

(6) The reasonable range of EXAFS fitting parameters, including R factor < 0.02. However, there are some results in Tables S2 and S3 that don't comply with this rule. The R factor for pure Bi_2O_3 at -0.65 V even reaches 0.024. Apart from these, all the fitting results using S_0^2 of 0.31, which is much more unreasonable. The rational range of the S_0^2 is 0.600 < S_0^2 < 1.000.

[Reply]: We sincerely thank the reviewer for the insightful and critical comments regarding the EXAFS fitting parameters, especially the amplitude reduction factor (S_0^2), which are crucial for ensuring the rigor of our data analysis. We acknowledge that fixing S_0^2 at 0.31 was inappropriate and likely led to unreliable structural parameters. In the EXAFS fitting procedure, the amplitude reduction factor S_0^2 is first determined by fitting α-Bi_2O_3 standard. This S_0^2 value is then fixed

during the fitting of experimental samples to extract structural parameters such as coordination number (CN). It should be noted that the α-Bi_2O_3 standard is subject to an improper constraint in the initial fitting, which introduces an error into the derived S_0^2 value. Specifically, in our previous manuscript, the Bi–O coordination number was fixed at 6 during the fitting of the α-Bi_2O_3 standard to determine the S_0^2. However, this assumption is inconsistent with the well-established structural data for α-Bi_2O_3, where the Bi–O CN is reported as 3 in most literature (see e.g., *Angew. Chem. Int. Ed.* 2025, e17618; *Chem. Eng. J.* 2025, 504, 158854; *Angew. Chem. Int. Ed.* 2025, *64*, e202423658; *Chem Catal.* 2023, 3, 100842). Given the strong coupling between S_0^2 and the CN in EXAFS analysis, fixing the CN at an erroneously high value of 6 likely resulted in an unrealistically low S_0^2 of 0.31—significantly below the rational range (0.6–1.0). We have now performed a new fitting of the α-Bi_2O_3 standard using a Bi–O coordination number of 3, yielding a reasonable S_0^2 value of 0.70, which is fixed in all subsequent EXAFS fits of the experimental samples. As shown in **Tables R3–R5** and **Figs. R22–R24**, the refined EXAFS fits yield R-factors below 0.02 for both pure Bi_2O_3 and the δ-Bi_2O_3/La_2O_3 samples. In these fits, the Bi–O coordination number in pure Bi_2O_3 decreases from ~3.2 to ~2.1 as the potential is swept from OCP to −0.95 V vs. RHE, whereas that in δ-Bi_2O_3/La_2O_3 decreases from ~4.9 to ~4.3 over the range from OCP to −1.05 V vs. RHE. We have updated all related results and discussion in the revised manuscript accordingly.

Figs R22, R23, R24 and Tables R3, R4, R5 have been updated as Supplementary Figs. 47, 49, 50 and Supplementary Tables 5, 6, 7 in the revised Supporting Information, respectively.

Revised parts in the manuscript:

Pages 9–10, lines 339–342

The Fourier transform (FT) k^2-weighted extended X-ray absorption fine structure (EXAFS) spectra reveal that the dominant Bi–O scattering peak in R-space shifts from ~1.60 Å at OCP to ~1.67 Å at −0.95 V vs. RHE (Fig. 3b), accompanied by a decrease in the Bi–O coordination number from ~3.2 to ~2.1 (Supplementary Table 5 and Supplementary Fig. 47).

Page 10, lines 358–371

Additionally, the EXAFS fitting results (Supplementary Table 6 and Supplementary Fig. 49) reveal a shorter average Bi–O bond length in 7% δ-Bi_2O_3/La_2O_3 (~2.10 Å) compared to pure Bi_2O_3 (~2.15 Å), indicating that the interphase interaction between La_2O_3 and δ-Bi_2O_3 effectively contracts the Bi−O bonds. This bond contraction suggests an enhanced local structural rigidity, which contributes to improve the stability under operational CO_2RR conditions. As shown in Fig. 3d, when the potential is swept from OCP to −1.15 V vs. RHE, the Bi–O coordination number decreases slightly from ~4.9 to ~4.3 and remains stable.

Concurrently, the peak position of the first Bi–O coordination shell in R-space exhibits negligible shift, and no Bi–Bi scattering features are detected in the EXAFS spectra. Furthermore, an extended electrolysis experiment is performed at −1.15 V vs. RHE for 150 minutes to assess the robustness of the Bi–O bonds in the δ-Bi_2O_3/La_2O_3. Throughout this process, the Bi L_3-edge XANES spectra exhibit only a minor energy shift (Fig. 3e), and the Bi–O coordination number undergoes a slight change from ~4.3 to ~4.1 (Fig. 3f, Supplementary Fig. 50 and Supplementary Table 7).

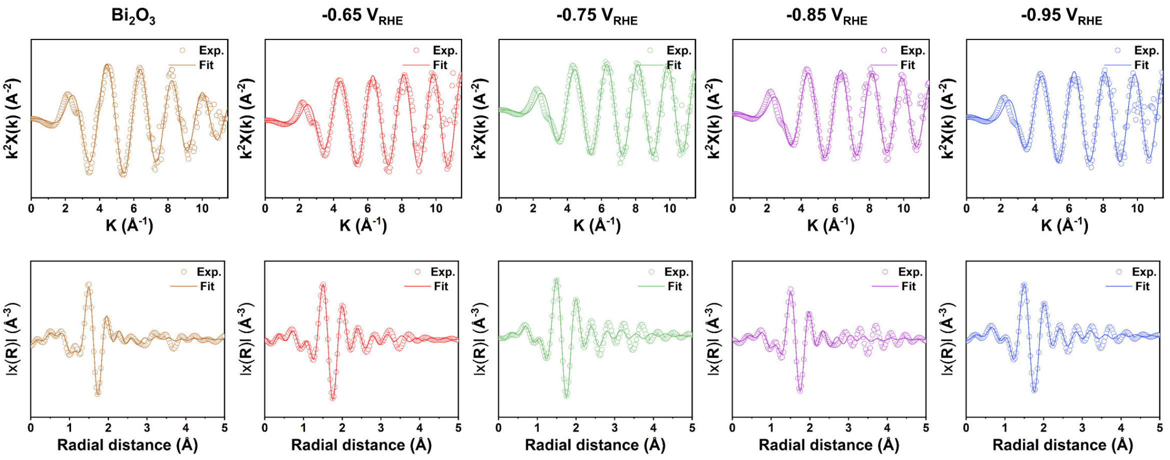

Fig. R22 | k^2-weighted Bi L_3-edge EXAFS oscillations (up) and their associated Fourier transforms (down) for pure Bi_2O_3 under different applied potential; Experimental data, dotted lines; Fitting curves, solid lines.

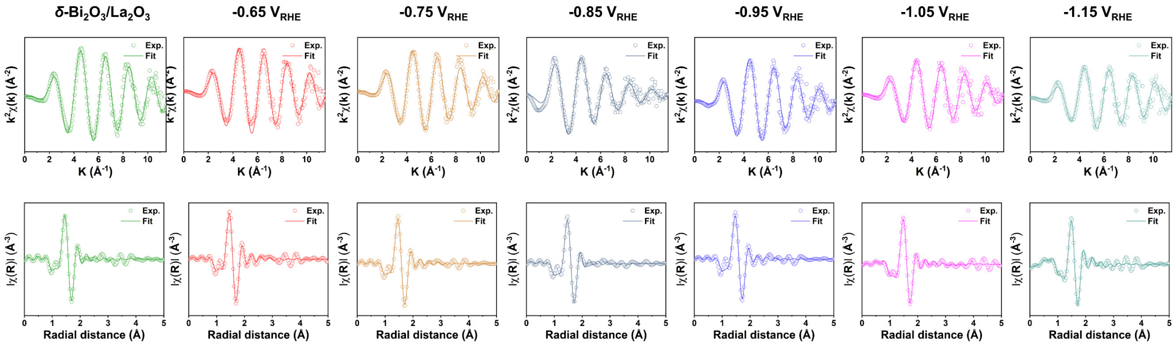

Fig. R23 | k^2-weighted Bi L_3-edge EXAFS oscillations (up) and their associated Fourier transforms (down) for the 7% δ-Bi_2O_3/La_2O_3 under different applied potential; Experimental data, dotted lines; Fitting curves, solid lines.

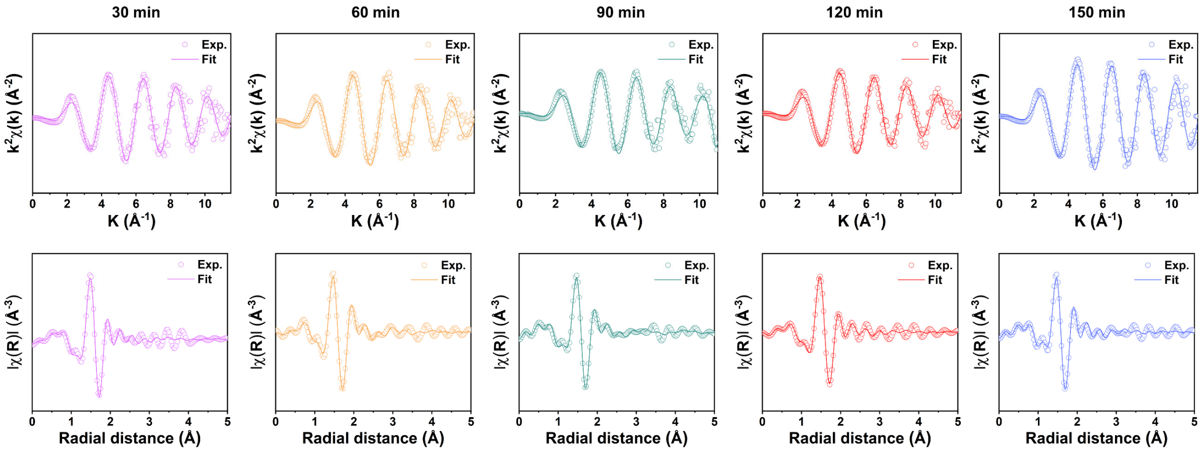

Fig. R24 | k^2-weighted Bi L_3-edge EXAFS oscillations (up) and their associated Fourier transforms (down) for the 7% δ-Bi_2O_3/La_2O_3 during 150 min of reaction at −1.15 V vs. RHE; Experimental data, dotted lines; Fitting curves, solid lines.

Table R2 | Structural parameters of Bi_2O_3 standard extracted from the Bi standard L_3-edge EXAFS fitting.

Sample	CN	S_0^2	R (Å)	ΔE_0 (eV)	σ^2 * 10^{-3} (Å^2)	R factor
Bi_2O_3-standard	3*	0.70	2.16±0.01	-2.58±1.30	4.14±1.14	0.004
	6*	0.31	2.15±0.01	-3.25±1.30	-2.09±1.56	0.003

Table R3 | Structural parameters of pure Bi_2O_3 under CO_2RR conditions extracted from the Bi L_3-edge EXAFS fitting. (S_0^2 = 0.70).

Sample	CN	S_0^2	R (Å)	ΔE_0 (eV)	σ^2 * 10^{-3} (Å^2)	R factor
OCP	3.2±0.4	0.70	2.15±0.01	-3.77±1.82	5.50±1.32	0.005
-0.65	2.8±0.4	0.70	2.18±0.02	-3.48±1.21	0.59±2.38	0.014
-0.75	2.3±0.5	0.70	2.17±0.01	-0.64±2.51	0.75±2.46	0.012
-0.85	2.2±0.4	0.70	2.17±0.02	-1.92±1.89	2.56±2.32	0.006
-0.95	2.1±0.3	0.70	2.18±0.01	-0.57±1.33	0.58±2.59	0.008
Bi_2O_3-standard	3*	0.70	2.16±0.01	-2.58±1.30	4.14±1.14	0.004

* This value was fixed during EXAFS fitting, based on the known structure of Bi_2O_3.

Table R4 | Structural parameters of the 7% δ-Bi_2O_3/La_2O_3 under CO_2RR conditions extracted from the Bi L_3-edge EXAFS fitting. (S_0^2 = 0.70).

Sample	CN	S_0^2	R (Å)	ΔE_0 (eV)	σ^2 * 10^{-3} (Å^2)	R factor
OCP	4.9±0.7	0.70	2.09±0.02	-6.56±1.28	6.11±2.40	0.016
-0.65	4.8±0.6	0.70	2.10±0.01	-5.94±1.30	6.13±3.00	0.017
-0.75	4.8±0.4	0.70	2.11±0.02	-5.39±1.27	7.29±3.10	0.015
-0.85	4.7±0.5	0.70	2.11±0.02	-6.05±1.02	7.84±2.10	0.019
-0.95	4.6±0.4	0.70	2.12±0.01	-5.21±1.60	8.76±3.02	0.013
-1.05	4.5±0.6	0.70	2.12±0.01	-5.22±1.32	8.69±3.56	0.010

-1.15	4.3±0.6	0.70	2.12±0.01	-7.57±0.92	7.27±1.58	0.009
Bi_2O_3-standard	3*	0.70	2.16±0.01	-2.58±1.30	4.14±1.14	0.004

* This value was fixed during EXAFS fitting, based on the known structure of Bi_2O_3.

Table R5 | Structural parameters of the 7% δ-Bi_2O_3/La_2O_3 under CO_2RR conditions at -1.15V vs. RHE, extracted from the Bi L_3-edge EXAFS fitting. (S_0^2 = 0.70).

Sample	CN	S_0^2	R (Å)	ΔE_0 (eV)	$\sigma^2 * 10^{-3}$ (Å^2)	R factor
30 min	4.3±0.6	0.70	2.12±0.01	-7.57±0.92	7.27±1.58	0.009
60 min	4.3±0.5	0.70	2.11±0.02	-5.35±1.48	5.90±1.64	0.005
90 min	4.2±0.3	0.70	2.11±0.01	-4.99±1.21	6.30±1.20	0.006
120 min	4.1±0.4	0.70	2.11±0.01	-6.63±1.11	6.09±1.80	0.010
150 min	4.1±0.3	0.70	2.10±0.02	-6.27±1.35	5.83±1.45	0.007
Bi_2O_3-standard	3*	0.70	2.16±0.01	-2.58±1.30	4.14±1.14	0.004

* This value was fixed during EXAFS fitting, based on the known structure of Bi_2O_3.

(7) There are many experiment details missing, such as the electrode preparation methods. The authors use PTFE in the MEA electrolyzer while the work doesn't show the method to overcome the conductivity issues. The information of the GDL in the flow cell is also missing. Apart from these, La_2O_3 or the heterojunctions show a relatively large morphology of more than 1 μm with average diameters of the pores of about 2.39 nm. La_2O_3 has poor conductivity and the method to improve the catalyst conductivity is also missing. Then, the potentials in the H cell were referenced to a reversible hydrogen electrode (RHE) without iR compensation while the potentials in the flow cell and the voltages in the MEA weren't clarified. Finally, the produced formate can move across the AEM to the cathode and encounter its reoxidation, especially in the MEA system. However, the authors don't explain it.

[Reply]: We sincerely thank the reviewer for these critical and constructive comments. We fully agree that the missing details are essential for reproducibility and a thorough understanding of our work. We have now comprehensively supplemented the Experimental Section in the revised manuscript as follows:

Point 1: Illustration of a gas diffusion electrode & Clarification iR compensation

We apologize for the omission. The detailed electrode preparation protocol for all three configurations (H-cell, flow cell, and MEA), including the ink formulation, sonication, and coating, has been added to the Experimental Section ("***Fabrication of the working electrodes***") on *Pages 2-3* of the revised Supporting Information. Specifically, the catalyst ink was formed by mixing 4 mg of catalyst, 1 mg of Vulcan XC-72 carbon black (Cabot Corporation), 50 μL of 5 wt% Nafion solution (Sigma-Aldrich), and 950 μL of ethanol (99.9%) together to form a homogeneous ink by ultrasonicating for at least 1 hour. This blending treatment with conductive

carbon black would significantly enhance the conductivity of the catalyst layer (CL). Then, 250 μL of homogeneous ink was evenly spray-coated onto a PTFE-modified gas diffusion layer (GDL, 1 cm^2, SGL 38BC, Germany) surface under ambient conditions. The catalyst loading was controlled to be 1 mg cm^{-2} by weighing. Additionally, we apologize for the confusion caused by the inappropriate labeling of PTFE in the original Fig. 1b. In fact, PTFE was used as a hydrophobic binder at a low weight ratio and was homogeneously mixed with highly conductive acetylene black prior to catalyst loading. As displayed in **Fig. R25**, CF-supported GDLs typically consist of a microporous layer (MPL) and a hydrophobic carbon fiber substrate (CFS). The MPL is usually composed of hydrophobic PTFE and conductive carbon black (e.g., acetylene black), which plays a key role in striking a fine balance between the hydrophobicity and conductivity of the GDE while preventing electrolyte flooding. Regarding the use of PTFE in our method, according to the manufacturer's technical specifications, this hydrophobic GDL consists of a CFS containing 5 wt% PTFE and a MPL with 20–25 wt% PTFE. Fig. R25 has been added as Supplementary Fig. 5, and the GDL architecture details have been added in *Page 2* of the revised Supporting Information.

In this work, since the CO_2RR performance was evaluated under galvanostatic conditions in both flow cell and MEA electrolyzers, the reported electrode potentials were generally not iR-corrected based on previous literatures (*Nat. Commun.* 2025, 16, 10169; *Nat. Synth.* 2025, 4, 799); *Science* 2025, 388, 3834; *Nat. Commun.* 2025, 16, 10783; *Nat. Commun* 2025, 16, 3407). We have added "*All potentials were referenced to a reversible hydrogen electrode (RHE) without iR compensation.*"and "*The full-cell potentials in the two-electrode MEA system were shown without iR correction.*" in the revised Supporting Information. (Page 4, lines 122, 123; Page 5, lines 136, 137).

Fig. R25a have been updated as Fig. 1b in the revised main text, Fig. R25b have been added as Supplementary Fig. 5 in the revised Supporting Information, respectively.

Revised parts in the Supporting Information:

Pages 2–3, lines 54–61

Fabrication of the working electrodes. The catalyst ink was formed by mixing 4 mg of catalyst, 1 mg of Vulcan XC-72 carbon black (Cabot Corporation), 50 μL of 5 wt% Nafion solution (Sigma-Aldrich), and 950 μL of ethanol (99.9%) together to form a homogeneous ink by ultrasonicating for at least 60 min. This blending treatment with conductive carbon black would significantly enhance the conductivity of the catalyst layer (CL). Then, 250 μL of homogeneous ink was evenly spray-coated onto a PTFE-modified gas diffusion layer (GDL, 1 cm^2, SGL

38BC, Germany) surface under ambient conditions. The catalyst loading was controlled to be 1 mg cm^{-2} by weighing.

Revised parts in the manuscript:

Page 3, lines 106–108

The cross-sectional SEM image in Fig. 1b shows a well-defined, densely packed catalyst layer on the carbon fiber-supported microporous layer (MPL). The detailed architecture of the gas diffusion layer (GDL) can be seen in Supplementary Fig. 5.

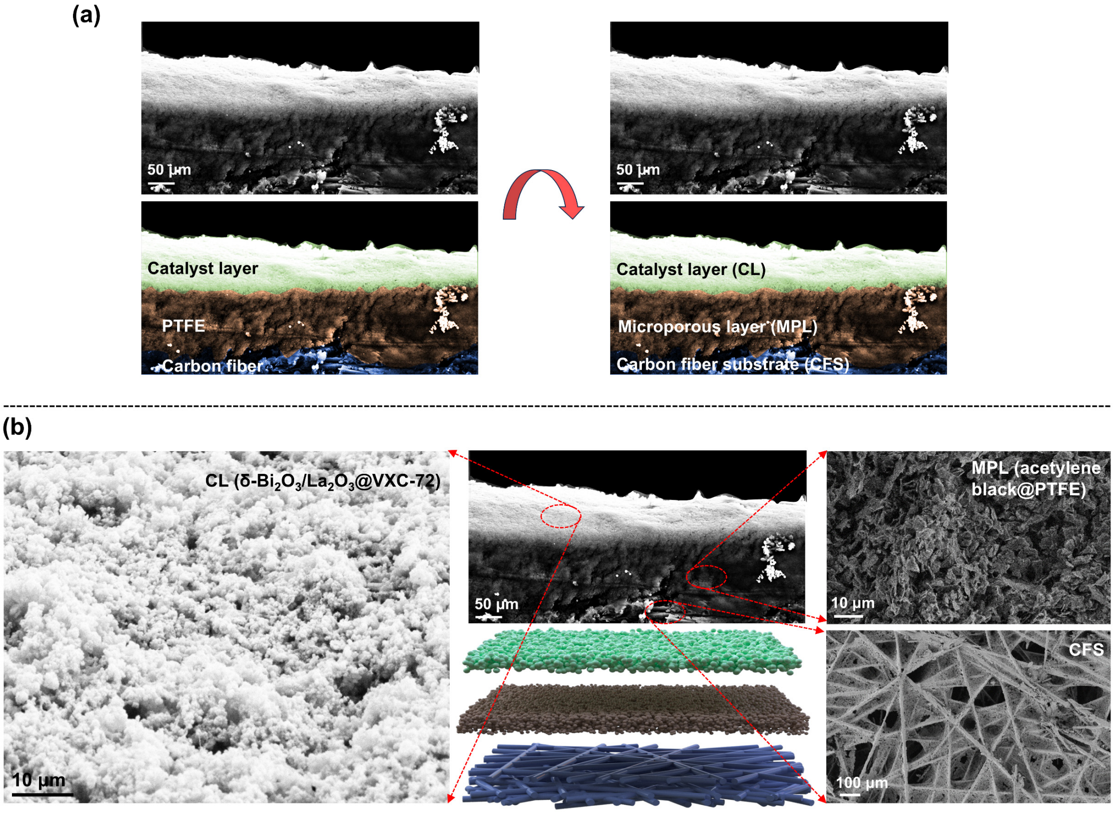

Fig. R25 | Illustration of a gas diffusion electrode. (a) The inappropriate labeling of PTFE in the original Fig. 1b has been revised. (b) Scanning electron microscopy (SEM) images of the MPL, the CFS and the CL.

Point 3: Formate crossover and reoxidation

We thank the reviewer for raising this insightful question. In line with recently established protocols for collecting anionic CO_2 reduction products (e.g., formate and acetate), the formate produced in our MEA and flow-cell reactors were collected from both the cathodic and anodic compartments across all current densities. In the AEM-based MEA reactors, the cathode side is supplied with humidified CO_2. The generated formate accumulates in the thin aqueous layer at the catalyst–membrane interface, leading to a locally high formate concentration. This concentration gradient drives the diffusion of formate anions from the cathode to the anode

through the anion exchange membrane (AEM). Based on it, the formate Faradaic efficiency (FE) reported in Fig. 2e accounts for all formate detected in both the cathode and anode compartments of the MEA reactor; that is, the efficiency was calculated based on the total formate yield from both compartments. To ensure accurate quantification, the formate that migrated to the anolyte was quantified by ^{1}H NMR (**Fig. R26b**) and high-performance liquid chromatography (HPLC, **Fig. R27b**) after electrolysis for 45 min. Based on the multiple-point calibration curves (**Fig. R28**), the formate concentrations in the catholyte and anolyte were quantified. The FEs of formate as a function of current density, along with the corresponding crossover ratios (i.e., $FE_{anolyte}$ / [$FE_{anolyte}$+$FE_{catholyte}$]), are listed in **Table R7**. The crossover ratio shows a progressively increasing trend with rising current density, ascribed to the enhanced electromigration driven by the applied potential gradient. Specifically, at 500 mA cm^{-2}, about 32.6% of formate was crossed to the anolyte chamber. We also examined formate crossover in a flow-cell reactor. Fig. 2c reports the formate Faradaic efficiency corrected for anolyte crossover. The formate in the anolyte was also quantified by ^{1}H NMR (**Fig. R26a**) and HPLC (**Fig. R27a**) after electrolysis for 45 min. As presented in **Table R6**, the crossover ratio is approximately 6.9% at 500 mA cm^{-2}, much lower than the values observed in MEA-based systems. While in a Nafion-based H-cell (Fig. 2a), no formate was detected in the anolyte at potentials between −0.65 and −1.25 V vs. RHE (**Fig. R29**). It is noteworthy that even when accounting for the Faradaic efficiency corresponding to formate detected on the anode side, the total Faradaic efficiency of all detected reduction products (including formate, H_2, and CO) remains below 100% in the flow-cell and MEA electrolyzers. This discrepancy may arise from product loss due to the reoxidation of formate that migrated to the anode, as pointed out by the reviewer. To prevent severe accumulation and anodic oxidation of formate at the anode, the anolyte was circulated at a high flow rate of 30 mL min^{-1}, as previously described in Supporting Information (*see Experimental Details, Page-4*). Additionally, the catholyte and anolyte were refreshed every 45 min during electrolysis experiments at varying current densities, ensuring that formate diffused into the anode could be recovered as much as possible before its complete oxidation. These greatly shorten the residence time of formate at the anode, thereby minimizing its anodic oxidation. As displayed in Figs. 2c, 2e, the FE loss due to anodic oxidation of formate is less than 5% in both flow cell and MEA electrolyzers. Additionally, we supplemented a control experiment to verify whether the severe formate oxidation occurs at the anode during the electrolysis process. In the identical MEA reactor, identical IrO_2/C is used as the anode catalyst for the oxygen evolution reaction (OER), and commercial Pt/C is used as the cathode catalyst for the hydrogen evolution reaction (HER). The anolyte, a mixture of 1 M $KHCO_3$ and 34.4 mM HCOOK is circulated at a flow rate of 30 mL min^{-1} by a mixed-flow pump, consistent

with the conditions employed in our CO_2RR measurements. The MEA reactor was operated under varying current density from 50 to 500 mA cm^{-2} at 70 °C, with the anolyte refreshed every 45 min. As shown in **Figs. R30, R31**, ^{1}H NMR analysis reveals that the formate concentration in the anolyte decreased slightly, indicating that the extent of formate oxidation at the anode is negligible under the present operating conditions. This control experiment supports the validity of formate quantification in our MEA system, in agreement with recent literatures on similar MEA-based measurements (*Nat. Catal.* 2022, 5, 564; *Energy Environ. Sci.*, 2020, 13, 977; *Energy Environ. Sci.*, 2021, 14, 4169; *J. Catal.*, 2020, 385, 140). We have added the following descriptions into the revised manuscript and Supplementary Information:

Figs R26, R27, R28 and Tables R6, R7 have been added as Supplementary Figs. 37, 38, 35 and Supplementary Tables 2, 3 in the revised Supporting Information, respectively.

Revised parts in the manuscript:

Page 7, lines 257–262

All potentials were referenced to a reversible hydrogen electrode (RHE) without iR compensation. Because the produced formate can diffuse from the cathode to the anode compartment through the anion exchange membrane (AEM), it was collected from both compartments. To ensure accurate quantification, the formate that migrated to the anolyte was quantified by both ^{1}H NMR and high-performance liquid chromatography (HPLC, Supplementary Fig. 35).

Page 8, lines 269–272

Supplementary Figs. 37a and 38a show the quantitative analysis results of formate in the anolyte by ^{1}H NMR and HPLC, respectively. The corresponding crossover ratios (i.e., $FE_{anolyte}$ / [$FE_{anolyte}$+$FE_{catholyte}$]) as a function of current density are listed in Supplementary Table 2.[34]

Page 8, lines 281–287

...while the 10% and 15% δ-Bi_2O_3/La_2O_3 catalysts exhibit lower formate Faradaic efficiency and poorer operational stability due to the aggregation of δ-Bi_2O_3 sub-nanoparticles (Supplementary Fig. 42). Specifically, the 10% δ-Bi_2O_3/La_2O_3 catalyst delivers a formate Faradaic efficiency of ~91.5% and maintains stable performance for ~175 h, whereas the 15% sample exhibits a lower efficiency (~89.3%) and deactivates after ~154 h. These results suggest that a 7% Bi loading may represent an optimal balance between active site density and dispersion.

Page 8, lines 293–302

The galvanostatic curves in the current range of 50–500 mA cm^{-2} are shown in Supplementary

Fig. 44. The analysis results of formate in the anolyte by ^{1}H NMR and HPLC are displayed in Supplementary Fig. 37b and 38b, respectively. The corresponding crossover ratios as a function of current density are listed in Supplementary Table 3. The MEA-based electrolyzer achieves a formate Faradaic efficiency of ~93.2% and a current density of 500 mA cm^{-2} at a cell voltage of ~2.95 V, with the H_2 Faradaic efficiency remaining below 4% (Fig. 2e). Notably, to prevent severe accumulation and anodic oxidation of formate at the anode, the anolyte was circulated at a flow rate of 30 mL min^{-1} and refreshed every 45 minutes. Therefore, the Faradaic efficiency loss due to anodic oxidation of formate is less than 5% in both flow cell and MEA electrolyzers.[34]

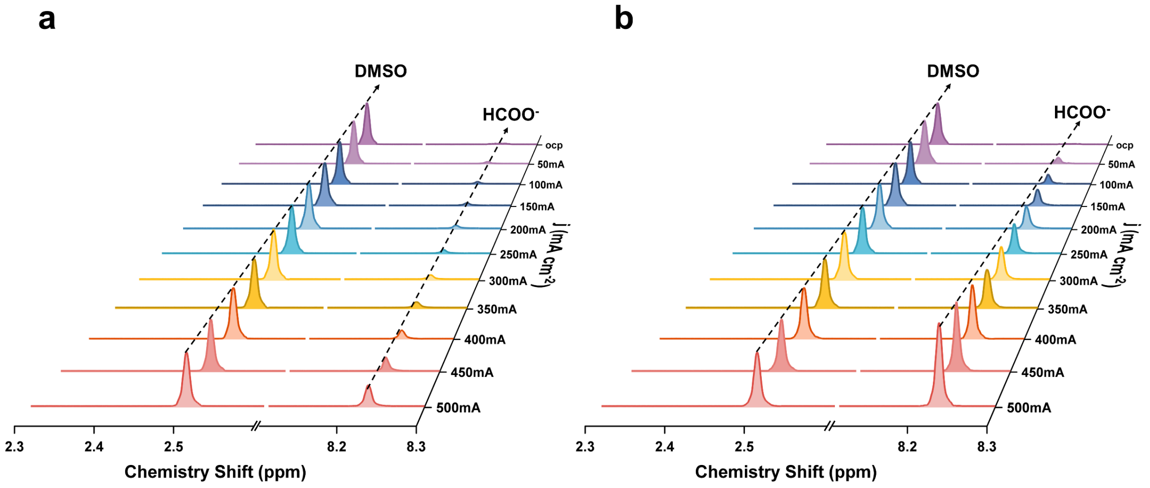

Fig. R26 | ^{1}H NMR spectra of the anolyte for formate analysis. Representative signals at different current densities on 7% δ-Bi_2O_3/La_2O_3 within (a) Flow-cell and (b) MEA electrolyzer.

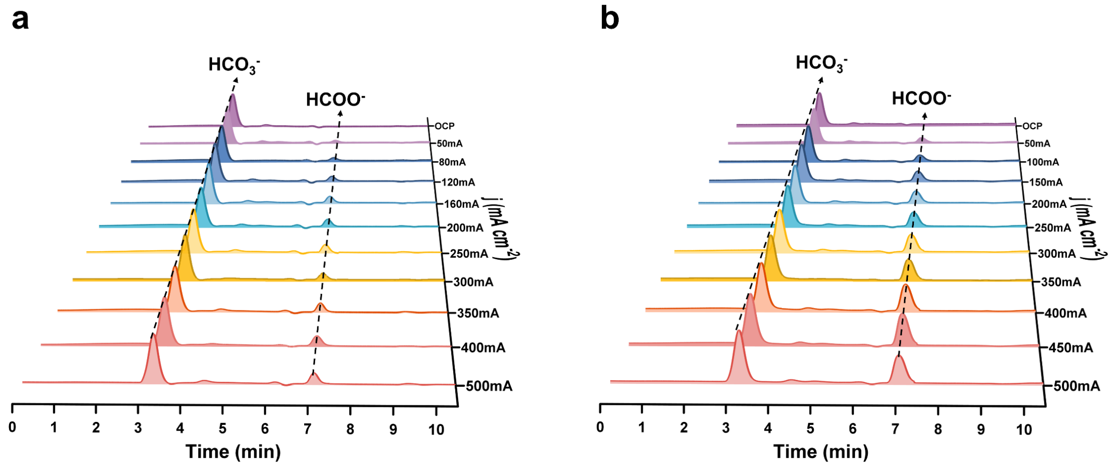

Fig. R27 | HPLC chromatograms of the anolyte for formate analysis. Representative signals at different current densities on 7% δ-Bi_2O_3/La_2O_3 within (a) Flow-cell and (b) MEA electrolyzer.

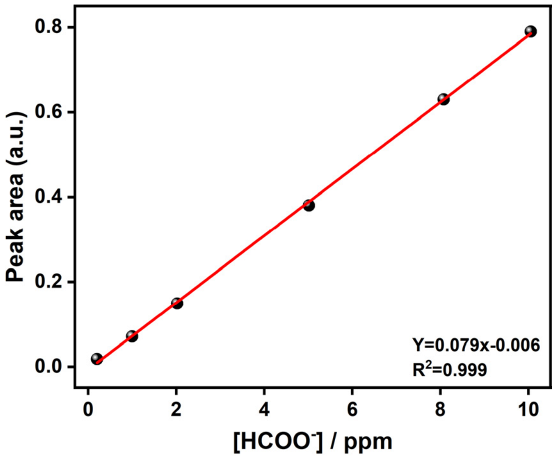

Fig. R28 | HPLC calibration curves of the formate.

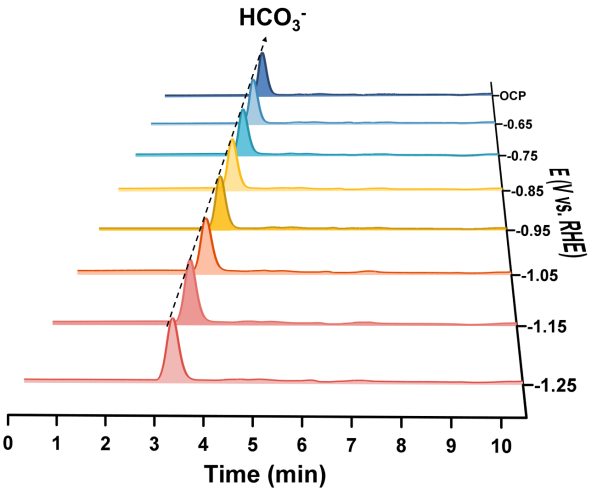

Fig. R29 | HPLC chromatograms of the anolyte from H-Cell for formate analysis.

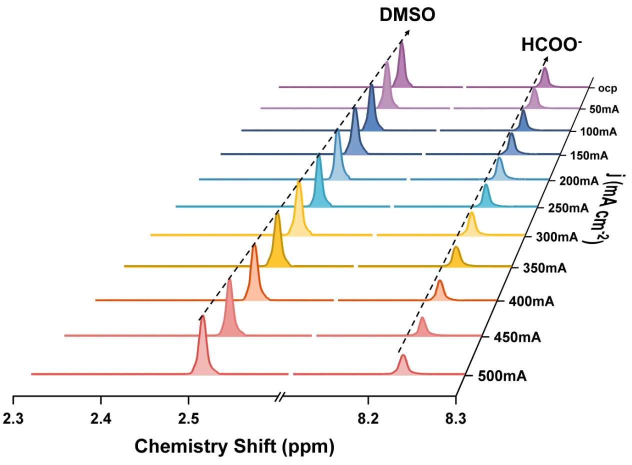

Fig. R30 | ^{1}H NMR spectra of the anolyte in the MEA reactor for formate analysis.

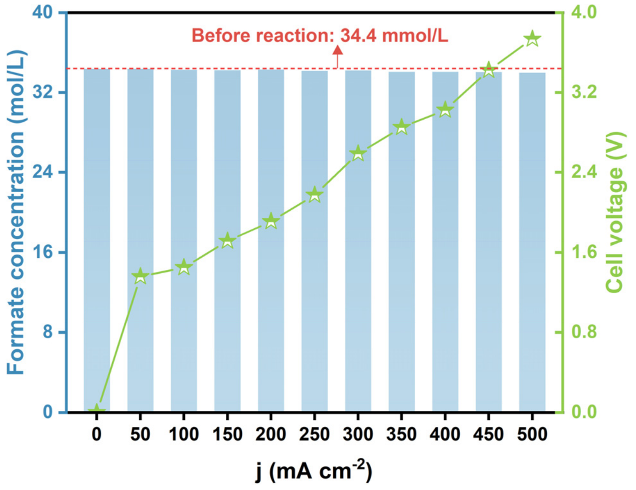

Fig. R31 | Formate quantification of the anolyte in the MEA reactor.

Table R6 | Faradaic Efficiencies of formate on 7% δ-Bi_2O_3/La_2O_3 in Flow-cell electrolyzer.

Current density (mA cm^{-2})	$FE_{formate}$ (%) in catholyte	$FE_{formate}$ (%) in anolyte	Crossover ratio (%)
50	85.8	2.2	2.5
80	87.8	2.7	3.0
120	88.5	3.2	3.5
160	88.7	3.5	3.8
200	89.3	3.9	4.2
250	89.7	4.1	4.4
300	89.5	4.6	4.9
350	84.1	5.8	6.4
400	78.2	6.0	7.1
500	74.3	5.5	6.9

Table R7 | Faradaic Efficiencies of formate on 7% δ-Bi_2O_3/La_2O_3 in MEA electrolyzer.

Current density (mA cm^{-2})	$FE_{formate}$ (%) in catholyte	$FE_{formate}$ (%) in anolyte	Crossover ratio (%)
50	72.6	6.1	7.8
100	74.3	8.8	10.5
150	73.9	11.6	13.6
200	72.7	16.6	18.6
250	70.9	21.4	23.2
300	68.0	26.7	28.2
350	66.2	29.5	30.9
400	63.6	30.7	32.5
450	58.7	32.6	35.7
500	60.6	29.3	32.6

Reviewer:2

The manuscript titled "Stabilizing Sub-2 nm δ-Bi_2O_3 via Strong Lanthanide-Oxide-Support Interaction for Durable CO_2 Electroreduction to Formate Beyond 200 Hours" by Qianmin Wu et al discusses the new Bi-La catalyst for CO_2RR, which shows good performance for formate production. Authors demonstrate the stability of the catalyst, and, in particular, the stabilization of Bi cationic nature by La oxide support. The presented results are interesting, however, there are some issues regarding the proposed structure model of the catalyst which need to be explained or corrected.

[Reply]: We appreciate reviewer's positive assessment on our work, and thank for highlighting the importance of the structural model of the Bi–La catalyst. We agree that a clear and accurate structural description is essential to support our mechanistic conclusions. In response to your comments, we have performed additional characterization and analysis to further validate the proposed structure. We hope these clarifications adequately address the reviewer's concerns and strengthen the structural foundation of our work.

(1) Authors continuously refer to their catalyst as "delta-Bi_2O_3". However, I do not see any conclusive evidence that the formed oxide particles indeed assume delta phase and not some other of the numerous possible crystallographic phases for bismuth oxides. In particular, authors show ex-situ and in-situ XRD data for their catalysts. However, these have little resemblance (if any) to the XRD of delta Bi_2O_3. Not only all peak positions are different, but also relative Bragg peak intensities do not match those in the pure Bi_2O_3. Perhaps even more critically, I do not understand why there should be any peaks of bismuth oxide at all in the XRD data, if authors model featuring 2 nm particles is correct. Such small particles would be X-ray amorphous, and would not produce any strong Bragg reflections at all. I would rather consider that Bi is incorporated into lanthanum oxide support, forming a new oxide phase that has nothing to do with the delta Bi_2O_3. Can authors exclude incorporation of significant amounts of Bi into the support?

[Reply]: We sincerely appreciate the reviewer for raising these critical and insightful points. First, theoretical XAS simulations are conducted for multiple structural models (α-, β-, and δ-Bi_2O_3) to distinguish the δ-phase from other Bi_2O_3 polymorphs by comparing the simulated and experimental XANES spectra. As shown in **Fig. R32**, the simulated Bi L_3-edge XANES spectra for α-Bi_2O_3 and β-Bi_2O_3 do not match the experimental spectrum of the Bi_2O_3/La_2O_3 sample, whereas that for δ-Bi_2O_3 shows excellent agreement with the experimental data, indicating that the Bi_2O_3 in the composite predominantly exists as the δ-phase. Additionally, the Raman spectra of Bi_2O_3/La_2O_3 (**Fig. R33**) show only the characteristic peak at 620 cm^{-1}, assigned to the T_{2g} mode of δ-Bi_2O_3 (i.e., symmetric Bi–O stretching vibrations; *J. Alloys Compd.* 2017, 723, 520;

J. Alloys Compd. 2016, 682, 80; *Appl. Phys. Lett.* 2005, 87, 231916; *J. Appl. Spectrosc.* 2002, 69, 1), along with peaks at 110, 189, and 449 cm^{-1}, attributed to the La−O vibrational mode, which further supports that the Bi_2O_3 phase in Bi_2O_3/La_2O_3 is the δ-phase. This suggests that the δ-Bi_2O_3 phase, which is typically stable only at high temperatures, is stabilized at room temperature through strong metal oxide–support interaction in the constructed Bi_2O_3/La_2O_3 heterostructure. Additionally, to assess whether Bi is significantly incorporated into the La_2O_3 lattice, we further compare the experimental Bi L_3-edge XANES spectrum of the δ-Bi_2O_3/La_2O_3 sample with the spectrum simulated for Bi-doped La_2O_3. As shown in Fig. R32, the simulated XANES spectrum for Bi-doped La_2O_3 does not match the experimental features, indicating that Bi is not substantially incorporated into the La_2O_3 lattice. Furthermore, the newly supplemented La L_3-edge XANES spectra show that the La absorption edge position in the δ-Bi_2O_3/La_2O_3 is essentially identical to that of pure La_2O_3 (**Fig. R34**), also verifying negligible incorporation of Bi into the La_2O_3 lattice. Besides, we further performed La L_3-edge XANES simulations based on these structural models—La-doped α-Bi_2O_3, La-doped β-Bi_2O_3, La-doped δ-Bi_2O_3, and undoped La_2O_3—to identify the local coordination environment of La in the δ-Bi_2O_3/La_2O_3 catalyst. As shown in **Fig. R35**, the simulated La L_3-edge XANES spectra for La-doped α-, β-, and δ-Bi_2O_3 show poor agreement with the experimental spectrum of the δ-Bi_2O_3/La_2O_3 sample, whereas the simulated spectrum for pristine La_2O_3 closely matches the experimental data, also implying negligible incorporation of La into the δ-Bi_2O_3 lattice.

Figs. R32, R34, R35 have been added as Supplementary Figs. 23, 20, 24 in the revised Supporting Information, respectively. Fig. R33a has been updated as Supplementary Fig. 14 in the revised Supporting Information.

Revised parts in the manuscript:

Page 5, lines 179–201

To further determine whether interfacial Bi and La atoms interdiffuse, we perform X-ray absorption spectroscopy (XAS) measurements and simulations. First, the La L_3-edge XANES spectra show that the La absorption edge position in the δ-Bi_2O_3/La_2O_3 is essentially identical to that of pure La_2O_3 (Supplementary Fig. 20), demonstrating that Bi is not significantly incorporated into the La_2O_3 lattice. Notably, the fitted La–O bond lengths are shorter in δ-Bi_2O_3/La_2O_3 than in pure La_2O_3, further suggesting a lattice contraction in the La_2O_3 support (Supplementary Fig. 21, 22 and Supplementary Table 1). We further carry out theoretical XAS simulations for Bi-doped La_2O_3 and pure α-, β-, and δ-Bi_2O_3 to identify the local coordination structure of Bi in δ-Bi_2O_3/La_2O_3 by comparing the simulated and experimental XANES spectra. As shown in Supplementary Fig.23, the simulated Bi L_3-edge XANES spectrum for Bi-doped

La_2O_3 does not match the experimental spectrum of the δ-Bi_2O_3/La_2O_3 sample, whereas the simulated spectrum for pure δ-Bi_2O_3 shows excellent agreement, also verifying negligible incorporation of Bi into the La_2O_3 lattice. Besides, the simulated Bi L_3-edge XANES spectra for α-Bi_2O_3 and β-Bi_2O_3 do not match the experimental spectrum, further distinguishing that the Bi_2O_3 in the composite exists as the δ-phase. Subsequently, to determine whether La is incorporated into the δ-Bi_2O_3 lattice, we perform La L_3-edge XANES simulations based on these structural models—La-doped α-Bi_2O_3, La-doped β-Bi_2O_3, La-doped δ-Bi_2O_3, and undoped La_2O_3. As shown in Supplementary Fig. 24, the simulated La L_3-edge XANES spectra for La-doped α-, β-, and δ-Bi_2O_3 show poor agreement with the experimental spectrum of the δ-Bi_2O_3/La_2O_3 sample, whereas the simulated spectrum for pristine La_2O_3 closely matches the experimental data, also indicating that La is not significantly incorporated into the δ-Bi_2O_3 lattice. Consequently, large-scale interdiffusion of Bi and La atoms is unlikely to occur.

Page 4, lines 146–150

Additionally, the Raman spectra of δ-Bi_2O_3/La_2O_3 (Supplementary Fig. 14) exhibit only the δ-Bi_2O_3 T_{2g} mode at ~620 cm^{-1} and La_2O_3-related peaks at ~110, ~189, and ~449 cm^{-1}. This suggests that the δ-Bi_2O_3 phase, which is typically stable only at high temperatures, is stabilized at room temperature through strong interfacial interaction in the constructed Bi_2O_3/La_2O_3 heterostructure.[19] Therefore, Bi_2O_3 in Bi_2O_3/La_2O_3 is recognized as the δ-Bi_2O_3 phase.

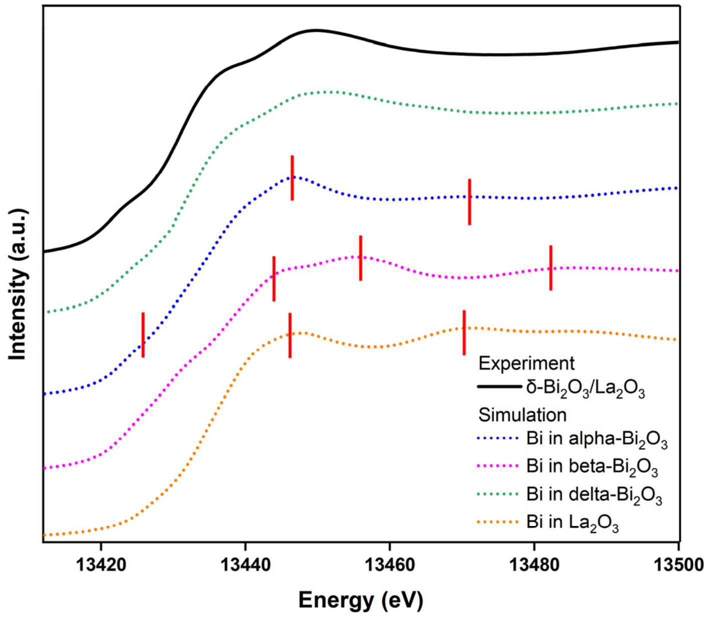

Fig. R32 | Comparison of the experimental Bi L_3-edge XANES spectrum of the Bi_2O_3/La_2O_3 sample with simulated spectra for various Bi_2O_3 phases and Bi-doped La_2O_3 models.

[FIGURE REDACTED]

Fig. R33 | (a) Raman spectra of δ-Bi_2O_3/La_2O_3 samples compared to reference spectra of α- and β-Bi_2O_3. (b) Raman spectra cited from the Fig. 4b in *J. Alloys Compd.* 2017, 723, 520. (c) Raman spectra of δ-Bi_2O_3 thin films cited from the Fig. 1b in *Appl. Phys. Lett.* 2005, 87, 231916. Noted, based on previous literatures, in δ-Bi_2O_3 (fluorite type structure), the vibration around the 620 cm^{-1} is related to the active photon T_{2g}, which is caused by the symmetrical oscillation between O–M–O (oxygen–metal–oxygen) and the width of the peak referred to the intrinsic disorder of the structure.

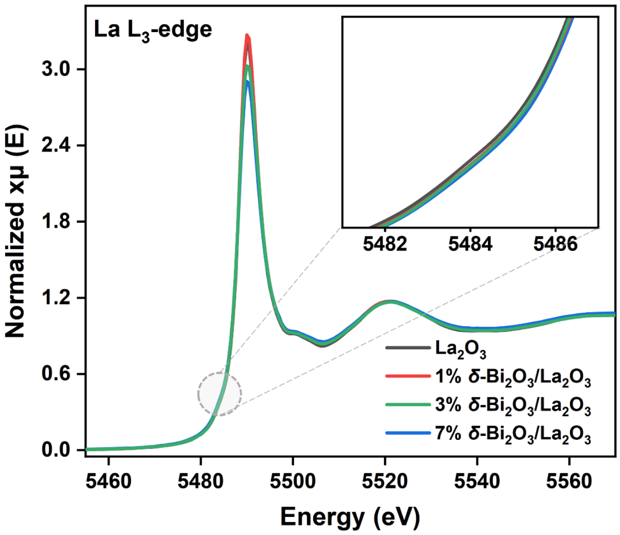

Fig. R34 | La L_3-edge XANES spectra over pure La_2O_3 and δ-Bi_2O_3/La_2O_3 samples.

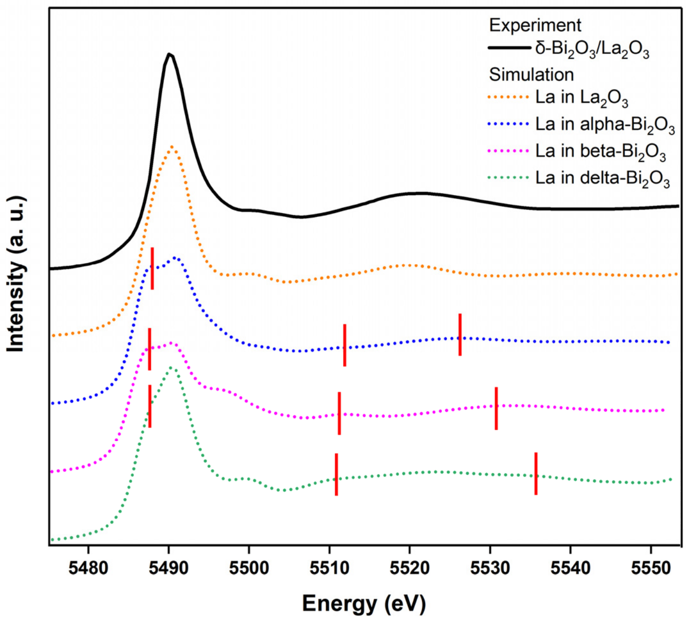

Fig. R35 | Comparison of the experimental La L_3-edge XANES spectrum of the δ-Bi_2O_3/La_2O_3 sample with simulated spectra for various La-doped Bi_2O_3 models and pristine La_2O_3.

Second, we thank the reviewer's meticulous observations regarding (i) the discrepancy between our XRD data and the δ-Bi_2O_3 reference standard and (ii) the apparent contradiction in detecting diffraction peaks from the sub-2 nm particles. These points are invaluable for strengthening the structural foundation of our work. We agree with your assessment regarding the X-ray amorphous nature of such ultrasmall δ-Bi_2O_3 particles. Nanoparticles smaller than 2 nm exhibit low crystallinity and limited coherent scattering volume, which leads to broadened XRD diffraction peaks that are often indistinguishable or even undetectable. Our previous aberration-corrected HAADF-STEM images (Fig. 1d, e and Figs. S9-11) have confirmed that Bi_2O_3 is dispersed on the La_2O_3 support in the form of sub-2 nm particles in the 1%, 3%, and 7% δ-Bi_2O_3/La_2O_3 catalysts. Therefore, the XRD patterns presented in Supplementary Fig. 7 were likely misassigned due to our negligence and do not correspond to the 1%, 3%, and 7% Bi_2O_3/La_2O_3 samples. Actually, during catalyst preparation, we employed two calcination temperatures: 800 °C was used to synthesize samples in which the sub-2 nm particles are dispersed on the La_2O_3 support, while 900 °C was applied to prepare a control sample with significantly grown particles (~20 nm, Supplementary Fig. 43) to investigate the effect of particle size on catalytic performance. Therefore, the strong diffraction peaks in Supplementary Fig. 7 most likely originate from the control sample calcined at 900 °C, in which δ-Bi_2O_3 particles sintered to ~20 nm, rather than from sub-2 nm dispersed samples prepared at 800 °C. Additionally, regarding the mismatch between the observed XRD peaks and reference patterns, we note a systematic shift toward lower 2θ angles in both pure La_2O_3 and Bi_2O_3/La_2O_3 samples.

This is likely due to instrumental zero-point drift, i.e., insufficient calibration. To address this issue, we have recalibrated the XRD instrument using standard silicon powder and included the corrected XRD patterns in **Fig. R36**. The XRD patterns of the Bi_2O_3/La_2O_3 sample show good agreement with the reference pattern of δ-Bi_2O_3 (JCPDS No. 01-071-0466), particularly in the positions of the (111) and (200) reflections, suggesting the presence of the delta phase. We are very sorry for such negligence and checked these issues in the revised manuscript and SI.

To correct the misassignment in the original submission, we now provide the XRD patterns of the desired 1%, 3%, and 7% δ-Bi_2O_3/La_2O_3 samples calcined at 800 °C (see **Fig. R37**). As the reviewer pointed out, the sub-2 nm Bi_2O_3 nanoparticles lack long-range crystalline order, and consequently, no Bragg reflections attributable to Bi_2O_3 are discernible in the XRD pattern. To further study the effect of loading, following your third suggestion, we prepared 10% and 15% δ-Bi_2O_3/La_2O_3 samples with higher Bi loadings. The HAADF-STEM imaging reveals the highly active sub-2 nm δ-Bi_2O_3 particles begin to agglomerate into larger particles (~8 nm) as the Bi loading increases, resulting in a coexistence of sub-2 nm and ~8 nm particles (**Fig. R38**). Accordingly, the XRD patterns of the 10% and 15% Bi_2O_3/La_2O_3 samples gradually exhibit two broad diffraction features centered at $2\theta \approx 27.8°$ and 32.5° (Fig. R37), which can be indexed to the (111) and (200) planes of δ-Bi_2O_3 (JCPDS No. 01-071-0466), consistent with particle growth to ~8 nm as observed by HAADF-STEM. The slight positive shift of all XRD diffraction peaks of δ-Bi_2O_3/La_2O_3 compared to pure La_2O_3 indicates a contraction in the lattice spacing (Fig. R37b).

Figs. R37 and R38 have been added as Supplementary Figs. 13 and 12 in the revised Supporting Information, respectively.

Revised parts in the manuscript:

Page 4, lines 140–145

On the contrary, the XRD patterns (Supplementary Fig. 13) reveal that the diffraction peaks of the Bi_2O_3/La_2O_3 samples can be well indexed to cubic δ-Bi_2O_3 (JCPDS: 01-071-0466) and hexagonal La_2O_3 ((JCPDS: 01-074-2430), with no α-Bi_2O_3 phase detected. Notably, the sub-2 nm Bi_2O_3 nanoparticles in the 1%, 3%, and 7% Bi_2O_3/La_2O_3 samples lack long-range crystalline order, and consequently, no Bragg reflections attributable to Bi_2O_3 are discernible in the XRD pattern.

Pages 3–4, lines 125–130

In the 10% and 15% Bi_2O_3/La_2O_3 samples, the highly active sub-2 nm Bi_2O_3 particles begin to agglomerate into larger particles (~8 nm) as the Bi loading increases, resulting in a coexistence

of sub-2 nm and ~8 nm particles. This agglomeration reduces the density of highly active sub-nanoparticles and may diminish the Bi_2O_3/La_2O_3 interfacial area, both of which are critical for improving catalytic activity and stability.

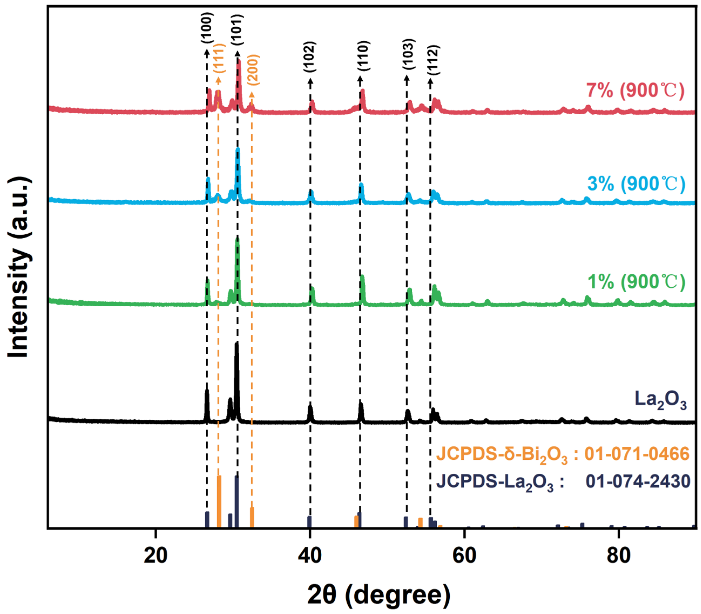

Fig. R36 | XRD patterns of 1%, 3%, and 7% δ-Bi_2O_3/La_2O_3 calcined at 900 °C, used as control samples.

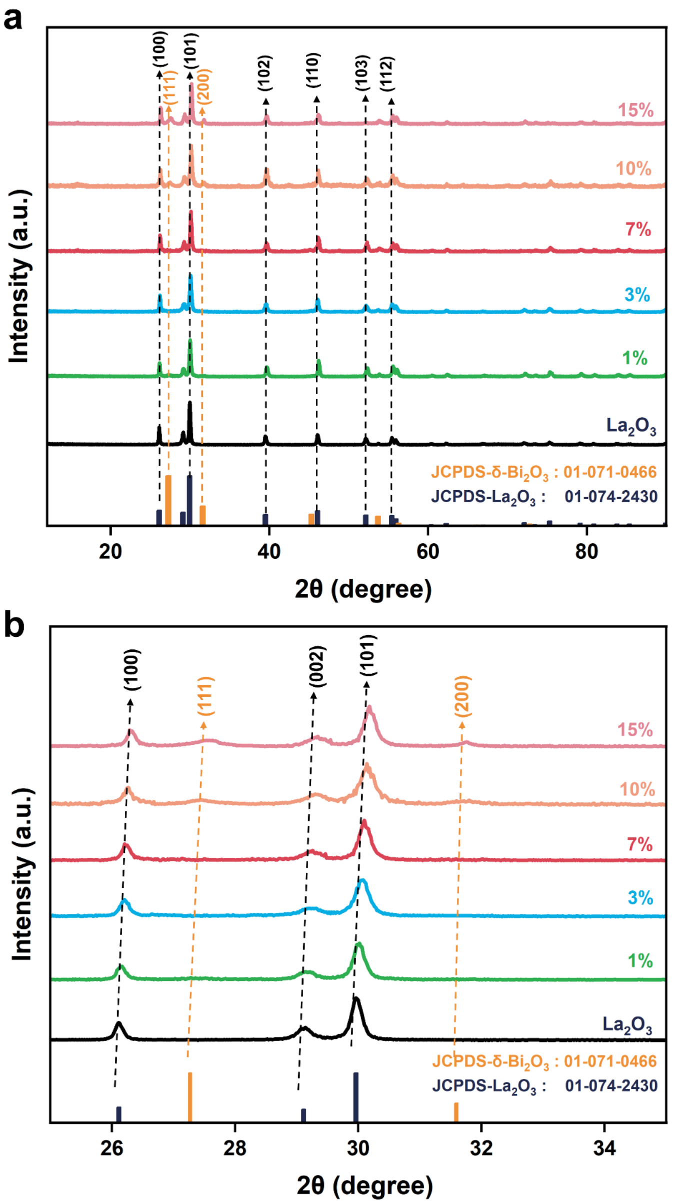

Fig. R37 | (a) XRD patterns of the desired 1%, 3%, 7%, 10%, and 15% δ-Bi_2O_3/La_2O_3 samples calcined at 800 °C, with an enlarged 25–35° region shown in (b).

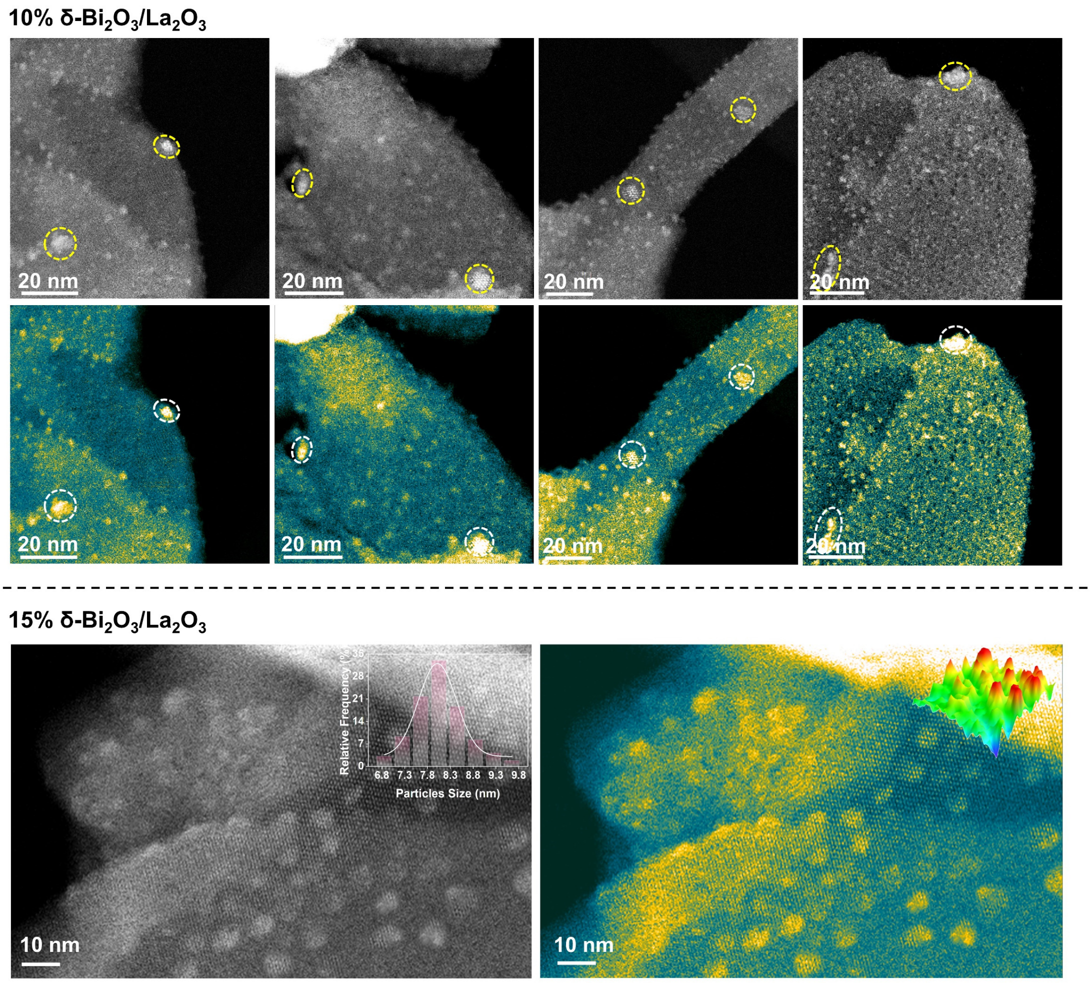

Fig. R38 | Aberration-corrected HAADF-STEM images as well as the corresponding elemental distribution intensity maps of 10% δ-Bi_2O_3/La_2O_3 and 15% δ-Bi_2O_3/La_2O_3, the inset is corresponding size distribution.

(2) Comparison of FE between different catalysts is confusing. Authors state that "7% δ-Bi_2O_3/La_2O_3 exhibits a FE_{HCOO-} of ≥ 90% over a wide potential interval of –0.65 to –1.25 V_{RHE} with a maximum FE_{HCOO-} of 97.8% at –0.95 V_{RHE}, which is superior to that of pure δ-Bi_2O_3 powder with a FE_{HCOO-} of 79.3% at –1.05 V_{RHE}." However, a few lines below authors say that for pure δ-Bi_2O_3 "FEs of $HCOO^-$ distinctly drop from 88.5% to 45.6% only after electrolysis for 30 h at –0.95 V_{RHE}." From this I conclude that that for pure Bi_2O_3 FE to formate is higher and –0.95 V (88.5%) than at –1.05 V (79.3%). Why then the FE for La_2O_3-supported catalyst at –0.95 V is compared with that of pure Bi_2O_3 at –0.95 V?

[Reply]: We thank the reviewer for this valuable comment, which helps clarify the performance metrics and avoids potential confusion. To enable a fairer comparison, the original statement on page 13, "...which is superior to that of pure δ-Bi_2O_3 powder with a FE_{HCOO-} of 79.3% at –1.05 V_{RHE}..." has been revised to "...which is superior to that of pure Bi_2O_3 powder with a FE_{HCOO-} of 88.5% at –0.95 V vs. RHE..." in the revised manuscript. We are sorry for such

negligence and checked these issues in the revised manuscript and Supporting Information.

(3) Authors also compare performance of catalysts with different amounts of bismuth oxide (1%, 3% and 7%). The best performance was observed for the catalyst with the highest bismuth loading. What will happen, if one would further increase the bismuth loading? Would this catalyst show even better catalytic properties?

[Reply]: We appreciate this insightful comment. As suggested, we have prepared two additional δ-Bi_2O_3/La_2O_3 catalysts with higher Bi loadings (9.6 and 15.2 at.%, determined by ICP-AES), denoted as 10% and 15% δ-Bi_2O_3/La_2O_3, respectively, and evaluated their CO_2 electroreduction performance. As shown in the supplementary aberration-corrected HAADF-STEM images (**Fig. R38**), the sub-2 nm, highly active δ-Bi_2O_3 particles begin to agglomerate into larger particles (~8 nm or larger) with increasing Bi loading. This agglomeration reduces the density of highly active sites and diminishes the metal–support interface, both of which are crucial for catalytic activity and stability. As shown in **Fig. R39**, the 10% and 15% δ-Bi_2O_3/La_2O_3 catalysts exhibit lower overall formate Faradaic efficiency (FE_{HCOO^-}) and poorer operational stability than the 7% δ-Bi_2O_3/La_2O_3, suggesting that 7% is near the optimum. Specifically, the 10% δ-Bi_2O_3/La_2O_3 delivers a FE_{HCOO^-} of ~91.5% and retains stable performance for ~175 h, whereas the 15% sample shows a lower FE_{HCOO^-} (89.3%) and deactivates after ~154 h under identical conditions. Furthermore, we supplemented *in-situ* Raman and FT-IR spectroscopy on the 10% and 15% δ-Bi_2O_3/La_2O_3 samples to explore the origin of the performance degradation caused by higher Bi loading. *In-situ* Raman spectroscopy reveal that the δ-Bi_2O_3 phase in the high-loading (10% and 15%) samples exhibits lower stability compared to that in the 7% sample, as evidenced by a pronounced positive shift in the onset potential for its reduction to metallic Bi. As shown in **Fig. R40**, two new characteristic peaks at ~70 and ~90 cm^{-1}, assigned to the E_g and A_{1g} vibrational modes of metallic Bi^0, appear at approximately −1.45 V vs. RHE for the 10% δ-Bi_2O_3/La_2O_3 and at −1.25 V vs. RHE for the 15% δ-Bi_2O_3/La_2O_3, respectively. Accordingly, the potentiodynamic ATR-SEIRA spectra reveal that the signal intensity of the ν_s(OCO) band of bidentate *HCOO intermediate at ~1310 cm^{-1} on the 10% and 15% δ-Bi_2O_3/La_2O_3 samples shows a pronounced decreasing trend at more negative potentials (**Figs. R41**, **R42**). In contrast, the ν_s(OCO) signal on the 7% δ-Bi_2O_3/La_2O_3 sample remains largely constant over the potential range of −0.85 to −1.15 V vs. RHE (Supplementary Fig. 63), suggesting sustained stabilization of the key *HCOO intermediate. Therefore, 7% represents an optimal balance between active site density and dispersion. We further acquired time-resolved ATR-SEIRA spectra to gain deeper insight into the long-term CO_2RR process. At a fixed potential of −0.95 V vs. RHE, the contour plots show that the ν_s(OCO) bands of the adsorbed bidentate *HCOO intermediate on

the 10% and 15% δ-Bi_2O_3/La_2O_3 samples gradually decrease in intensity over the 900-min CO_2RR (**Fig. R43**). The above results reveal the unique advantage of the highly dispersed sub-2 nm δ-Bi_2O_3 in the optimal 7% δ-Bi_2O_3/La_2O_3 catalyst, which enables stable electrochemical CO_2 reduction to formate.

Figs R39, R40a, R41, and R42 have been added as Supplementary Figs. 42, 53, 62, and 63 in the revised Supporting Information, respectively.

Revised parts in the manuscript:

Page 8, lines 281–287

...while the 10% and 15% δ-Bi_2O_3/La_2O_3 catalysts exhibit lower formate Faradaic efficiency and poorer operational stability due to the aggregation of δ-Bi_2O_3 sub-nanoparticles (Supplementary Fig. 42). Specifically, the 10% δ-Bi_2O_3/La_2O_3 catalyst delivers a formate Faradaic efficiency of ~91.5% and maintains stable performance for ~175 h, whereas the 15% sample exhibits a lower efficiency (~89.3%) and deactivates after ~154 h. These results suggest that a 7% Bi loading may represent an optimal balance between active site density and dispersion.

Page 12, lines 401–407

Notably, in the high-loading (10% and 15%) samples, the Bi–O and Bi–O–La signals could not be maintained over such a wide potential window. As shown in Fig. 4c and Supplementary Fig. 53, the characteristic peaks of metallic Bi−Bi at ~70 and ~95 cm^{-1} appeared at −1.45 V vs. RHE for the 10% δ-Bi_2O_3/La_2O_3 sample and at −1.25 V vs. RHE for the 15% δ-Bi_2O_3/La_2O_3 sample, respectively. The Raman results again highlight the distinct advantage of well-dispersed sub-2 nm δ-Bi_2O_3 particles in the 7% δ-Bi_2O_3/La_2O_3 catalyst in enhancing the SMOSI effect.

Page 13, lines 461–464

Notably, the signal intensity of the v_s(OCO) band on the 10% and 15% δ-Bi_2O_3/La_2O_3 samples shows a pronounced decreasing trend at more negative potentials (Supplementary Figs. 62, 63), indicating that the agglomeration of sub-nanoparticles induced by high Bi loading reduces the catalytic stability.

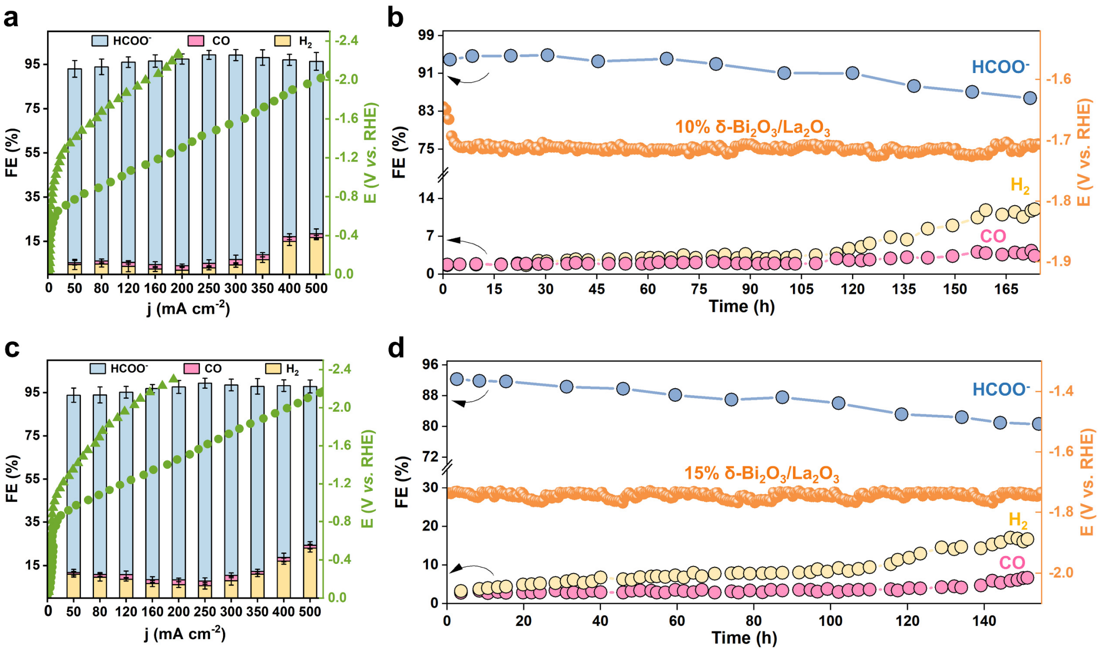

Fig. R39 | LSV curves under CO_2-saturated (circular) and Ar-saturated (triangle) 0.5 M $KHCO_3$ solution, and corresponding FEs of various products as a function of current density for (a) 10% δ-Bi_2O_3/La_2O_3 and (c) 15% δ-Bi_2O_3/La_2O_3 catalysts. Long-term chronopotentiometric curves and corresponding FEs of (b) 10% δ-Bi_2O_3/La_2O_3 and (d) 15% δ-Bi_2O_3/La_2O_3 at 350 mA cm^{-2} in a flow cell.

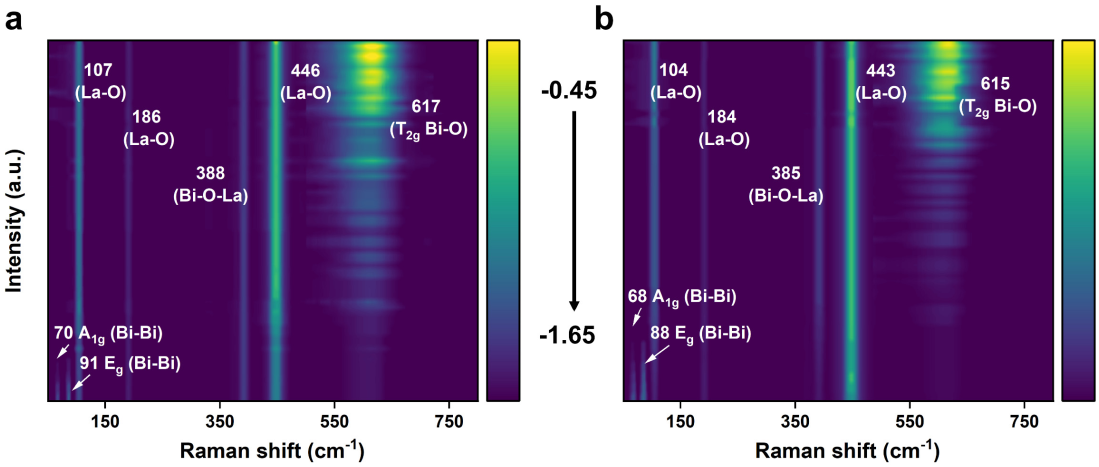

Fig. R40 | In-situ Raman spectra monitoring the surface variation of (a) 10% δ-Bi_2O_3/La_2O_3 and (b) 15% δ-Bi_2O_3/La_2O_3 under different applied potentials.

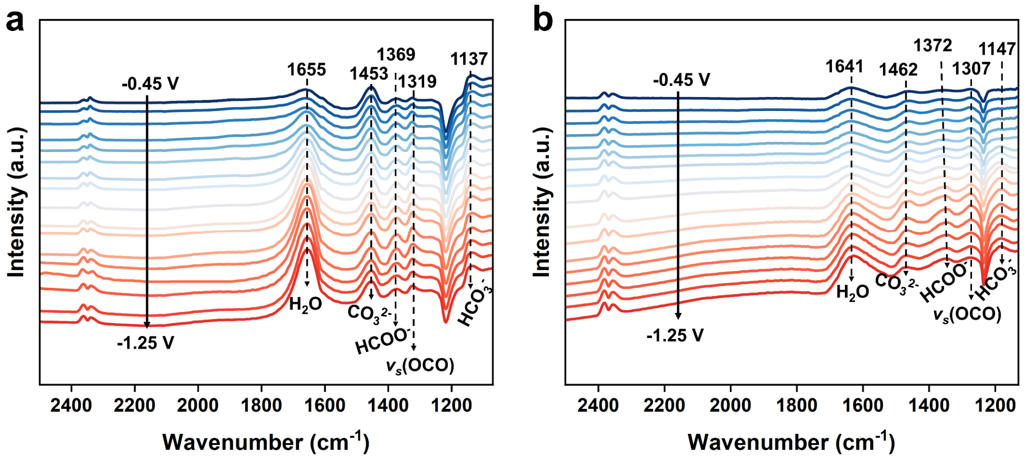

Fig. R41 | *In-situ* ATR-SEIRA spectra of (a) 10% and (b) 15% δ-Bi_2O_3/La_2O_3 recorded at different applied potentials in CO_2-saturated 0.5 M $KHCO_3$ solution, with the spectrum at −0.25 V vs. RHE as the reference.

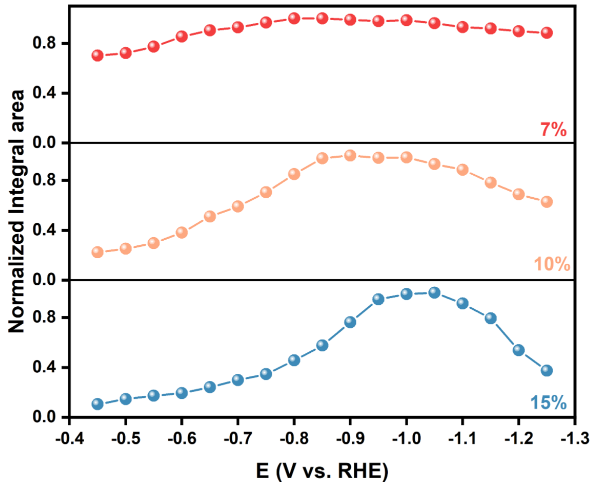

Fig. R42 | Potential-dependent integrated intensities of the ν_s(OCO) band for the 7%, 10%, and 15% δ-Bi_2O_3/La_2O_3 samples.

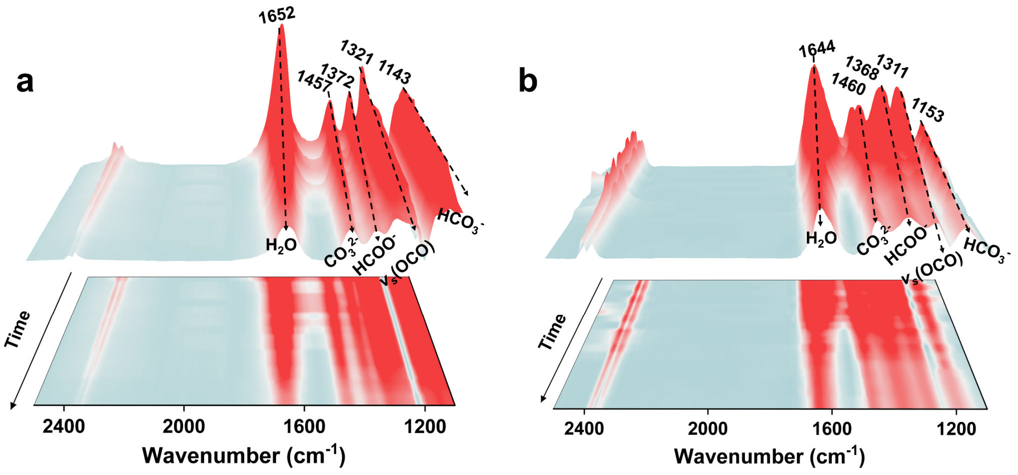

Fig. R43 | Time-evolved ATR-SEIRAS color contour map for (a) 10% and (b) 15% δ-Bi_2O_3/La_2O_3 at a fixed potential of −0.95 V vs. RHE.

(4) When discussing XAS data, authors state that "The X-ray absorption near edge structure (XANES) spectra of the Bi L_3-edge (Supplementary Fig. 32a) reveal that the oxidation state of Bi in the initial δ-Bi_2O_3 is below +3 as its absorption edge shifts to lower energy than that of the Bi_2O_3 standard." However, this is likely not true. The position of absorption edge depends not only on the chemical state of Bi, but also on its local environment, in particular, on interatomic distances. In particular, the shift of the absorption edge to higher energies is likely a result of shortened Bi-O bonds, observed also in XAS and XRD, but is not related changes in oxidation state.

[Reply]: This is a very good comment. On one hand, we agree with the reviewer's comment that the position of absorption edge depends not only on the oxidation state of metal but also on its local coordination environment (e.g., bond lengths or symmetry). In many cases, an increase in the metal oxidation state is often accompanied by a shortening of the metal-oxygen bond length. On the other hand, in X-ray absorption spectroscopy (XAS), the absorption edge energy generally increases with the oxidation state of the absorbing metal. This trend arises because a higher oxidation state reduces the valence electron density, weakening the shielding of the nuclear charge. As a result, core electrons undergo a stronger effective nuclear attraction, requiring higher photon energy for excitation to unoccupied states—manifested as a shift of the absorption edge to higher energy. On the contrary, reduction (lower oxidation state) increases electron density, enhances shielding, and shifts the edge to lower energy. This correlation, extensively validated across diverse materials systems, is now regarded as one of the most reliable criteria for oxidation state assignment in XAS analysis (e.g., *Chem. Soc. Rev.* 2024, 53, 11850; *Chem. Rev.* 2023, 123, 10750; *Nature* 2025, 644, 668; *Nat. Mater.* 2024, 23, 1695). For example, a review on synchrotron radiation analysis techniques states: "*Due to the influence of*

the valence electron distribution on the nuclear electron energy, the absorption edge energy in XANES is highly responsive to the electronic structure of the target elements and it can vary by several eVs merely due to the valence state of the atom changing by a single electron. Therefore, the position shift of the absorption edge typically correlates directly with alterations in the oxidation states and charge transfer of the materials (cited from *Angew. Chem. Int. Ed.* 2024, 63, e202404213)." However, as noted by the reviewer, given the inherent differences in the local coordination environment (e.g., bond lengths and symmetry) between our δ-Bi_2O_3 sample and the α-Bi_2O_3 reference standard, the previous interpretation—that a lower-energy absorption edge shift implies a Bi oxidation state below +3—is inaccurate. We have therefore removed the XANES comparison shown in Supplementary Fig. 32 and the corresponding description: "*the oxidation state of Bi in pure Bi_2O_3 is below +3 as its absorption edge shifts to lower energy than that of the Bi_2O_3 standard*." in the revised manuscript. Notably, in operando XANES studies, it is common practice to monitor oxidation state changes via shifts in the absorption edge energy of a given sample as a function of applied potential and electrolysis time. Accordingly, we have revised the relevant discussion to focus on the evolution of pure Bi_2O_3 sample and the δ-Bi_2O_3/La_2O_3 heterostructure under these operational conditions.

Revised parts in the manuscript:

Pages 9–10, lines 336–371

For pure Bi_2O_3, as the potential is scanned from the open-circuit potential (OCP) to −1.05 V vs. RHE, the Bi L_3-edge absorption edge progressively shifts to lower energy and toward the Bi foil reference (Fig. 3a), signifying a continuous decrease in the Bi oxidation state. The Fourier transform (FT) k^2-weighted extended X-ray absorption fine structure (EXAFS) spectra reveal that the dominant Bi–O scattering peak in R-space shifts from ~1.60 Å at OCP to ~1.67 Å at −0.95 V vs. RHE (Fig. 3b), accompanied by a decrease in the Bi–O coordination number from ~3.2 to ~2.1 (Supplementary Table 5 and Supplementary Fig. 47). Additionally, new peaks corresponding to Bi–Bi scattering emerge at ~2.56 Å and ~3.11 Å in the FT-EXAFS spectrum at −1.05 V vs. RHE, suggesting the formation of metallic Bi. This is further confirmed by the appearance of characteristic Bi–Bi features in the wavelet transform (WT) spectra (Supplementary Fig. 48). In contrast, the Bi L_3-edge absorption energy of 7% δ-Bi_2O_3/La_2O_3 decreases slightly with applied potentials (Fig. 3c), reflecting a relatively stable Bi valence state under operating conditions. Moreover, the average Bi L_3-edge energy of 7% δ-Bi_2O_3/La_2O_3 is approximately 0.9 eV higher than that of Bi_2O_3 reference (Fig. 3c), indicative of enhanced electron deficiency at Bi sites and supporting the presence of Bi_2O_{3+x} moiety with a highly oxidized local structure. This effect arises from the formed interfacial Bi–O–La bonding, which

polarizes the Bi–O bonds and redistributes electron density. Consequently, the Bi–O coordination environment in δ-Bi_2O_3 is perturbed by adjacent La atoms at the heterointerface, leading to reduced electron density around Bi.[37, 38] This electron-deficient state is consistent with the positive binding energy shift observed in the Bi 4f XPS spectra (Supplementary Fig. 15b). These experimental observations are further rationalized by DFT calculations presented in the following section, which quantify the interfacial charge transfer and orbital hybridization. Additionally, the EXAFS fitting results (Supplementary Table 6 and Supplementary Fig. 49) reveal a shorter average Bi–O bond length in 7% δ-Bi_2O_3/La_2O_3 (~2.10 Å) compared to pure Bi_2O_3 (~2.15 Å), indicating that the interphase interaction between La_2O_3 and δ-Bi_2O_3 effectively contracts the Bi−O bonds. This bond contraction suggests an enhanced local structural rigidity, which contributes to improve the stability under operational CO_2RR conditions. As shown in Fig. 3d, when the potential is swept from OCP to −1.15 V vs. RHE, the Bi–O coordination number decreases slightly from ~4.9 to ~4.3 and remains stable. Concurrently, the peak position of the first Bi–O coordination shell in R-space exhibits negligible shift, and no Bi–Bi scattering features are detected in the EXAFS spectra. Furthermore, an extended electrolysis experiment is performed at −1.15 V vs. RHE for 150 minutes to assess the robustness of the Bi–O bonds in the δ-Bi_2O_3/La_2O_3. Throughout this process, the Bi L_3-edge XANES spectra exhibit only a minor energy shift (Fig. 3e), and the Bi–O coordination number undergoes a slight change from ~4.3 to ~4.1 (Fig. 3f, Supplementary Fig. 50 and Supplementary Table 7).

Reviewer:3

The manuscript titled 'Stabilizing Sub-2 nm δ-Bi_2O_3 via Strong Lanthanide-Oxide-Support Interaction for Durable CO_2 Electroreduction to Formate Beyond 200 Hours' presents intriguing experimental results and a potentially impactful heterojunction catalyst for CO_2RR. However, the reviewer finds that the theoretical (DFT) section suffers from significant clarity, consistency, and interpretability issues, which undermine the mechanistic claims. Thus, major revision is required before further consideration. Specific concerns are detailed below:

[Reply]: We sincerely thank the reviewer for the positive assessment of our experimental results and the potential impact of our work, as well as for the insightful and constructive feedback regarding the theoretical interpretation. We agree that the clarity and presentation of the DFT calculations in the original manuscript were insufficient, which hindered the interpretability of our mechanistic conclusions. In response, we have thoroughly revised the theoretical section to address these concerns. More discussions can be seen in the subsequent point-by-point responses or the revised main text and Supporting Information. Overall, we hope that the

supplementary computational data and expanded discussion have strengthened the mechanistic interpretation and enhanced the clarity and robustness of this manuscript.

(1) The description of oxygen atom migration is contradictory. The main text states: "as several oxygen atoms transfer from the first δ-Bi_2O_3 layer to the second" and simultaneously, "Specifically, one oxygen atom moves from the first layer to the second." This inconsistency must be resolved: How many oxygen atoms migrate in the model used?

[Reply]: We sincerely thank the reviewer for identifying this inconsistency in our description of oxygen migration, which was not clearly demonstrated in our previous manuscript. To build a reasonable heterojunction model, the δ-Bi_2O_3(111) layer was cut and loaded on the La_2O_3(001) surface. As shown in **Fig. R44a**, there are three O layers (each with 3 oxygen atoms) in the δ-Bi_2O_3 phase, with Bi atoms at the bottom and O atoms at the top. For the La_2O_3 support, there are four O atoms at the surface. The initial structure was then optimized using DFT method and the final heterojunction structure was shown in **Fig. R44b**. Compared to the initial structure, the structural relaxation due to the strong interfacial interactions leads to significant atomic rearrangements, particularly among the oxygen atoms in the top two Bi_2O_3 layer. Specifically, one oxygen atom in the 2nd O layer migrates into the 3rd O layer, and simultaneously, three oxygen atoms in the 1st layer migrate into the 2nd layer. The newly supplemented charge density difference plot shows electron accumulation in the La_2O_3 phase and electron depletion in the δ-Bi_2O_3 phase (**Fig. R45**), suggesting an obvious charge transfer and redistribution between the two phases. In δ-Bi_2O_3 region of the δ-Bi_2O_3/La_2O_3 heterostructure, the average Bader charge on Bi atoms increases from +1.66 |e| in the topmost (surface) layer to +2.03 |e| in the subsurface (interface) layer, indicating more electrons transfer to neighboring O and La atoms. This electrostatic gradient drives the spontaneous migration of surface oxygen atoms toward the more positively charged Bi sites in the subsurface (interface) layer. As a result, this oxygen migration and rearrangement leads to the contraction of the supported δ-Bi_2O_3 phase, as shown in Fig. 5a, which is further supported by the experimental HRTEM (Figs. 1f, g) and XAS analyses (Supplementary Table 6). In addition, the structural relaxation leads to the formation of interfacial Bi−O−La bonds, which were detected by Raman spectroscopy as a distinct vibrational mode at ~390 cm^{-1}, indicating a strong interaction between the δ-Bi_2O_3 and La_2O_3 phases. The strong interaction at the heterojunction interface is further supported by the *p-d* hybridization between Bi and La atoms (Supplementary Fig. 71) and the crystal orbital bond index (COBI) analysis (Supplementary Fig. 72). We apologize for the confusion caused by the original ambiguous phrasing and appreciate the reviewer's careful inspection.

Figs R44 and R45 have been added as Supplementary Figs. 68 and 70 in the revised Supporting

Information, respectively.

Revised parts in the manuscript:

Page 16, lines 527–529

Specifically, one oxygen atom in the second O layer migrates into the third O layer, and simultaneously, three oxygen atoms in the first layer migrate into the second layer.

Pages 16–17, lines 556–563

The charge density difference plot shows electron accumulation in the La_2O_3 phase and electron depletion in the δ-Bi_2O_3 phase (Supplementary Fig. 70), demonstrating an obvious interfacial charge transfer and redistribution between the two phases. In δ-Bi_2O_3 region of the δ-Bi_2O_3/La_2O_3 heterostructure, the average Bader charge on Bi atoms increases from +1.66 |e| in the topmost (surface) layer to +2.03 |e| in the subsurface (interface) layer, indicating more electrons transfer to neighboring O and La atoms. This electrostatic gradient facilitates the migration of surface oxygen atoms toward the more positively charged Bi sites in the subsurface (interface) layer.

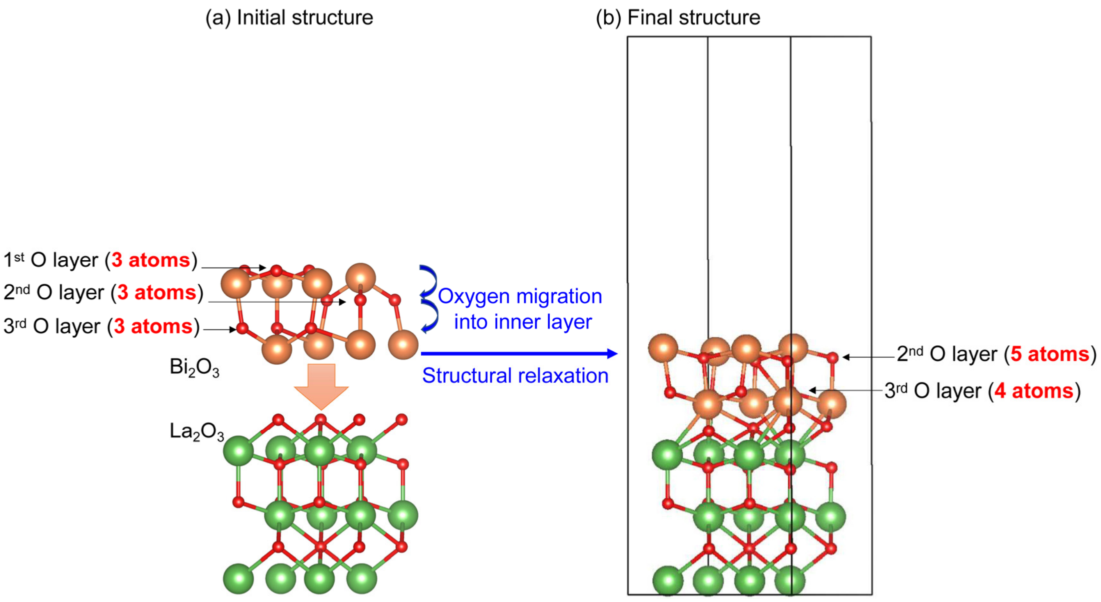

Fig. R44. Scheme of the formation of δ-Bi_2O_3/La_2O_3 model from (a) initial structure to (b) final structure.

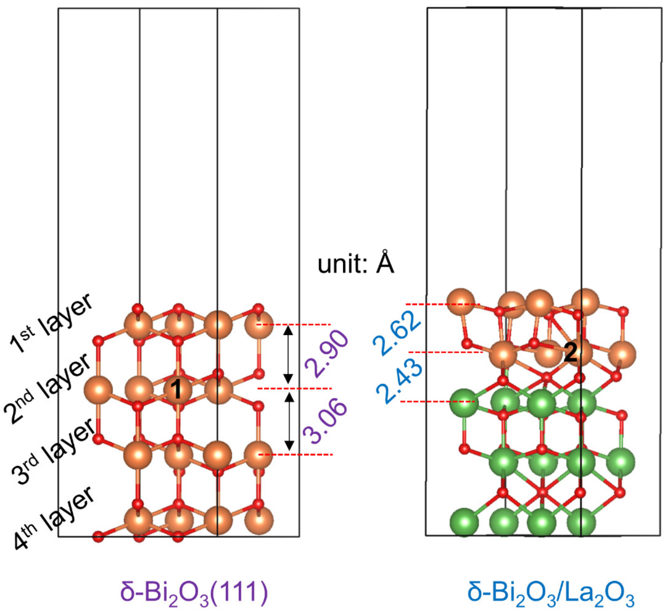

Fig. 5a | Optimized catalyst models for δ-Bi_2O_3(111) and δ-Bi_2O_3/La_2O_3 heterojunction.

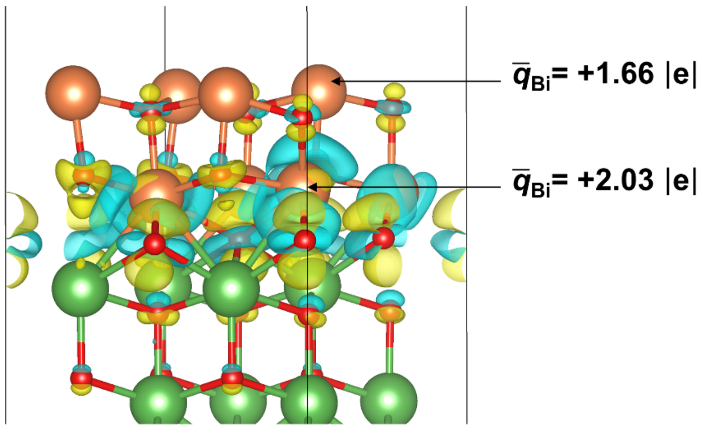

Fig. R45 | The charge density difference plot ($\rho_{CDD} = \rho_{heterojunction} - \rho_{Bi2O3} - \rho_{La2O3}$) is also given for the obtained heterojunction model, with an isosurface value of 0.005 e/Å^3.

(2) The text compares Bi–La and Bi–Bi distances in δ-Bi_2O_3 and the heterojunction, suggesting "Bi–La distances are smaller than in the pure δ-Bi_2O_3(111) surface." However, pure δ-Bi_2O_3 does not contain La, and thus Bi–La bonds cannot exist in that structure. This statement must be revised to reflect meaningful comparisons.

[Reply]: Many thanks for your reminder here. We intended to convey that, in the δ-Bi_2O_3/La_2O_3 heterojunction, the distance between the second (Bi) layer and the third (La) layer is reduced compared to the corresponding interlayer distance (between the second and third Bi layers) in pristine δ-Bi_2O_3(111). However, the original statement was misleading and has been revised for clarity.

Revised parts in the manuscript:

Page 16, lines 531–535

Fig. 5a shows that the interlayer distances of d_{12} (between the 1st and 2nd Bi layers) and d_{23} (between the 2nd Bi layer and 3rd La layers) in the δ-Bi_2O_3/La_2O_3 are 2.62 and 2.43 Å, respectively, which are significantly smaller than the corresponding distances of d_{12} = 2.90 Å (between the 1st and 2nd Bi layers) and d_{23} = 3.06 Å (between the 2nd and 3rd Bi layers) in pristine δ-Bi_2O_3(111).

(3) The manuscript states: "The theoretical results are in excellent agreement with experimental observations..." But it remains vague: Which specific experimental observations do the computational results agree with? Furthermore, the mechanistic origin of oxygen migration and layer contraction is not convincingly explained.

[Reply]: We sincerely thank the reviewer for these insightful comments, which have helped us significantly strengthen the connection between theory and experiment, as well as clarify the mechanistic origin of the observed structural evolution. First, in the original manuscript, the statement "*The theoretical results are in excellent agreement with experimental observations...*" was indeed overly general. Specifically, the interlayer contraction of the Bi_2O_3 layer in the δ-Bi_2O_3/La_2O_3 heterojunction predicted by DFT matches the lattice compression observed in HRTEM (Fig. 1f, g) and the reduced bond length (*R* value) in the Bi–O coordination shell from EXAFS fitting (Supplementary Tables S5 and S6). In addition, the migration of oxygen atoms into the inner layers near the interface leads to the condensation of oxygen atoms. The obtained 5O-4Bi-4O layer near the interface shows an increased O:Bi ratio in the δ-Bi_2O_3/La_2O_3 heterojunction (i.e., the existence of Bi_2O_{3+x} species), which is in agree with the higher absorption edge energy in the XANES spectra relative to that of reference Bi_2O_3 (**Fig. 3c**). Second, the driving force for oxygen migration and the resulting interlayer contraction in the δ-Bi_2O_3/La_2O_3 heterojunction can be elucidated by analyzing the electronic structure. The newly supplemented charge density difference plot shows electron accumulation in the La_2O_3 phase and electron depletion in the δ-Bi_2O_3 phase (**Fig. R45**), demonstrating an obvious interfacial charge transfer and redistribution between the two phases. In the δ-Bi_2O_3 region of the δ-Bi_2O_3/La_2O_3 heterostructure, the average Bader charge on Bi atoms increases from +1.66 |e| in the topmost (surface) layer to +2.03 |e| in the subsurface (interface) layer, indicating more electron transfer to neighboring O and La atoms. This electrostatic gradient facilitates the migration of surface oxygen atoms toward the more positively charged Bi sites in the subsurface (interface) layer.

Revised parts in the manuscript:

Page 16, lines 547–553

The interlayer contraction of the Bi_2O_3 layer in the δ-Bi_2O_3/ La_2O_3 heterojunction predicted by DFT is consistent with the reduced bond length (*R* value) in the Bi–O coordination shell from EXAFS fitting (Supplementary Tables 5, 6). The formation of 5O-4Bi-4O layer near the interface shows an increased O:Bi ratio in the δ-Bi_2O_3/La_2O_3 (i.e., the existence of Bi_2O_{3+x} species), which is in agree with the higher absorption edge energy in the XANES spectra relative to that of reference Bi_2O_3 (Fig. 3c).

Pages 16–17, lines 554–563

The driving force for oxygen migration and the resulting interlayer contraction in the δ-Bi_2O_3/La_2O_3 heterojunction can be elucidated by analyzing the electronic structure. First, strong interfacial charge transfer was identified between the two phases. The charge density difference plot shows electron accumulation in the La_2O_3 phase and electron depletion in the δ-Bi_2O_3 phase (Supplementary Fig. 70), demonstrating an obvious interfacial charge transfer and redistribution between the two phases. In δ-Bi_2O_3 region of the δ-Bi_2O_3/La_2O_3 heterostructure, the average Bader charge on Bi atoms increases from +1.66 |e| in the topmost (surface) layer to +2.03 |e| in the subsurface (interface) layer, indicating more electrons transfer to neighboring O and La atoms. This electrostatic gradient facilitates the migration of surface oxygen atoms toward the more positively charged Bi sites in the subsurface (interface) layer.

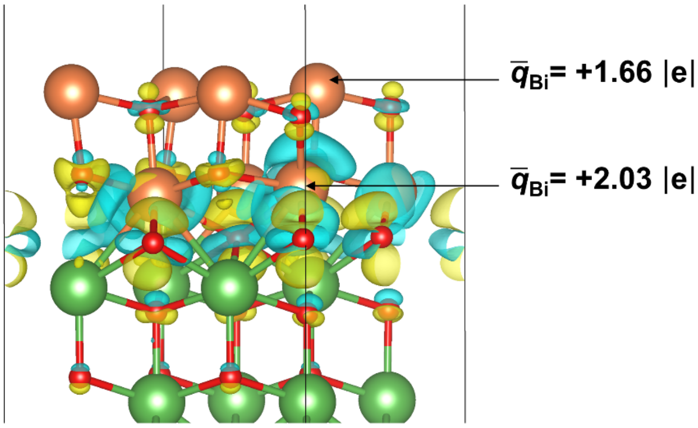

Fig. R45 | The charge density difference plot ($\rho_{CDD} = \rho_{heterojunction} - \rho_{Bi2O3} - \rho_{La2O3}$) is also given for the obtained heterojunction model, with an isosurface value of 0.005 e/Å^3.

(4) The Bader results show a charge increase from 0.65 |e| to 2.53 |e| in δ-Bi_2O_3/La_2O_3. The meaning of "total charge" per structure is unclear. δ-Bi_2O_3 as a neutral solid should not carry net positive charge.

[Reply]: We apologize for the confusing description regarding Bader charge analysis. Both the pristine δ-Bi_2O_3 and the δ-Bi_2O_3/La_2O_3 heterojunction are modeled as charge-neutral systems. To build a reasonable heterojunction model, the δ-Bi_2O_3(111) layer was cut and loaded on the La_2O_3(001) surface (Fig. 5a). Therefore, our original intention was to highlight that interfacial electron transfer is more pronounced in the heterojunction than in pristine δ-Bi_2O_3. Specifically, Bader charge analysis reveals that the top two Bi_2O_3 layers transfers approximately 2.53 |e| to the bottom two La_2O_3 layer in the δ-Bi_2O_3/La_2O_3 heterojunction, whereas the top two Bi_2O_3 layers only transfers approximately 0.65 |e| to the bottom two Bi_2O_3 layer in pristine δ-Bi_2O_3. However, as noted by the reviewer, Bader charges generally refer to atomic charges. To avoid any misunderstanding, we have removed the statement "*The Bader charge of the top two Bi_2O_3 layers is 0.65 |e| and 2.53 |e| in pristine δ-Bi_2O_3 and the δ-Bi_2O_3/La_2O_3 heterojunction, respectively*" in the revised manuscript, and we now describe the average Bader charges on Bi atoms in specific atomic layers, as detailed in our response to *Comment* #3 and Fig. R45.

Revised parts in the manuscript:

Pages 16–17, lines 556–563

The charge density difference plot shows electron accumulation in the La_2O_3 phase and electron depletion in the δ-Bi_2O_3 phase (Supplementary Fig. 70), demonstrating an obvious interfacial charge transfer and redistribution between the two phases. In δ-Bi_2O_3 region of the δ-Bi_2O_3/La_2O_3 heterostructure, the average Bader charge on Bi atoms increases from +1.66 |e| in the topmost (surface) layer to +2.03 |e| in the subsurface (interface) layer, indicating more electrons transfer to neighboring O and La atoms. This electrostatic gradient facilitates the migration of surface oxygen atoms toward the more positively charged Bi sites in the subsurface (interface) layer.

(5) The following sentence is internally contradictory: "The process is endothermic, with an adsorption energy of −2.96 eV..." A negative adsorption energy indicates an exothermic process, not endothermic. This appears to be a typographical or conceptual error, and must be corrected.

[Reply]: We thank the reviewer for identifying this typographical error. The text has been corrected accordingly in the revised manuscript (page 17): "*The process is exothermic, with an adsorption energy of −2.96 eV...*".

Revised parts in the manuscript:

Page 17, lines 588–590

The process is exothermic, with an adsorption energy of −2.96 eV (Supplementary Fig. 73), indicating that it is thermodynamically favorable for the heterojunction to be covered with oxygen.

(6) The structure used for HER and CO_2RR studies (oxygen-molecule adsorbed δ-Bi_2O_3/La_2O_3-1 model) lacks sufficient justification: Is this structure relevant and stable under operating potentials? Surface Pourbaix diagram could be calculated to help understand this. Could surface oxygen atoms, rather than the interface, dominate the activity difference between δ-Bi_2O_3 and δ-Bi_2O_3/La_2O_3-1 model observed?

[Reply]: We sincerely appreciate the insightful comments and suggestions. Indeed, the stability of the catalyst model under operating potentials is critical and thus must be rigorously justified. As suggested, surface Pourbaix diagrams with respect to electrode potential versus standard hydrogen electrode (U_{SHE}) and pH have been plotted for both the δ-Bi_2O_3/La_2O_3-1 and δ-Bi_2O_3 models. Considering that in an aqueous electrochemical environment, surface-adsorbed oxygen can be protonated, leading to surface hydroxylation (i.e., forming a termination state covered with hydroxyl (–OH) groups). To more accurately reflect the realistic surface structure under operating conditions, we have therefore included hydroxylated surface configurations in our computational models. As shown in **Fig. R46**, the monohydroxylated surface (denoted as δ-Bi_2O_3/La_2O_3-2) is thermodynamically more stable than both the oxygen-adsorbed surface (i.e., δ-Bi_2O_3/La_2O_3-1 model in the original manuscript) and dihydroxylated surfaces with two –OH groups (denoted as δ-Bi_2O_3/La_2O_3-3) under experimentally relevant operating conditions. Specifically, in CO_2-saturated 0.5 M $KHCO_3$ solution (pH ≈ 7.2), within the potential window optimal for formate production (U_{RHE} = −0.75 V to −1.05 V, corresponding to U_{SHE} = −1.17 V to −1.47 V), the δ-Bi_2O_3/La_2O_3-2 model exhibits the lowest free energy among all considered terminations, making it the thermodynamically preferred structure for catalytic simulations under CO_2RR conditions. In contrast, for the δ-Bi_2O_3 model, the Pourbaix diagram shows that the pristine surface is more stable than the hydroxylated models in the potential window of U_{RHE} = −0.75 V to −1.05 V (U_{SHE} = −1.17 V to −1.47 V), and thus the pristine δ-Bi_2O_3 model is a reasonable model for catalytic simulations under CO_2RR conditions.

In fact, the ATR-SEIRA spectra in **Fig. 4i, j** of the original manuscript have demonstrated that the differences in H_2O adsorption and activation on pure Bi_2O_3 and δ-Bi_2O_3/La_2O_3 electrodes. The v(O−H) stretching band of H_2O in the range of 3000 to 3800 cm^{-1} can be deconvoluted into three gaussian peaks, corresponding to: (i) 4-coordinated hydrogen-bonded

water (4-HB·H_2O), observed at ~3250 cm^{-1} (gray); (ii) 2-coordinated hydrogen-bonded water (2-HB·H_2O), observed at ~3400 cm^{-1} (red); and (iii) K^+-hydrated water with weak hydrogen bonding ($K^+(H_2O)_n$, high wavenumber component of ~3580 cm^{-1}, blue), respectively. The $K^+(H_2O)_n$ signal is primarily present on the δ-Bi_2O_3/La_2O_3 electrodes, and the $K^+(H_2O)_n$ population increases as the potential shifts negatively, which indicates that the introduction of La_2O_3 support as well as the induced SMOSI effect promotes interfacial water enrichment and facilitates H_2O adsorption and activation at the heterojunction catalyst. Therefore, compared to δ-Bi_2O_3, the δ-Bi_2O_3/La_2O_3 heterojunction surface is more readily protonated, facilitating the formation of surface-bound OH* species, in agreement with the stability trends predicted by the Pourbaix diagram. We further studied the projected densities of states (PDOS) of surface oxygen atoms in the δ-Bi_2O_3 and δ-Bi_2O_3/La_2O_3 (**Fig. R47**). The δ-Bi_2O_3/La_2O_3 exhibits an obvious upshift in the O 2p-band centers (E_p) of −2.09 eV, compared to −2.58 eV in pristine δ-Bi_2O_3. This shift suggests that the strong oxide–support interaction reduces the occupancy of O-centered antibonding states, thereby enhancing the surface protonation propensity. The adsorption and activation behavior of water molecules on both δ-Bi_2O_3/La_2O_3-1 and δ-Bi_2O_3 surfaces are also compared. Theoretical results in **Fig. 5d** of the original manuscript show that the water dissociation is more favorable on δ-Bi_2O_3/La_2O_3-1 with a free energy of −1.16 eV, compared to −0.53 eV for δ-Bi_2O_3. This implies that the introduction of La_2O_3 can efficiently increase the *H coverage, which not only facilitates the surface protonation but also enhances the hydrogenation kinetics for formate production on δ-Bi_2O_3 sites.

Therefore, the δ-Bi_2O_3/La_2O_3-2 model is then used to investigate its CO_2RR and HER activities. The recalculated CO_2RR and HER pathways are shown in **Fig. R48**. For pristine δ-Bi_2O_3, the formation of *HCOO from *CO_2 intermediate, with an energy barrier of 0.83 eV, is identified as the rate-determining step (RDS). For the δ-Bi_2O_3/La_2O_3-2, the conversion of *CO_2 to *HCOO is also the RDS, but with a lower energy barrier of 0.15 eV, indicating a superior CO_2RR activity toward formate production on the heterojunction. Furthermore, the energy barriers for the competitive HER are 1.35 and 1.78 eV for δ-Bi_2O_3 and δ-Bi_2O_3/La_2O_3-2, respectively, both of which are higher than the barriers of RDS for *HCOO formation in the CO_2RR, indicating that the HER is less favorable on both catalysts. Moreover, the higher energy barrier of 1.78 eV indicates that the HER is kinetically more sluggish on the δ-Bi_2O_3/La_2O_3-2 heterojunction. Additionally, we also calculated the CO_2RR pathway on the monohydroxylated δ-Bi_2O_3 model. **Fig. R49** shows that the energy barrier for the RDS (*CO_2 → *OCHO) is significantly higher than that of the δ-Bi_2O_3/La_2O_3-2 model and is comparable to that of pristine δ-Bi_2O_3. This result further confirms that the enhanced CO_2RR activity of δ-Bi_2O_3/La_2O_3 primarily arises from the heterointerface, rather than from the intrinsic properties of surface-

adsorbed oxygen atoms. To further elucidate the effect of strong oxide–support interaction on the properties of catalysts, we supplemented H_2 temperature programmed reduction (H_2-TPR) measurements on the δ-Bi_2O_3/La_2O_3 and a physically mixed Bi_2O_3-La_2O_3 sample. The H_2-TPR profiles show that the high-temperature peak at 616.5 °C is attributed to the reduction of La_2O_3 species, while a broad medium-temperature feature (350–450 °C) corresponds to the reduction of Bi_2O_3 species (**Fig. R50**). An obvious shift of Bi_2O_3 reduction peak to a higher temperature region (414.5 and 454.6 °C) is observed in the δ-Bi_2O_3/La_2O_3, indicating that the Bi_2O_3 species in this heterostructure are more difficult to be reduced, which contributes to maintaining high CO_2RR selectivity under operating conditions. These critical results and discussions have been added to the revised manuscript and the Supporting Information.

Figs R46, R47 and R50 have been added as Supplementary Figs. 76, 77, and 58 in the revised Supporting Information, respectively. Fig. R48 has been updated as Fig. 5c in the revised main text.

Revised parts in the manuscript:

Pages 17–18, lines 599–625

Before the oxygen-molecule adsorbed δ-Bi_2O_3/La_2O_3-1 model is used in CO_2RR and HER studies, its stability under realistic operating conditions must be evaluated. To this end, surface Pourbaix diagrams with respect to electrode potential versus standard hydrogen electrode (U_{SHE}) and pH are plotted for both the δ-Bi_2O_3/La_2O_3-1 and δ-Bi_2O_3 models. Considering that in an aqueous electrochemical environment, surface-adsorbed oxygen can be protonated, leading to surface hydroxylation (i.e., forming a termination state covered with hydroxyl (–OH) groups). To more accurately reflect the realistic surface structure, we also included hydroxylated surface configurations in our computational models. As shown in Supplementary Fig. 76, the monohydroxylated surface (denoted as δ-Bi_2O_3/La_2O_3-2) is thermodynamically more stable than both the oxygen-adsorbed surface (i.e., δ-Bi_2O_3/La_2O_3-1 model in the original manuscript) and dihydroxylated surfaces with two –OH groups (denoted as δ-Bi_2O_3/La_2O_3-3) under experimentally relevant operating conditions. Specifically, in CO_2-saturated 0.5 M $KHCO_3$ solution (pH ≈ 7.2), within the potential window optimal for formate production (U_{RHE} = −0.75 V to −1.05 V, corresponding to U_{SHE} = −1.17 V to −1.47 V), the δ-Bi_2O_3/La_2O_3-2 model exhibits the lowest free energy among all considered terminations, making it the thermodynamically preferred structure for catalytic simulations under CO_2RR conditions. In contrast, for the δ-Bi_2O_3 model, the Pourbaix diagram shows that the pristine surface is more stable than the hydroxylated models in the potential window of U_{RHE} = −0.75 V to −1.05 V (U_{SHE} = −1.17 V to −1.47 V), and thus the pristine δ-Bi_2O_3 model is a reasonable model for

catalytic simulations under CO_2RR conditions. In fact, the aforementioned SEIRA spectra in Figs. 4i, j have shown that, compared to pure Bi_2O_3, the δ-Bi_2O_3/La_2O_3 heterojunction surface is more readily protonated, facilitating the formation of surface-bound OH* species. We further studied the projected densities of states (PDOS) of surface oxygen atoms in the δ-Bi_2O_3 and δ-Bi_2O_3/La_2O_3 (Supplementary Fig. 77). The δ-Bi_2O_3/La_2O_3 exhibits an obvious upshift in the O 2p-band centers (E_p) of −2.09 eV, compared to −2.58 eV in pristine δ-Bi_2O_3. This shift suggests that the strong oxide–support interaction reduces the occupancy of O-centered antibonding states, thereby enhancing the surface protonation propensity.

Page 12, lines 427–435

To further confirm the strong oxide–support interaction on the δ-Bi_2O_3/La_2O_3 heterojunction, we conducted H_2 temperature programmed reduction (H_2-TPR) measurements on the 7% δ-Bi_2O_3/La_2O_3 and a physically mixed Bi_2O_3-La_2O_3 sample. The H_2-TPR profiles demonstrated that the high-temperature peak at 616.5 °C is attributed to the reduction of La_2O_3 species,[44] while a broad medium-temperature feature (350–450 °C) corresponds to the reduction of Bi_2O_3 species.[45] An obvious shift of Bi_2O_3 reduction peak to a higher temperature region (414.5 and 454.6 °C) is observed on the 7% δ-Bi_2O_3/La_2O_3 (Supplementary Fig. 58), indicating that the Bi_2O_3 species in this heterostructure are more difficult to be reduced.

Page 18, lines 633–642

Complete reaction pathways are calculated and are illustrated in Fig. 5c and Supplementary Figs. 79, 80. For pristine δ-Bi_2O_3, the formation of *HCOO from *CO_2 intermediate, with an energy barrier of 0.83 eV, is identified as the rate-determining step (RDS). For δ-Bi_2O_3/La_2O_3-2, the conversion of *CO_2 to *HCOO is also the RDS, but with a lower energy barrier of 0.15 eV, indicating a superior CO_2RR activity toward formate production on the heterojunction. Additionally, the energy barriers for the competitive HER are 1.35 and 1.78 eV for δ-Bi_2O_3 and δ-Bi_2O_3/La_2O_3-2, respectively, both of which are higher than the barriers of RDS for *HCOO formation in the CO_2RR, indicating that the HER is less favorable on both catalysts. Moreover, the higher energy barrier of 1.78 eV indicates that the HER is kinetically more sluggish on the δ-Bi_2O_3/La_2O_3-2 heterojunction.

Revised parts in the Supporting Information.

Page 6, lines 193–210

The Gibbs free energy of CO_2 reduction and hydrogen evolution reactions were calculated based on the computational hydrogen electrode (CHE). The van der Waals interactions were included by Grimme's D3 method.[9] For the HER pathway, the Gibbs free energies Δ*G* were

calculated by the binding energies ΔE with corrections for zero-point energy (ΔE_{ZPE}) and entropy (S), according to

$$\Delta G = \Delta E + \Delta E_{ZPE} - T\Delta S \quad \text{(S1)}$$

Zero-point energy corrections are calculated by summing vibrational frequencies over all normal modes, and the temperature T was set to 298 K. The Gibbs free energy for the adsorption of *H on the surface was calculated by $\Delta G_{H*} = \Delta E_{H*}$ +0.30 eV.[10]

The surface Pourbaix diagrams of Bi_2O_3, Bi_2O_3/La_2O_3 heterojunction, and their hydrogenated surfaces with different H coverage, were plotted under the relevant U_{SHE} ($U_{RHE} = U_{SHE} + 0.059*pH$) and pH. The hydrogenation process of the O* species on the surfaces of catalysts were assumed to occur with the following step:

$$O* + H^+ + e^- \rightarrow OH* \quad \text{(S2)}$$

The Gibbs free energy of eq (S2) was is obtained

$$\Delta G = \Delta E + \Delta E_{ZPE} - T\Delta S \quad \text{(S3)}$$

The effects of potential U_{SHE} and pH were included by:

$$\Delta G' = \Delta G - k_b T \ln 10 \times \mathrm{pH} - eU_{\mathrm{SHE}} \quad \text{(S4)}$$

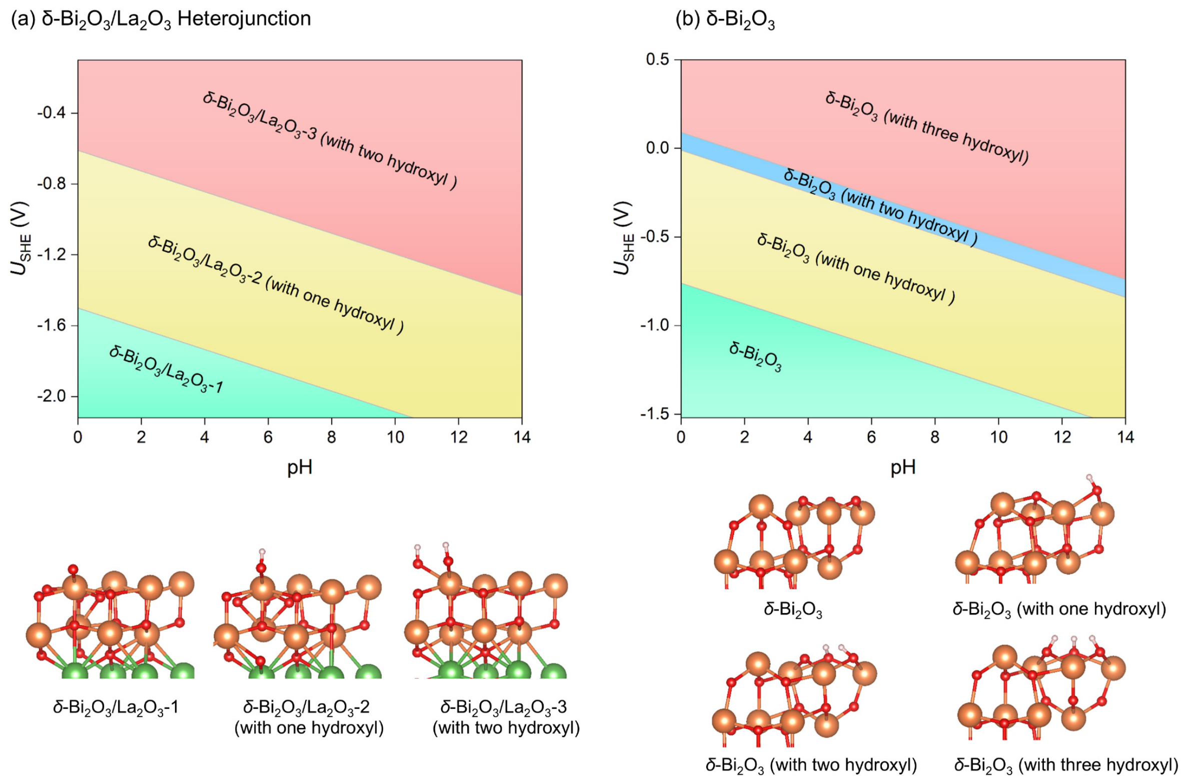

Fig. R46 | Pourbaix diagrams of the δ-Bi_2O_3/La_2O_3 heterojunction, pristine δ-Bi_2O_3, and their hydroxylated surface terminations.

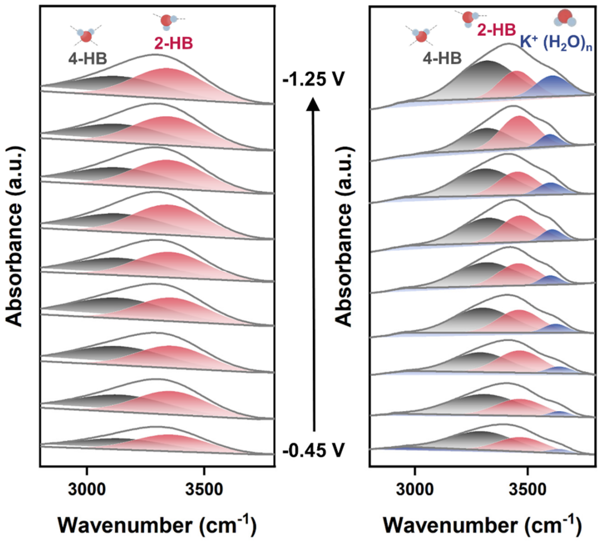

Figs. 4j, k | Gaussian deconvolution of the O–H stretching region (3000–3800 cm^{-1}) into three components for (i) Bi_2O_3 and (j) δ-Bi_2O_3/La_2O_3, derived from ATR-SEIRA spectra.

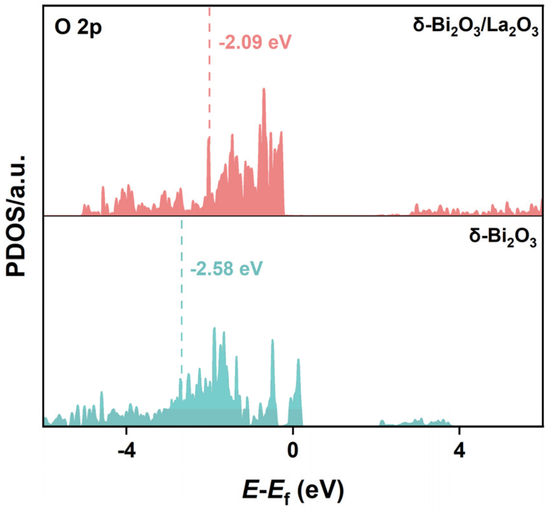

Fig. R47 | Projected density of states (PDOS) of surface oxygen atoms in δ-Bi_2O_3 and δ-Bi_2O_3/La_2O_3.

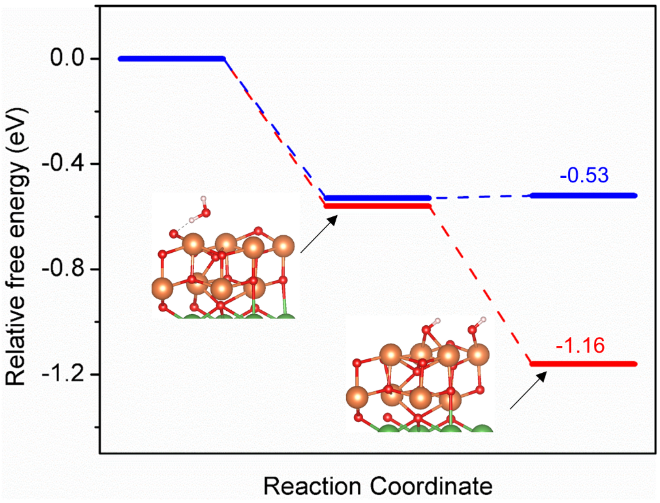

Fig. 5d | Comparison of H_2O adsorption and dissociation pathways on the surfaces of δ-Bi_2O_3/La_2O_3-1 and δ-Bi_2O_3 models. La: green; Bi: yellow; C: brown; O: red; H: pink.

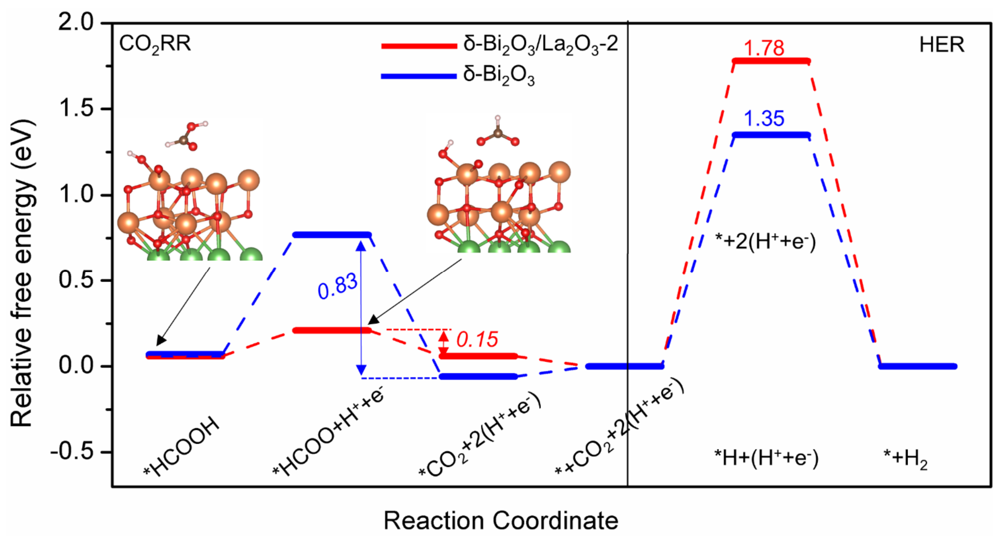

Fig. R48 | Comparison of the CO_2RR and HER pathways on the δ-Bi_2O_3/La_2O_3-2 and δ-Bi_2O_3 models.

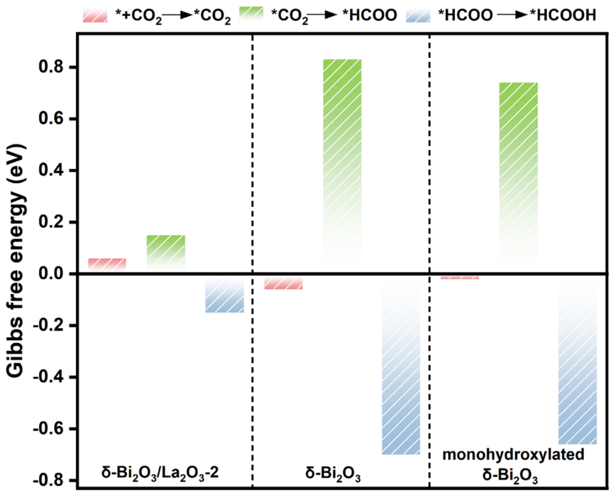

Fig. R49 | Comparison of the Gibbs free energy for CO_2RR to HCOOH on δ-Bi_2O_3/La_2O_3-2, pristine δ-Bi_2O_3, and monohydroxylated δ-Bi_2O_3 models.

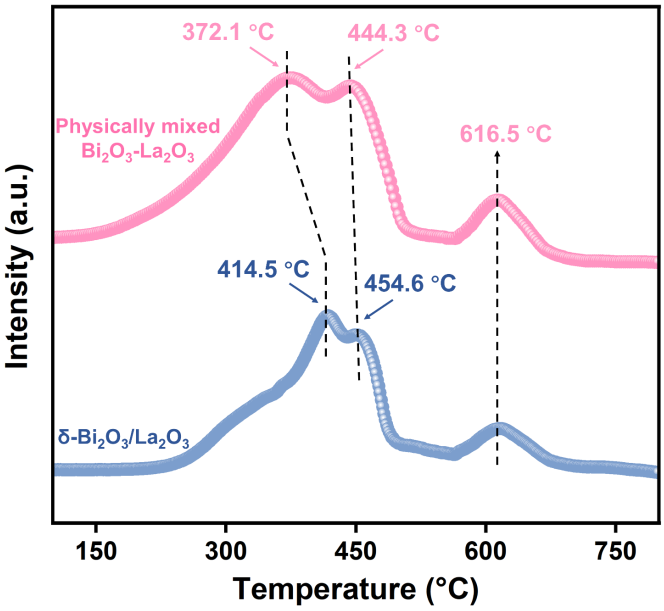

Fig. R50 | H_2-TPR profiles of the δ-Bi_2O_3/La_2O_3 and a physically mixed Bi_2O_3-La_2O_3 sample.

Minor Comments

"Bader charge analyses reveals" → should be "analysis reveals"

"is the thermodynamically favorable" → should be "thermodynamically favorable"

[Reply]: Many thanks for your reminder. The corresponding revisions have been made into the revised manuscript (**Page 1, line 26; Page 17, line 589**).

Point-by-point Responses to the Reviewers' Comments

We thank the editors and referees for their time and comments on our manuscript. These comments are all valuable and helpful for revising and improving our paper. In the following, by supplementing more details, we provide a point-by-point response to their comments. The author's responses are marked in red, and all changes have been highlighted in yellow in the revised main text and the revised SI files.

Reviewer: 1

Comments:

The authors have addressed all the proposed questions. I have no more concerns. The manuscript is ready for publication.

[Reply]: Thanks for your insightful comments, which have significantly improved the quality of this work.

Reviewer: 2

Comments:

During the revision, authors have addressed my previous comments, and paper can be published now, if the following is addressed: Authors have now included Figures S23 and S24, showing XANES simulation results. Please provide the details of these simulations. Which code was used? What were the values of the most important simulation parameters? How the structures used for simulations were constructed?

[Reply]: Thanks for the reviewers' recognition of our work. The theoretical XANES spectra were simulated using the ab initio multiple-scattering code FEFF 9.0 [Ref: *Phys. Chem. Chem. Phys.*, 2010, 12, 5503-5513], which uses spherical muffin-tin potentials and the extended continuum approximation to treat all XANES features in terms of the scattering of continuum photoelectrons.

Hedin-Lundqvist exchange correlation potential was used, and the radiuses for full multiple scattering calculations and self-consistent calculations were set to 8.0 and 5.0 Å, respectively. The maximum value of automatic overlapping of all muffin-tins was set to 1.3 to reduce the effects of potential discontinuities. All other parameters were set to default values in FEFF 9.0.

The structural models used for simulations of La_2O_3, alpha-Bi_2O_3 and beta-Bi_2O_3 were constructed based on the experimental data from Inorganic Crystal Structure Database (ICSD). For delta-Bi_2O_3, there are different proposed models related to the arrangements of oxygen vacancies, therefore DFT optimized structures were used as input for FEFF calculations. All clusters used for calculations contains more than 120 atoms and all possible absorption sites have been considered. Substitution doping (Bi occupying at La

sites in La_2O_3 and La occupying at Bi sites in Bi_2O_3) has been simulated to determine whether the large-scale interdiffusion of Bi and La atoms occurs. The corresponding structural models have been provided in the revised Figures S23 and S24. These details of XANES simulations have been added to the "***XANES Simulations***" section of the revised Supporting Information (Pages 5–6, lines 170–186).

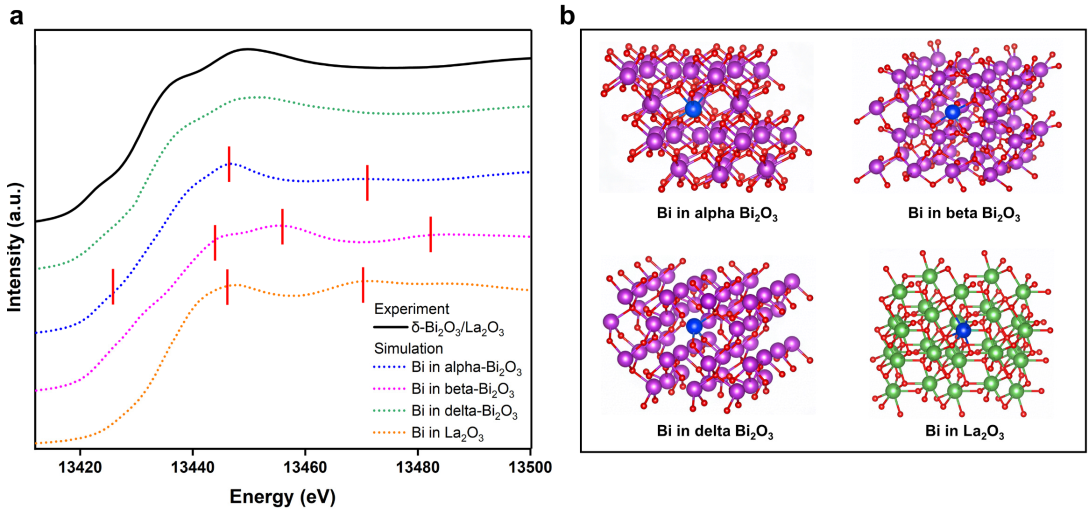

Supplementary Fig. 23 | (a) Comparison of the experimental Bi L_3-edge XANES spectrum of the δ-Bi_2O_3/La_2O_3 sample with simulated spectra for various Bi_2O_3 phases and Bi-doped La_2O_3 models. (b) Sketches of structural model used for XANES simulations.

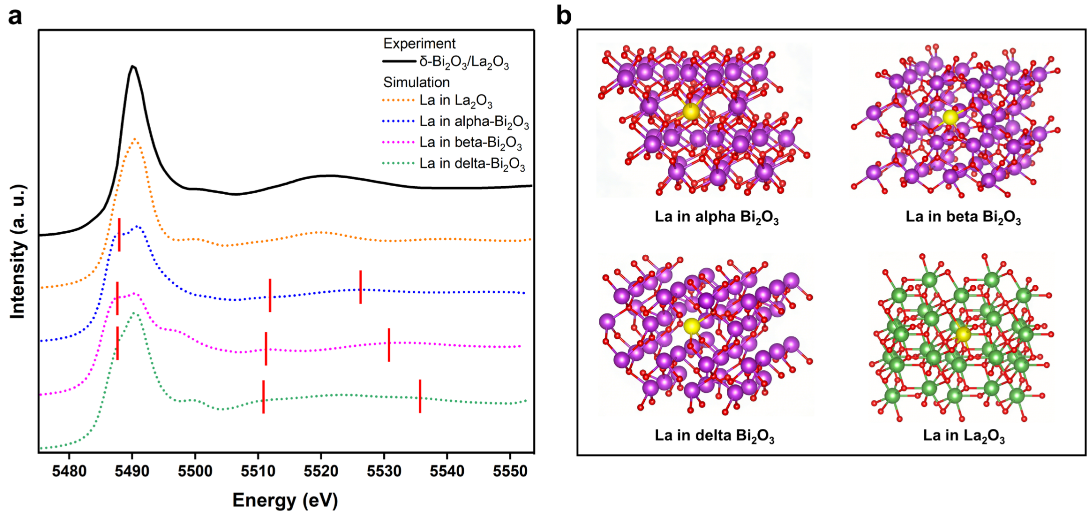

Supplementary Fig. 24 | (a) Comparison of the experimental La L_3-edge XANES spectrum of the δ-Bi_2O_3/La_2O_3 sample with simulated spectra for various Bi_2O_3 phases and La-doped La_2O_3 models. (b) Sketches of structural model used for XANES simulations.

Reviewer: 3

Comments:

The authors have addressed the reviewer's previous concerns regarding the DFT calculations. The inconsistencies in the description of oxygen migration have been clarified, and the structural comparisons have been revised to avoid misleading statements. The connection between theoretical results and experimental observations is now more clearly articulated, and the stability and relevance of the computational model under operating conditions are better justified. Overall, the revisions have significantly improved the clarity and rigor of the theoretical part, and the reviewer believes the manuscript is now suitable for publication.

[Reply]: Thanks for your insightful comments, which have significantly improved the quality of this work.
